# The type II RAF inhibitor tovorafenib in relapsed/refractory pediatric low-grade glioma: the phase 2 FIREFLY-1 trial

**DOI:** 10.1038/s41591-023-02668-y

**Published:** 2023-11-17

**Authors:** Lindsay B. Kilburn, Dong-Anh Khuong-Quang, Jordan R. Hansford, Daniel Landi, Jasper van der Lugt, Sarah E. S. Leary, Pablo Hernáiz Driever, Simon Bailey, Sébastien Perreault, Geoffrey McCowage, Angela J. Waanders, David S. Ziegler, Olaf Witt, Patricia A. Baxter, Hyoung Jin Kang, Timothy E. Hassall, Jung Woo Han, Darren Hargrave, Andrea T. Franson, Michal Yalon Oren, Helen Toledano, Valérie Larouche, Cassie Kline, Mohamed S. Abdelbaki, Nada Jabado, Nicholas G. Gottardo, Nicolas U. Gerber, Nicholas S. Whipple, Devorah Segal, Susan N. Chi, Liat Oren, Enrica E. K. Tan, Sabine Mueller, Izzy Cornelio, Lisa McLeod, Xin Zhao, Ashley Walter, Daniel Da Costa, Peter Manley, Samuel C. Blackman, Roger J. Packer, Karsten Nysom

**Affiliations:** 1 Children’s National Hospital, Washington, DC USA; 2 Children’s Cancer Centre, Royal Children’s Hospital Melbourne, Melbourne, Victoria Australia; 3 Michael Rice Centre for Hematology and Oncology, Women’s and Children’s Hospital, Adelaide, South Australia Australia; 4 South Australia Health and Medical Research Institute, Adelaide, Australia; South Australian Immunogenomics Cancer Institute, University of Adelaide, Adelaide, South Australia Australia; 5 Duke University, Durham, NC USA; 6 Princess Máxima Center for Pediatric Oncology, Utrecht, The Netherlands; 7 Cancer and Blood Disorders Center, Seattle Children’s, Seattle, WA USA; 8 Charité Universitätsmedizin Berlin, corporate member of Freie Universität Berlin and Humboldt-Universität Berlin, German HIT-LOGGIC-Registry for LGG in Children and Adolescents, Berlin, Germany; 9 Great North Children’s Hospital and Newcastle University Centre for Cancer, Newcastle-upon-Tyne, UK; 10 CHU Sainte-Justine, Université de Montréal, Montréal, Quebec Canada; 11 Sydney Children’s Hospitals Network, Westmead, New South Wales Australia; 12 Ann & Robert H. Lurie Children’s Hospital, Chicago, IL USA; 13 Kids Cancer Centre, Sydney Children’s Hospital, Randwick, New South Wales Australia; 14 Children’s Cancer Institute, Lowy Cancer Research Centre, University of New South Wales, Sydney, New South Wales Australia; 15 School of Clinical Medicine, University of New South Wales, Sydney, New South Wales Australia; 16 Hopp Children’s Cancer Center Heidelberg (KiTZ), Heidelberg, Germany; 17 Clinical Cooperation Unit, Pediatric Oncology, German Cancer Research Center (DKFZ), Heidelberg, Germany; 18 Department of Pediatric Oncology, Hematology, Immunology and Pulmonology, Heidelberg University Hospital, Heidelberg, Germany; 19 German Cancer Consortium (DKTK), Heidelberg, Germany; 20 National Center for Tumor Diseases (NCT), Heidelberg, Germany; 21 Texas Children’s Cancer Center, Texas Children’s Hospital, Baylor College of Medicine, Houston, TX USA; 22 Department of Pediatrics, Seoul National University College of Medicine, Seoul National University Cancer Research Institute, Seoul National University Children’s Hospital, Seoul, Republic of Korea; 23 Children’s Health Queensland Hospital and Health Service, South Brisbane, QLD Australia; 24 Severance Hospital, Yonsei University Health System, Seoul, Republic of Korea; 25 UCL Great Ormond Street Institute of Child Health and Great Ormond Street Hospital for Children, London, UK; 26 C.S. Mott Children’s Hospital, University of Michigan Medical School, Ann Arbor, MI USA; 27 Pediatric Hemato-Oncology, Sheba Medical Center, Ramat Gan, Israel; 28 Department of Pediatric Oncology, Schneider Children’s Medical Center, Petach Tikva, and Faculty of Medicine, Tel Aviv University, Tel Aviv, Israel; 29 Department of Pediatrics, Centre Mère-Enfant Soleil du CHU de Québec-Université Laval, Quebec City, Quebec Canada; 30 Division of Oncology, Department of Pediatrics, Children’s Hospital of Philadelphia, University of Pennsylvania Perelman School of Medicine, Philadelphia, PA USA; 31 Division of Hematology and Oncology, Department of Pediatrics, School of Medicine, Washington University, St. Louis, MO USA; 32 McGill University Health Centre (MUHC), Montreal Children’s Hospital (MCH), Montreal, Quebec Canada; 33 Department of Pediatric and Adolescent Oncology and Hematology, Perth Children’s Hospital, Perth, Australia, and Brain Tumor Research Program, Telethon Kids Cancer Centre, Telethon Kids Institute, University of Western Australia, Perth, Western Australia Australia; 34 Department of Oncology, University Children’s Hospital, Zurich, Switzerland; 35 Primary Children’s Hospital and University of Utah, Salt Lake City, UT USA; 36 NYU Langone Health, New York, NY USA; 37 Pediatric Neuro-Oncology, Department of Pediatrics, Dana-Farber/Boston Children’s Cancer and Blood Disorders Center, Boston, MA USA; 38 Department of Hematology & Oncology, Rambam Healthcare Campus, Haifa, Israel; 39 Haematology/Oncology Service, KK Women’s and Children’s Hospital, Singapore, Singapore; 40 Department of Neurology, Neurosurgery and Pediatrics, University of California, San Francisco, San Francisco, CA USA; 41 Day One Biopharmaceuticals, Brisbane, CA USA; 42 Division of Neurology, Brain Tumor Institute, Center for Neuroscience and Behavioral Medicine, Children’s National Hospital, Washington, DC USA; 43 Department of Pediatrics and Adolescent Medicine, Copenhagen University Hospital – Rigshospitalet, Copenhagen, Denmark

**Keywords:** Paediatric cancer, Targeted therapies, Drug development, Randomized controlled trials, Phase II trials

## Abstract

*BRAF* genomic alterations are the most common oncogenic drivers in pediatric low-grade glioma (pLGG). Arm 1 (*n* = 77) of the ongoing phase 2 FIREFLY-1 (PNOC026) trial investigated the efficacy of the oral, selective, central nervous system–penetrant, type II RAF inhibitor tovorafenib (420 mg m^−^^2^ once weekly; 600 mg maximum) in patients with *BRAF*-altered, relapsed/refractory pLGG. Arm 2 (*n* = 60) is an extension cohort, which provided treatment access for patients with *RAF*-altered pLGG after arm 1 closure. Based on independent review, according to Response Assessment in Neuro-Oncology High-Grade Glioma (RANO-HGG) criteria, the overall response rate (ORR) of 67% met the arm 1 prespecified primary endpoint; median duration of response (DOR) was 16.6 months; and median time to response (TTR) was 3.0 months (secondary endpoints). Other select arm 1 secondary endpoints included ORR, DOR and TTR as assessed by Response Assessment in Pediatric Neuro-Oncology Low-Grade Glioma (RAPNO) criteria and safety (assessed in all treated patients and the primary endpoint for arm 2, *n* = 137). The ORR according to RAPNO criteria (including minor responses) was 51%; median DOR was 13.8 months; and median TTR was 5.3 months. The most common treatment-related adverse events (TRAEs) were hair color changes (76%), elevated creatine phosphokinase (56%) and anemia (49%). Grade ≥3 TRAEs occurred in 42% of patients. Nine (7%) patients had TRAEs leading to discontinuation of tovorafenib. These data indicate that tovorafenib could be an effective therapy for *BRAF*-altered, relapsed/refractory pLGG. ClinicalTrials.gov registration: NCT04775485.

## Abstract (web summary)

In a phase 2 trial, the oral type II RAF inhibitor tovorafenib exhibited an overall response rate of 67% in patients with *BRAF*-altered relapsed/refractory pediatric low-grade glioma.

## Main

Pediatric low-grade glioma (pLGG) is the most common childhood central nervous system (CNS) tumor, representing approximately 30% of pediatric brain tumors^1^. Although considered indolent and potentially curable in certain locations by complete surgical resection^2^, tumors located in critical areas of the brain not amenable to complete resection may induce functional deficits and/or have an aggressive biology. In such cases, therapies are required that may lead to significant morbidity, with disease progression potentially causing further functional deficits. Given the chronic nature of pLGG, children often require several lines of treatment in their first two decades of life^2,3^. Treatment goals include regression or stabilization (at a minimum) of disease and neurologic deficits; avoidance of potential acute and long-term toxicities (especially those limiting cognitive functions); and the ability to participate in age-appropriate activities. Frontline systemic therapy currently consists of chemotherapy, which can achieve overall response rates (ORRs) of around 30% and can provide durable responses in some children^2,4^. However, frequent clinic visits are required; an indwelling catheter is often necessary; and tumors often progress or relapse, necessitating additional treatment^4^. Moreover, in patients receiving chemotherapy, more than 75% may experience grade 3/4 hematologic toxicity; approximately 20% may experience peripheral neuropathy; and, for patients diagnosed in infancy, the number of chemotherapy regimens may be associated with an increased risk of neurodevelopmental disruption^4,5^. Although MEK inhibitors may represent an emerging standard of care (SOC) in the United States for patients with relapsed/refractory disease, these daily medications require fasting before and after dosing, which can be particularly challenging in younger children. Moreover, many patients will progress during or after therapy with these agents. There is currently no global uniform consensus on first-line or second-line/further-line SOC in this setting for most patients. Approved therapies that demonstrate treatment response and balance quality of life, with minimal risk of long-term toxicities, are urgently needed for patients with pLGG.

Most pLGGs are driven by genomic alterations affecting components of the mitogen-activated protein kinase (MAPK)/extracellular signal-regulated kinase (ERK) pathway, which regulates cell proliferation and survival^6,7^. They are often somatic activating alterations of *BRAF*, including *KIAA1549*::*BRAF* gene fusions or BRAF V600E point mutations^8,9^. The frequency of *BRAF* alterations varies across pLGG subtypes, with *KIAA1549*::*BRAF* fusions present in 30–40% of tumors overall and in 70–80% of pilocytic astrocytomas, the most common subtype^9–12^.

A randomized phase 2 trial, which investigated the type I RAF inhibitor dabrafenib plus the MEK inhibitor trametinib versus vincristine and carboplatin as first-line therapy in patients with pLGG with a BRAF V600 mutation, showed the superiority of dabrafenib plus trametinib over vincristine–carboplatin in this setting^13^. The US Food and Drug Administration (FDA) recently approved dabrafenib plus trametinib for first-line treatment of patients 1 year of age and older with a pLGG harboring a BRAF V600E mutation who require systemic therapy^14–16^. However, type I BRAF inhibitors—either alone or in combination with a MEK inhibitor—are not indicated for the treatment of patients with tumors harboring *BRAF* fusions. Type I RAF inhibitors are ineffective in targeting the encoded RAF kinase dimers^17^ and may cause paradoxical upregulation of MAPK pathway signaling, in *BRAF* wild-type tumors or tumors harboring *BRAF* fusions, promoting accelerated tumor growth^18,19^. For patients with tumors harboring *BRAF* fusions, or for tumors that progress on or after dabrafenib and/or trametinib therapy, treatment options are limited. Although MEK inhibitor monotherapy has demonstrated early evidence of efficacy in clinical trials in children with progressive pLGG^20–23^, it is not approved for use in this setting. Phase 3 trials in the frontline setting are ongoing^24^.

Tovorafenib (previously known as DAY101, TAK-580, MLN2480 or BIIB024) is an investigational, oral, selective, CNS-penetrant type II RAF inhibitor in clinical development for the treatment of patients with tumors harboring an activating *RAF* alteration. Studies in murine models have shown that it has potent activity against both oncogenic BRAF fusions, which signal as RAS-independent dimers, and BRAF V600 mutations, which signal as RAS-independent monomers. In contrast to type I BRAF inhibitors, tovorafenib did not induce paradoxical activation of the MAPK pathway in these models^25^. In an adult phase 1 trial, tovorafenib showed single-agent activity in patients with *BRAF*-mutated melanoma naive to RAF and MEK inhibitors^26^. Additionally, anti-tumor activity was reported in a child with a recurrent spindle cell sarcoma harboring a novel *SNX8*::*BRAF* fusion and in three adult patients with melanomas harboring different *BRAF* fusions^27,28^. The phase 1b PNOC014 (NCT03429803) clinical trial evaluated the safety and tolerability of tovorafenib monotherapy across a dose range up to 530 mg m^−^^2^ per week, providing early evidence of anti-tumor activity in patients with *BRAF*-altered relapsed/refractory pLGG, and supported the selected phase 2 dose of 420 mg m^−^^2^ (maximum dose 600 mg) administered orally once per week^29^.

The phase 2 FIREFLY-1 (PNOC026; NCT04775485) trial objective was to evaluate the safety and efficacy of tovorafenib monotherapy in children, adolescents and young adults with *RAF*-altered, relapsed/refractory pLGG or advanced solid tumors. Here we report efficacy outcomes for arm 1 and safety outcomes for arms 1 and 2, which enrolled patients 6 months to 25 years of age with pLGG harboring a known activating *BRAF* (arm 1; registrational) or *RAF* (arm 2; extension) alteration and is the basis for a US New Drug Application. The primary endpoint of overall response rate (ORR) was assessed per independent radiology review committee (IRC) according to Response Assessment in Neuro-Oncology High-Grade Glioma (RANO-HGG) criteria^30^, as these were considered the only validated assessment criteria at the time of trial initiation. The Response Assessment in Pediatric Neuro-Oncology Low-Grade Glioma (RAPNO) criteria had been recently published, so efficacy assessments by RAPNO criteria were included as secondary endpoints due to their relevance for this patient population^31^. Efficacy by RANO-LGG criteria was added as a post hoc exploratory endpoint per regulatory authority request and was prespecified in the updated statistical analysis plan before initiation of the current analysis^32^. Assessment of changes in quality of life and health utilities measures were exploratory objectives. This report presents the efficacy analysis from the evaluable population in arm 1 (enrolled patients who received ≥1 dose of tovorafenib and met the prespecified efficacy analysis criteria per IRC for the relevant radiological assessment criteria) and safety from arms 1 and 2 (safety analysis set) as of a 5 June 2023 datacut.

## Results

### Endpoints

The primary endpoint in arm 1 was the ORR as assessed by the IRC according to RANO-HGG criteria. Secondary endpoints for arm 1 included ORR by RAPNO criteria per IRC; clinical benefit rate (CBR), progression-free survival (PFS), duration of response (DOR) and time to response (TTR), per IRC by RANO-HGG and RAPNO criteria; and safety and tolerability (primary endpoint in arm 2), assessed by the type, frequency and severity of adverse events (AEs) and laboratory abnormalities. Additional planned secondary endpoints not reported in this manuscript are described in detail in the full trial protocol (confidential information redacted) in the Supplementary Information and include pharmacokinetics, the effect of tovorafenib on the corrected QT interval by Fredericia (QTcF) and other electrocardiogram (ECG) parameters, change in visual acuity and concordance of molecular profiling approaches. The characteristics of the three different radiological response assessment criteria used in the assessment of efficacy in the trial are summarized in Extended Data Table 1.

### Patients and disposition

**Fig. 1:**
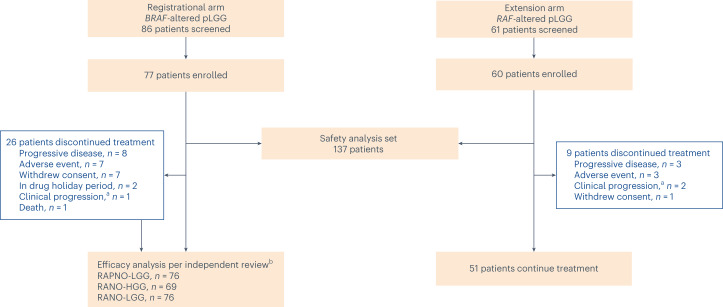
FIREFLY-1 patient disposition/CONSORT diagram. This report presents efficacy data from the evaluable population (enrolled patients who received at least one dose of tovorafenib and met the prespecified efficacy analysis criteria per each radiological assessment method according to the IRC) in arm 1 (registrational) and the safety data from arms 1 and 2 (safety analysis set) as of a 5 June 2023 datacut. Patients were enrolled in arm 2 (extension) after arm 1 had fully accrued and closed for enrollment. ^a^Not radiologically confirmed. ^b^Only patients with measurable disease at baseline per independent review were included.

Between 22 April 2021 and 26 January 2023, 137 patients were enrolled to arms 1 and 2 and received tovorafenib—77 in arm 1 and 60 in arm 2. Seven (9%) patients in arm 1 and 21 (35%) patients in arm 2 received tovorafenib as a liquid formulation; all other patients received the tablet formulation. As of the datacut, 102 patients remained on treatment (Fig. 1). The most common reasons for treatment discontinuation were progressive disease (PD) and AEs. Patient demographics and baseline characteristics are summarized in Table 1 and were similar between arms. In all 137 patients, the median age was 9 years (range, 1–24); most patients were White (58%); and most patients had astrocytic tumors (93%). The most common tumor locations were optic pathway (50%) and deep midline structures (15%). Seventy-four percent of patients had a tumor harboring a *KIAA1549*::*BRAF* fusion; 10% had a chromosomal rearrangement involving *BRAF* (as detected by fluorescence in situ hybridization or in situ hybridization and presumed to represent a *KIAA1549*::*BRAF* or other *BRAF* fusion); and 16% had a BRAF V600E mutation. Patients had received a median of three lines of prior therapy (range, 1–10), with 61% having received a prior MEK and/or BRAF inhibitor.

**Table 1:** Patient and baseline characteristics (*n* = 137)

Characteristic	Arm 1 *n* = 77	Arm 2 *n* = 60	Total *n* = 137
Age, years			
Median (range)	8 (2–21)	10 (1–24)	9 (1–24)
Gender, *n* (%)			
Male	40 (52)	33 (55)	73 (53)
Female	37 (48)	27 (45)	64 (47)
Race, *n* (%)^a^			
White	41 (53)	38 (63)	79 (58)
Asian	5 (6)	5 (8)	10 (7)
Black	2 (3)	1 (2)	3 (2)
Multiple	3 (4)	0	3 (2)
Other	6 (8)	2 (3)	8 (6)
Not specified	20 (26)	14 (23)	34 (25)
Missing	0	0	0
Ethnicity, *n* (%)			
Hispanic or Latino	3 (4)	1 (2)	4 (3)
Not Hispanic or Latino	51 (66)	47 (78)	98 (72)
Not stated	21 (27)	12 (20)	33 (24)
Missing	2 (3)	0	2 (1)
Primary tumor location, *n* (%)			
Optic pathway	39 (51)	29 (48)	68 (50)
Deep midline structures	9 (12)	11 (18)	20 (15)
Cerebral hemisphere	6 (8)	5 (8)	11 (8)
Brain stem	6 (8)	2 (3)	8 (6)
Cerebellum	5 (6)	2 (3)	7 (5)
Other^b^	12 (16)	11 (18)	23 (17)
Histology, *n* (%)			
Astrocytic	72 (94)	55 (92)	127 (93)
Oligodendroglial	0	1 (2)	1 (1)
Mixed glial-neuronal	4 (5)	4 (7)	8 (6)
Other	1 (1)	0	1 (1)
BRAF alteration status, *n* (%)			
*BRAF* fusion	64 (83)	51 (85)	115 (84)
*KIAA1549*::*BRAF* fusion	56 (73)	45 (75)	101 (74)
Other	8^c^ (10)	6 (10)	14 (10)
BRAF V600E mutation	13 (17)	9 (15)	22 (16)
Baseline Lansky performance score, *n*/*n* (%)^d^			
50–70	3/71 (4)	9/51 (18)	12/122 (10)
80–100	68/71 (96)	42/51 (82)	110/122 (90)
Baseline Karnofsky performance score, *n*/*n* (%)^d^			
50–70	2/6 (33)	0	2/15 (13)
80–100	4/6 (67)	9/9 (100)	13/15 (87)
Prior lines of systemic therapy			
Median (range)	3 (1–9)	3 (1–10)	3 (1–10)
Number of prior lines, *n* (%)			
1	17 (22)	14 (23)	31 (23)
2	21 (27)	13 (22)	34 (25)
≥3	39 (51)	33 (55)	72 (53)
Prior MAPK pathway targeted therapy, *n* (%)			
Prior MEK inhibitor	43 (56)	34 (57)	77 (56)
Prior BRAF inhibitor	8 (10)	7 (12)	15 (11)
Prior MEK and BRAF inhibitors	5 (7)	4 (7)	9 (7)
Prior MEK and/or BRAF inhibitor^e^	46 (60)	37 (62)	83 (61)
Any prior surgery for treatment of primary disease, *n* (%)			
Pre-operative staging			
Localized disease	60 (78)	42 (70)	102 (74)
Disseminated/metastatic disease	9 (12)	11 (18)	20 (15)
Leptomeningeal spread	8 (10)	7 (12)	15 (11)
Post-operative staging			
Gross total resection	1 (1)	0	1 (1)
Subtotal resection	36 (47)	27 (45)	63 (46)
Biopsy only, resection not attempted	40 (52)	33 (55)	73 (53)
Prior radiotherapy for primary disease, *n* (%)	6 (8)	4 (7)	10 (7)

^a^There were no Native Hawaiian or other Pacific Islander, American Indian or Alaska Native participants.

^b^Includes tumors that were extending into multiple regions of the brain, leptomeningeal disease and/or spinal disease.

^c^Includes six patients with *BRAF* duplication and two with *BRAF* rearrangement per fluorescence in situ hybridization or in situ hybridization.

^d^Denominators for Lansky performance score and Karnofsky performance score summaries are the number of patients whose ages are <16 years and ≥16 years, respectively. Baseline is defined as the last available assessments before start of tovorafenib on cycle 1, day 1.

^e^Five patients in arm 1 and four patients in arm 2 had previously received both a MEK inhibitor and also a BRAF inhibitor. These patients are recorded in both the ‘Prior MEK inhibitor’ and ‘Prior BRAF inhibitor’ groups.

The IRC deemed 69 patients in arm 1 who received ≥1 dose of tovorafenib to have measurable disease at baseline per RANO-HGG criteria. Seventy-six patients in arm 1 were deemed evaluable for efficacy by the IRC per RAPNO and RANO-LGG criteria, which use T2-weighted imaging for response assessment. Safety was analyzed in all patients in arms 1 and 2 who received ≥1 dose of tovorafenib (*n* = 137).

Patients in arm 1 had received a median of 18 treatment cycles (range, 1–26), with median treatment compliance of 100% (range, 93–100) (treatment compliance (%) was the total actual dose (mg) / total expected dose (mg) × 100%). The median duration of treatment (DOT) was 15.8 months (range, 0.7–23.7), with 66% (*n* = 51) continuing treatment. Two patients had completed 26 treatment cycles and opted to enter a drug holiday period. Neither patient had subsequent imaging after initiation of the drug holiday period. All other patients (except the ones who discontinued drug early) were still on treatment as of the 5 June 2023 datacut. Patients in arm 2 had received a median of 11 treatment cycles (range, 2–15), with median treatment compliance of 100% (range, 83–100). The median DOT was 9.7 months (range, 1.2–13.3), with 85% (*n* = 51) continuing treatment. There was no decline from baseline in Karnofsky or Lansky performance status scores during treatment with tovorafenib.

### RANO-HGG criteria (primary and secondary endpoints)

The ORR per RANO-HGG criteria (primary endpoint) in patients with response-evaluable disease was 67% (95% confidence interval (CI): 54–78), including 12 (17%) patients with a complete response (CR) and 34 (49%) patients with a partial response (PR). Eighteen (26%) patients had a best overall response (BOR) of stable disease (SD), giving a CBR of 93% (Table 2). The ORR was 69% (95% CI: 56–81) in patients with tumors harboring *BRAF* fusions and 50% (95% CI: 19–81) in patients with tumors harboring BRAF V600E mutations. The ORR was 71% (95% CI: 54–84) in the 59% of evaluable patients who had received prior MAPK inhibitor (MAPKi) therapy and 61% (95% CI: 41–78) in those who had not.

**Table 2:** Response by radiological criteria for patients in arm 1

Response	RANO-HGG		RAPNO^c^		RANO-LGG	
	*n*		*n*		*n*	
ORR,^a^ *n* (%)	69	46 (67)	76	39 (51)	76	40 (53)
* BRAF* fusion	59	41 (69)	64	33 (52)	64	33 (52)
BRAF mutation	10	5 (50)	12	6 (50)	12	7 (58)
Prior MAPKi	41	29 (71)	45	22 (49)	45	23 (51)
MAPKi-naive	28	17 (61)	31	17 (55)	31	17 (55)
CBR,^a^ *n* (%) (SD of any length of time)	69	64 (93)	76	62 (82)	76	63 (83)
* BRAF* fusion	59	55 (93)	64	53 (83)	64	53 (83)
BRAF mutation	10	9 (90)	12	9 (75)	12	10 (83)
Prior MAPKi	41	37 (90)	45	38 (84)	45	38 (84)
MAPKi-naive	28	27 (96)	31	24 (77)	31	25 (81)
CBR,^a^ *n* (%) (SD ≥ 6 months)	69	57 (83)	76	45 (59)	76	52 (68)
* BRAF* fusion	59	52 (88)	64	39 (61)	64	43 (67)
BRAF mutation	10	5 (50)	12	6 (50)	12	9 (75)
Prior MAPKi	41	34 (83)	45	26 (58)	45	31 (69)
MAPKi-naive	28	23 (82)	31	19 (61)	31	21 (68)
CBR,^a^ (SD ≥ 12 months)	69	54 (78)	76	43 (57)	76	46 (61)
*BRAF* fusion	59	49 (83)	64	37 (58)	64	39 (61)
BRAF mutation	10	5 (50)	12	6 (50)	12	7 (58)
Prior MAPKi	41	33 (80)	45	25 (56)	45	26 (58)
MAPKi-naive	28	21 (75)	31	18 (58)	31	20 (65)
BOR,^a^ *n* (%)	69		76		76	
CR		12 (17)		0		0
PR		34 (49)		28 (37)		20 (26)
MR		n/a		11 (14)		20 (26)
SD		18 (26)		23 (30)		23 (30)
SD <12 months		10 (14)		19 (25)		17 (22)
SD ≥12 months		8 (12)		4 (5)		6 (8)
PD		4 (6)		13 (17)		11 (14)
NE		1 (1)		1 (1)		2 (3)
Median DOR, months (95% CI)^b^	46	16.6 (11.6–NR)	39	13.8 (11.3–NR)	40	14.4 (11.0–NR)
* BRAF* fusion	41	16.8 (11.6–NR)	33	13.8 (11.3–NR)	33	16.3 (11.0–NR)
BRAF mutation	5	15.1 (8.3–NR)	6	NR (8.4–NR)	7	12.0 (8.4–NR)
Prior MAPKi	29	15.1 (9.0–16.8)	22	13.8 (11.3–NR)	23	12.0 (8.5–NR)
MAPKi-naive	17	NR (11.6–NR)	17	NR (8.4–NR)	17	16.3 (8.4–NR)
Median TTR, months (range)	46	3.0 (2.6–16.6)	39	5.3 (1.6–11.2)	40	5.5 (1.6–11.3)
* BRAF* fusion	41	3.0 (2.6–16.6)	33	5.5 (2.3–11.2)	33	5.5 (2.3–11.0)
BRAF mutation	5	2.7 (2.6–16.4)	6	2.8 (1.6–3.0)	7	2.9 (1.6–11.3)
Prior MAPKi	29	2.8 (2.6–16.6)	22	5.4 (1.6–11.2)	23	5.5 (1.6–11.3)
MAPKi-naive	17	5.3 (2.6–11.1)	17	5.3 (2.3–11.0)	17	5.3 (2.3–11.0)

^a^ORR, CBR and BOR for RAPNO and RANO-LGG criteria included MRs (that is, ORR = CR + PR + MR; CBR = CR + PR + MR + SD (calculated based on SD of any length of time, SD ≥ 6 months and SD ≥ 12 months)). For CR, PR and MR, confirmation of response by a subsequent scan approximately 3 months after the initial response was required.

^b^The 95% CIs were calculated using the Kaplan–Meier method.

^c^A subgroup analysis of IRC-assessed ORR based on RAPNO criteria (sex, age group, race, geographical location (US/ex-US)) was conducted. Although the small number of patients in some subgroups limits interpretation of these data, responses were observed among all subgroups, with no trends in ORR apparent (Extended Data Fig. 3). Patients with *BRAF* duplication or rearrangement are considered in the *BRAF* fusion group. n/a, not applicable.

**Fig. 2:**
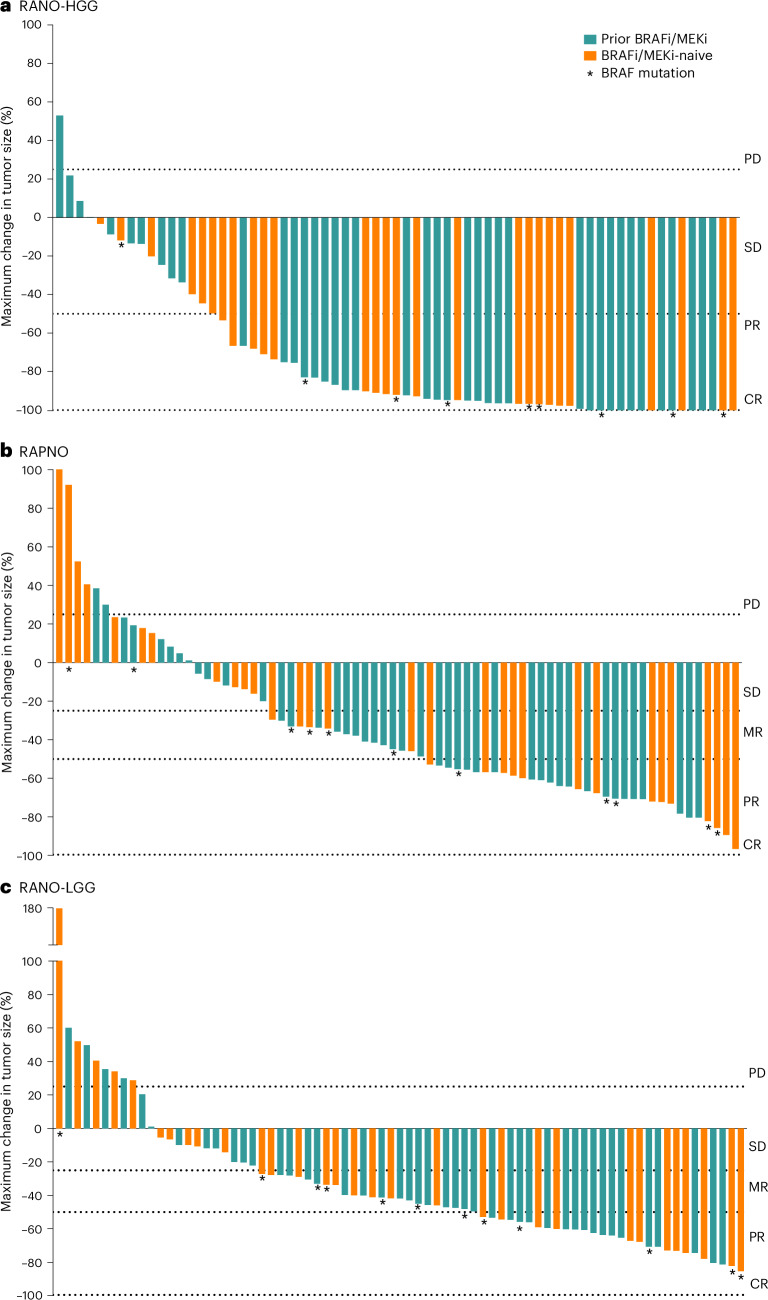
Maximal change in tumor size for evaluable patients. Per RANO-HGG (**a**), RAPNO (**b**) and RANO-LGG (**c**) criteria. Two patients are not shown in the waterfall plots. One patient died due to PD (not tovorafenib related) before the first tumor assessment, and one patient with missing T1 gadolinium-enhanced imaging at baseline was deemed not evaluable. The dashed lines indicate the range of growth/shrinkage of target lesions to be considered as one of the requirements for PD, SD, MR, PR or CR. BRAFi, BRAF inhibitor.

**Fig. 3:**
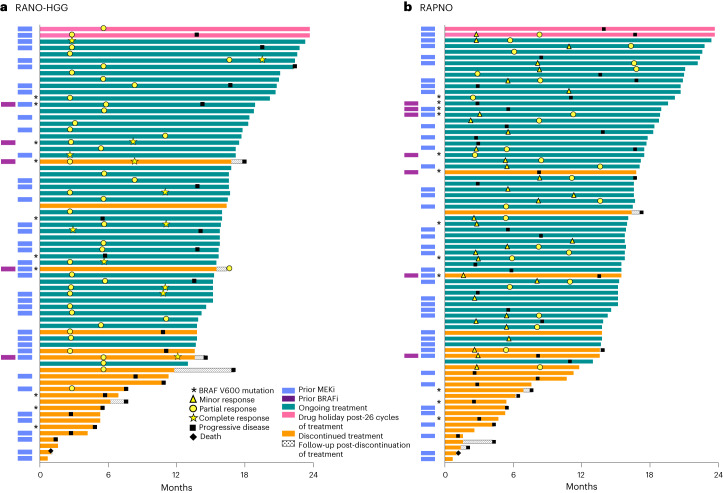
Swimlane plot of TTR and DOT. Per RANO-HGG (**a**) and RAPNO (**b**) criteria. In patients with response, symbols indicate the start of response (MR, PR or CR). If initial responses improved with continued treatment (from MR to confirmed PR or from PR to confirmed CR), both the timepoint of the initial response and the timepoint that the response initially improved are marked accordingly. BRAFi, BRAF inhibitor.

**Extended Data Fig. 1:**
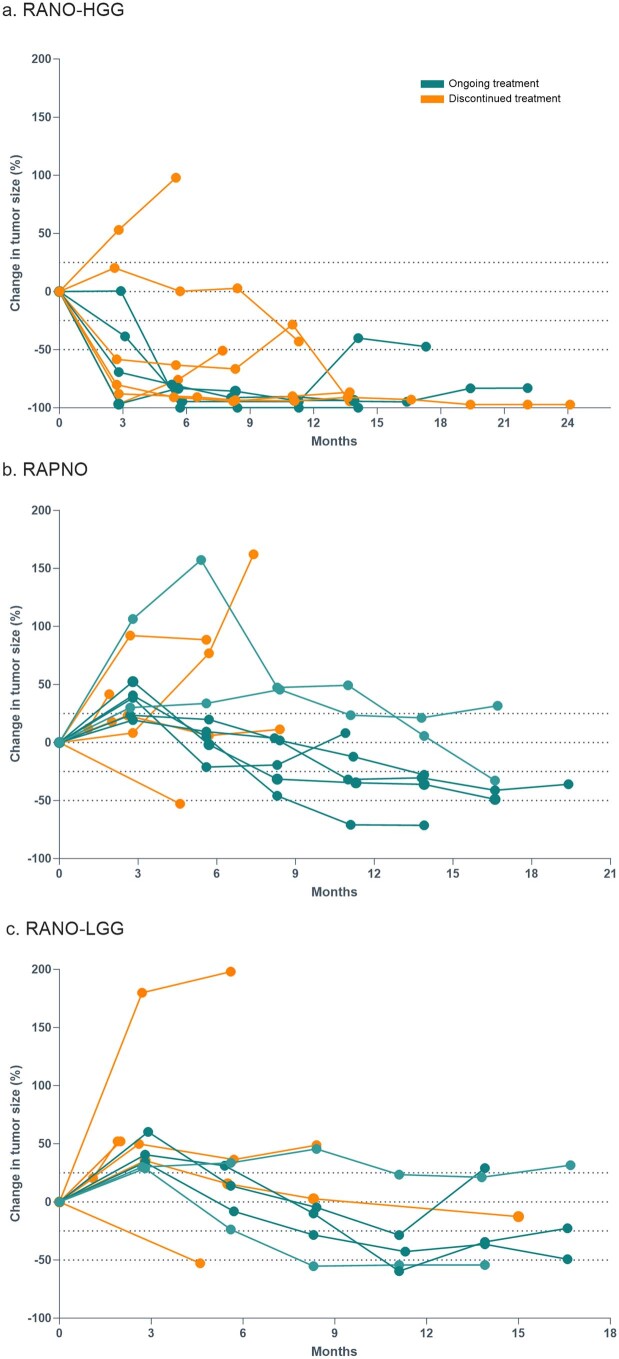
Tumor kinetics. Fig. 1a shows tumor kinetics in patients who progressed while on therapy per RANO-HGG but continued to receive tovorafenib and who had at least one assessment from a scheduled visit post-PD. Fig. 1b,c show tumor kinetics in patients with best response of progressive disease according to RAPNO and RANO-LGG criteria, respectively. In some patients, who continued therapy due to the absence of a progressive disease assessment per RANO-HGG criteria, an apparent initial increase in tumor size per RAPNO and RANO-LGG criteria was subsequently followed by a sustained decrease in size, suggesting that the initial apparent increase may not represent true progression. HGG, high-grade glioma; LGG, low-grade glioma; PD, progressive disease; RANO, Response Assessment in Neuro-Oncology; RAPNO, Response Assessment in Pediatric Neuro-Oncology.

Figure 2a shows the best tumor response from baseline, and Fig. 3a shows DOT and timing of response. In 13% (*n* = 9) of patients, an initial PR was followed by a CR with continued treatment. The median time to initial response (PR or CR) was 3.0 months (range, 2.6–16.6), and the median DOR was 16.6 months (95% CI: 11.6–not reached (NR)). The median PFS was 19.4 months (95% CI: 16.9–NR). Of the patients who progressed while on therapy per RANO-HGG criteria and continued to receive tovorafenib, 10 had at least one assessment from a scheduled visit after PD. Tumor kinetics for these patients are shown in Extended Data Fig. 1a, with six having tumor shrinkage close to CR.

### RAPNO criteria (secondary endpoints)

The ORR per RAPNO criteria was 51% (95% CI: 40–63), including 28 (37%; 95% CI: 26–49) patients with a PR (≥50% reduction from baseline by sum of the product of the perpendicular diameters (SPPD)) and 11 (14%; 95% CI: 8–24) patients with a minor response (MR; 25–49% reduction from baseline by SPPD). Twenty-three (30%) patients had a BOR of SD, resulting in a CBR of 82%. The ORR was similar in patients with tumors harboring *BRAF* fusions (52%; 95% CI: 39–64) and BRAF V600E mutations (50%; 95% CI: 21–79) and in patients who had received prior MAPKi therapy (49%; 95% CI: 34–64) and those who had not (55%; 95% CI: 36–73) (Table 2).

Figure 2b shows the best tumor response from baseline, with most tumors showing some degree of shrinkage. Figure 3b shows DOT and timing of response. In 29% (*n* = 22) of patients, an initial MR was followed by a confirmed PR with continued treatment. The median time to initial response (MR or PR) was 5.3 months (range, 1.6–11.2) overall; 2.8 months (range, 1.6–3.0) in patients with tumors harboring BRAF V600E mutations; and 5.5 months (range, 2.3–11.2) in patients with tumors harboring *BRAF* fusions. The median DOR was 13.8 months (95% CI: 11.3–NR), and the median PFS of the 76 patients was 13.8 months (95% CI: 8.3–16.9).

Thirteen (17.1%) patients initially had a best overall response of PD according to RAPNO criteria per IRC evaluation but continued tovorafenib treatment due to the absence of PD by RANO-HGG criteria per investigator, consistent with the trial design and primary endpoint assessment. All but two patients had no symptoms or signs of clinical progression. Plots of tumor size (SPPD) over time in several of these patients showed subsequent tumor shrinkage after the initial assessment of PD (Extended Data Fig. 1b).

### RANO-LGG criteria (post hoc exploratory endpoints)

The ORR per RANO-LGG criteria was 53% (95% CI: 41–64), including 20 (26%; 95% CI: 17–38) patients with a PR and 20 (26%; 95% CI: 17–38) with an MR. Twenty-three (30%) patients had a BOR of SD, giving a CBR of 83%. ORR was similar in patients with tumors harboring *BRAF* fusions and BRAF V600E mutations and in patients previously treated with a MAPKi or not (Table 2).

**Extended Data Fig. 2:**
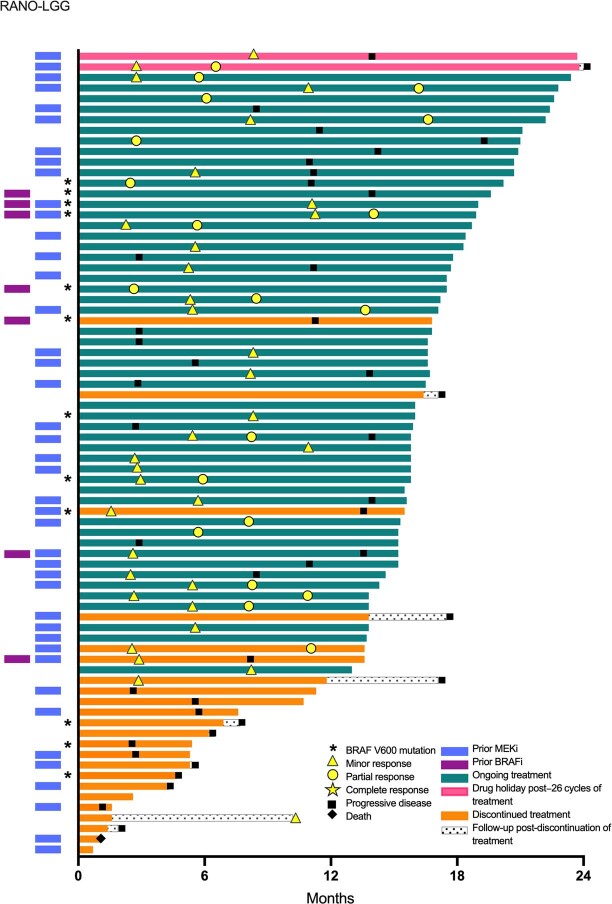
Swimlane plot of time to response and duration of therapy per RANO-LGG criteria. In patients with response, symbols indicate the start of response (MR or PR). If initial response improved with continued treatment (from MR to confirmed PR), both the timepoint of the initial response and the timepoint that response initially improved are marked accordingly. BRAFi, BRAF inhibitor; LGG, low-grade glioma; MEKi, MEK inhibitor; MR, minor response; PR, partial response; RANO, Response Assessment in Neuro-Oncology.

Figure 2c shows the best tumor response from baseline, again with most tumors showing some decrease in size. Extended Data Fig. 2 shows the DOT and timing of response. In 18% (*n* = 14) of patients, an initial MR was followed by a confirmed PR with continued treatment. The median time to initial response (MR or PR) was 5.5 months (range, 1.6–11.3) overall; 2.9 months (range, 1.6–11.3) in patients with tumors harboring BRAF V600E mutations; and 5.5 months (range, 2.3–11.0) in patients with tumors harboring *BRAF* fusions. The median DOR was 14.4 months (95% CI: 11.0–NR), and the median PFS was 13.9 months (95% CI: 11.1–19.1).

Similar to the RAPNO analysis, there was a subset of patients who initially had PD according to RANO-LGG criteria per IRC evaluation but who continued on treatment due to the absence of PD according to RANO-HGG criteria. Plots of tumor size (SPPD) over time in several of these patients showed a pattern of subsequent tumor shrinkage after the initial PD assessment (Extended Data Fig. 1c).

### Response to tovorafenib in patients with PD while on a MAPKi as their most recent prior therapy (post hoc analysis)

A post hoc analysis was completed using all three radiological criteria to assess responses to tovorafenib in patients who received a MAPKi as their most recent line of therapy and discontinued due to progression. For RANO-HGG criteria, 33% (n = 15) of patients who had progressed on a MAPKi as their most recent prior therapy had a CR or a PR to tovorafenib; similar trends were observed for RAPNO and RANO-LGG crieria, which include MRs: 33% (*n* = 13) had a PR or an MR (31% (*n* = 12) with PR), and 30% (*n* = 12) had a PR or an MR (20% (*n* = 8) with PR), respectively (Extended Data Table 2).

### Safety

Among 137 patients (arms 1 and 2), 118 (86%) had been treated for at least 6 months and 67 (49%) for at least 1 year. All patients experienced at least one treatment-emergent adverse event (TEAE). The most common TEAEs at any grade occurring in ≥20% of patients were hair color changes (76%), anemia (59%) and elevated creatine phosphokinase (CPK) (58%) (Table 3). Grade ≥3 TEAEs occurred in 63% of patients, the most common being elevated CPK (12%), anemia (11%) and maculopapular rash (8%). The most common treatment-related adverse events (TRAEs) of any grade generally followed a similar trend to the most common TEAEs. (A full listing of TRAEs of any grade and grade ≥3 occurring in ≥1 patient can be found in Supplementary Table 1). Grade ≥3 TRAEs occurred in 42% of patients and followed a similar trend as the most common grade ≥3 TEAEs, although pyrexia was less often assessed as a related event. Two patients had grade 5/fatal TEAEs, neither of which was assessed as treatment related (one patient in arm 1 with disease progression and one patient in arm 2 with a disseminated leptomeningeal mixed-glial-neuronal tumor who experienced tumor hemorrhage 21 d after their final dose of tovorafenib (discontinued due to disease progression)).

**Table 3:** TEAEs and TRAEs (safety analysis set, *n* = 137)

Preferred term, *n* (%)	TEAEs		TRAEs	
	Any grade	Grade ≥3	Any grade	Grade ≥3
Patients with any AE	137 (100)	86 (63)	134 (98)	58 (42)
Hair color changes	104 (76)	0	104 (76)	0
Anemia	81 (59)	15 (11)	67 (49)	14 (10)
Elevated CPK	80 (58)	16 (12)	77 (56)	16 (12)
Fatigue	76 (55)	6 (4)	60 (44)	6 (4)
Vomiting	68 (50)	6 (4)	28 (20)	3 (2)
Hypophosphatemia	64 (47)	0	48 (35)	0
Headache	61 (45)	2 (1)	29 (21)	0
Maculopapular rash	60 (44)	11 (8)	56 (41)	11 (8)
Pyrexia	53 (39)	5 (4)	17 (12)	1 (1)
Dry skin	49 (36)	0	45 (33)	0
Elevated LDH	48 (35)	0	42 (31)	0
Increased AST	47 (34)	4 (3)	41 (30)	4 (3)
Constipation	45 (33)	0	31 (23)	0
Nausea	45 (33)	0	25 (18)	0
Upper RTI	43 (31)	2 (1)	2 (1)	0
Dermatitis acneiform	42 (31)	1 (1)	41 (30)	1 (1)
Epistaxis	42 (31)	1 (1)	27 (20)	0
Decreased appetite	39 (28)	5 (4)	28 (20)	4 (3)
Paronychia	36 (26)	2 (1)	32 (23)	2 (1)
Pruritus	35 (26)	1 (1)	32 (23)	1 (1)
COVID-19	34 (25)	0	0	0
Weight decreased	30 (22)	2 (1)	21 (15)	0
Cough	30 (22)	1 (1)	2 (1)	0
Increased ALT	29 (21)	7 (5)	24 (18)	7 (5)
Hypokalemia	29 (21)	4 (3)	15 (11)	3 (2)
Diarrhea	29 (21)	2 (1)	14 (10)	1 (1)
Eczema	25 (18)	2 (1)	22 (16)	2 (1)
Hypocalcemia	22 (16)	2 (1)	13 (9)	1 (1)
Erythema	20 (15)	2 (1)	18 (13)	1 (1)
Photosensitivity reaction	19 (14)	1 (1)	19 (14)	1 (1)
Decreased growth velocity	18 (13)	7 (5)	17 (12)	7 (5)
Increased bilirubin	18 (13)	1 (1)	16 (12)	1 (1)
Hyponatremia	17 (12)	3 (2)	4 (3)	1 (1)
Erythematous rash	14 (10)	1 (1)	14 (10)	1 (1)
Decreased WBC count	13 (9)	2 (1)	11 (8)	1 (1)
Tumor hemorrhage	12 (9)	5 (4)	10 (7)	3 (2)
Decreased neutrophil count	12 (9)	4 (3)	9 (7)	3 (2)
Seizure	11 (8)	6 (4)	1 (1)	1 (1)
Weight increased	10 (7)	2 (1)	6 (4)	1 (1)
Lethargy	9 (7)	1 (1)	6 (4)	1 (1)
Pericardial effusion	9 (7)	1 (1)	5 (4)	1 (1)
Decreased phosphorus	7 (5)	1 (1)	5 (4)	1 (1)
Folliculitis	5 (4)	1 (1)	4 (3)	1 (1)
Prolonged APTT	3 (2)	1 (1)	2 (1)	1 (1)
Increased GGT	3 (2)	1 (1)	1 (1)	1 (1)
Systemic infection	2 (1)	2 (1)	1 (1)	1 (1)
Follicular rash	2 (1)	1 (1)	2 (1)	1 (1)
Optic nerve disorder	2 (1)	1 (1)	1 (1)	1 (1)
CSF circulation disorder	1 (1)	1 (1)	1 (1)	1 (1)
Cholecystitis	1 (1)	1 (1)	1 (1)	1 (1)
Eye infection viral	1 (1)	1 (1)	1 (1)	1 (1)
Pruritic rash	1 (1)	1 (1)	1 (1)	1 (1)

TEAEs, TRAEs and laboratory abnormalities in ≥20% of patients and all TRAEs grade ≥3 occurring in ≥1 patient are reported. Patients are counted only once per event and are shown in the worst CTCAE grade that was reported for each event they experienced. MedDRA version 23.1; CTCAE version 5.0. APTT, activated partial thromboplastin time; AST, aspartate aminotransferase; COVID-19, coronavirus disease 2019; CSF, cerebrospinal fluid; GGT, gamma-glutamyl transferase; LDH, lactate dehydrogenase; RTI, respiratory tract infection; WBC, white blood cell.

A decrease in growth velocity was observed in patients treated with tovorafenib. At cycle 13 (*n* = 74), the median decrease in height *z*-score was 0.7, or less than 1 s.d. Eighty percent of these patients remained within 1 s.d. from baseline height *z*-score. The remaining patients had a decrease from baseline height *z*-score of between 1 s.d. and 2 s.d. At the time of this report, on-treatment bone age results were available for 11 patients. Nine of these 11 patients were reported as within normal limits for age; one patient was read as delayed; and one patient with a bone age advanced by 3.5 years before the start of treatment had an on-treatment bone age that was reported as advanced by 2.5 years. So far, there has been no evidence of bone age advancement or premature closure of growth plates. In patients with available height data off-treatment, growth velocity was recovering.

Serious TEAEs were reported for 45% of patients, the most common being pyrexia (most cases were confounded by intercurrent infectious disease), seizure and vomiting (each 5%). Serious TRAEs occurred in 15% of patients, the most common being tumor hemorrhage (3%), decreased appetite, hyponatremia and vomiting (each 2%). Two of four patients with serious tumor hemorrhage TRAEs had a history of intratumoral hemorrhage before initiating tovorafenib, and, in all four, the serious events of tumor hemorrhage resolved.

Nine (7%) patients had TRAEs leading to tovorafenib discontinuation, the most common being tumor hemorrhage (three patients) and decrease in growth velocity (two patients). TRAEs leading to dose reduction were seen in 33 (24%) patients, the most common being maculopapular rash (4%) and decreased appetite (3%). TRAEs leading to dose interruption occurred in 50 (37%) patients, the most common being maculopapular rash (9%), vomiting, fatigue, increased alanine aminotransaminase (ALT) and elevated CPK (each 4%). The median duration of dose interruption due to any TEAE was 14 d, or two doses.

## Discussion

In this international, multicenter, single-arm phase 2 trial, tovorafenib monotherapy resulted in clinically meaningful, rapid and durable tumor responses in children and young adults with *BRAF*-altered relapsed/refractory pLGG. The clinical activity of tovorafenib is particularly noteworthy given that patients had received a median of three prior lines of systemic therapy, and more than half had previously received RAF and/or MEK inhibitors. Tumor responses were demonstrated across the three response assessment criteria (RANO-HGG, RAPNO and RANO-LGG), *BRAF* alteration type (mutation versus fusion) and prior MAPKi use, including patients who progressed on a MAPKi as their most recent prior therapy. There was a consistent pattern of improvement of response over time on both T1-weighted and T2-weighted magnetic resonance imaging (MRI) sequences.

This trial was designed with IRC-assessed ORR as the primary endpoint, evaluated according to RANO-HGG criteria. These criteria assess tumor response based primarily on T1-weighted, contrast-enhanced imaging. At the time the original protocol was initiated, the largest trial in pLGG with published efficacy results in the relapsed/refractory population primarily used T1-weighted, contrast enhancement–based, centrally reviewed assessment criteria to evaluate the treatment effect of vinblastine^33^. According to RANO-HGG criteria, the FIREFLY-1 trial met its primary endpoint by rejecting the null hypothesis ORR of 21% observed for single-agent vinblastine in this setting^34^.

For patients with pLGG, decrease in contrast-enhancing tumor may not represent all aspects of anti-tumor activity. The RAPNO working group recommendations highlighted the lack of standard response criteria in clinical trials of pLGG as well as the biologic difference between pediatric and adult gliomas^31^. RAPNO criteria focus on T2-weighted fluid-attenuated inversion recovery (FLAIR) imaging for assessing response in pLGG rather than changes in contrast-enhancing disease. This is beneficial as pLGGs have clinical and biological features distinct from adult LGGs, and assessment of the contrast-enhancing portion of the tumor may not be the best indicator of response in this population^31,35^. RAPNO criteria take into account changes in tumor-associated cysts; include an MR category (tumor reduction of 25% to <50%); and recommend including visual outcomes in response assessment (optic pathway and hypothalamic pLGGs), given their clinical importance in pLGG. Therefore, assessment of tumor response by IRC according to RAPNO and RANO-LGG criteria (both of which assess tumor response based primarily on non-enhancing disease by T2/FLAIR) were included in this trial as secondary and post hoc exploratory endpoints, respectively (Extended Data Table 1 summarizes the characteristics of the three different response assessment criteria). Sustained decreases in tumor size were observed in most patients and represent clinically meaningful changes by T2/FLAIR-based assessment criteria. As in the current trial, a similar pattern of response with a greater magnitude of decrease for contrast enhancement assessments compared to T2/FLAIR assessments in patients with relapsed/refractory pLGG was previously reported in a phase 1 trial of the MEK inhibitor selumetinib^36^. ORRs and CBRs for RAPNO and RANO-LGG criteria were very similar in arm 1. Although in limited numbers of patients, TTR in patients with tumors harboring BRAF V600E mutations appeared to be shorter by both assessment criteria (2.8 months and 2.9 months, respectively) compared to patients with tumors harboring *BRAF* fusions (5.5 months and 5.5 months).

Although responses to tovorafenib generally occurred early, the kinetic analysis of tumor size over time per RAPNO and RANO-LGG criteria revealed that some patients who remained on treatment after an initial assessment of radiographic PD subsequently had marked tumor shrinkage, suggestive of a delayed response to treatment. This type of response pattern (that is, tumor flare or pseudoprogression) was previously observed in patients treated with immune checkpoint inhibitors, where some patients experience an initial increase in tumor size, followed later by an objective response^37,38^. It has been suggested that such effects may be related to a transient immune cell infiltration of the tumor, leading to an initial increase in apparent tumor burden^37,39^. Immune cells, especially microglia, may comprise 40% of all cells in pilocytic astrocytomas and can account for differences in RNA expression profiles between tumor locations and subtypes^40^. The possibility of a late response to tovorafenib after an apparent initial increase in tumor size highlights the challenge of efficacy evaluation in this patient population using established response assessment criteria, and it raises the possibility that some patients may benefit from tovorafenib treatment until radiographic progression is confirmed by a second MRI scan. Moving forward, follow-up imaging 8–12 weeks after an initial PD assessment will be suggested for patients receiving tovorafenib with early radiographic progression, in the absence of clinical evidence of progression.

By using both T1-weighted contrast-enhanced (RANO-HGG) and T2/FLAIR-based (RAPNO and RANO-LGG) approaches, the current trial assessed the impact of tovorafenib on different aspects of pLGG tumor biology and response. Despite the unique challenges of ascertaining an optimal, single set of response assessment criteria for this heterogeneous disease, confirmed responses to tovorafenib over time were observed across three different neuro-oncology response assessment criteria. To our knowledge, this is the first trial to report outcomes for these three criteria for a large, uniformly treated patient group.

The main limitation of the current trial is that it is a single-arm clinical trial. However, this design was considered both sufficient and necessary due to the lack of a SOC for most patients with relapsed/refractory pLGG. In addition, there was a lack of diversity in relation to ethnicity/race of the patient population. The efficacy results of tovorafenib in FIREFLY-1 are similar to those in earlier non-registrational studies investigating MAPK inhibitors in this setting. The phase 2 trial of the MEKi selumetinib^20^ demonstrated a sustained PR in nine of 25 patients (36%, excluding MRs) with *BRAF*-altered pLGG as assessed by T2/FLAIR imaging, with a median time to progression of 22.9 months and a median follow-up of 26.9 months. Other reports of MEKi monotherapy in this patient population demonstrated anti-tumor activity to varying degrees^21–23,41^. The over 50% ORR reported for tovorafenib using RAPNO and RANO-LGG criteria in the current trial is particularly noteworthy given that over half of patients had previously received MAPKi therapy.

The safety and tolerability profile of tovorafenib monotherapy in children and young adults with pLGG was encouraging, with TRAEs being predominantly grade 1 or 2 and only nine (7%) of 137 patients discontinuing due to a TRAE. The most common TRAEs of any grade, excluding laboratory abnormalities, were hair color changes, fatigue, maculopapular rash, dry skin, acneiform dermatitis, pruritus and paronychia. Although grade 1 and 2 laboratory abnormalities were commonly reported as AEs, most were not associated with any clinical symptoms or need for clinical intervention or change in therapy. The most common grade ≥3 TRAEs included elevated CPK, anemia and maculopapular rash. Other common grade ≥3 TEAEs (not assessed as related to tovorafenib), including seizures, were consistent with effects of the underlying disease.

Overall, the AEs were consistent with the tovorafenib phase 1 trial in adults dosed once weekly and similar to other targeted agents used as pLGG therapy, including MAPK inhibitors, with some notable exceptions^26^. Hair color changes, which have been reported in clinical studies of pediatric patients with pLGG treated with other MAPK inhibitors, occurred more often with tovorafenib^42^. However, pyrexia, diarrhea and weight gain—common adverse reactions for BRAFi/MEKi combination therapy—were less often reported as tovorafenib related and did not significantly disrupt treatment. No signs of ocular toxicity, adverse impact on cardiac function or abnormal weight gain were observed in children treated with tovorafenib, unlike those observed with MEK inhibitors^43,44^. Although skin rashes were common in children treated with tovorafenib, no patients experienced life-threatening skin reactions, and development of squamous cell carcinomas and keratoacanthomas were not observed.

There were no on-trial treatment-related deaths, and TRAEs requiring discontinuation were infrequent. Intratumoral hemorrhage was reported in 15 patients and led to discontinuation of therapy in three patients. However, half (eight of 15) of the patients with intratumoral hemorrhage were asymptomatic, with areas of tumor hemorrhage identified on routine trial MRI only. Three of the seven patients with symptomatic tumor hemorrhage had tumor bleeds assessed by the investigator as consistent with the natural history of their underlying tumor and not related to tovorafenib. Of the four patients in which the symptomatic hemorrhage was considered by the investigator to be possibly related to tovorafenib, two patients had a history of tumor hemorrhage before starting the study; the third patient had a disseminated tumor with leptomeningeal disease; and the fourth patient had bone marrow failure secondary to prior treatment with multiple alkylating agents that was diagnosed before the onset of tumor hemorrhage. Although the incidence of intratumoral hemorrhage in this patient population is not well described, case reports suggest that the tumor hemorrhage risk across pLGG tumor types with heterogenous histology, morphology and prior interventions may be underappreciated^45–48^.

Decreases in growth velocity were observed in children treated with tovorafenib. Among patients with complete endocrinology assessments reported, radiographic studies of the wrist supported conservation of growth potential with no advancement of bone age or evidence of premature fusion of growth plates. Furthermore, patients with available data after discontinuation of tovorafenib in the setting of FIREFLY-1 and prior studies show various degrees of recovery of growth velocity, including achievement of catch-up growth. A more detailed analysis of growth during and after treatment with tovorafenib is planned. Notably, children with cancer, and with midline CNS tumors such as pLGG, commonly develop endocrine abnormalities that may impact growth trajectories and the likelihood of achieving genetic potential for height. In one report, nearly half of children with hypothalamic/chiasmatic gliomas developed at least one endocrine disorder, most commonly a growth hormone deficiency^49^. Normative data for growth in this patient population are lacking, and future studies of targeted therapies in pLGG should include baseline and longitudinal assessments of endocrine function and monitoring of growth velocity during therapy. Long-term follow-up of FIREFLY-1 patients after cessation of treatment is ongoing to assess the impact of transient reduction in growth velocity on final adult height.

Collectively, these results show that tovorafenib monotherapy was generally well tolerated and demonstrated encouraging evidence of clinically meaningful, rapid and durable clinical activity in children and young adults with *BRAF*-altered pLGG. Tovorafenib may, consequently, offer an important treatment option for *BRAF*-altered, relapsed/refractory pLGG, as the observed safety profile compares favorably to currently available therapies for pLGG with a positive benefit–risk ratio. Notably, the availability of a liquid formulation, a weekly dosing regimen and lack of food effect allow for better adherence to the prescribed treatment regimen. Management of common adverse reactions was achieved in most patients, with only brief dose interruptions. These data provide a strong rationale for the ongoing phase 3 LOGGIC/FIREFLY-2 (NCT05566795) trial of tovorafenib monotherapy versus current SOC chemotherapy in children and young adults with pLGG requiring primary systemic treatment.

## Methods

### Trial design

FIREFLY-1 (PNOC026; NCT04775485) is an ongoing, phase 2, multicenter, open-label study evaluating tovorafenib monotherapy in children, adolescents and young adults with *RAF*-altered pLGGs or advanced solid tumors who have received at least one prior systemic therapy. The trial consists of three treatment arms, with patients enrolled from 32 centers in 11 countries (Supplementary Table 2). Arm 1 enrolled patients with relapsed or refractory pLGG harboring an activating *BRAF* alteration, including BRAF V600 mutations and *KIAA1549*::*BRAF* fusions; arm 2 (pLGG expansion cohort) enrolled patients with relapsed or refractory pLGG harboring an activating *RAF* alteration; and arm 3 is enrolling patients with advanced solid tumors harboring an activating *RAF* fusion. *RAF* alterations were identified through molecular assays as routinely performed as part of SOC diagnostic testing at Clinical Laboratory Improvement Amendments (CLIA) of 1988 certified or other similarly certified laboratories. The classification of *BRAF* fusion included *BRAF* tandem duplication, *BRAF* rearrangement and *BRAF* fusions involving partner genes other than *KIAA1549*.

Sex and/or gender were not considered in the trial design as no sex differences have been seen in previous clinical trials in pLGG, although, in line with rates generally seen in childhood cancer^50^, there is one report of the incidence of pLGG being slightly higher in males than in females^51^. The current trial recruited any patient independent of sex or gender. The sex of the participants was based on either parental report or self-report. The gender of patients was not captured or considered as part of this trial as, at the time the protocol was written and the FIREFLY-1 trial was initiated, there was less of a focus than currently on collecting gender-related information.

Tovorafenib was administered at the recommended phase 2 dose of 420 mg m^−^^2^ (not to exceed 600 mg) by mouth (tablet or liquid formulation), once weekly, on days 1, 8, 15 and 22 of 28-d cycles. Treatment cycles were to be repeated every 28 d until radiographic evidence of disease progression as determined by the treating investigator according to RANO-HGG criteria, unacceptable toxicity, decision to enter a drug holiday period, patient withdrawal of consent or death. Patients who had radiographic evidence of disease progression were allowed to continue tovorafenib treatment if, in the opinion of the investigator and approved by the sponsor, the patient was deriving clinical benefit from continuing trial treatment. Patients were to be treated with tovorafenib for a planned period of 26 cycles (approximately 24 months), after which they could continue on tovorafenib or, at any point, opt to enter a drug holiday period. During this drug holiday period, patients could be re-treated with tovorafenib if there was radiographic evidence of disease progression.

An independent data safety monitoring board (DSMB) was established before initiation of the trial and was in place for the duration of the trial. Medical monitoring of the trial was provided by the sponsor and used a safety review committee that included the principal investigator (or their designee) from each active clinical site.

The protocol, protocol amendments, informed consent form, pediatric assent form, investigator brochure and other relevant documents were approved by an institutional review board/independent ethics committee at each trial site. As applicable according to local regulations, the protocol and all protocol amendments were reviewed and approved by each pertinent competent authority.

This trial was conducted in accordance with the protocol and consensus ethical principles derived from international guidelines, including the Declaration of Helsinki, Council for International Organizations of Medical Sciences (CIOMS) International Ethical Guidelines, applicable International Council for Harmonization Good Clinical Practice (GCP) guidelines and other applicable laws and regulations. All patients and/or their legally authorized representative provided written informed consent and pediatric assent before enrollment in the trial, according to local regulations. No direct compensation was provided to patients or families for participating in the trial.

### Data collection

Clinical data required by the protocol were entered into the electronic case report forms (eCRFs) and used a fully validated secure web-enabled electronic data capture (EDC) system, Medidata Classic Rave 2022.3.2, which is compliant with 21 CRF Part 11 requirements. Automatic validation edit checks in the EDC system and offline listings were programmed to capture data discrepancies in the eCRFs and allowed modification and validation of the entered data. The investigator verified and signed off the eCRFs in the EDC system to confirm that the clinical data captured were complete and accurate. The sponsor can attest that all data and metadata will be archived in perpetuity. The data are in the EDC system and the trial master file (TMF), which are retained in perpetuity. In addition, these data have been filed with the US FDA.

### Eligibility

Full inclusion and exclusion criteria are listed in the trial protocol, located in the Supplementary Information for this publication. In brief, eligible patients in arm 1 were aged 6 months to 25 years, inclusive, with a histopathologically verified pLGG, which had previously been treated with at least one line of prior systemic therapy with evidence of radiographic progression, a documented known activating *BRAF* alteration and measurable disease as defined by RANO-HGG criteria, a Lansky (aged <16 years) or Karnofsky (aged ≥16 years) performance score of ≥50 and adequate organ function. Radiation therapy to the measurable lesion(s) must have been completed at least 6 months before administration of tovorafenib, and patients must have fully recovered from the acute toxic effects of all prior anti-cancer chemotherapy and from any prior surgery. Patients must have had adequate bone marrow and organ function, including a left ventricular ejection fraction (LVEF) of ≥50% as measured by ECG or multiple-gated acquisition (MUGA) scan or fractional shortening (FS) ≥25% as measured by ECG^52^, within 28 d before the first dose of tovorafenib. Tumor tissue (archival) was obtained at enrollment whenever available. Tissue biopsy was required during screening only if an archival tumor tissue sample was not available.

Patients were excluded if their tumor harbored an additional previously known or expected to be activating molecular alteration; if they had symptoms of clinical progression without radiographically recurrent or radiographically progressive disease; if they had a known or suspected diagnosis of neurofibromatosis type 1 by genetic testing or current diagnostic criteria; if they had a history or current evidence of central serous retinopathy, retinal vein occlusion or ophthalmopathy present at baseline that would be considered a risk factor for either; if they had clinically significant active cardiovascular disease; or if they were neurologically unstable despite adequate treatment.

### Trial endpoints

The assessment of response was undertaken using three different radiological response assessment criteria: RANO-HGG criteria, which assess tumor response primarily based on T1-weighted, contrast-enhanced imaging, and RAPNO-LGG and RANO-LGG criteria, both of which assess tumor response based primarily on non-enhancing disease by T2/FLAIR. The characteristics of these different response criteria are summarized in Extended Data Table 1. As per the trial design, patients were initially enrolled based on investigator assessment of eligibility per RANO-HGG criteria. Investigator response assessments per RANO-HGG were also the criteria on which cessation of treatment due to PD were based. Response was subsequently analyzed per all three criteria by blinded independent central review.

The primary endpoint in arm 1 was ORR, defined as the proportion of patients with a confirmed response of CR or PR according to RANO-HGG criteria, as assessed by an IRC. Secondary endpoints for arm 1 included CBR, PFS, DOR and TTR, as assessed by the IRC using RANO-HGG criteria. CBR is defined as the proportion of patients with a confirmed response or SD lasting any length of time, 6 months or more or 12 months or more. ORR, CBR, PFS, DOR and TTR were also assessed by the IRC according to RAPNO criteria. Secondary endpoints for safety included evaluation of AEs, laboratory abnormalities and cardiac function assessments (change from baseline in QTcF, PR interval, QRS interval, heart rate or ECG waveform morphology). Post hoc exploratory endpoints for arm 1 included ORR and CBR according to RANO-LGG criteria by IRC assessment. For RAPNO and RANO-LGG ORR assessments, patients with confirmed MRs were considered responders in accordance with published guidelines. Changes in quality of life and health utilities measures were exploratory objectives.

### Assessments

Disease assessments in arm 1 were conducted by MRI of the brain and spine and were performed at screening up to 28 d before first dose, at the end of cycle 3 and then at the end of every three cycles thereafter. Spinal scans were only required to be repeated after screening in patients with known or clinically suspected intraspinal disease. Patients who had an optic pathway glioma (OPG) or underlying visual function deficit related to the primary malignancy had a visual acuity examination every time they had a radiographic disease assessment.

A central imaging laboratory was used. Imaging Endpoints (IE) is a research and imaging core laboratory providing blinded independent central review of response assessments with dual reader plus adjudication paradigm using neuro-radiologists trained in all three response assessment criteria as readers for the following assessments: RANO-HGG criteria, RAPNO-LGG criteria and RANO-LGG criteria. All activities at IE meet or exceed GCP standards, and IE underwent a GCP audit by the sponsor. A prospectively designed imaging charter was developed for the FIREFLY-1 study before the initiation of the study. This outlined the processes for initial imaging review, data transfers and data review, and queries were followed throughout the study. IE functions as the centralized imaging core laboratory responsible for the collection, quality control, archiving and blinded independent central review (BICR) of imaging for the FIREFLY-1 trial. IE is responsible for management of the image analysis system, reporting methods, implementation of the analysis criteria and reader management, including qualification, training and oversight.

Reader performance was assessed by evaluating reader variability at defined and prespecified milestones during ongoing imaging interpretation. Variability metrics included inter-reader and intra-reader variability to monitor for consistency of reads. If the reader acceptance rate fell outside the caution or alert limits, IE determined the appropriate unbiased action(s).

For this report, safety was assessed in the arm 1 and 2 safety analysis set, which comprised all enrolled patients who received at least one dose of trial treatment. The assessment period for AEs was from the first dose of tovorafenib until 30 d after the last dose. For the current trial, an AE was defined as treatment emergent if it occurred at any time after the first dose of trial drug until 30 d after the last dose of trial drug. A TRAE was any treatment-emergent event that the investigator assessed had at least a reasonable possibility of having a causal relationship with the trial drug based on temporal association with initiation of treatment and assessment of other potential etiologies. An AE was considered serious if it met one of the following criteria: required or prolonged hospitalization, was life-threatening, caused disability or was considered a medically important event by the investigator (regardless of symptoms or Common Terminology Criteria for Adverse Events (CTCAE) grade).

Routine laboratory tests were performed locally and included pregnancy tests for female patients of childbearing potential (at screening and on day 1 of every cycle); assessments of hematology parameters and serum chemistries (at screening, days 1 and 15 of cycle 1, day 1 of cycle 2 and every cycle thereafter); and thyroid function (at screening, on day 1 of cycles 1–3 and every other cycle thereafter). Scheduled cardiac function assessments included independently centrally reviewed 12-lead resting ECGs (performed in triplicate at baseline, on days 1 and 15 of cycle 1, on day 1 of cycles 2 and 4 and on day 1 of every third cycle thereafter) and ECGs or MUGA scans (conducted throughout by the same technique, on day 1 of cycles 2 and 4 and on day 1 of every third cycle thereafter). CPK level was assessed at screening, on day 1 of cycles 2 and 4 and on day 1 of every fourth cycle thereafter.

In patients 2 years of age or older, health-related quality of life was assessed using the PedsQL-Core, PedsQL-Cancer and PROMIS assessments for the patient or their parent/caregiver every third cycle. The PROMIS questionnaire was administered only to English-speaking patients enrolled in the United States, Australia and the United Kingdom.

### Statistical considerations

In terms of the ‘evaluable’ population, patients ‘evaluable for efficacy’ were all patients enrolled in the trial who received at least one dose of trial treatment and met the definition for the prespecified efficacy analyses criteria (RANO-HGG, RAPNO and RANO-LGG)^30–32^; those in the ‘evaluable for DOR’ population were patients evaluable for efficacy who had a best overall confirmed response of CR, PR or MR (for RAPNO and RANO-LGG criteria). The ‘safety’ population consisted of all patients enrolled in the trial who received at least one dose of trial treatment. This report presents efficacy data from the evaluable population in arm 1 and the safety data from arms 1 and 2 as of a 5 June 2023 data cutoff.

The primary endpoint analysis was performed in the arm 1 evaluable population, which included all enrolled patients who received at least one dose of tovorafenib and had measurable disease at baseline per RANO-HGG criteria as determined by the IRC. A sample size of 60 patients in the evaluable population was considered to provide 88% power to reject the null hypothesis ORR of 21%, at the two-sided 0.05 level, assuming that the true underlying ORR of tovorafenib was 40% in this disease population. An exact binomial test was used for hypothesis testing. Responses per RAPNO and RANO-LGG criteria were evaluated in evaluable populations, which included all enrolled patients who received at least one dose of tovorafenib and had measurable disease at baseline per RAPNO and RANO-LGG criteria, respectively, as determined by the IRC.

Prespecified subgroup analysis of the uniformity of the treatment effects for ORR in arm 1 was planned for subgroups defined by *BRAF* alteration (*BRAF* fusion versus BRAF mutation), number of prior lines of therapies, prior MAPKi status (prior MEKi and/or prior BRAFi), sex, age group (6 months to <2 years of age, 2 years to <6 years of age, 6 years to <12 years of age, 12 years to <16 years of age and 16 years to ≤25 years of age) and race.

The ORR and CBR were calculated with 95% CIs determined using the Clopper–Pearson method. PFS and DOR were estimated by the Kaplan–Meier method and were summarized along with the corresponding two-sided 95% CI. Waterfall plots were generated for each patient’s best percentage change in sum of perpendicular diameters of measurable lesions.

Safety endpoints were analyzed using descriptive statistics based on the safety population in arms 1 and 2. AEs were coded by system organ class and preferred terms using the Medical Dictionary for Regulatory Activities (MedDRA) version 23.1 and graded according to the National Cancer Institute CTCAE version 5.0.

Statistical analyses were carried out using SAS version 9.4.

### Management of cutaneous AEs

Guidance for the management of rash/dermatitis was included in the trial protocol, in line with the stepwise approach proposed by Song et al.^53^ for the prevention and treatment of common cutaneous adverse reactions to BRAF, MEK and mTOR inhibitors in children with CNS tumors^53^. Dermatology assessment was performed at baseline and was symptom directed thereafter. Patients were to be referred to a dermatology department if cutaneous symptoms were impairing function (for example, if the patient could not sleep or sit still) or were psychosocially bothersome and/or when management techniques failed to resolve the condition. It was recommended that all trial patients followed a gentle skin care routine comprising short lukewarm showers/baths, the use of unscented, gentle cleansers and the application of unscented thick moisturizers (creams over lotions) immediately after showers. In addition, the prophylactic use of SPF 30+ sunscreen whenever going outside, with this reapplied every 2 h, and the wearing of sun-protective clothing were recommended for all patients. Specific guidance was also provided to manage mild or moderate/severe follicular, eczematous, paronychia or periungual reactions or hand–foot syndrome.

In the event of grade 2 macular or papular eruption, erythema with pruritus or other associated symptoms, localized desquamation or other lesions covering less than 50% of body surface area (BSA), tovorafenib dose reduction by one dose level once weekly could be considered. In the event of grade 3 or higher severe, generalized erythroderma or macular, papular or vesicular eruption, desquamation covering ≥50% of BSA or generalized exfoliative, ulcerative or bullous dermatitis, tovorafenib dosing was to be delayed until the condition improved, a dermatologist was consulted and a dose reduction by two dose levels or holding tovorafenib administration until resolution to grade 1 or baseline could be considered.

### Key protocol amendments

#### Current version 3.0, 21 October 2021

The protocol was amended primarily to add two new arms to the trial, to add a powder for reconstitution formulation of tovorafenib and to incorporate other changes based on feedback from regulatory authorities. This version of the full trial protocol (some confidential information redacted) is in the Supplementary Information supporting the article.

#### Version 2.0, 23 October 2020

The protocol was amended primarily to change the recommended phase 2 dose of tovorafenib from 530 mg m^−^^2^ to 420 mg m^−^^2^ and decrease the maximum dose from 800 mg to 600 mg once weekly. The planned number of cycles was reduced from 27 cycles to 26 cycles (patients could continue on trial treatment beyond this if criteria were met), and the upper age limit was increased from 18 years to 25 years, inclusive.

### Clinical trial registration

The study is registered on ClinicalTrials.gov as NCT04775485 and on EudraCT as 2020-003657-30.

### Reporting summary

Further information on research design is available in the Nature Portfolio Reporting Summary linked to this article.

## Online content

Any methods, additional references, Nature Portfolio reporting summaries, source data, extended data, supplementary information, acknowledgements, peer review information; details of author contributions and competing interests; and statements of data and code availability are available at 10.1038/s41591-023-02668-y.

## Supplementary information

Supplementary Tables 1 and 2 and trial protocol.

Reporting Summary

## Figures and Tables

**Extended Data Fig. 3:**
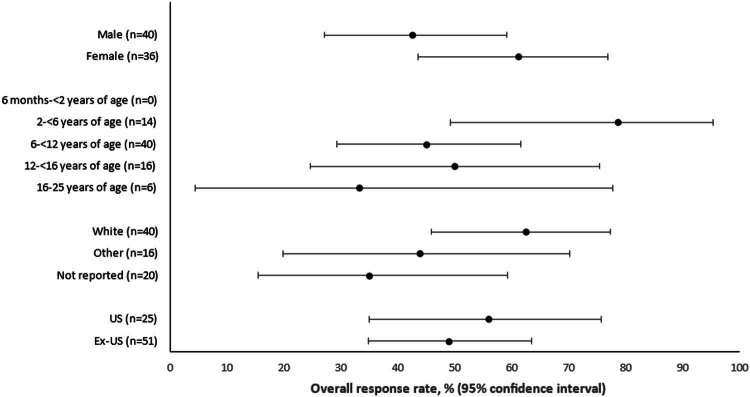
Forest plot of response according to RAPNO in subgroups defined by baseline characteristics. Filled circles represent the overall response rates, and whiskers represent the 95% confidence intervals. Other races included Asian (n = 5), Black (n = 2), Multiple (n = 3), and Other (n = 6). There were no Native Hawaiian or other Pacific Islander or American Indian or Alaska Native participants. No race information was missing. Ex, External to; US, United States.

**Extended Data Table 1:**
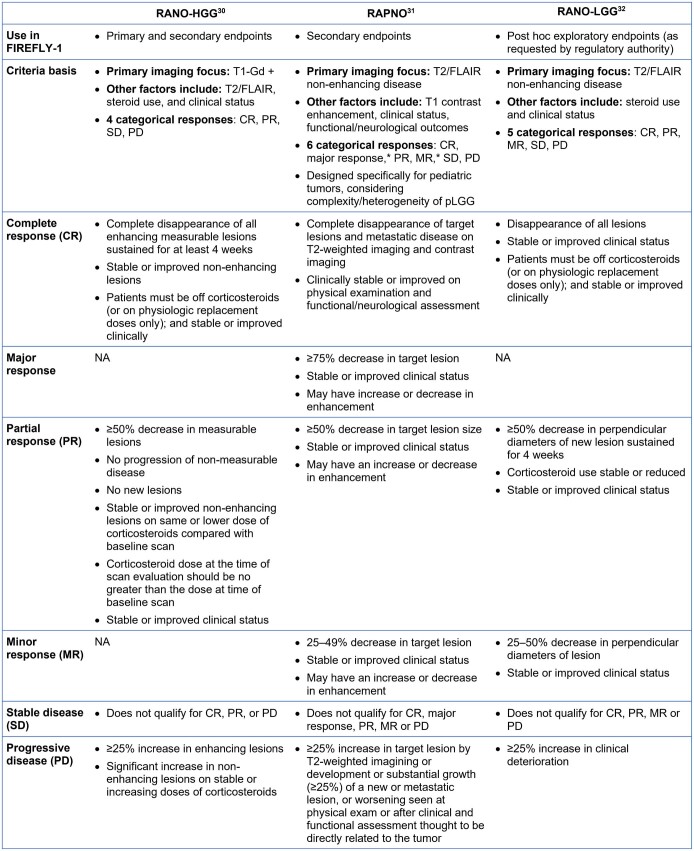
Comparison of radiological response assessment criteria

*Major and minor response guidelines included as recommendations if they were part of the response definition used within a specific clinical trial. Gd, gadolinium; NA, not applicable.

**Extended Data Table 2:**
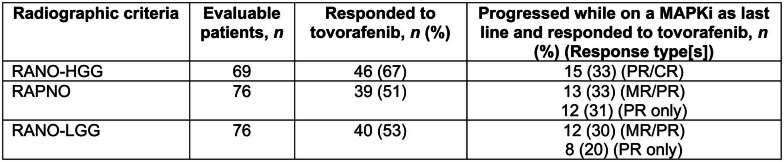
Responses to tovorafenib in patients on a MAPKi as their most recent prior therapy and discontinued due to progression*

*Note: RAPNO-LGG and RANO-LGG endpoints include MR.

## Data Availability

The trial protocol (confidential information redacted) is provided in the Supplementary Information. The authors declare that all data supporting the findings of this trial are available within the article and the Supplementary Information. Requests for full datasets will be considered after completion of the trial and analysis of the data, which is anticipated to be in December 2024. To request individual participant data associated with any Day One Biopharmaceuticals clinical trial, email clinical@dayonebio.com. All requests will be evaluated within 8 weeks.
