# Author Correction: The type II RAF inhibitor tovorafenib in relapsed/refractory pediatric low-grade glioma: the phase 2 FIREFLY-1 trial

**DOI:** 10.1038/s41591-025-03709-4

**Published:** 2025-04-16

**Authors:** Lindsay B. Kilburn, Dong-Anh Khuong-Quang, Jordan R. Hansford, Daniel Landi, Jasper van der Lugt, Sarah E. S. Leary, Pablo Hernáiz Driever, Simon Bailey, Sébastien Perreault, Geoffrey McCowage, Angela J. Waanders, David S. Ziegler, Olaf Witt, Patricia A. Baxter, Hyoung Jin Kang, Timothy E. Hassall, Jung Woo Han, Darren Hargrave, Andrea T. Franson, Michal Yalon Oren, Helen Toledano, Valérie Larouche, Cassie Kline, Mohamed S. Abdelbaki, Nada Jabado, Nicholas G. Gottardo, Nicolas U. Gerber, Nicholas S. Whipple, Devorah Segal, Susan N. Chi, Liat Oren, Enrica E. K. Tan, Sabine Mueller, Izzy Cornelio, Lisa McLeod, Xin Zhao, Ashley Walter, Daniel Da Costa, Peter Manley, Samuel C. Blackman, Roger J. Packer, Karsten Nysom

**Affiliations:** 1 Children’s National Hospital, Washington, DC USA; 2 Children’s Cancer Centre, Royal Children’s Hospital Melbourne, Melbourne, Victoria Australia; 3 Michael Rice Centre for Hematology and Oncology, Women’s and Children’s Hospital, Adelaide, South Australia Australia; 4 South Australia Health and Medical Research Institute, Adelaide, Australia; South Australian Immunogenomics Cancer Institute, University of Adelaide, Adelaide, South Australia Australia; 5 Duke University, Durham, NC USA; 6 Princess Máxima Center for Pediatric Oncology, Utrecht, The Netherlands; 7 Cancer and Blood Disorders Center, Seattle Children’s, Seattle, WA USA; 8 Charité Universitätsmedizin Berlin, corporate member of Freie Universität Berlin and Humboldt-Universität Berlin, German HIT-LOGGIC-Registry for LGG in Children and Adolescents, Berlin, Germany; 9 Great North Children’s Hospital and Newcastle University Centre for Cancer, Newcastle-upon-Tyne, UK; 10 CHU Sainte-Justine, Université de Montréal, Montréal, Quebec Canada; 11 Sydney Children’s Hospitals Network, Westmead, New South Wales Australia; 12 Ann & Robert H. Lurie Children’s Hospital, Chicago, IL USA; 13 Kids Cancer Centre, Sydney Children’s Hospital, Randwick, New South Wales Australia; 14 Children’s Cancer Institute, Lowy Cancer Research Centre, University of New South Wales, Sydney, New South Wales Australia; 15 School of Clinical Medicine, University of New South Wales, Sydney, New South Wales Australia; 16 Hopp Children’s Cancer Center Heidelberg (KiTZ), Heidelberg, Germany; 17 Clinical Cooperation Unit, Pediatric Oncology, German Cancer Research Center (DKFZ), Heidelberg, Germany; 18 Department of Pediatric Oncology, Hematology, Immunology and Pulmonology, Heidelberg University Hospital, Heidelberg, Germany; 19 German Cancer Consortium (DKTK), Heidelberg, Germany; 20 National Center for Tumor Diseases (NCT), Heidelberg, Germany; 21 Texas Children’s Cancer Center, Texas Children’s Hospital, Baylor College of Medicine, Houston, TX USA; 22 Department of Pediatrics, Seoul National University College of Medicine, Seoul National University Cancer Research Institute, Seoul National University Children’s Hospital, Seoul, Republic of Korea; 23 Children’s Health Queensland Hospital and Health Service, South Brisbane, QLD Australia; 24 Severance Hospital, Yonsei University Health System, Seoul, Republic of Korea; 25 UCL Great Ormond Street Institute of Child Health and Great Ormond Street Hospital for Children, London, UK; 26 C.S. Mott Children’s Hospital, University of Michigan Medical School, Ann Arbor, MI USA; 27 Pediatric Hemato-Oncology, Sheba Medical Center, Ramat Gan, Israel; 28 Department of Pediatric Oncology, Schneider Children’s Medical Center, Petach Tikva, and Faculty of Medicine, Tel Aviv University, Tel Aviv, Israel; 29 Department of Pediatrics, Centre Mère-Enfant Soleil du CHU de Québec-Université Laval, Quebec City, Quebec Canada; 30 Division of Oncology, Department of Pediatrics, Children’s Hospital of Philadelphia, University of Pennsylvania Perelman School of Medicine, Philadelphia, PA USA; 31 Division of Hematology and Oncology, Department of Pediatrics, School of Medicine, Washington University, St. Louis, MO USA; 32 McGill University Health Centre (MUHC), Montreal Children’s Hospital (MCH), Montreal, Quebec Canada; 33 Department of Pediatric and Adolescent Oncology and Hematology, Perth Children’s Hospital, Perth, Australia, and Brain Tumor Research Program, Telethon Kids Cancer Centre, Telethon Kids Institute, University of Western Australia, Perth, Western Australia Australia; 34 Department of Oncology, University Children’s Hospital, Zurich, Switzerland; 35 Primary Children’s Hospital and University of Utah, Salt Lake City, UT USA; 36 NYU Langone Health, New York, NY USA; 37 Pediatric Neuro-Oncology, Department of Pediatrics, Dana-Farber/Boston Children’s Cancer and Blood Disorders Center, Boston, MA USA; 38 Department of Hematology & Oncology, Rambam Healthcare Campus, Haifa, Israel; 39 Haematology/Oncology Service, KK Women’s and Children’s Hospital, Singapore, Singapore; 40 Department of Neurology, Neurosurgery and Pediatrics, University of California, San Francisco, San Francisco, CA USA; 41 Day One Biopharmaceuticals, Brisbane, CA USA; 42 Division of Neurology, Brain Tumor Institute, Center for Neuroscience and Behavioral Medicine, Children’s National Hospital, Washington, DC USA; 43 Department of Pediatrics and Adolescent Medicine, Copenhagen University Hospital – Rigshospitalet, Copenhagen, Denmark

**Keywords:** Paediatric cancer, Targeted therapies, Drug development, Randomized controlled trials, Phase II trials

Correction to: *Nature Medicine* 10.1038/s41591-023-02668-y, published online 17 November 2023.

In the version of the article initially published, the colors in the Fig. 2 key were switched and have now been amended so that teal represents “Prior BRAFi/MEKi” and orange represents “BRAFi/MEKi-naive”. Additionally, in Fig. 2a, the tenth bar from the left was teal and has now been corrected to orange as the patient was BRAFi/MEKi-naive. These corrections have been made to the HTML and PDF versions of the article.

