# Supplementary material for: The type II RAF inhibitor tovorafenib in relapsed/refractory pediatric low-grade glioma: the phase 2 FIREFLY-1 trial
Source: Nat Med. 2023 Nov 17;30(1):207–17. doi: 10.1038/s41591-023-02668-y (PMC10803270; doi:10.1038/s41591-023-02668-y)
Supplement: Supplementary file 1 — Supplementary Tables 1 and 2 and trial protocol. [file 41591_2023_2668_MOESM1_ESM.pdf]

nature medicine

Article

https://doi.org/10.1038/s41591-023-02668-y

The type II RAF inhibitor tovorafenib in relapsed/refractory pediatric low-grade glioma: the phase 2 FIREFLY-1 trial

In the format provided by the authors and unedited

Supplementary Table 1. Treatment-related adverse events (safety analysis set, *n*=137)

Preferred term, *n*, (%)	Treatment-related AEs	
	Any grade	Grade ≥3
Patients with any AE	134 (98)	58 (42)
Hair color changes	104 (76)	0
Elevated CPK	77 (56)	16 (12)
Anemia	67 (49)	14 (10)
Fatigue	60 (44)	6 (4)
Maculopapular rash	56 (41)	11 (8)
Hypophosphatemia	48 (35)	0
Dry skin	45 (33)	0
Elevated LDH	42 (31)	0
Dermatitis acneiform	41 (30)	1 (1)
Increased AST	41 (30)	4 (3)
Paronychia	32 (23)	2 (1)
Pruritus	32 (23)	1 (1)
Constipation	31 (23)	0
Headache	29 (21)	0
Decreased appetite	28 (20)	4 (3)
Vomiting	28 (20)	3 (2)
Epistaxis	27 (20)	0
Nausea	25 (18)	0
Increased ALT	24 (18)	7 (5)
Alopecia	23 (17)	0
Eczema	22 (16)	2 (1)
Weight decreased	21 (15)	0
Face edema	20 (15)	0
Photosensitivity reaction	19 (14)	1 (1)
Erythema	18 (13)	1 (1)
Decreased growth velocity	17 (12)	7 (5)
Pyrexia	17 (12)	1 (1)
Increased bilirubin	16 (12)	1 (1)
Hypokalemia	15 (11)	3 (2)
Decreased lymphocyte count	15 (11)	0
Diarrhea	14 (10)	1 (1)
Erythematous rash	14 (10)	1 (1)
Rash	14 (10)	0
Hypocalcemia	13 (9)	1 (1)
Myalgia	13 (9)	0
Stomatitis	13 (9)	0
Flushing	12 (9)	0

Hypoalbuminemia	12 (9)	0
Decreased WBC count	11 (8)	1 (1)
Pain in extremity	11 (8)	0
Tumor hemorrhage	10 (7)	3 (2)
Arthralgia	10 (7)	0
Periorbital edema	10 (7)	0
Decreased neutrophil count	9 (7)	3 (2)
Cheilitis	9 (7)	0
Decreased platelet count	9 (7)	0
Upper abdominal pain	9 (7)	0
Abdominal pain	8 (6)	0
Angular cheilitis	8 (6)	0
Dry lip	8 (6)	0
Gingival bleeding	8 (6)	0
Muscle spasms	8 (6)	0
Papular rash	8 (6)	0
Eosinophilia	7 (5)	0
Lethargy	6 (4)	1 (1)
Weight increased	6 (4)	1 (1)
Abnormal hair texture	6 (4)	0
Skin hypopigmentation	6 (4)	0
Decreased phosphorus	5 (4)	1 (1)
Pericardial effusion	5 (4)	1 (1)
Conjunctivitis	5 (4)	0
Acne	5 (4)	0
Amenorrhea	5 (4)	0
Peripheral edema	5 (4)	0
Skin discoloration	5 (4)	0
Skin hyperpigmentation	5 (4)	0
Folliculitis	4 (3)	1 (1)
Hyponatremia	4 (3)	1 (1)
Blurred vision	4 (3)	0
Contusion	4 (3)	0
Dermatitis	4 (3)	0
Dizziness	4 (3)	0
Drug eruption	4 (3)	0
Hypernatremia	4 (3)	0
Seborrheic dermatitis	4 (3)	0
Skin exfoliation	4 (3)	0
Skin infection	4 (3)	0
Back pain	3 (2)	0

Blepharitis	3 (2)	0
Dry eye	3 (2)	0
Dysgeusia	3 (2)	0
Eye swelling	3 (2)	0
Hordeolum	3 (2)	0
Melanocytic naevus	3 (2)	0
Onychoclasis	3 (2)	0
Photophobia	3 (2)	0
Xeroderma	3 (2)	0
Follicular rash	2 (1)	1 (1)
Prolonged APTT	2 (1)	1 (1)
Atopic dermatitis	2 (1)	0
Bullous dermatitis	2 (1)	0
Decreased TSH	2 (1)	0
Depression	2 (1)	0
Diabetes insipidus	2 (1)	0
Ephelides	2 (1)	0
Eyelash discoloration	2 (1)	0
Eye pruritus	2 (1)	0
Hand dermatitis	2 (1)	0
Hypertension	2 (1)	0
Hypoglycemia	2 (1)	0
Hypomagnesemia	2 (1)	0
Increased cholesterol	2 (1)	0
Increased creatinine	2 (1)	0
Increased TSH	2 (1)	0
Ingrowing nail	2 (1)	0
Insomnia	2 (1)	0
Irregular menstruation	2 (1)	0
Irritability	2 (1)	0
Localized infection	2 (1)	0
Macular rash	2 (1)	0
Muscular weakness	2 (1)	0
Nasal congestion	2 (1)	0
Nasopharyngitis	2 (1)	0
Ocular icterus	2 (1)	0
Onychomadesis	2 (1)	0
Papule	2 (1)	0
Urinary incontinence	2 (1)	0
Urticaria	2 (1)	0
Vertigo	2 (1)	0

Cough	2 (1)	0
Upper RTI	2 (1)	0
Cholecystitis	1 (1)	1 (1)
CSF circulation disorder	1 (1)	1 (1)
Eye infection viral	1 (1)	1 (1)
Increased GGT	1 (1)	1 (1)
Optic nerve disorder	1 (1)	1 (1)
Pruritic rash	1 (1)	1 (1)
Seizure	1 (1)	1 (1)
Systemic infection	1 (1)	1 (1)
Abnormal thyroid function test	1 (1)	0
Acquired achromotrichia	1 (1)	0
Acquired dacryostenosis	1 (1)	0
Allergic dermatitis	1 (1)	0
Altered mood	1 (1)	0
Androgenetic alopecia	1 (1)	0
Angiopathy	1 (1)	0
Anorectal cellulitis	1 (1)	0
Anxiety	1 (1)	0
Aphthous ulcer	1 (1)	0
Asthenopia	1 (1)	0
Atelectasis	1 (1)	0
Bacterial infection	1 (1)	0
Cardiomegaly	1 (1)	0
Chalazion	1 (1)	0
Chapped lips	1 (1)	0
Chest discomfort	1 (1)	0
Chills	1 (1)	0
Cholelithiasis	1 (1)	0
Conjunctival papillae	1 (1)	0
Corneal lesion	1 (1)	0
Decreased bicarbonate	1 (1)	0
Decreased ECG T wave amplitude	1 (1)	0
Decreased haptoglobin	1 (1)	0
Decreased hematocrit	1 (1)	0
Decreased hemoglobin	1 (1)	0
Decreased iron	1 (1)	0
Decreased RBC count	1 (1)	0
Decreased total protein	1 (1)	0
Delayed menstruation	1 (1)	0
Dysarthria	1 (1)	0

Dyspepsia	1 (1)	0
Dystonia	1 (1)	0
Dysuria	1 (1)	0
Ear infection	1 (1)	0
Eyelid eczema	1 (1)	0
Episcleritis	1 (1)	0
Erythema multiforme	1 (1)	0
Erythema of eyelid	1 (1)	0
Eye irritation	1 (1)	0
Eyelash hypopigmentation	1 (1)	0
Eyelid infection	1 (1)	0
Facial pain	1 (1)	0
Flank pain	1 (1)	0
Furuncle	1 (1)	0
GERD	1 (1)	0
Gingival pain	1 (1)	0
Glaucoma	1 (1)	0
Growth disorder	1 (1)	0
Growth failure	1 (1)	0
Hand-foot-and-mouth disease	1 (1)	0
Hematochezia	1 (1)	0
Hemolysis	1 (1)	0
Hemorrhoids	1 (1)	0
Hot flush	1 (1)	0
Hyperkalemia	1 (1)	0
Hypermagnesemia	1 (1)	0
Hyperparathyroidism	1 (1)	0
Hypersomnia	1 (1)	0
Hyperthyroidism	1 (1)	0
Hypoacusis	1 (1)	0
Hypochloremia	1 (1)	0
Hypoesthesia	1 (1)	0
Hypoxia	1 (1)	0
Hypoproteinemia	1 (1)	0
Impetigo	1 (1)	0
Increased ALP	1 (1)	0
Increased chloride	1 (1)	0
Increased free T3	1 (1)	0
Increased hepatic enzyme	1 (1)	0
Increased INR	1 (1)	0
Increased intraocular pressure	1 (1)	0

Increased lymphocyte count	1 (1)	0
Increased mean cell volume	1 (1)	0
Increased thyroxine	1 (1)	0
Increased troponin T	1 (1)	0
Increased urine output	1 (1)	0
Increased urobilinogen urine	1 (1)	0
Infected dermatitis	1 (1)	0
Influenza-like illness	1 (1)	0
Injection site erythema	1 (1)	0
Intracranial tumor hemorrhage	1 (1)	0
Jaundice	1 (1)	0
Joint stiffness	1 (1)	0
Keratosis pilaris	1 (1)	0
Left ventricular hypertrophy	1 (1)	0
Lip disorder	1 (1)	0
Lip infection	1 (1)	0
Lip injury	1 (1)	0
Lip ulceration	1 (1)	0
Lymphopenia	1 (1)	0
Memory impairment	1 (1)	0
Micturition urgency	1 (1)	0
Miliaria	1 (1)	0
Molluscum contagiosum	1 (1)	0
Mouth ulceration	1 (1)	0
Muscle fatigue	1 (1)	0
Muscle tightness	1 (1)	0
Nail disorder	1 (1)	0
Nail ridging	1 (1)	0
Nasal discomfort	1 (1)	0
Nasal inflammation	1 (1)	0
Nasal ulcer	1 (1)	0
Neutropenia	1 (1)	0
Ocular hyperemia	1 (1)	0
Odynophagia	1 (1)	0
Onychomycosis	1 (1)	0
Oral candidiasis	1 (1)	0
Oral herpes	1 (1)	0
Oral hypoesthesia	1 (1)	0
Oral pain	1 (1)	0
Oropharyngeal pain	1 (1)	0
Otorrhea	1 (1)	0

Pain	1 (1)	0
Pain of skin	1 (1)	0
Paresthesia	1 (1)	0
Periorbital swelling	1 (1)	1 (1)
Peripheral swelling	1 (1)	0
Petechiae	1 (1)	0
Pharyngitis	1 (1)	0
Photopsia	1 (1)	0
Platelet count increased	1 (1)	0
Pleural effusion	1 (1)	0
Pollakiuria	1 (1)	0
Proctitis	1 (1)	0
Prolonged ECG QT	1 (1)	0
Purpura	1 (1)	
Salivary hypersecretion	1 (1)	0
Scab	1 (1)	0
Scleral discoloration	1 (1)	0
Sensitive skin	1 (1)	0
Skin depigmentation	1 (1)	0
Skin hypertrophy	1 (1)	0
Skin irritation	1 (1)	0
Skin lesion	1 (1)	0
Skin toxicity	1 (1)	0
Skin ulcer	1 (1)	0
Sleep disorder	1 (1)	0
Slow response to stimuli	1 (1)	0
Somnolence	1 (1)	0
Staphylococcal infection	1 (1)	0
Staphylococcal skin infection	1 (1)	0
Sunburn	1 (1)	0
Swelling face	1 (1)	0
Telangiectasia	1 (1)	0
Throat clearing	1 (1)	0
Throat irritation	1 (1)	0
Thrombocytopenia	1 (1)	0
Thrombocytosis	1 (1)	0
Tongue eruption	1 (1)	0
Tooth discoloration	1 (1)	0
Varicella	1 (1)	0
Ventricular extrasystoles	1 (1)	0
Viral upper RTI	1 (1)	0

Visual impairment	1 (1)	0
Vulval edema	1 (1)	0
Vulvovaginal pain	1 (1)	0
Vulvovaginal pruritus	1 (1)	0
Uncoded	1 (1)	0

TRAEs and laboratory abnormalities (any grade and grade ≥3 occurring in ≥1 patient).
TRAEs and laboratory abnormalities: Patients are counted only once per event and are shown in the worst CTCAE grade that was reported for each event they experienced.
MedDRA version 23.1; CTCAE version 5.0.
AE, adverse event; AKI, acute kidney injury; ALP, alkaline phosphatase; ALT, alanine aminotransferase; APTT, activated partial thromboplastin time; AST, aspartate aminotransferase; COVID-19, coronavirus disease 2019; CPK, creatine phosphokinase; CSF, cerebrospinal fluid; CTCAE, Common Terminology Criteria for Adverse Events; ECG, electrocardiogram; GERD, gastroesophageal reflux disease; GGT, gamma-glutamyl transferase; INR, international normalized ratio; LDH, lactate dehydrogenase; MedDRA, Medical Dictionary for Regulatory Activities; RBC, red blood cell; RTI, respiratory tract infection; T3, triiodothyronine; TEAE, treatment-emergent adverse event; TRAE, treatment-related adverse event; TSH, thyroid-stimulating hormone; WBC, white blood cell.

Supplementary Table 2. Enrollment centers

Center	Location
Children's Health Queensland Hospital and Health Service	Queensland, Australia
Children's Hospital at Westmead	Western Sydney, Australia
Perth Children's Hospital	Western Australia, Australia
Royal Children's Hospital	Victoria, Australia
Sydney Children's Hospital	Sydney, Australia
Centre Hospitalier Universitaire Ste Justine	Québec, Canada
Centre Mere-Enfant Soleil du CHU – Pediatric Hemato-Oncology	Québec, Canada
McGill University Health Centre (MUHC) - The Montreal Children's Hospital	Québec, Canada
Copenhagen University Hospital - Rigshospitalet	Copenhagen, Denmark
Charite - Campus Virchow Klinikum	Berlin, Germany
Universitatsklinikum Heidelberg	Heidelberg, Germany
Rambam Health Care Campus	Haifa, Israel
Schneider Children's Medical Center of Israel	Petah Tikva, Israel
The Chaim Sheba Medical Center	Tel Aviv, Israel
Prinses Máxima Centrum	Utrecht, Netherlands
Seoul National University Hospital	Seoul, South Korea
Severance Hospital, Yonsei University Health System	Seoul, South Korea
KK Women's and Children's Hospital	Singapore
Kinderspital Zürich	Zürich, Switzerland
Great Ormond Street Hospital for Children	London, United Kingdom
The Newcastle Hospitals NHS Trust	Newcastle upon Tyne, United Kingdom
Ann & Robert H. Lurie Children's Hospital - Oncology	Illinois, United States
Children's Hospital of Philadelphia	Pennsylvania, United States
Children's National Medical Center	The District of Columbia, United States
Dana-Farber Cancer Institute-Medicine	Massachusetts, United States
Duke University Medical Center	North Carolina, United States
NYU Langone Health	New York, United States
Seattle Children's Hospital	Washington, United States
St. Louis Children's Hospital	Missouri, United States
Texas Children's Hospital	Texas, United States
University of Michigan	Michigan, United States
University of Utah	Utah, United States

CLINICAL PROTOCOL DAY101-001/PNOC026

FIREFLY-1: A Phase 2, Open-Label, Multicenter Study to Evaluate the Safety and Efficacy of the Oral Pan-RAF Inhibitor DAY101 in Pediatric Patients with RAF-Altered, Recurrent or Progressive Low-Grade Glioma and Advanced Solid Tumors

**Investigational Product:**	DAY101
**Protocol Number:**	DAY101-001
**IND #:**	108340
**EudraCT #:**	2020-003657-30
**Development Phase:**	2
**Sponsor:**	DOT Therapeutics-1 Inc. (Day One)<br>395 Oyster Point Boulevard<br>Suite 217<br>South San Francisco, CA 94080
**Current Version 3.0**	21 October 2021
**Version 2.5 (Germany)**	19 March 2021
**Version 2.4 (Germany)**	12 February 2021
**Version 2.3 (Switzerland)**	08 January 2021
**Version 2.2 (South Korea)**	21 December 2020
**Version 2.1 (UK Only)**	09 November 2020
**Version 2.0**	23 October 2020
**Original Version 1.0**	28 July 2020

Confidentiality Statement

This document contains confidential information that is the property of Day One, and may not be disclosed to anyone other than the recipient clinical investigator, research staff, and respective members of the Institutional Review Board//Research Ethics Board/Independent Ethics Committee/Human Research Ethics Committee. This information cannot be used for any purpose other than the evaluation of conduct of the clinical investigation without the prior written consent of Day One.

Protocol Approval Page

**Protocol Title:**	A Phase 2, Open-Label, Multicenter Study to Evaluate the Safety and Efficacy of the Oral Pan-RAF Inhibitor DAY101 in Pediatric Patients with RAF-Altered, Recurrent or Progressive Low-Grade Glioma and Advanced Solid Tumors
**Protocol Number:**	DAY101-001
**Current Version 3.0**	21 October 2021

The current version of the protocol has been reviewed and approved.

Samuel C. Blackman, MD, PHD
Chief Medical Officer

21-October-2021
Date

I have carefully read Protocol DAY101-001 entitled "A Phase 2, Open-Label, Multicenter Study to Evaluate the Safety and Efficacy of the Oral Pan-RAF Inhibitor DAY101 in Pediatric Patients with RAF-Altered, Recurrent or Progressive Low-Grade Glioma and Advanced Solid Tumors." I confirm that I have read and agree to conduct the clinical study as outlined in the protocol and in compliance with Good Clinical Practice (GCP), the Declaration of Helsinki as amended, and all other applicable regulatory requirements. Furthermore, I understand that the Sponsor, Day One, and the Institutional Review Board/Research Ethics Board/Independent Ethics Committee/Human Research Ethics Board (IRB/REB/IEC/HREC) must approve any changes to the protocol in writing before implementation.

I agree to inform all patients that the study drug DAY101 is being used for investigational purposes, and I will ensure that the requirements related to obtaining informed consent and pediatric assent are in accordance with International Council for Harmonisation (ICH) guidelines for GCP and local regulatory requirements.

I agree, on behalf of myself and all other personnel involved in the clinical study who are employed by me, to maintain confidentiality of all information received or developed in connection with this protocol. All data pertaining to this study will be provided to Day One and any presentation or publication of study data will be reviewed by Day One before release.

Principal Investigator's Signature

Date

Print Principal Investigator's Name

PROTOCOL AMENDMENT SUMMARY OF CHANGES

Protocol DAY101-001 has been amended primarily to add two new arms to the study, add a powder for reconstitution formulation of DAY101, and incorporate changes based on feedback from regulatory authorities.

Section(s)	Description of Change	Brief Rationale
Synopsis	The protocol synopsis has been updated to reflect the changes in the protocol.	For consistency with revisions.
Global	Two new arms have been added to the protocol: Arm 2 is an extension cohort to enroll patients with a low-grade glioma harboring an activating RAF alteration (e.g., BRAF or CRAF/RAF1 fusion or BRAF V600 mutations) and will provide treatment options to patients following the closure of Arm 1 and prior to commercial availability of DAY101. Arm 3 will enroll patients with advanced solid tumors harboring activating RAF fusions (e.g., BRAF or CRAF/RAF1 fusions).<br>The background, rationale, objectives, design, eligibility criteria, assessments, and statistical analyses sections have been revised to incorporate these new arms.	Based on data obtained from other DAY101 studies and feedback from health authority.
Global	[redacted]	[redacted]
1.1.1, 1.1.2, 1.2.1, 1.3.1	The introduction has been revised to refer to BRAF fusion instead of BRAF wild-type fusion.	Administrative clarification.
1.1.2	The summary of pediatric low-grade gliomas has been updated.	To include additional background on disease and treatment-related morbidities in patients with pediatric low-grade glioma.
1.3.3	The benefit/risk assessment has been revised to include a summary of risks from nonclinical studies and the Phase 1 clinical study, dosing considerations, drug metabolism, considerations for required study procedures for the pediatric population, the safety monitoring and risk management plan, a summary of potential benefits, and an integrated benefit/risk assessment.	Based on feedback from health authority.
2	The RAPNO criteria will be determined by the IRC only (Arms 1 and 2).	Administrative clarification.
2, 6.9.2, 7.3.3	The Pediatrics Quality of Life™—Cancer has been specified as a health-related QoL assessment.	Administrative clarification for consistency with planned study objectives.

Section(s)	Description of Change	Brief Rationale
2, 6.6.4, 6.9.1.2, Appendix I	The requirements for specific visual acuity testing procedures have been revised to allow age-appropriate assessments.	To allow for regional differences and age-appropriate testing procedures across sites and countries.
3.1	Clarified the reconsenting process for patients being treated beyond radiographic disease progression with DAY101.	Based on feedback from health authority.
3.1, 5.1, 5.2	A powder for reconstitution formulation has been added. The investigational product description and general dosing instructions have been revised to include details specific to this formulation.	To enable use of a liquid formulation of DAY101 in patients who are unable to swallow tablets.
3.1.4	The definition for end of study has been updated to the date when the last visit occurs for the last patient enrolled.	Based on feedback from health authority.
3.2	The number of study sites has changed from 35 to 40.	To enable enrollment of pediatric patients with advanced solid tumors.
4.1 (Inclusion Criterion 8)	Inclusion Criterion 8 has been clarified to add criteria for patients with ongoing retinopathy from prior anticancer therapy.	To minimize the risk of significant ocular toxicities in patients with pre-existing retinopathy.
4.1 (Inclusion Criterion 12)	Removed the table of maximum and minimum serum creatinine levels by age from Inclusion Criterion 12.<br>The eligibility requirement for patients to have an international normalized ratio < 2.0 has been removed from Inclusion Criterion 12.<br>The limit for estimated glomerular filtration rate has been changed from ≥ 30 to ≥ 60 mL/min/1.73 m^2.	Based on feedback from health authority.<br>Administrative clarification.<br>Based on feedback from health authority.
4.1 (Inclusion Criterion 17)	The contraception methods have been changed from double effective to highly effective birth control.	For consistency with Appendix K, based on feedback from health authority.
4.1 (Inclusion Criterion 18)	Inclusion Criterion 18 has been revised to allow patients who are able to swallow liquid or are willing to comply with feeding tube administration to be eligible for the study.	To allow enrollment of patients who are unable or unwilling to swallow tablets.
5.1	The storage conditions for DAY101 tablets have been updated to between 15°C and 25°C rather than between 15°C and 30°C.	To align with the IMPD.
5.2, 7.1	The dose-limiting toxicity (DLT) section has been consolidated with dose modifications and management of treatment-related events (Sections 5.2.2 and 5.2.3). Treatment-emergent adverse events (TEAEs) previously considered DLTs for patients enrolled in the safety run-in have been incorporated into Table 9 requiring dose interruption.	Terminology changes, as the safety run-in portion of this study, which planned to evaluate a dose of 530 mg/m^2, will no longer be conducted (previously removed in Protocol v2.0).

Section(s)	Description of Change	Brief Rationale
5.2.1	The restriction with regard to intake of food has been removed: patients can now be fed or fasted.<br>Clarified that the dose of study intervention should be increased or decreased for any change in body surface area (BSA) greater than 10%.	[redacted]<br>Administrative clarification.
5.2.3.2	Added instruction for patients to monitor for signs and symptoms of photosensitivity.	Based on feedback from health authority.
5.2.3.5	The increased liver transaminase recommendation for asymptomatic ≥ Grade 3 AST or ALT AND > 2 × increase from baseline has been revised to "withhold DAY101 until the laboratory abnormality resolves to < 5 × ULN or baseline" rather than "withhold DAY101 until the laboratory abnormality resolves to < 5 × ULN AND baseline."	Administrative clarification.
5.3.2, 6.17, 8.2	Additional references to the safety run-in portion of the study have been removed.	For consistency with the removal of the safety run-in in V2.0 of the protocol.
5.3.3	Herbal supplements are now prohibited during the study.	Based on feedback from the local review board
5.3.3, Appendix C	Medications that are substrates of breast cancer resistance protein (BCRP) have been added as prohibited concomitant medications. A table of BCRP substrates was added to Appendix C.	Based on feedback from health authority.
6.6.4, 6.9.1.2, Appendix A	On-treatment ophthalmology examinations and visual acuity assessments have been clarified to be only required for patients with optic pathway glioma or patients with underlying visual deficits related to the primary malignancy. For all other patients, examinations are only required at screening, and symptom-directed examinations may be completed at subsequent visits.	For consistency with standard clinical practice.
6.6.7	Added assessments of pubertal development according to the Tanner Stages.	Based on feedback from health authority.
6.6.8.1	Required echocardiogram/ multiple-gated acquisition and 12-lead electrocardiogram assessments were added at the end of treatment (EOT) visit.	Based on feedback from health authority.
6.7.3	Phosphorous will now be assessed as part of the serum chemistry panel.<br>International normalized ratio, cholesterol, and direct bilirubin will no longer be assessed.	To follow as part of regular safety monitoring.<br>To reduce unnecessary analyte collection.
6.7.4	Thyroid stimulating hormone (TSH) will now be assessed as part of the thyroid function tests.	To ensure comprehensive monitoring of thyroid function.

Section(s)	Description of Change	Brief Rationale
6.8	Clarified that blood sample collections for DAY101 PK after Cycle 4 will be collected every subsequent third cycle through Cycle 13, rather than until Cycle 27.	To reduce patient sampling burden after adequate characterization of DAY101 PK through approximately one year after the initiation of therapy.
6.9.1.2, Appendix A	Clarification that visual acuity assessment may be performed based on local standard practice.	To allow assessment based on local standard practice.
7.3.1	The description of interim data review was removed.<br><br>The efficacy analyses section has been updated.	Based on feedback from health authority.<br><br>Based on feedback from health authority and incorporation of Arms 2 and 3.
8.1.4	Clarified that prompt notification means within 24 hours of awareness.<br>Removed US-specific details on safety reporting requirements to health authorities.	To provide global reporting instructions.
8.6, Appendix J	The definitions for assessing relationship to study intervention have been modified.	Based on feedback from health authority.
9.1.1	The applicable German regulations and guidelines have been added	Based on feedback from health authority.
Appendix A	The schedule of assessments has been updated to reflect the changes in the protocol.	For consistency.
Appendix E	The revised Response Evaluation Criteria in Solid Tumors: Revised RECIST Guideline (version 1.1) has been added.	To provide the RECIST v1.1 guidelines for evaluation of patients with advanced solid tumors in Arm 3.
Appendix F	References to cell line development have been removed.	No cell line development is planned.
Appendix F	Previous Appendix F (List of Research Uses of Tissue and Clinical Data) has been removed.	Administrative clarification.
Appendix K	The appendix has been revised to delete alternative birth control methods with a failure rate of > 1%. These methods are now considered unacceptable.	Based on feedback from health authority.

Additional minor formatting and editorial changes (not listed in the table above) have been made to improve clarity and to correct grammar and typographical errors. These include revisions to abbreviations and updates to literature references, as applicable.

PROTOCOL SYNOPSIS

TITLE:

FIREFLY-1: A Phase 2, Open-Label, Multicenter Study to Evaluate the Safety and Efficacy of the Oral Pan-RAF Inhibitor DAY101 in Pediatric Patients with RAF-Altered, Recurrent or Progressive Low-Grade Glioma and Advanced Solid Tumors

PROTOCOL NUMBER:

DAY101-001/PNOC026

STUDY SITES:

Approximately 40 institutions will participate in this study.

PHASE:

2

ARM 1 (LOW-GRADE GLIOMA) OBJECTIVES:

Primary Objective:

The primary objective of Arm 1 is to evaluate the overall response rate (ORR) as determined by an independent radiology review committee (IRC) and measured by the proportion of patients with best overall confirmed response of complete response (CR) or partial response (PR) by Response Assessment in Neuro-Oncology (RANO) criteria following treatment with DAY101 in pediatric patients aged 6 months to 25 years, inclusive, with a relapsed or progressive low-grade glioma harboring a known activating v-raf murine sarcoma viral oncogene homolog B (BRAF) alteration.

Secondary Objectives:

- To assess the safety and tolerability of DAY101
- To determine the relationship between pharmacokinetics (PK) and drug effects, including efficacy and safety
- To evaluate the effect of DAY101 on the QT interval corrected for heart rate by Fridericia's formula (QTcF) prolongation and to explore the effects of DAY101 on electrocardiogram (ECG) parameters
- To determine the ORR based on the treating investigator's response assessment using RANO criteria
- To determine the ORR based on Response Assessment in Pediatric Neuro-Oncology (RAPNO)–low-grade glioma criteria as determined by an IRC
- To evaluate the duration of progression-free survival (PFS) based on RANO and RAPNO criteria following initiation of DAY101 as determined by 1) an IRC and 2) the treating investigator (RANO only)
- To evaluate the duration of response (DOR) in patients with best overall response (BOR) of CR or PR based on RANO and RAPNO criteria as determined by 1) an IRC and 2) the treating investigator (RANO only)
- To evaluate time to response (TTR) (CR or PR based on RANO and RAPNO criteria) following initiation of DAY101 as determined by 1) an IRC and 2) the treating investigator (RANO only)
- To evaluate the clinical benefit rate based on the proportion of patients with BOR of CR, PR, or stable disease (SD), based on RANO and RAPNO criteria, lasting 12 months or more following initiation of DAY101, as determined by 1) an IRC and 2) the treating investigator (RANO only)
- To evaluate changes in best corrected visual acuity (BCVA) outcomes
- To evaluate the concordance of prior local laboratory BRAF molecular profiling with a central BRAF alteration assay being evaluated by the Sponsor

Exploratory Objectives:

- To compare the response and time to progression following initiation of DAY101 to that of the prior line of systemic therapy
- To characterize changes in total tumor volume following treatment with DAY101 by magnetic resonance imaging (MRI) volumetric image analysis
- To characterize changes in apparent diffusion coefficients following treatment with DAY101 using diffusion-weighted imaging analysis
- To evaluate changes in quality of life and health utilities measures using the Pediatrics Quality of Life™—Core (PedsQL-Core), Pediatrics Quality of Life™—Cancer (PedsQL-Cancer), and Patient-Reported Outcomes Measurement Information System (PROMIS®) assessment
- To describe improvement in motor function
- To determine the durability of response following discontinuation of DAY101 for patients with a radiographic response to DAY101 (CR or PR as based on RANO and RAPNO criteria) as determined by 1) an IRC and 2) the treating investigator (RANO only)
- To evaluate time to initiation of next treatment following discontinuation of DAY101

ARM 2 (LOW-GRADE GLIOMA EXTENSION) OBJECTIVES

Primary Objective:

The primary objective of Arm 2 is to assess the safety and tolerability of DAY101 in pediatric patients aged 6 months to 25 years, inclusive, with a relapsed or progressive low-grade glioma harboring a known or expected to be activating RAF alteration.

Secondary Objectives:

- To determine the ORR based on RANO criteria as determined by 1) an IRC and 2) the treating investigator
- To determine the ORR based on RAPNO–low-grade glioma criteria as determined by an IRC
- To evaluate the duration of PFS based on RANO and RAPNO criteria following initiation of DAY101 as determined by 1) an IRC and 2) the treating investigator (RANO only)
- To evaluate the DOR in patients with BOR of CR or PR based on RANO and RAPNO criteria as determined by 1) an IRC and 2) the treating investigator (RANO only)
- To evaluate TTR (CR or PR based on RANO and RAPNO criteria) following initiation of DAY101 as determined by 1) an IRC and 2) the treating investigator (RANO only)
- To evaluate the clinical benefit rate based on the proportion of patients with BOR of CR, PR, or SD, based on RANO and RAPNO criteria, lasting 12 months or more following initiation of DAY101, as determined by 1) an IRC and 2) the treating investigator (RANO only)
- To determine the relationship between PK and drug effects, including efficacy and safety
- To evaluate the effect of DAY101 on QTcF prolongation and to explore the effects of DAY101 on ECG parameters

Exploratory Objectives:

- To characterize changes in total tumor volume following treatment with DAY101 by MRI volumetric image analysis
- To characterize changes in apparent diffusion coefficients following treatment with DAY101 using diffusion-weighted imaging analysis
- To evaluate changes in BCVA outcomes
- To evaluate changes in quality-of-life and health utilities measures using the PedsQL-Core, PedsQL-Cancer, and PROMIS® assessment
- To describe the improvement in motor function
- To determine the durability of response following discontinuation of DAY101 for patients with a radiographic response to DAY101 (CR or PR as based on RANO and RAPNO criteria) as determined by 1) an IRC and 2) the treating investigator (RANO only)
- To evaluate the concordance of prior local laboratory BRAF molecular profiling with a central BRAF alteration assay being evaluated by the Sponsor

ARM 3 (ADVANCED SOLID TUMOR) OBJECTIVES

Primary Objective:

The primary objective of Arm 3 is to evaluate the preliminary efficacy of DAY101 as measured by the ORR as determined by an IRC following treatment with DAY101 in pediatric patients aged 6 months to 25 years, inclusive, with a relapsed or progressive advanced solid tumor harboring a known or expected to be activating RAF fusion.

Secondary Objectives:

- To assess the safety and tolerability of DAY101 in pediatric patients with advanced solid tumors
- To determine the relationship between PK and drug effects, including efficacy and safety
- To evaluate the effect of DAY101 on QTcF prolongation and to explore the effects of DAY101 on ECG parameters
- To determine the ORR based on the treating investigator's response assessment using RECIST v1.1 criteria
- To evaluate the duration of PFS based on RECIST v1.1 criteria following initiation of DAY101 as determined by 1) an IRC and 2) the treating investigator
- To evaluate the DOR in patients with BOR of CR or PR based on RECIST v1.1 criteria as determined by 1) an IRC and 2) the treating investigator
- To evaluate TTR (CR or PR based on RECIST v1.1 criteria) following initiation of DAY101 as determined by 1) an IRC and 2) the treating investigator
- To evaluate the clinical benefit rate based on the proportion of patients with BOR of CR, PR, or SD, based on RECIST v1.1 criteria, lasting 12 months or more following initiation of DAY101 as determined by 1) an IRC and 2) the treating investigator
- To evaluate the concordance of prior local laboratory RAF molecular profiling with a central RAF alteration assay being evaluated by the Sponsor

Exploratory Objectives:

- To evaluate changes in quality-of-life and health utilities measures using the PedsQL-Core, PedsQL-Cancer, and PROMIS® assessment
- To determine the durability of response following discontinuation of DAY101 for patients with a radiographic response to DAY101 (CR or PR as based on RECIST v1.1 criteria) as determined by 1) an IRC and 2) the treating investigator

STUDY DESIGN:

This is a Phase 2, multicenter, multi-arm, open-label study evaluating DAY101 in pediatric patients with low-grade gliomas and advanced solid tumors. The study will consist of the following treatment arms:

- **Arm 1 (Low-Grade Glioma)**: Patients aged 6 months to 25 years, inclusive, with recurrent or progressive low-grade glioma harboring a known activating BRAF alteration, including BRAF V600 mutations and KIAA1549:BRAF fusions. Patients with BRAF alterations will be identified through molecular assays as routinely performed at Clinical Laboratory Improvement Amendments (CLIA) of 1988 or other similarly certified laboratories.
- **Arm 2 (Low-Grade Glioma Extension)**: Patients aged 6 months to 25 years, inclusive, with recurrent or progressive low-grade glioma harboring a known or expected to be activating RAF alteration (e.g., BRAF or CRAF/RAF1 fusion or BRAF V600 mutations). Patients with RAF alterations will be identified through molecular assays as routinely performed at CLIA-certified or other similarly certified laboratories. Opening of Arm 2 to enrollment will be based on the recommendation of the Data Safety Monitoring Board (DSMB) as described in the protocol.
- **Arm 3 (Advanced Solid Tumor)**: Patients aged 6 months to 25 years, inclusive, with advanced solid tumors harboring a known or expected to be activating RAF fusion (e.g., BRAF or CRAF/RAF1 fusion). Patients with RAF fusions will be identified through molecular assays as routinely performed at CLIA-certified or other similarly certified laboratories.

For all arms, treatment cycles will repeat every 28 days in the absence of disease progression or unacceptable toxicity. Patients will continue on DAY101 until radiographic evidence of disease progression as determined by treating investigator, unacceptable toxicity, decision to enter a “drug holiday” period, patient withdrawal of consent, or death.

Patients will be treated with DAY101 for a planned period of 26 cycles (approximately 24 months), after which they may continue on DAY101 or, at any point, opt to enter a “drug holiday” discontinuation period. Patients will continue to undergo routine periodic radiographic evaluations per protocol-defined timelines during the discontinuation period and may be re-treated with DAY101 if there is radiographic evidence of disease progression after drug discontinuation.

Patients who have radiographic evidence of disease progression may be allowed to continue DAY101 if, in the opinion of the investigator, and approved by the Sponsor, the patient is deriving clinical benefit from continuing study treatment. Patients being treated beyond progression with DAY101 will be reconsented prior to continuation of therapy. Disease assessments for patients being treated beyond progression should continue as per regular schedule.

The study will consist of a screening period, a treatment period, an end of treatment (EOT) visit, a safety follow-up visit, and long-term follow-up assessments. Ongoing safety, disease stability/progression, survival, and subsequent anticancer therapies will be assessed in the long-term follow-up period.

DAY101 will be administered at the recommended Phase 2 dose (RP2D) of 420 mg/m^2 (not to exceed 600 mg), orally (PO) once weekly (QW) (Days 1, 8, 15, and 22 of a 28-day cycle). Body surface area (BSA) will be determined by the Mösteller Formula [$\sqrt{}$(height × weight)/3600)]. DAY101 is administered as an oral tablet or reconstituted liquid suspension formulation.

MAJOR ELIGIBILITY CRITERIA:

INCLUSION CRITERIA

Patients must meet all of the following criteria to be eligible for enrollment in the study:

1. Patients must be age 6 months to 25 years, inclusive, with:
    a) Arm 1 (Low-Grade Glioma): A relapsed or progressive low-grade glioma with a documented known activating BRAF alteration, as identified through molecular assays as routinely performed at CLIA or other similarly certified laboratories
    b) Arm 2 (Low-Grade Glioma Extension): A relapsed or progressive low-grade glioma with a documented known or expected to be activating BRAF mutation or RAF fusion, as identified

through molecular assays as routinely performed at CLIA-certified or other similarly certified laboratories

c) Arm 3 (Advanced Solid Tumor): Locally advanced or metastatic solid tumor with a documented known or expected to be activating RAF fusion, as identified through molecular assays as routinely performed at CLIA-certified or other similarly certified laboratories, that has relapsed or progressed or was nonresponsive to available therapies and for which no standard or available systemic curative therapy exists

2. Patients must have histopathologic verification of malignancy at either original diagnosis or relapse.
3. Patients must have received at least one line of prior systemic therapy and have documented evidence of radiographic progression.
4. Patients must have evaluable and/or measurable disease (imaging must be performed within 28 days of the initiation of treatment) as specified below:
    a) Arm 1 (Low-Grade Glioma): Must have at least one measurable lesion as defined by RANO criteria (T1-weighted lesion that can be reproducibly measured in at least 2 dimensions of at least 10 mm, visible on 2 or more axial slices that are preferably, at most, 5 mm apart with 0-mm skip)
    b) Arm 2 (Low-Grade Glioma Extension): Must have evaluable (either unidimensionally measurable lesions, masses with margins not clearly defined, or lesions with maximal perpendicular diameters less than 10 mm) and/or measurable disease as defined by RANO criteria
    c) Arm 3 (Advanced Solid Tumor): Must have at least one measurable lesion as defined by RECIST v1.1 (≥ 10 mm by CT/MRI scan [slice thickness ≤ 5 mm], ≥ 20 mm by chest X-ray, or ≥ 10 mm with calipers by clinical exam; or pathologic lymph nodes with a short axis of ≥ 15 mm by CT scan/MRI)
5. Radiation therapy to the measurable lesion(s) must be completed at least six months prior to administration of DAY101. Patients who have documented radiographic progression less than six months from radiotherapy in one or more measurable lesions are eligible.
6. Patients must have Karnofsky (those 16 years and older) or Lansky (those younger than 16 years) performance score of at least 50. Patients who are unable to walk because of paralysis, but who are able to sit in a wheelchair, will be considered ambulatory for the purpose of assessing the performance score.
7. Patients must have fully recovered from the acute toxic effects of all prior anticancer chemotherapy and have undergone the following washout periods, as applicable:
    a) Myelosuppressive chemotherapy: At least 21 days must have elapsed after the last dose of myelosuppressive chemotherapy (42 days if prior nitrosourea).
    b) Radiation therapy (XRT): At least 14 days must have elapsed after the last dose fraction of XRT.
    c) Stem cell transplant or adoptive cell therapy: At least 100 days must have elapsed after cell infusion.
    d) Investigational agent or any other anticancer therapy not defined above: At least four weeks prior to planned start of DAY101 or five half-lives, whichever is shorter.
8. Chronic toxicities from prior anticancer therapy must be stable and at Common Terminology Criteria for Adverse Events (CTCAE) version 5.0 Grade ≤ 2.
    a) Ongoing retinopathy must be ≤ Grade 1.
9. Patients must have fully recovered from any prior surgery.
10. An archival tumor tissue sample must be available. If an archival tumor tissue sample is not available, a fresh biopsy should be performed at baseline. For patients enrolling to Arm 2 (Low-Grade Glioma Extension) that do not have archival tumor tissue, enrollment may be considered on a case-by-case basis following a discussion between the investigator and the Day One Medical Monitor.
11. Patients must have adequate hematologic function, as defined by the following:
    a) Absolute neutrophil count ≥ 1000/mm^3

b) Platelet count ≥ 75.0 × 10^9/L (transfusions allowed per institutional guidelines; last transfusion > 2 weeks prior to C1D1)

c) Hemoglobin ≥ 10.0 g/dL (transfusions allowed per institutional guidelines; last transfusion > 4 weeks prior to C1D1)

d) Hematopoietic growth factors: At least 14 days after the last dose of a long-acting growth factor (e.g., Neulasta®) or seven days for short-acting growth factor

12. Patients must have adequate hepatic and renal function, defined as the following:

    a) Total bilirubin ≤ 1.5 × upper limit of normal (ULN) for age (patients with documented Gilbert's Disease may be enrolled with Sponsor approval and total bilirubin ≤ 2.0 × ULN)

    b) Serum glutamic-pyruvic transaminase (SGPT)/alanine aminotransferase (ALT) ≤ 2.5 × ULN

    c) Serum glutamic-oxaloacetic transaminase (SGOT)/aspartate transaminase (AST) ≤ 2.5 × ULN

    d) Serum creatinine within normal limits, or estimated glomerular filtration rate ≥ 60 mL/min/1.73 m^2 based on local institutional practice for determination

13. Thyroid function tests must be consistent with stable thyroid function. Patients on a stable dose of thyroid replacement therapy for a minimum of three weeks before starting DAY101 are eligible.
14. Patients must have left ventricular ejection fraction (LVEF) of ≥ 50% as measured by echocardiogram (ECHO) or multiple-gated acquisition (MUGA) scan, or fractional shortening (FS) ≥ 25% as measured by ECHO, within 28 days before the first dose of DAY101. If normal practice at the institution is to provide the LVEF result as a range of values, then the upper value of the range will be used to determine the result.
15. Patients receiving steroids for tumor-associated symptoms must be on a stable dose (e.g., no initial/loading dose, no increase or decrease) for 14 days prior to C1D1.
16. Patients must be able to comply with treatment, laboratory monitoring, and required clinic visits for the duration of study participation.
17. Male and female patients with reproductive potential must be willing to use a highly effective birth control method for the duration of treatment and for 180 days following the last dose of study drug.
18. Patients must have ability to swallow tablets or liquid, or be willing to comply with administration of a nasal or gastric tube for gastric access.
19. Parent/guardian of child or adolescent patient must have the ability to understand, agree to, and sign the study informed consent form (ICF) and applicable pediatric assent form before initiation of any protocol-related procedures; patient must have the ability to give assent, as applicable, at the time of parental/guardian consent.

EXCLUSION CRITERIA

Patients meeting any of the following criteria are to be excluded from study participation:

1. Patient's tumor has additional previously known or expected to be activating molecular alteration(s) (e.g., histone mutation, IDH1/2 mutations, FGFR mutations or fusions, MYBL alterations, NF1 somatic or germline mutations).
2. Patient has symptoms of clinical progression without radiographically recurrent or radiographically progressive disease.
3. Patient has known or suspected diagnosis of neurofibromatosis type 1 (NF-1) via genetic testing or current diagnostic criteria.
4. Patient has history of any major disease, other than the primary malignancy under study, that might interfere with safe protocol participation.
5. Patient has a history or current evidence of central serous retinopathy (CSR), retinal vein occlusion (RVO), or ophthalmopathy present at baseline that would be considered a risk factor for CSR or RVO. Ophthalmological findings secondary to long-standing optic pathway glioma (such as visual loss, optic nerve pallor, or strabismus) will NOT be considered a significant abnormality for the purposes of this study.

6. Patient has major surgery within 14 days (two weeks) prior to C1D1 (does not include central venous access, cyst fenestration or cyst drainage, or ventriculoperitoneal shunt placement or revision).
7. Patient has clinically significant active cardiovascular disease, or history of myocardial infarction, or deep vein thrombosis/pulmonary embolism within six months prior to C1D1, ongoing cardiomyopathy, or current prolonged QT interval corrected for heart rate by Fridericia's formula (QTcF) interval > 470 ms based on triplicate ECG average.
8. Patient is currently enrolled in any other investigational treatment study. Participation in a concurrent observational or bio-sampling study is allowed.
9. Patient has active systemic bacterial, viral, or fungal infection.
10. Patient has nausea and vomiting ≥ National Cancer Institute (NCI) Common Terminology Criteria for Adverse Events (CTCAE) v5.0 Grade 2, malabsorption requiring supplementation, or significant bowel or stomach resection that would preclude adequate absorption of DAY101.
11. Patient is neurologically unstable despite adequate treatment (e.g., uncontrolled seizures).
12. Patient is currently being treated with a strong cytochrome P450 2C8 (CYP2C8) inhibitor or inducer other than those allowed per protocol. Medications that are substrates of CYP2C8 are allowed but should be used with caution.
13. Patient is pregnant or lactating.
14. Patient has a history of any drug reaction with eosinophilia and systemic symptoms (DRESS) syndrome or Stevens Johnsons syndrome (SJS), or hypersensitivity to the investigational medicinal product or to any drug with similar chemical structure or to any other excipient present in the pharmaceutical form of the investigational medicinal product.
15. There are other unspecified reasons that, in the opinion of the investigator, make the patient unsuitable for enrollment.

PLANNED SAMPLE SIZE:

Approximately 140 patients in total will be enrolled across all treatment arms of this study. The arms will be enrolled as follows:

Arm 1 (Low-Grade Glioma): Approximately 60 patients

Arm 2 (Low-Grade Glioma Extension): Up to 60 patients

Arm 3 (Advanced Solid Tumor): Up to 20 patients

Patients will be considered enrolled when they have ingested a dose of DAY101 on C1D1.

INVESTIGATIONAL DRUG:

DAY101 for oral dosing is provided as an immediate-release tablet in one strength, 100 mg. The 100-mg tablets are red to yellowish red oval tablets and are labeled DAY101. In addition, DAY101 is provided in bottles as a powder for reconstitution (430 mg per bottle to deliver 300-mg dose) that is reconstituted with water to form a suspension with a concentration of 25 mg/mL.

TREATMENT PROCEDURES:

Patients will initiate treatment at the RP2D dose of 420 mg/m^2, PO QW (not to exceed 600 mg QW), on C1D1. DAY101 will be administered on Days 1, 8, 15, and 22 of a 28-day cycle.

Individual patients will continue DAY101 using QW dosing until radiographic evidence of disease progression by RANO criteria (Arms 1 and 2) or RECIST v1.1 criteria (Arm 3) as determined by treating investigator, unacceptable toxicity, decision to enter a "drug holiday" period after completion of 26 cycles, patient withdrawal of consent, or death. Patients who have radiographic evidence of disease progression may be allowed to continue DAY101 if, in the opinion of the investigator and approved by the Sponsor, the patient is deriving clinical benefit from continuing study treatment. Disease assessments for patients being treated beyond progression should continue as per regular schedule.

Patients may opt to enter a "drug holiday" discontinuation period at any point following 26 cycles (approximately two years) of therapy. Patients will continue to undergo routine periodic radiographic evaluations per protocol-defined timelines during the discontinuation period and may be re-treated with DAY101 if there is radiographic evidence of disease progression after drug discontinuation.

For patients requiring surgery, DAY101 should be held for six days prior to and after the surgical procedure.

All treated patients will undergo a safety follow-up visit at 30 days after the last dose. Patients who discontinue therapy early will be followed for long-term survival until end of study.

STUDY ASSESSMENTS:

Standard monitoring for safety is outlined in the protocol and will include physical examination and anthropometric measurements, Karnofsky/Lansky score, clinical adverse events (AEs), laboratory variables (e.g., hematology and serum chemistries), ECG, ECHO/MUGA, and vital signs.

Arms 1 and 2: Patients will undergo radiographic evaluation of their disease at the end of Cycle (C) 3 and at the end of every three cycles thereafter (i.e., on or up to seven days before Day [D] 1 of the next cycle).

Arm 3: Patients will undergo radiographic evaluation of their disease at the end of every second cycle, starting with the end of C2 (i.e., on or up to seven days before D1 of the next cycle), and at the end of every two cycles through 12 months of treatment. Thereafter, patients will undergo radiographic evaluation at the end of every three cycles.

Blood for PK assessments will be collected as follows:

- C1D1 at 1, 2, and 4 hours postdose
- C1D15 a sample between 1 and 4 hours postdose
- C2D1 within 1 hour predose
- C4D1 a sample between 1 and 4 hours postdose
- For D1 of every subsequent third cycle through 13 cycles (e.g., C4D1, C7D1, C10D1, and C13D1), a sample between 1 and 4 hours postdose
- At time of toxicity and/or time of surgery if clinically indicated
- At the time of a change in formulation (e.g., tablets to liquid, or vice versa), on Day 1 of the next cycle following the formulation change: predose, 1, 2, and 4 hours postdose

In patients 2 years of age or older, Health-Related Quality of Life will be assessed using the PedsQL-Core, PedsQL-Cancer, and PROMIS® assessment for the patient or their parent/caregiver every third cycle. The PROMIS® questionnaire will only be administered to English-speaking patients enrolled in the U.S., Australia, and U.K.

Central radiology review: All patients receiving protocol therapy will have their imaging scans centrally reviewed, in addition to review by a site or local radiologist. Copies of all eligibility, baseline, and on-treatment patient scans, as well as historical scans from the 12-month period immediately prior to the eligibility scan (if available), will be transferred to the central imaging laboratory as specified in the Imaging Manual.

During the course of treatment, at the EOT, or in the event of disease progression, should patients undergo a tumor-directed surgery, an optional fresh tumor specimen to evaluate tumor changes that may have resulted from treatment will be collected.

End of Study and Length of Study:

The end of this study is defined as the date when the last visit occurs for the last patient enrolled. The end of the study is expected to occur 25 months after the last patient is enrolled into Arm 1.

In addition, the Sponsor may decide to terminate the study at any time.

The total length of the study, from screening of the first patient to the end of the study, is expected to be approximately four years.

STATISTICAL METHODS:

Sample Size Considerations:

Arm 1 (Low-Grade Glioma): A sample size of 60 patients provides 88% power to reject the null ORR of 21%, assuming that the true underlying ORR of DAY101 is 40% based on a test at the 2-sided 0.05 level. A result of at least 20 out of 60 (0.33) will be statistically significant.

Arm 2 (Low-Grade Glioma Extension): Up to approximately 60 patients may be enrolled. Arm 2 is not powered based on formal statistical hypothesis testing, but for practical considerations to allow patients with pediatric low-grade glioma to receive treatment with DAY101 after Arm 1 has fully accrued and the DSMB has recommended opening to enrollment.

Arm 3 (Advanced Solid Tumor): Up to approximately 20 patients may be enrolled to provide a preliminary evaluation of DAY101 in pediatric patients with advanced solid tumors. For this portion of this study, a modified Simon 2-stage design will be used to enroll up to approximately 20 patients based on evaluation of ORR (confirmed PR or CR per RECIST v1.1) (Simon 1989). The statistical assumptions will require approximately 12 evaluable patients in the first stage and 20 evaluable patients in total. In the first stage, if ≤ 1 objective response (PR or CR per RECIST v1.1) is seen in the first 12 patients, the study will stop any further recruitment and the treatment will be considered as not effective in this setting; however, if ≥ 2 objective responses occur, an additional eight patients will be recruited. In patients with relapsed or progressive advanced solid tumors that have progressed after standard-of-care therapy and harbor an activating driver alteration, the anticipated response rate is expected to be near zero. However, for the purposes of sample size calculation, a null hypothesis (H_0) of 5% is assumed. A sample size of 20 patients provides 90% power to reject the null ORR of 5%, assuming that the true underlying ORR of DAY101 is 25% based on a test at alpha 1-sided at 0.1. A result of at least three out of 20 will be statistically significant.

Analysis Methods:

Efficacy Analyses

Arm 1 (Low-Grade Glioma): The efficacy analyses will include all patients who received at least one dose of study drug and have measurable disease as determined by the IRC at baseline and have follow-up imaging or radiographically confirmed progressive disease prior to the first imaging timepoint (Arm 1 Full Analysis Set [FAS]). Patients who do not meet this criterion (e.g., lost to follow-up with no follow-up imaging) will be replaced. Some efficacy analyses may be performed with one or more tumor types combined (e.g., subgroup of Arm 1 FAS consisting of patients with BRAF V600 mutation vs. BRAF fusion).

Overall response rate will be calculated by the number of patients with best overall confirmed response of CR or PR as determined by an IRC using the RANO criteria among the Arm 1 FAS. All responses will be confirmed by a second scan ≥ 28 days after the initial response. An exact binomial test will be used to compare the observed response rate to the hypothesized null ORR of 21%, and a 95% confidence interval (CI) will be calculated using the Clopper-Pearson method.

Arm 2 (Low-Grade Glioma Extension): Where appropriate (e.g., in patients with measurable disease), patients will be evaluated via the same statistical methods as described for Arm 1.

Arm 3 (Advanced Solid Tumor): The efficacy analyses will include all patients who received at least one dose of study drug, have measurable disease at baseline, and have follow-up imaging or radiographically confirmed progressive disease prior to the first imaging timepoint (Arm 3 FAS). Patients who do not meet this criterion (e.g., lost to follow-up with no follow-up imaging) will be replaced. These analyses may be summarized by tumor type, and some efficacy analyses may be performed with one or more tumor types combined.

The estimate of the ORR will be calculated by the number of patients with best overall confirmed response of CR or PR as determined by an IRC using RECIST v1.1 criteria among the Arm 3 FAS. All responses will be confirmed by a second scan ≥ 28 days after the initial response. The estimate of the ORR will be accompanied by 1- and 2-sided CIs with various coverage probabilities (e.g., 80%, 95%). The primary analysis of ORR will be based on the responses determined by an IRC. A secondary analysis will be based on the IRC's assessment.

Safety Analyses

A safety review committee (SRC) will be established to oversee the safety aspects of the study. The SRC membership will consist of the Sponsor's Medical Monitor for this study, a Clinical Operations representative, and a clinically qualified representative of the Principal Investigator from each active clinical site contributing patients to that cohort. The SRC will meet monthly until completion of enrollment, and then quarterly until study closure. In addition, an independent DSMB will be established and will meet approximately every three months to review safety and efficacy data across the entire DAY101 program. Any emergent safety issue identified by the SRC will be escalated to the DSMB.

Safety will be assessed by clinical review of all relevant parameters, including AEs, serious adverse events (SAEs), laboratory values, and vital signs. Unless specified otherwise, the safety analyses will be conducted for the safety population defined in the protocol.

Pharmacokinetic Analyses

Plasma concentrations of DAY101 will be determined with a validated bioanalytical assay. The following PK parameters may be calculated where appropriate: maximum drug concentration (C_{max}), minimum drug concentration (C_{min}), C_{ave}, time at which maximum observed concentration occurs ($T_{max,}$) area under the concentration-time curve from time zero to t ($AUC_{0\text{–}t}$), and apparent oral clearance of drug (CL/F). Summary statistics will be generated as appropriate.

Patient-Reported Outcomes

Changes in quality-of-life measures from baseline will be evaluated for all patients 2 years of age or older using the PedsQL-Core, PedsQL-Cancer, and PROMIS® assessment. The PROMIS® questionnaire will only be administered to English-speaking patients enrolled in the U.S., Australia, and U.K. Longitudinal changes in quality-of-life scores from baseline will be summarized in a descriptive manner.

TABLE OF CONTENTS

PROTOCOL AMENDMENT SUMMARY OF CHANGES 3
PROTOCOL SYNOPSIS 7
LIST OF ABBREVIATIONS AND DEFINITION OF TERMS 23
1. INTRODUCTION 26
1.1. Background on Disease 26
1.1.1. Cancer Physiology 26
1.1.2. Low-Grade Gliomas 27
1.1.2.1. Current Therapies 30
1.1.3. Pediatric Solid Tumors 33
1.1.3.1. Role of RAF Fusions in Pediatric Solid Tumors 33
1.2. Background on DAY101 34
1.2.1. DAY101 Nonclinical Data 34
1.2.2. DAY101 Clinical Data 35
1.2.2.1. Clinical Pharmacokinetics 36
1.2.2.2. Clinical Efficacy 36
1.2.2.3. Clinical Safety 37
1.3. Study Rationale and Dose Justification 39
1.3.1. Study Rationale 40
1.3.2. Justification for Dose 41
1.3.2.1. Potential Developmental Toxicities and Dosing Considerations 45
1.3.2.2. Drug Metabolism 45
1.3.3. Risk Management 46
1.3.4. Required Study Procedures 46
1.3.5. Continuous Review of Safety Data 47
1.3.6. Potential Benefit for Patients in Clinical Trials and Patients in General 47
1.3.7. Integrated Benefit/Risk Assessment 47
2. STUDY OBJECTIVES 48
3. INVESTIGATIONAL PLAN 53
3.1. Study Design 53
3.1.1. Arm 1: Low-Grade Glioma 55
3.1.2. Arm 2: Low-Grade Glioma Extension 55

3.1.3. Arm 3: Advanced Solid Tumor 56
3.1.4. End of Study and Length of Study 56
3.1.5. Number of Patients 57
3.2. Investigational Sites 57
4. SELECTION OF STUDY POPULATION 57
4.1. Inclusion Criteria 57
4.2. Exclusion Criteria 59
5. TREATMENT 60
5.1. Investigational Product 60
5.2. DAY101 Administration 61
5.2.1. General Dosing Instructions 61
5.2.2. Dose Modifications 63
5.2.3. Management of Treatment-Related Events 64
5.2.3.1. Ocular Disturbances 65
5.2.3.2. Photosensitivity 65
5.2.3.3. Rashes 65
5.2.3.4. Increased Creatine Phosphokinase 65
5.2.3.5. Increased Liver Transaminases 66
5.3. Prior and Concomitant Medications 67
5.3.1. General 67
5.3.2. Allowed Concomitant Medications 67
5.3.3. Prohibited Concomitant Medications 68
5.3.4. Concurrent Surgery 68
5.3.5. Palliative Radiotherapy 69
5.4. Removal of Patients from Therapy or Assessment 69
6. TESTS AND EVALUATIONS 70
6.1. Screening 70
6.2. Enrollment 71
6.3. Procedure Details 71
6.4. Medical History and Malignancy History 71
6.5. Archival Tumor 72
6.6. Safety Assessments 72
6.6.1. Physical Examination 72

6.6.2. Karnofsky or Lansky Performance Status Scales 72
6.6.3. Vital Signs 72
6.6.4. Ophthalmology Examination 72
6.6.5. Dermatologic Examination 73
6.6.6. Neurologic Examination 73
6.6.7. Assessment of Change in Pubertal Development 74
6.6.8. Cardiac Function 74
6.6.8.1. Electrocardiograms 74
6.6.8.2. Echocardiogram/Multiple-Gated Acquisition 74
6.7. Laboratory Tests 74
6.7.1. Pregnancy Test 74
6.7.2. Hematology 75
6.7.3. Serum Chemistries 75
6.7.4. Thyroid Function Tests 75
6.8. Pharmacokinetic Samples: Blood 75
6.9. Disease Assessment 76
6.9.1. Tumor Measurements 76
6.9.1.1. Collection of Radiographic Studies for Independent Review 76
6.9.1.2. Visual Acuity Assessments 76
6.9.1.3. Motor Function Evaluation Using the Vineland Motor Scale 77
6.9.2. Quality of Life: Pediatric Quality of Life Inventory—Core, Cancer, and Patient-Reported Outcomes Measurement Information System 77
6.10. Telephone/Telemedicine Visit 78
6.11. Survival Status 78
6.12. Study Drug Administration and Dosing Diary 78
6.13. Concomitant Medication 79
6.14. End of Treatment Visit 79
6.15. Safety Follow-Up Visit 79
6.16. Long-Term Follow-Up 79
6.17. Force Majeure Flexibility 79
7. PLANNED ANALYSES 80
7.1. Analysis Populations 80
7.2. Determination of Sample Size 81

7.3. Statistical Methods 82
7.3.1. Efficacy Analyses 82
7.3.1.1. Arm 1: Low-Grade Glioma 82
7.3.1.2. Arm 2: Low-Grade Glioma Extension 83
7.3.1.3. Arm 3: Advanced Solid Tumor 83
7.3.2. Safety Analyses 84
7.3.2.1. Deaths 85
7.3.2.2. Laboratory Values 85
7.3.2.3. Vital Signs 85
7.3.2.4. Concentration QTc (cQTc) Analysis 85
7.3.2.5. Concomitant Medications 85
7.3.2.6. Pharmacokinetic Analyses 85
7.3.3. Health-Related Quality of Life 86
7.3.4. Visual Acuity Outcomes 86
7.3.5. Motor Function (Vineland) Outcomes 86
8. SAFETY 86
8.1. Adverse Events and Serious Adverse Events 86
8.1.1. Time Period and Frequency for Collecting Adverse Event and Serious Adverse Event Information 86
8.1.2. Method of Detecting Adverse Events and Serious Adverse Events 87
8.1.3. Follow-Up of Adverse Events and Serious Adverse Events 87
8.1.4. Regulatory Reporting Requirements for Serious Adverse Events 87
8.1.5. Pregnancy 88
8.1.6. Deaths 88
8.2. Safety Review Committee and Data Safety Monitoring Board 89
8.3. Stopping Rules 89
8.4. Criteria for Pausing or Stopping the Study 89
8.5. Grading and Intensity of Adverse Events 90
8.6. Relationship to Study Drug 90
8.7. Serious Adverse Event Follow-Up 91
9. STUDY ADMINISTRATION 91
9.1. Regulatory and Ethical Considerations 91
9.1.1. Regulatory Authority Approval 91

9.1.2. Ethics Approval 91
9.1.3. Patient Informed Consent and Pediatric Assent 92
9.1.4. Investigator Reporting Requirements 93
9.2. Data Management 93
9.3. Study Monitoring 93
9.4. Termination 94
9.5. Records Retention 94
9.6. Confidentiality of Information 94
10. REFERENCES 95

LIST OF TABLES

Table 1: Histological Subgroups of Low-Grade Gliomas Demonstrating in Which Tumors RAF Alterations Have Been Identified 30
[redacted]
Table 4: Arm 1 (Low-Grade Glioma) Objectives and Endpoints 48
Table 5: Arm 2 (Low-Grade Glioma Extension) Objectives and Endpoints 50
Table 6: Arm 3 (Advanced Solid Tumor) Objectives and Endpoints 52
Table 7: Dose Rounding Guidelines for DAY101 Tablets (100 mg) 62
Table 8: Dose and Volume Rounding Guidelines for DAY101 Reconstituted Liquid Suspension (25 mg/mL) 63
Table 9: Treatment-Emergent Adverse Events Requiring Dose Interruption Until Resolution to ≤ Grade 1 or Baseline 64
Table 10: Increased Creatine Phosphokinase Recommendations 66
Table 11: Increased Liver Transaminases Recommendations 67
Table 12: Schedule of Blood Sample Collections for DAY101 Pharmacokinetics 75
Table 13: Population Definitions 81
Table 14: Summary of Adverse Event Collection Periods 87
Table 15: NCI CTCAE Definitions of Severity for Adverse Reactions 90
Table 16: Arms 1 and 2 Schedule of Assessments 101
Table 17: Arm 3 Schedule of Assessments 107
Table 18: Karnofsky Performance Score (≥ 16 years old) 113
Table 19: Lansky Performance Score (< 16 years old) 113

Table 20: List of Medications with Possible DAY101 CYP Interactions 114
Table 21: List of Medications with Possible DAY101 BCRP Interactions.......... 114
Table 22: RANO Response Criteria Incorporating MRI and Clinical Factor 116
Table 23: Overall Assessment by RECIST v1.1 at Each Time Point.................. 121
Table 24: Best Overall Assessment by RECIST v1.1... 122

LIST OF FIGURES

Figure 1: Mechanism of Fusion Oncogene Action 27
Figure 2: Pediatric Low-Grade Gliomas: Subtypes, Prognosis, and BRAF Alterations/Frequency.. 29
Figure 3: Mean ± SD DAY101 Plasma Concentration Time Profiles Following Oral Administration of DAY101 as Three Different Formulations in Differing Prandial States (100-mg Dose) 44
Figure 4: Mean ± SD DAY101 Plasma Concentration Time Profiles Following Oral Administration of DAY101 as Three Different Formulations in Differing Prandial States (300-mg Dose) 44
Figure 5: Study Design Schema 54
Figure 6: Treatment Schema 55

LIST OF APPENDICES

Appendix A: Study Assessments 101
Appendix B: Karnofsky and Lansky Performance Status Scales 113
Appendix C: List of Medications with Possible DAY101 CYP and BCRP Interactions 114
Appendix D: Response Assessment in Neuro-Oncology (RANO) Criteria for Primary CNS Malignancies 116
Appendix E: Response Evaluation Criteria in Solid Tumors: Revised RECIST Guideline (version 1.1) 118
Appendix F: Specimen and Data Use 123
Appendix G: Blood Pressure by Height and Age 124
Appendix H: Rash Management 127
Appendix I: Visual Acuity Testing 129
Appendix J: Adverse Events. Definitions and Procedures for Recording, Evaluating, Follow-Up, and Reporting.. 131
Appendix K: Contraceptive Guidance and Collection of Pregnancy Information 136

LIST OF ABBREVIATIONS AND DEFINITION OF TERMS

Abbreviation or Term	Definition
AE	adverse event
ALT	alanine aminotransferase
ASCO	American Society for Clinical Oncology
AST	aspartate aminotransferase
AUC	area under the concentration-time curve
AUC_{0-t}	area under the concentration-time curve from time zero to t
$AUC_{0-\infty}$	area under the concentration-time curve from time zero to infinity
AUC_{last}	area under the concentration-time curve from time zero to the last timepoint
BCRP	breast cancer resistance protein
BCVA	best corrected visual acuity
BOR	best overall response
BP	blood pressure
BRAF	v-raf murine sarcoma viral oncogene homolog B
BSA	body surface area
C	Cycle
CI	confidence interval
CL/F	apparent oral clearance of drug
CLIA	Clinical Laboratory Improvement Amendments
C_{max}	maximum drug concentration
C_{min}	minimum drug concentration
CNS	central nervous system
CPK	creatine phosphokinase
CR	complete response
CRF	case report form
CSR	central serous retinopathy
CTCAE	Common Terminology Criteria for Adverse Events
CYP	cytochrome P450
CYP2C8	cytochrome P450 2C8
CYP3A4	cytochrome P450 3A4
D	Day
DLT	dose-limiting toxicity
DOR	duration of response
DRESS	drug reaction with eosinophilia and systemic symptoms
DSMB	Data Safety Monitoring Board
ECG	electrocardiogram
eCRF	electronic case report form
ECHO	echocardiogram

Abbreviation or Term	Definition
EDC	electronic data capture
EMA	European Medicines Agency
EOT	end of treatment
ERK	extracellular signal-related kinase
ETDRS	Early Treatment Diabetic Retinopathy Study
FAS	Full Analysis Set
FFPE	formalin-fixed, paraffin-embedded
GCP	Good Clinical Practices
H_0	null hypothesis
HREC	Human Research Ethics Board
IB	Investigator's Brochure
ICF	informed consent form
ICH	International Council for Harmonisation
IEC	Independent Ethics Committee
IFS	infantile fibrosarcoma
IRB	Institutional Review Board
IRC	independent radiology review committee
IRT	interactive response technology
ITT	intent to treat
LVEF	left ventricular ejection fraction
MAPK	mitogen-activated protein kinase
MedDRA	Medical Dictionary for Regulatory Activities
MEK	mitogen-activated protein kinase
MRI	magnetic resonance imaging
MTD	maximum tolerated dose
MUGA	Multiple-gated acquisition
NCI	National Cancer Institute
NE	not evaluable
NTRK genes	neurotrophic tyrosine kinase receptor genes
OPG	optic pathway glioma
ORR	overall response rate
OS	overall survival
PD	progressive disease
PedsQL-Core	Pediatrics Quality of Life™—Core
PedsQL-Cancer	Pediatrics Quality of Life™—Cancer
pERK	phosphorylated ERK
PFS	progression-free survival
PK	pharmacokinetic(s)

Abbreviation or Term	Definition
PO	*per os*, orally
PR	partial response
PROMIS®	Patient-Reported Outcomes Measurement Information System
PXA	xanthoastrocytoma
Q2D	every other day
QOL	quality of life
QT_c	QT interval corrected for heart rate
QT_cF	QT interval corrected for heart rate by Fridericia's formula
ΔQTcF	change in QT interval corrected for heart rate by Fridericia's formula
QW	once weekly
RANO	Response Assessment in Neuro-Oncology
RAPNO	Response Assessment in Pediatric Neuro-Oncology
REB	Research Ethics Board
RP2D	recommended Phase 2 dose
RVO	retinal vein occlusion
SAE	serious adverse event
SCC	squamous cell carcinoma
SD	stable disease
SGOT	serum glutamic-oxaloacetic transaminase
SGPT	serum glutamic-pyruvic transaminase
SJS	Stevens Johnsons syndrome
SRC	Safety Review Committee
T3	triiodothyronine
T4	tetraiodothyronine
$t_{1/2}$	half-life
TAC	Teller Acuity Cards®
TEAE	treatment-emergent adverse event
T_{max}	time at which maximum observed concentration occurs
TNM	tumor, node, metastasis
TPCV	thioguanine, procarbazine, lomustine, and vincristine
TSH	thyroid stimulating hormone
TTR	time to response
ULN	upper limit of normal
VA	visual acuity
WHO	World Health Organization
XRT	radiation therapy

1. INTRODUCTION

DAY101 is an oral, selective, small-molecule, Type II pan-RAF kinase inhibitor that is being developed by DOT Therapeutics-1 Inc. (Day One, the Sponsor) for the treatment of recurrent or progressive pediatric low-grade gliomas and pediatric and adult solid tumors which harbor a known or expected to be activating RAF alteration.

1.1. Background on Disease

1.1.1. Cancer Physiology

The mitogen-activated protein kinase (MAPK) signaling pathway is an essential pathway that regulates key cell functions, such as growth, survival, and differentiation. Genomic alterations and dysregulation of the MAPK pathway have been described in many different types of malignancies, including pediatric low-grade glioma (Yaeger and Corcoran 2019). One of the most frequently altered genes in this pathway is v-raf murine sarcoma viral oncogene homolog B (BRAF), in which 90% of the mutations consist of the BRAF V600E point mutation. This point mutation leads to both increased BRAF kinase activity that is independent of extracellular stimuli or RAS-GTP, and constitutive signaling that drives cell proliferation and tumor growth (Davies et al. 2002). Currently, the US Food and Drug Administration (FDA) has approved three agents that inhibit BRAF-V600E/K mutations: vemurafenib, dabrafenib, and encorafenib.

Beyond point mutations, BRAF fusions and in-frame deletions have also been reported in human cancers (Ross et al. 2016). BRAF fusions are rearrangements in which the N-terminal inhibitory domain of BRAF is replaced with a different sequence that enables BRAF to signal as a dimer independent of RAS activation. This drives aberrant expression and constitutive activation of the kinase and downstream signaling pathways. Fusions involving oncogenes have historically been targetable by kinase inhibitors, and multiple targeted agents that have demonstrated clinical responses to cancer patients harboring fusions involving ABL, ALK, TRK, RET, and ROS1 have been approved by the US FDA and European Medicines Agency (EMA).

In 2008, several research groups published critical discoveries from tumor specimens obtained from patients with pediatric low-grade glioma that identified a fusion involving two genes, KIAA1549 and BRAF, that led to constitutive activation of BRAF kinase activity, as well as increased expression of the fusion (see Figure 1). The identification of this KIAA1549:BRAF fusion led to deeper understanding of the genomic events driving growth of pediatric low-grade glioma (Jones et al. 2008; Penman et al. 2015). The KIAA1549:BRAF fusion has been found to drive the majority of pediatric low-grade gliomas, and exhibits kinase activity and constitutive downstream signaling comparable to the canonical BRAF V600E point mutation (Jones et al. 2008). The KIAA1549:BRAF fusion is found in 30% to 40% of pediatric low-grade gliomas at the population level, and 70% to 80% of pilocytic astrocytomas (Ryall et al. 2020); the BRAF V600E point mutation is less common in

pediatric low-grade glioma, occurring in approximately 15% of pediatric low-grade gliomas, and is associated with worse clinical outcomes and worse response to chemotherapy (Nicolaides et al. 2020).

Figure 1: Mechanism of Fusion Oncogene Action

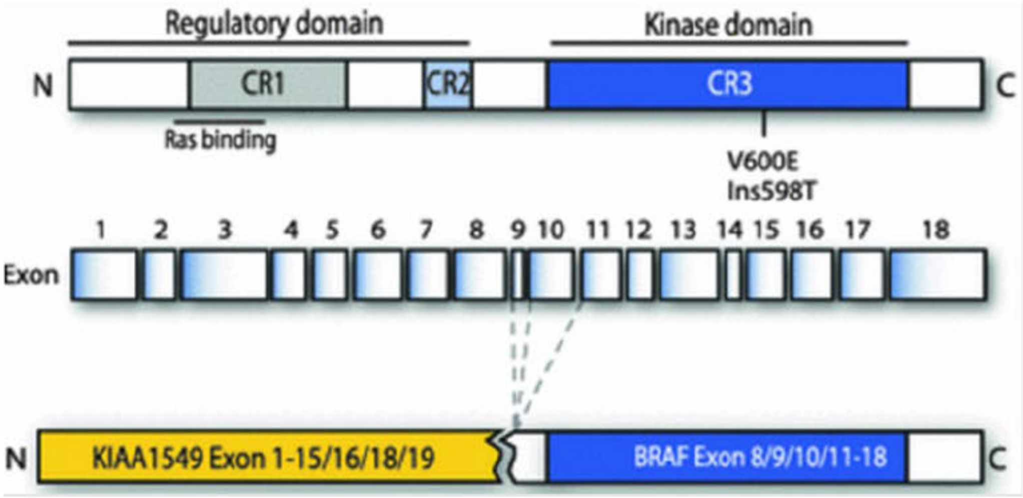

Abbreviations: BRAF, v-raf murine sarcoma viral oncogene homolog B.
Source: Jones et al. 2012.

1.1.2. Low-Grade Gliomas

As noted above, pediatric low-grade gliomas are the most common brain tumor diagnosed in children (Ostrom et al. 2019). They can occur anywhere within the central nervous system (CNS), including the cerebellum (15%–25%), cerebral hemisphere (10%–15%), optic pathway (5%), spinal cord (5%), and brain stem (2%–4%). Histopathologic classification is based on which normal cell is most closely resembled (astrocytomas, oligodendrogliomas, mixed glial-neuronal). Grading is determined by the presence of necrosis, giant cells, mitosis, endothelial proliferation, hyperchromatic nuclei, and pleomorphic cells. The World Health Organization (WHO) classifies Grade I and Grade II tumors as low-grade gliomas. For the most part, although malignant transformation and dissemination of pediatric low-grade gliomas are rare (Waanders et al. 2016), these tumors are slow-growing, chronic, and relentless. As a result, these diseases are highly morbid due to both the growth and the burden of the tumor, as well as to the short- and long-term treatment-associated morbidity. In fact, the majority of children with pediatric low-grade gliomas are long-term survivors and live well into adulthood, requiring more effective treatment strategies that minimize long-term morbidity and treatment-associated toxicity (Bergthold et al. 2014).

Despite the low-grade histology, pediatric low-grade gliomas are a serious medical condition, with both the high-potential tumor- and treatment-associated morbidity and mortality and significant and life-long late effects. Proximity of the tumor to critical neurovascular structures can lead to motor function impairment, such as hemiparesis, significantly impacting daily life and development of the child, or visual impairment, leading to blindness in a relevant proportion of patients. In addition, hypothalamic pediatric low-grade gliomas

can result in multiple endocrine dysfunctions, particularly in the pituitary gland, significantly impacting growth and development of the child. In infants, a diencephalic syndrome causes severe emaciation with high risk of fatal outcome. Treatment of pediatric low-grade gliomas with chemotherapy can result in both acute and chronic toxicities, depending on the treatment regimen and duration. This includes bone marrow toxicity in up to 100% of treated children, allergic reactions, impaired renal function, impaired hearing, and neurotoxicity. Radiation therapy can result in cerebrovascular disease and long-term neurocognitive deficits, particularly in younger patients, as well as increased risk of secondary malignancies. It is increasingly well established that patients, especially long-term survivors, have significant adverse outcomes related to the location and growth of the tumor (Sievert et al. 2009).

Cerebral hemisphere location, younger age at diagnosis, and hydrocephalus requiring a shunt have all been found to be significant predictors of lower cognitive performance on IQ scales. Children with infratentorial tumors can have significant language, cognitive, behavioral, and social dysfunctions. A combined analysis of Children's Cancer Group (CCG-9891) and Pediatric Oncology Group (POG-9130) data of 103 children with low-grade cerebellar astrocytomas treated with surgery alone revealed an elevated risk of cognitive and adaptive-behavioral impairment that was not associated with complications of the tumor or surgery (Beebe et al. 2005). Similar deficits were ubiquitous in children with extracerebellar tumors treated with surgery alone (Ris et al. 2008).

As noted above, multiple large-scale sequencing efforts of pediatric tumors have led to a better biological understanding of the molecular underpinnings of these tumors. The KIAA1549:BRAF fusion was found in the majority of tissue samples from patients with pilocytic astrocytomas (Jones et al. 2008). Over time, additional RAF genomic alterations, including the BRAF V600E mutation and both BRAF and CRAF/RAF1 fusions with novel partners, have been identified in patients with low-grade gliomas, cementing the idea of RAF as an oncogenic driver in low-grade glioma, just as an NTRK fusion is the key oncogenic event in patients with infantile fibrosarcoma (IFS) (Knezevich et al. 1998).

Pilocytic astrocytomas (Grade I) and diffuse fibrillary astrocytomas (Grade II) are the two most common pediatric low-grade gliomas. Pilocytic astrocytomas commonly occur in children up to 14 years of age. They can occur anywhere in the CNS, although they are typically located in the cerebellum, hypothalamus/optic nerve pathway, dorsally exophytic brain stem, or spinal cord. Conversely, diffuse fibrillary astrocytomas rarely occur before the age of 20 years and are located in the supratentorial region, deep midline structures, or cervicomedullary region of the brain stem (Waanders et al. 2016). Pilomyxoid astrocytomas (Grade II) are a subset of pilocytic tumors with a more aggressive clinical course. These occur in the hypothalamus in patients with a median age of 21 months. The Grade II tumor pleomorphic xanthoastrocytoma (PXA) is less aggressive than the diffuse fibrillary astrocytoma and more similar to the pilocytic astrocytoma. Gangliogliomas (Grade II), subependymal giant cell astrocytomas, and oligodendrogliomas are also included in the low-grade glioma classification (Waanders et al. 2016). The subtypes and prognosis of

pediatric low-grade gliomas and the nature and frequency of BRAF alterations associated with pediatric low-grade gliomas are summarized in Figure 2. The histological subgroups of low-grade gliomas, demonstrating in which tumors the BRAF fusion has been identified, are summarized in Table 1.

Figure 2: Pediatric Low-Grade Gliomas: Subtypes, Prognosis, and BRAF Alterations/Frequency

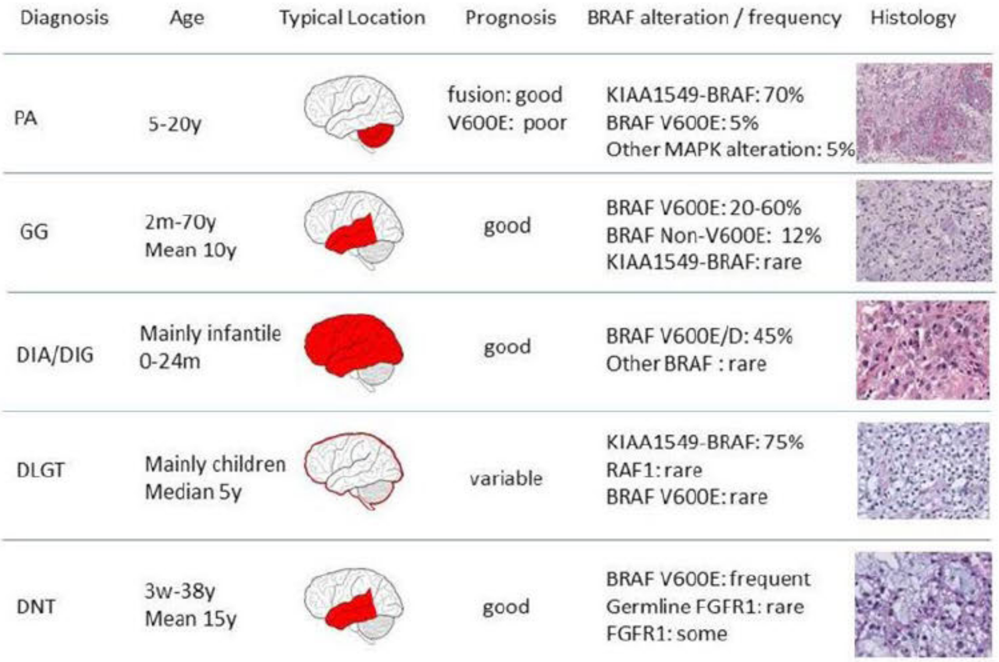

Abbreviations: BRAF, v-raf murine sarcoma viral oncogene homolog B; DIA/DIG, desmoplastic infantile astrocytoma/ganglioglioma; DLGT, diffuse leptomeningeal glioneuronal tumor; DNT, dysembryoplastic neuroepithelial tumor; FGFR1, fibroblast growth factor receptor 1; GG, ganglioglioma; MAPK, mitogen-activated protein kinase; PA, pilocytic astrocytoma; RAF1, Raf-1 proto-oncogene/serine/threonine kinase.

Adapted from (Behling and Schittenhelm 2019).

Table 1: Histological Subgroups of Low-Grade Gliomas Demonstrating in Which Tumors RAF Alterations Have Been Identified

Histological Subgroup	WHO Grade	KIAA1549: BRAF Fusion Described	Average % Fusion-Positive Patients	Other RAF Fusions	BRAF V600E Described	Average % BRAF V600E-Positive Patients
Pilocytic astrocytoma	I	Yes	77.2	FAM131B:BRAF<br>SRGAP3:RAF1<br>QK1:RAF1	Yes	6.2
Pilomyxoid astrocytoma	II	Yes	62.5		Yes	5.0
Diffuse fibrillary astrocytoma	II	Yes	3.0	FAM131B:BRAF	Yes	8.1
Pleomorphic xanthoastrocytoma	II	Yes	55.6	N/A	Yes	50.8
Dysembryoplastic neuroepithelial tumor	I	No	N/A	N/A	No	N/A
Desmoplastic infantile astrocytoma/glioma	II	No	N/A	FXR1:BRAF	Yes	8.5

Abbreviations: BRAF, v-raf murine sarcoma viral oncogene homolog B; FAM131B, family with sequence similarity 131 member B; FXR1, FMR1 autosomal homolog 1; N/A, not applicable; SRGAP3, SLIT-ROBO Rho GTPase activating protein 3; WHO, World Health Organization.

Adapted from Penman et al. 2015.

1.1.2.1. Current Therapies

The mainstays of treatment with pediatric low-grade gliomas have historically been surgery, radiation, and multiagent chemotherapy. While surgical resection of pediatric low-grade gliomas is associated with 10-year overall survival (OS) rates of 90% or more, the majority of children are unable to undergo complete resection. In addition, depending on the location of the tumor, surgical resection can be associated with significant and long-lasting morbidity. Incompletely resected or unresectable pediatric low-grade gliomas have a high rate of disease progression or recurrence. Patients with subtotal resections have a 10-year progression-free survival (PFS) of 55% (Tihan et al. 2012). Historically, focal radiation therapy was believed to lead to an improvement in 10-year PFS, with results in the 60% to 65% range. However, more recent studies have questioned the role of radiotherapy compared with chemotherapy or observation alone. Whole-brain radiotherapy has been shown to adversely affect IQ in survivors of pediatric brain tumors, as well as cause a number of other neurocognitive effects (Waanders et al. 2016), along with increased risk of cerebral vasculopathy and stroke.

Chemotherapy has been the mainstay of treatment options for patients with progressive low-grade glioma, with vincristine and carboplatin, single-agent vinblastine, or thioguanine,

procarbazine, lomustine, and vincristine (TPCV) being the most widely used combinations. The current standard-of-care front-line chemotherapeutic regimen (vincristine/carboplatin) has limited efficacy, and the long-term burden of both the disease- and treatment-related morbidity is significant. Results from the largest randomized Phase 3 study to date for children with newly diagnosed pediatric low-grade glioma showed a five-year event-free survival of only 47% for vincristine/carboplatin (Ater et al. 2012). Of note, the overall response rate (ORR) (complete response [CR] + partial response [PR]) to chemotherapy in newly diagnosed pediatric low-grade glioma was noted to be 35% for vincristine/carboplatin (33/93 patients; 95% confidence interval [CI]: 27, 46) and 30% for TPCV (31/103 patients; 95% CI: 22, 40). Patients whose tumors progressed following the failure of these combinations have few treatment options. The largest study of chemotherapy in this population was performed with single-agent vinblastine, which resulted in an ORR (CR + PR) of 21% (11/51 patients), and a five-year PFS of 42.3% ± 7.2% (Bouffet et al. 2012). At present, there is no consensus on treatment options for patients who have relapsed, refractory, progressive, or disseminate low-grade glioma (Waanders et al. 2016), and no targeted therapeutics have been approved for this patient population.

Currently, the FDA and EMA have approved three agents that specifically inhibit BRAF V600E (vemurafenib, dabrafenib, encorafenib). All three of these Type I RAF inhibitors exert their effect only on the BRAF V600 mutation and they do not address the KIAA1549:BRAF fusions endemic in pediatric low-grade gliomas. Moreover, in the presence of monomeric mutant BRAF, the mechanism of binding of these inhibitors is such that it results in the induced dimerization and paradoxical activation of wild-type RAF proteins in nontumor tissue. This leads to a common adverse event (AE) associated with these agents, the development of proliferative premalignant and malignant skin lesions (e.g., keratoacanthomas and squamous cell carcinomas). In addition, Type I agents are not able to bind to the RAF kinase domains in KIAA1549:BRAF fusions, and are thus unable to effectively inhibit the hyperactive extracellular signal-related kinase (ERK) signaling that results from this fusion.

Importantly, Type I RAF inhibitors have been shown to result in paradoxical activation of pediatric low-grade glioma models driven by the KIAA1549:BRAF fusion and to accelerate the growth of tumor models driven by the KIAA1549:BRAF fusion. Clinical proof-of-concept for this phenomenon occurred in 2014 when the multikinase inhibitor sorafenib, which blocks BRAF V600 mutations in a manner similar to other Type I inhibitors, was studied in children with pediatric low-grade gliomas and produced unexpected and unprecedented acceleration in tumor growth. This effect was later found to be related to paradoxical ERK activation (Karajannis et al. 2014). As such, the Type I RAF inhibitors are considered to be contraindicated in the large percentage of patients with pediatric low-grade glioma without a BRAF V600E activating mutation. Although the currently available Type I BRAF inhibitors are being studied in children with BRAF V600 mutated low-grade gliomas, and potentially being used off-label in this patient population,

this is thought to be an inferior treatment option with only transient tumor control, as well as the safety issues commonly seen across the class of Type I RAF inhibitors.

Mitogen-activated protein kinase (MEK) inhibitors have been studied in pediatric low-grade glioma because of their ability to inhibit downstream signaling from BRAF V600E and KIAA1549:BRAF fusion. For example, selumetinib has been shown to cause tumor regression and disease stabilization (Fangusaro et al. 2019) without paradoxical activation. The largest study to date of selumetinib in recurrent, refractory, or progressive pediatric low-grade glioma patients with a BRAF alteration (n = 25) reported nine patients (36% [95% CI 18–57]) with a sustained partial response. The most frequent Grade 3 or greater AEs in this study were elevated creatine phosphokinase (CPK) (10%) and maculopapular rash (10%). It is important to note that available MEK inhibitors do not directly target the driver mutations, are not CNS penetrant, and are also not approved for the treatment of relapsed/refractory pediatric low-grade glioma.

With regard to vemurafenib, there are two published datasets. Initially, a retrospective chart review of patients with BRAF V600E-positive low-grade glioma treated off-label with vemurafenib (Del Bufalo et al., 2018) reported one CR, three PRs, and one patient with stable disease (SD). More recently, the Phase 1 portion of a prospective Phase 1/2 study was published, which established the adult recommended Phase 2 dose as the pediatric recommended Phase 2 dose. The objective response rate in the Phase 1 portion was found to be 32%. Centrally reviewed best radiographic responses included one CR, five PRs, and 13 patients with SD (Nicolaides et al., 2020). Vemurafenib was well tolerated with a similar safety profile to what has been seen in adults. The most common treatment-related Grade 3 or greater AEs in this study were secondary keratoacanthoma (n = 1); rash (n =16); and fever (n = 5).

The Type I RAF inhibitor dabrafenib has been investigated in pediatric patients with BRAF V600 mutant low-grade glioma (Hargrave et al., 2019). Thirty-two patients ranging in age from 2 to 17 years with locally determined BRAF V600 mutation–positive refractory or recurrent low-grade glioma were treated. Independent radiological review utilizing the Response Assessment in Neuro-Oncology (RANO) criteria confirmed an objective response rate of 44% (14/32, CI 26-62) which included one CR and 13 PRs. Eleven patients with SD had significant tumor responses. Therapy was well tolerated with the most common treatment-related AEs being fatigue (34%), rash (31%), dry skin (28%), pyrexia (28%), and maculopapular rash (28%) (Hargrave et al., 2019).

Finally, data generated from ongoing studies of various MAPK pathway inhibitors in patients with low-grade gliomas have shown that responses in pediatric low-grade gliomas driven by either BRAF V600 mutations (treated with Type I RAF inhibitors and MEK inhibitors) or KIAA1549:BRAF fusions (treated with MEK inhibitors) are often durable for only as long as drug can be administered. Thus, specificity of targeting and tolerability over a long course of treatment are key determinants of duration of response (DOR), and therefore of clinical benefit and treatment success.

Because of the natural history of pediatric low-grade gliomas, the goals of therapy for patients with relapsed or progressive pediatric low-grade glioma are a reduction of the burden of tumor, the prevention of continued growth, and avoidance of the short- and long-term toxicities associated with subsequent need for surgery, radiation therapy, or additional cytotoxic chemotherapy between the time of recurrence or progression and senescence. Although these tumors, and the associated treatment, are a significant source of morbidity in the developing child, if these children survive into young adulthood, many of these tumors undergo senescence (Jacob et al. 2011).

1.1.3. Pediatric Solid Tumors

Globally, approximately 400,000 children and adolescents aged 0 to 19 years are diagnosed with cancer each year (Steliarova-Foucher et al. 2017). The most common of these are acute lymphoblastic leukemia, brain and other CNS tumors, neuroblastoma, lymphoma, and thyroid cancer. Patients who respond to intensive treatment regimens and ultimately survive their disease face long-term morbidity and the risk of early mortality as a result of their treatment (Armstrong et al. 2010, Yeh et al. 2010, Kirchoff et al. 2010, Mitby et al. 2003, Pui et al. 2003). Although 5-, 10-, and 15-year disease-related mortality has improved in recent decades with better therapies and supportive care, many patients still relapse and eventually succumb to their disease. Currently, treatment for advanced cancers is typically histology based and varies across tumor types, most often including combination chemotherapy and/or immunotherapy.

The application of next-generation sequencing approaches to a large collection of human tumors has led to the identification of activating alterations in the BRAF or CRAF (also known as RAF1) gene, including fusions and mutations, in a fraction of other tumor types, including embryonal sarcomas, melanoma, thyroid cancers, and Langerhans histiocytosis (Coffin et al. 2020, Penning et al. 2021, Rankin et al. 2021). Currently, a number of these patients receive chemotherapy and/or undergo amputations or other disfiguring surgery as standard of care.

To improve efficacy and reduce toxicity, molecularly targeted therapies present an attractive approach. As described in Section 1.1.1, multiple targeted agents that have demonstrated clinical responses to patients with cancer harboring fusions involving ABL, ALK, TRK, RET, and ROS1 have been approved by the US FDA and EMA. However, despite the availability of these agents, there have been few successful applications for solid tumors in pediatric populations (Bellantoni et al. 2021).

1.1.3.1. Role of RAF Fusions in Pediatric Solid Tumors

In pediatric populations, solid tumors with an activating fusion in the BRAF or CRAF/RAF1 (collectively referred to as RAF) gene occur infrequently, but data continue to emerge indicating a diverse landscape of cancer types that harbor these alterations (Coffin et al. 2020, Penning et al. 2021, Rankin et al. 2021). The application of next-generation sequencing in larger datasets is contributing to an increased understanding of these tumors. In one recently published work of comprehensive genomic profiling

representing 3633 pediatric tumors, 221 (6.1%) of samples were found to harbor BRAF alterations likely to represent driver events. Forty-five of those samples represented solid tumors or hematologic malignancies and included known activating BRAF fusions such as KIAA1549:BRAF in embryonal rhabdomyosarcoma and sarcoma (not otherwise specified [NOS]), CUX1-BRAF in sarcoma (NOS), STAR-D3NL-BRAF in sarcoma (NOS), PPP1CC-BRAF in acinar cell carcinoma, and KHDRBS2-BRAF in large cell neuroendocrine carcinoma (Rankin et al. 2021). Activating BRAF fusions and mutations have recently been reported in mesenchymal tumors with IFS-like morphology (Coffin et al. 2020, Penning et al. 2021). Beyond activating BRAF fusions, activating CRAF/RAF1 fusions have also been detected in melanoma and low-grade glioma. These can also constitutively activate the MAPK pathway (Williams et al. 2020, Rankin et al. 2021, Wright et al. 2020). Importantly, few co-occurring genomic driver alterations were identified (Rankin et al. 2021).

As demonstrated in the Rankin, Coffin, Penning, Drilon, and Stransky works, oncogenic fusions normally occur in isolation from other driver mutations. This leads to the hypothesis that an activating RAF fusion of the BRAF or CRAF/RAF1 gene is the likely oncogenic alteration in these tumors. As with other known oncogenic drivers such as ALK, ROS-1, RET, and TRK, these solid tumors are amenable to monotherapy treatment, which can result in profound tumor shrinkage. DAY101, a pan-RAF inhibitor that has been shown to inhibit both BRAF and CRAF/RAF1 fusions (Sun et al. 2017, Wright et al. 2020), may provide clinical benefit from a molecularly targeted approach in pediatric patients whose tumors have progressed after standard of care therapy. Chemotherapy has a less than favorable side effect and long-term sequelae profile in a pediatric patient population, commonly leading to negative long-term outcomes.

Therefore, there is an urgent need to identify new targeted therapies that potently inhibit activating RAF fusions in tumors while sparing other kinase and non-kinase off-targets that contribute to significant toxicity. A significant number of patients with advanced cancers harboring a BRAF or CRAF/RAF1 fusion could benefit from potent and selective RAF kinase inhibition.

1.2. Background on DAY101

1.2.1. DAY101 Nonclinical Data

A comprehensive package of nonclinical studies has been conducted with DAY101 and provides a thorough pharmacological and toxicological profile of DAY101 in multiple species, as detailed in the Investigator's Brochure (IB).

DAY101 has been extensively studied in both in vitro and in vivo systems and has been found to result in potent inhibition of oncogenic BRAF V600E mutations (Olszanski et al. 2017). In contrast to the approved Type I RAF inhibitors (e.g., vemurafenib, dabrafenib, encorafenib), DAY101 inhibits both wild-type BRAF and CRAF/RAF1 kinases and, importantly, hyperactivated signaling resulting from BRAF fusions, including the KIAA1549:BRAF fusion (Sun et al. 2017). In vitro studies have demonstrated that DAY101

inhibits phosphorylated ERK (pERK) signaling, along with cell proliferation, in a wide array of cell lines with high MAPK activity. In vivo studies have shown that pERK is suppressed in xenograft tumor models harboring mutant BRAF, wild-type BRAF, or KIAA1549:BRAF fusions. Xenograft models also demonstrated antitumor activity resulting from monotherapy treatment with DAY101.

DAY101 has been shown to inhibit the kinase activity of BRAF in the context of BRAF kinase domain fusions with various 5′ gene partners, most notably fusion with the KIAA1549 gene. KIAA1549:BRAF fusion kinase activity is inhibited by DAY101 with comparable potency to inhibition of BRAF V600E in cell model systems, without the paradoxical activation of the MAPK pathway reported for Type I BRAF inhibitors (Sun et al. 2017).

Importantly, in contrast to Type I RAF inhibitors, DAY101 does not result in paradoxical activation of MAPK signaling in BRAF wild-type tissues (Sun et al. 2017), which limits the use of Type I inhibitors. In contrast to MEK inhibitors, DAY101 directly inhibits BRAF dimers, blocking downstream pERK signaling. As detailed below, the profile of DAY101 does not appear to have the severe dermatologic, cardiac, or ophthalmologic liabilities of the incumbent RAF or MEK inhibitors. Finally, as opposed to all approved MEK and BRAF inhibitors, DAY101 has significantly greater CNS penetration (Sun et al. 2017).

1.2.2. DAY101 Clinical Data

Two clinical studies (C28001 and C28002) in adult patients with advanced solid tumors were completed by the previous Sponsor. In addition, one investigator-initiated clinical study (PNOC014) is ongoing with DAY101 in pediatric patients with relapsed/refractory pediatric low-grade glioma. [redacted]

Study C28001 was a Phase 1 first-in-human study conducted in adult patients with advanced solid tumors. DAY101 was administered orally (PO) once every other day (Q2D) in 22- and 28-day treatment cycles, at doses of 20, 40, 80, 135, 200, and 280 mg. DAY101 was also administered once weekly (QW) in 28-day treatment cycles with a starting dose of 400 mg, then 600 and 800 mg. The adult maximum tolerated doses (MTDs) were reached at 200 mg Q2D and 600 mg QW. Expansion cohorts included patients with advanced, metastatic, or unresectable melanoma with MAPK alterations. Details of this study can be found in the DAY101 IB.

Study C28002 was a Phase 1b study conducted in patients with advanced solid tumors who had failed standard therapies. This study was conducted as an “umbrella study” in which DAY101 was studied in combination with various chemotherapy agents or targeted therapies in adult patients with either advanced colorectal cancer or advanced non–small cell lung cancer (with either KRAS exon 2 mutations or non-V600 BRAF mutations). Details of this study can be found in the DAY101 IB.

Study PNOC014 is an investigator-initiated, Phase 1, dose-escalation study in patients aged 1 to < 25 years with radiographically confirmed recurrent or progressive nonhematologic malignancies (solid tumors or primary CNS disease) with a genomic alteration resulting in the activation of the RAS/RAF/MEK/ERK pathway, excluding patients with NF1 loss-of-function. Part A of this ongoing study was a dose escalation that used a 3+3 design. DAY101 was administered as an oral tablet with a starting dose of 280 mg/m^2 (80% of the adult recommended Phase 2 dose [RP2D] of 600 mg PO QW, adjusted for body surface area [BSA]).

[redacted]

1.2.2.1. Clinical Pharmacokinetics

Details of the PK from Studies C28001, [redacted], and [redacted] are included in Section 1.3.2, as well as in the IB.

1.2.2.2. Clinical Efficacy

<u>Study C28001</u>

Preliminary antitumor activity was most evident in the BRAF mutation–positive, treatment-naive RP2D expansion melanoma cohort (ORR: 8/16 patients, 50% [95% CI: 25, 75]; median DOR [PR in 8 patients]: 6.0 months [95% CI: 3.7, not estimable]; median PFS: 5.7 months [95% CI: 1.9, 14.3]). A PR was also reported in 1 of 6 patients in the BRAF mutation–positive previously treated cohort (ORR: 17%; 95% CI: < 1, 64).

Further details of the efficacy data from C28001 can be found in the DAY101 IB.

[redacted]

[redacted]

1.2.2.3. Clinical Safety

Study C28001

Takeda Study C28001 was a Phase 1, multicenter, nonrandomized, open-label, FIH clinical study designed to evaluate the safety, tolerability, PK, pharmacodynamics, and antitumor activity of oral DAY101 in adult patients with advanced solid tumors (excluding lymphoma). The study was composed of two phases (dose-escalation and dose-expansion phases) and evaluated two dosing schedules (Q2D and QW). Patients in the dose-escalation phase were not selected based on BRAF status. The dose-expansion phase evaluated multiple cohorts of patients with melanoma, including patients who were BRAF mutation–positive (including cohorts of those who had, and those who had not, received a prior RAF or MEK inhibitor), NRAS mutation–positive (including cohorts of those who had, and those who had not, received a prior RAF or MEK inhibitor), and BRAF/NRAS mutation–negative patients (including cohorts who either had or had not received a prior immune checkpoint inhibitor).

Over the life of this study, which was completed on 16 October 2018, 110 patients were administered at least one dose of DAY101 on a Q2D dose schedule (30 in the dose-escalation phase and 80 in the dose-expansion phase). Thirty-nine patients were administered at least one dose of DAY101 on a QW dose schedule (20 in the QW dose-escalation phase [three in the 400-mg cohort, 13 in the 600-mg cohort, and four in the 800-mg cohort] and 19 dosed at 600 mg QW in the expansion phase).

Of the 149 patients treated, all had at least one treatment-emergent adverse event (TEAE). The most frequently reported TEAEs across all dose cohorts in the Q2D dose-escalation phase included fatigue (67%), maculopapular rash (37%), and arthralgia and constipation (30% each). The most frequently reported TEAEs in the Q2D dose-expansion phase included fatigue (43%), anemia (41%), constipation and maculopapular rash (38% each), nausea (36%), and dyspnea (30%).

The most frequently reported TEAEs across all dose cohorts in the QW dose-escalation phase included fatigue (55%), anemia (40%), nausea (35%), and headache (30%). In the QW dose-expansion cohort, the most frequently reported TEAE was fatigue (47%). More details can be found in the DAY101 IB.

The monotherapy treatment of 149 adults has provided a comprehensive database of safety information that allows the Sponsor to understand the potential AEs in the pediatric population. In addition, Takeda sponsored a second study (C28002; NCT 02327169) in 71 patients with non–small cell lung carcinoma. While this study provides additional supportive data on DAY101, applicability of the safety data from this study to pediatric low-grade glioma patients is lower due to the fact that in study C28002, DAY101 was used in

combination with other standard-of-care and experimental agents. In addition, Study C28002 was conducted in adult patients with relapsed non–small cell lung cancer, and thus these patients had different comorbidities that confound interpretation of AE data.

Additional safety data, including data of DAY101 in combination with other therapies from Study C28002, can be found in the IB.

[redacted]

[redacted]

[redacted] [redacted]

[redacted]	[redacted]
[redacted]	[redacted]
[redacted]	[redacted]
[redacted]	[redacted]
[redacted]	[redacted]
[redacted]	[redacted]
[redacted]	[redacted]
[redacted]	[redacted]
[redacted]	[redacted]
[redacted]	[redacted]
[redacted]	[redacted]
[redacted]	[redacted]
[redacted]	[redacted]
[redacted]	[redacted]
[redacted]	[redacted]
[redacted]	[redacted]

[redacted]	[redacted]
[redacted]	[redacted]
[redacted]	[redacted]
[redacted]	[redacted]
[redacted]	[redacted]
[redacted]	[redacted]
[redacted]	[redacted]
[redacted]	[redacted]
[redacted]	[redacted]
[redacted]	[redacted]
[redacted]	[redacted]
[redacted]	[redacted]
[redacted]	[redacted]
[redacted]	[redacted]
[redacted]	[redacted]
[redacted]	[redacted]
[redacted]	[redacted]
[redacted]	[redacted]
[redacted]	[redacted]
[redacted]	[redacted]
[redacted]	[redacted]
[redacted]	[redacted]
[redacted]	[redacted]
[redacted]	[redacted]
[redacted]	[redacted]
[redacted]	[redacted]
[redacted]	[redacted]
[redacted]	[redacted]

[redacted]

1.3. Study Rationale and Dose Justification

This is a Phase 2 study evaluating DAY101 monotherapy in pediatric patients with low-grade gliomas and advanced solid tumors. Patients will be enrolled to one of three treatment arms. Arm 1 is to enroll approximately 60 patients with a pediatric low-grade glioma harboring an activating BRAF alteration. Arm 2 is an extension cohort to enroll patients with a low-grade glioma harboring an activating RAF alteration (e.g., BRAF or CRAF/RAF1 fusion or BRAF

V600 mutations), to provide treatment options to patients following the closure of Arm 1 and prior to commercial availability of DAY101. Arm 3 is to enroll patients with advanced solid tumors harboring activating RAF fusions (e.g., BRAF or CRAF/RAF1 fusions).

1.3.1. Study Rationale

Relapsed or progressive pediatric low-grade glioma is a serious, life-altering, and potentially fatal disease with significant disease- and treatment-associated morbidity. There is currently no standard of care for patients who progress after initial therapy and no FDA-approved therapies. As noted in Section 1.1, a significant body of data clearly shows that a majority of patients with pediatric low-grade glioma also have an alteration in BRAF that drives tumor growth: either a KIAA1549:BRAF fusion, or a BRAF V600 mutation. Type I BRAF inhibitors are mechanistically only able to inhibit the BRAF V600 mutations. Using a Type I RAF inhibitor in a patient with a BRAF fusion has the potential to cause paradoxical activation of the MAPK pathway and tumor growth. Because the majority of BRAF alterations in patients with pediatric low-grade glioma are BRAF fusions, use of Type I RAF inhibitors is not indicated. Currently approved MEK inhibitors can block MAPK signaling resulting from either BRAF V600 mutations or the KIAA1549:BRAF fusion, but do not directly inhibit BRAF. In addition, MEK inhibitors are associated with significant cardiac, dermatologic, and ophthalmologic toxicities. The Type II BRAF inhibitor DAY101 has been shown preclinically to be able to inhibit both BRAF V600 and BRAF fusion alterations, and has been shown to have significantly greater CNS penetration than any of the available Type I RAF inhibitors or MEK inhibitors. [redacted]

As noted in Section 1.1.3.1, a significant number of patients with advanced solid tumors harboring a BRAF or CRAF/RAF1 fusion could benefit from potent and selective RAF kinase inhibition. The application of receptor tyrosine kinase inhibition to treat driver alterations across different tumor types and ages was demonstrated by the tissue-agnostic approval of larotrectinib in patients of any age with TRK fusion–positive solid tumors. An ORR of 75% by independent review was reported in 55 patients ranging from 4 months to 76 years, spanning 17 different tumor histologies (Drilon et al. 2018). Similar tissue-agonistic activity of DAY101 is anticipated based on the mechanism of action targeting molecular subgroups. Recently, an 8-year-old male with a spindle cell sarcoma harboring a novel SNX8-BRAF fusion received DAY101 in a compassionate-use basis. The patient exhibited a near-complete response following two cycles of therapy (Offer et al. 2021). Treatment is ongoing at the time of this protocol finalization.

The purpose of this study is to evaluate the efficacy and safety of DAY101 as monotherapy in pediatric patients with a recurrent or progressive low-grade glioma or advanced solid tumor harboring a known activating RAF alteration. The primary endpoint is ORR as defined by the RANO or RECIST v1.1 criteria, determined by an independent radiology review committee (IRC).

Rationale for Arm 2: Low-Grade Glioma Extension

Due to the lack of treatment options for patients with low-grade gliomas harboring activating BRAF alterations, established in Section 1.1.2 , the Sponsor opened FIREFLY-1 on 1 March 2021.

In order to provide access of a targeted therapy to a defined patient population, Arm 2 will open based on recommendation of the Data Safety Monitoring Board (DSMB) to enroll additional patients prior to approval of DAY101 and access to commercial drug. This arm will include the same patient population as Arm 1, with the addition of CRAF/RAF1 fusions. Patients with low-grade gliomas harboring CRAF/RAF1 fusions have been identified, and preliminary data have shown that DAY101 is effective in this patient population (Sun et al. 2017, Wright et al. 2020). Adding the extension arm into the FIREFLY-1 study would ensure patients receive continuous access to DAY101 following the complete enrollment of the primary analysis arm (intent-to-treat [ITT] population) and until the drug is commercially available to new patients.

1.3.2. Justification for Dose

A sustained, high degree of target suppression in tumor tissue has been reported to be necessary for maximal activity with RAF and MEK inhibitors in adult solid tumors, both in nonclinical models and in the vemurafenib Phase I clinical study (Flaherty et al. 2010). In preclinical pharmacodynamic studies in tumor-bearing mice, DAY101 caused strong and sustained suppression of the pERK biomarker at a dose of 50 mg/kg (> 80% suppression for > 16 hours following a single oral dose). This dose also caused antitumor activity in multiple tumor xenograft models, on both once-daily and Q2D dosing schedules. This strong and sustained inhibition of RAF is expected to translate to the clinical setting because the half-life ($t_{1/2}$) of DAY101 is significantly longer in humans (63 hours) than in mice (8 hours). Therefore, it is anticipated that administering DAY101 on a once-weekly schedule will provide the required degree of target inhibition over the 168-hour dosing interval. DAY101 is not a substrate for P-glycoprotein and is an inhibitor of breast cancer resistance protein (BCRP); thus, it is expected that a once-weekly dose of DAY101 will achieve a maximum drug concentration (C_{max}) for optimal CNS penetration. Furthermore, the low rate of accumulation of DAY101 with the once-weekly dose schedule while maintaining continuous exposure at a steady-state concentration that inhibits BRAF-driven disease is predicted to reduce the risk of toxicity due to drug accumulation.

The initial safety and clinical activity of the 600-mg once-weekly dose of DAY101 appears supportive of this exposure-AE modeling.

Study C28001

The PK of DAY101 has been well characterized in the dose-escalation phases of the first-in-human, Phase 1 study C28001 in adult patients with advanced solid tumors. In Study C28001, DAY101 was administered PO Q2D in 22-day and 28-day treatment cycles at doses of 20, 40, 80, 135, 200, and 280 mg. DAY101 was also administered QW in 28-day treatment cycles with a starting dose of 400 mg, then 600 and 800 mg. The adult MTDs were reached at 200 mg Q2D and 600 mg QW.

DAY101 was absorbed into systemic circulation following oral administration with a median time to maximum concentration between 2 and 4 hours postdose. Steady-state DAY101 area under the concentration-time curve (AUC) increased in an approximately dose-proportional manner over the dose ranges of 20 to 280 mg Q2D and 400 to 800 mg QW. Administration of DAY101 PO Q2D resulted in approximately 2.5-fold accumulation in area under the concentration-time curve from time zero to 48 hours at steady state. No apparent accumulation was observed with the once-weekly dose regimens. The terminal $t_{1/2}$ of DAY101 was 63 hours. At both 200 mg Q2D and 600 mg QW, DAY101 AUC exceeded the AUC (111,000 ng • h/mL) associated with 83% tumor growth inhibition in the WM-266-4 melanoma xenograft model.

[redacted]

[redacted]

[redacted]

[redacted]

[redacted] [redacted]

[redacted]	[redacted]	[redacted]	[redacted]	[redacted]	[redacted]	[redacted]	[redacted]	[redacted]
[redacted]	[redacted]	[redacted]	[redacted]	[redacted]	[redacted]	[redacted]	[redacted]	[redacted]
[redacted]	[redacted]	[redacted]	[redacted]	[redacted]	[redacted]	[redacted]	[redacted]	[redacted]
[redacted]	[redacted]	[redacted]	[redacted]	[redacted]	[redacted]	[redacted]	[redacted]	[redacted]
[redacted]	[redacted]	[redacted]	[redacted]	[redacted]	[redacted]	[redacted]	[redacted]	[redacted]
[redacted]	[redacted]	[redacted]	[redacted]	[redacted]	[redacted]	[redacted]	[redacted]	[redacted]
[redacted]	[redacted]	[redacted]	[redacted]	[redacted]	[redacted]	[redacted]	[redacted]	[redacted]
[redacted]	[redacted]	[redacted]	[redacted]	[redacted]	[redacted]	[redacted]	[redacted]	[redacted]
[redacted]	[redacted]	[redacted]	[redacted]	[redacted]	[redacted]	[redacted]	[redacted]	[redacted]

[redacted]

[redacted]

[redacted]

[redacted]

[redacted]

[redacted] [redacted]

[redacted]

[redacted]

[redacted] [redacted]

[redacted]

[redacted].

[redacted]

1.3.2.1. Potential Developmental Toxicities and Dosing Considerations

In repeat-dose toxicity studies of DAY101 in the rat, target organs included the ovary, vagina, and testes. These findings were not observed in the repeat-dose studies in monkey, which may be more relevant to humans as these pathways are similar between monkeys and humans (Haschek 2002). Dedicated reproduction toxicity studies, including a fertility study, are ongoing to evaluate the effects of DAY101 on the reproductive organs.

Nonclinical juvenile toxicity data are available for other drugs in the same pharmacological class (e.g., the RAF inhibitor, dabrafenib), as well as for MEK inhibitors, which target the same MAPK signaling pathway (e.g., trametinib and cobimetinib). Furthermore, data from fertility and embryofetal development studies has been generated with other drugs in the same pharmacological class (e.g., the RAF inhibitors, dabrafenib and encorafenib) as well as for MEK inhibitors (binimetinib and trametinib). In addition, safety data is available from 32 pediatric low-grade glioma patients enrolled in a Phase 1/2a study with dabrafenib (Hargrave et al., 2019). With a minimum follow-up of 26.2 months, dabrafenib exhibited a favorable tolerability profile that was consistent with that observed in adults across other indications. Results from all these studies have been taken into consideration in the safety monitoring program for this study.

The justification for the clinical dose took into consideration multiple parameters including the nonclinical safety data and the observed clinical data from the adult study. In the three-month repeat-dose GLP rat toxicology study, at the NOAEL of 50 mg/kg for male rats, the total AUC_{24hr} was 184,000 hr•ng/mL and unbound AUC_{24hr} was 976 hr•ng/mL. Assuming dose linearity, the unbound exposure of AUC_{24hr}, multiplied by 7, provides an estimated AUC_{168hr} that would cover the dosing period of a QW administration. [redacted] In the three-month repeat-dose GLP toxicology study in cynomolgus monkeys, at the NOAEL of 3 mg/kg for male monkeys, the total AUC_{24hr} was 17,800 hr•ng/mL, and unbound AUC_{24hr} was 766 hr•ng/mL. [redacted]

[redacted]

1.3.2.2. Drug Metabolism

The selected RP2D of DAY101 in this study (420 mg/m^2; not to exceed a dose of 600 mg) is based on data from the pediatric Phase 1 study [redacted]

DAY101 is metabolized by cytochrome P450 2C8 (CYP2C8) and has a low potential for drug-drug interactions. In addition, CYP2C8 inhibitors or inducers are prohibited in this study. Furthermore, the RP2D for DAY101 (see Section 1.3.2) was not the MTD, thereby reducing potential toxicity.

1.3.3. Risk Management

A safety review committee (SRC) will oversee the safety of the study (see Section 8.2) and will render dose safety decisions. The SRC membership will consist of the Sponsor's Medical Monitor for this study, a Clinical Operations representative, and a clinically qualified representative of the Principal Investigator from each active clinical site contributing patients to that cohort. The SRC will meet monthly during enrollment, and quarterly until study closure.

In addition, an independent DSMB will be established and will meet approximately every three months to review safety and efficacy data across the entire DAY101 program. Any emergent safety issue identified by the SRC will be escalated to the DSMB.

The safety profile of DAY101 was established in the adult first-in-human study C28001 [redacted]. For this study, the algorithms for potential adverse effects of both BRAF inhibitors as well as MEK inhibitors will be used in addition to supportive care measures (see Section 5.2.3). For expected toxicity of DAY101, in general, dose delays and observation are adequate for low-grade AEs. For moderate- and high-grade AEs, dose reductions will also be used (see Sections 5.2.2 and 5.2.3).

1.3.4. Required Study Procedures

This study will be conducted at sites with experience in pediatric oncology early phase studies. Nevertheless, all investigators will ensure that measures to reduce pain and discomfort for all enrolled patients are used as much as reasonably possible. Examinations will be adapted to the age of children.

After baseline examinations and initiation of treatment, monthly study visits with standard-of-care and study-specific examinations will be performed (see Section 6 and Appendix A). Response assessments will be performed every three cycles after initiation of treatment for patients in Arms 1 and 2. Magnetic resonance imaging is the preferred modality. For Arm 3, response assessments will be performed every two cycles after initiation of treatment for 12 months of treatment, then every three cycles thereafter. For young patients, sedation may be necessary for imaging according to institutional guidelines. Overall, the majority of procedures correspond to standard of care in this pediatric cancer population.

For younger children, blood volumes drawn will be minimized, in accordance with the European Commission (EC)'s recommendations, "Ethical considerations for clinical trials on medicinal products conducted with the pediatric population" (EC 2008) and "Ethical considerations for clinical trials on medicinal products conducted with minors" (EC 2017).

1.3.5. Continuous Review of Safety Data

The eCRF used for this study allows real-time tracking of safety data. Safety data review will be reviewed by the Sponsor or designee regularly, and safety signals or trends will be escalated to the SRC and DSMB accordingly.

Serious adverse events (SAEs) will be reported within 24 hours to the safety team by email and be subject to a second assessment by the safety physician (see Section 8.1.4).

1.3.6. Potential Benefit for Patients in Clinical Trials and Patients in General

The available nonclinical and clinical data of DAY101 predict responses in a clinically relevant group of patients.

In patients with RAF-altered low-grade glioma, there have been two CRs, three PRs, two SDs, and two patients with primary progressive disease (ORR: 56%). Median TTR was 10.5 weeks (range: 8–32 weeks) (see Section 1.2.2.2). Two patients reached end of treatment (i.e., completed two years of therapy). In addition, no patients with a confirmed response have progressed or discontinued therapy due to toxicity. One patient with a spindle cell sarcoma harboring a BRAF fusion, who was treated in a compassionate-use setting, has achieved a near-complete response (Offer et al. 2021).

The expected high response rate and short TTR will potentially improve tumor burden and induced functional deficits such as visual and motor function impairment (see Section 1.1.2), associated with positive impact on growth and development of patients.

Compared with the majority of currently applied chemotherapy regimens, which require hospitalization/in-patient treatment, patients enrolled in this study will receive an oral drug that is taken at home, allowing continuation of daily activities such as attending school. For future patients, the efficacy and safety data generated from this study can potentially address the unmet clinical need in treatment of patients with solid tumors harboring a BRAF alteration.

1.3.7. Integrated Benefit/Risk Assessment

As discussed in Sections 1.1.2 and 1.1.2.1, pediatric low-grade glioma is a highly morbid, but not a life-threatening, disease. Patients suffer from a relentless progression of symptoms directly related to a tumor growth in a closed space. Additionally, long-term survivors may experience considerable chronic toxicities as a result of the current standard approaches to therapy. The chances for cure in children with relapsed or refractory pediatric low-grade gliomas are minimal, which illustrates an unmet medical need for innovative treatment strategies in this population. Currently, there are no standard treatment options available for this patient group. The vast majority of patients receive non-controlled, individual experimental treatments outside of clinical trials according to institutional experience (see Section 1.1.2.1).

Advanced solid tumors harboring activating RAF fusions have an unmet medical need in identifying treatments specifically targeted to the oncogenic driver alteration, as discussed in Sections 1.1.3 and 1.3.1. Children and adolescents with relapsed or progressive solid tumors harboring an activating RAF fusion that have exhausted current therapeutic options do have a realistic potential to benefit from this study.

Phase 1 clinical data of DAY101 in children confirmed the RP2D dose in the pediatric population (420 mg/m^2, with a maximum dose of 600 mg) and confirm the same favorable safety profile as reported in adults. Nonclinical data detail CYP2C8 metabolism and low risk for drug-drug interaction. Clinical data from the Phase 1 trials confirm a safe and tolerable AE profile of DAY101 in adults. The study protocol adequately addresses safety concerns, such as potential male and female reproductive organ toxicity in pediatric patients, using measures of strict safety monitoring covering all foreseeable relevant toxicities and potential target organs, including adequate contraceptive measures.

In summary, the nonclinical and clinical data in pediatric patients treated with DAY101 to date show a favorable benefit/risk profile for conducting this study in pediatric patients with RAF-altered CNS and other solid tumors that have not shown clinical improvement with current standard-of-care therapeutic regimens.

2. STUDY OBJECTIVES

Study objectives and endpoints are outlined in Table 4, Table 5, and Table 6.

Table 4: Arm 1 (Low-Grade Glioma) Objectives and Endpoints

PRIMARY OBJECTIVE AND ENDPOINTS	
**OBJECTIVE**	**ENDPOINTS**
• To evaluate the efficacy of DAY101 as measured by the overall response rate (ORR) as determined by an independent radiology review committee (IRC) following treatment with DAY101 in pediatric patients aged 6 months to 25 years, inclusive, with a relapsed or progressive low-grade glioma harboring a known activating BRAF alteration	• Overall response rate, defined as the proportion of patients with best overall confirmed response of complete response (CR) or partial response (PR) as determined by the Response Assessment in Neuro-Oncology (RANO) criteria
**SECONDARY OBJECTIVES AND ENDPOINTS**	
**OBJECTIVE**	**ENDPOINTS**
• To assess the safety and tolerability of DAY101	• Type, frequency, and severity of AEs and laboratory abnormalities
• To determine the relationship between pharmacokinetics (PK) and drug effects, including efficacy and safety	• Pharmacokinetic profile of DAY101 (e.g., area under the concentration-time curve [AUC], C_{min}, etc.)

• To evaluate the effect of DAY101 on the QT interval corrected for heart rate by Fridericia's formula (QTcF) prolongation and to explore the effects of DAY101 on electrocardiogram (ECG) parameters	• Change from baseline QT interval corrected for HR by Fridericia's formula (ΔQTcF)<br>• Change from baseline PR interval (ΔPR)<br>• Change from baseline QRS interval (ΔQRS)<br>• Change from baseline heart rate (ΔHR)<br>• ECG waveform morphology
• To determine the ORR based on the treating investigator's response assessment using RANO criteria	• Measured by the proportion of patients with best overall confirmed response of CR or PR by RANO criteria
• To determine the ORR based on Response Assessment in Pediatric Neuro-Oncology (RAPNO)–low-grade glioma criteria as determined by an IRC	• Measured by the proportion of patients with best overall confirmed response of CR or PR by RAPNO–low-grade glioma criteria
• To evaluate the duration of progression-free survival (PFS) based on RANO and RAPNO criteria following initiation of DAY101 as determined by 1) an IRC and 2) the treating investigator (RANO only)	• Measured by the time following initiation of DAY101 to progression or death in patients treated with DAY101
• To evaluate the duration of response (DOR) in patients with best overall response (BOR) of CR or PR based on RANO and RAPNO criteria as determined by 1) an IRC and 2) the treating investigator (RANO only)	• Measured by the length of response in patients with best overall confirmed response of CR or PR by RANO and RAPNO criteria, as applicable
• To evaluate time to response (TTR) (CR or PR based on RANO and RAPNO criteria) following initiation of DAY101 as determined by 1) an IRC and 2) the treating investigator (RANO only)	• Measured by the time to first response following initiation of DAY101 in patients with best overall confirmed response of CR or PR by RANO and RAPNO criteria
• To evaluate the clinical benefit rate based on the proportion of patients with BOR of CR, PR, or stable disease (SD), based on RANO and RAPNO criteria, lasting 12 months or more following initiation of DAY101, as determined by 1) an IRC and 2) the treating investigator (RANO only)	• Measured by the proportion of patients with BOR of CR, PR, or SD lasting 12 months or more following initiation of DAY101
• To evaluate changes in best corrected visual acuity (BCVA) outcomes	• Measured by change from baseline in BCVA (converted as logMAR) for each eye
• To evaluate the concordance of prior local laboratory BRAF molecular profiling with a central BRAF alteration assay being evaluated by the Sponsor	• Molecular analysis of cells obtained from archival tissue
**EXPLORATORY OBJECTIVES AND ENDPOINTS**	
**OBJECTIVES**	**ENDPOINTS**
• To compare the response and time to progression following initiation of DAY101 to that of the prior line of systemic therapy	• Measured by the proportion of patients with best overall confirmed response of CR or PR and TTR by RANO criteria based on the prior line of therapy

• To characterize changes in total tumor volume following treatment with DAY101 by magnetic resonance imaging (MRI) volumetric image analysis	• Measured by determining tumor volume and volume changes based on MRI scan data
• To characterize changes in apparent diffusion coefficients following treatment with DAY101 using diffusion-weighted imaging analysis	• Measured by diffusion-weighted imaging based on MRI scan data
• To evaluate changes in quality-of-life and health utilities measures using the Pediatrics Quality of Life™—Core Module (PedsQL-Core), Pediatrics Quality of Life™—Cancer (PedsQL-Cancer), and Patient-Reported Outcomes Measurement Information System (PROMIS®) assessment	• Measured by changes from baseline in quality-of-life and health utilities measures using the PedsQL-Core, PedsQL-Cancer, and PROMIS® assessment
• To describe the improvement in motor function	• Measured by changes from baseline in the Vineland 3 Adaptive Behavior Scales
• To determine the durability of response following discontinuation of DAY101 for patients with a radiographic response to DAY101 (CR or PR as based on RANO and RAPNO criteria) as determined by 1) an IRC and 2) the treating investigator (RANO only)	• Measured by the proportion of patients with best overall confirmed response of CR or PR who enter a drug holiday period and time to progression as determined by RANO, RAPNO, or clinical criteria
• To evaluate time to initiation of next treatment following discontinuation of DAY101	• Measured by the proportion of patients who discontinue DAY101 therapy and time to next therapy initiation

Table 5: Arm 2 (Low-Grade Glioma Extension) Objectives and Endpoints

**PRIMARY OBJECTIVES AND ENDPOINTS**	
**OBJECTIVE**	**ENDPOINTS**
• To assess the safety and tolerability of DAY101 in pediatric patients aged 6 months to 25 years, inclusive, with a relapsed or progressive low-grade glioma harboring a known or expected to be activating RAF alteration	• Type, frequency, and severity of AEs and laboratory abnormalities
**SECONDARY OBJECTIVES AND ENDPOINTS**	
**OBJECTIVE**	**ENDPOINTS**
• To determine the ORR per RANO criteria as determined by 1) an IRC and 2) the treating investigator	• Measured by the proportion of patients with best overall confirmed response of CR or PR as determined by the RANO criteria
• To determine the ORR based on RAPNO–low-grade glioma criteria as determined by an IRC	• Measured by the proportion of patients with best overall confirmed response of CR or PR by RAPNO–low-grade glioma criteria
• To evaluate the duration of PFS based on RANO and RAPNO criteria following initiation of DAY101 as determined by 1) an IRC and 2) the treating investigator (RANO only)	• Measured by the time following initiation of DAY101 to progression or death in patients treated with DAY101

• To evaluate the DOR in patients with BOR of CR or PR based on RANO and RAPNO criteria as determined by 1) an IRC and 2) the treating investigator (RANO only)	• Measured by the length of response in patients with best overall confirmed response of CR or PR by RANO and RAPNO criteria, as applicable
• To evaluate TTR (CR or PR based on RANO and RAPNO criteria) following initiation of DAY101 as determined by 1) an IRC and 2) the treating investigator (RANO only)	• Measured by the time to first response following initiation of DAY101 in patients with best overall confirmed response of CR or PR by RANO and RAPNO criteria, as applicable
• To evaluate the clinical benefit rate based on the proportion of patients with BOR of CR, PR, or SD, based on RANO and RAPNO criteria, lasting 12 months or more following initiation of DAY101, as determined by 1) an IRC and 2) the treating investigator (RANO only)	• Measured on the proportion of patients with BOR of CR, PR, or SD lasting 12 months or more following initiation of DAY101
• To determine the relationship between PK and drug effects, including efficacy and safety	• Pharmacokinetic profile of DAY101 (e.g., AUC, C_{min}, etc.)
• To evaluate the effect of DAY101 on QTcF prolongation and to explore the effects of DAY101 on ECG parameters	• ΔQTcF<br>• ΔPR<br>• ΔQRS<br>• ΔHR<br>• ECG waveform morphology
**EXPLORATORY OBJECTIVES AND ENDPOINTS**	
**OBJECTIVES**	**ENDPOINTS**
• To characterize changes in total tumor volume following treatment with DAY101 by MRI volumetric image analysis	• Measured by determining tumor volume and volume changes based on MRI scan data
• To characterize changes in apparent diffusion coefficients following treatment with DAY101 using diffusion-weighted imaging analysis	• Measured by diffusion-weighted imaging based on MRI scan data
• To evaluate changes in BCVA outcomes	• Measured by change from baseline BCVA (converted as logMAR) for each eye
• To evaluate changes in quality-of-life and health utilities measures using the PedsQL-Core, PedsQL-Cancer, and PROMIS® assessment	• Measured by changes from baseline in quality-of-life and health utilities measures using the PedsQL-Core, PedsQL-Cancer, and PROMIS® assessment
• To describe the improvement in motor function	• Measured by changes from baseline in the Vineland 3 Adaptive Behavior Scales
• To determine the durability of response following discontinuation of DAY101 for patients with a radiographic response to DAY101 (CR or PR as based on RANO and RAPNO criteria) as determined by 1) an IRC and 2) the treating investigator (RANO only)	• Measured by the proportion of patients with best overall confirmed response of CR or PR who enter a drug holiday period and time to progression as determined by RANO, RAPNO, or clinical criteria

• To evaluate the concordance of prior local laboratory BRAF molecular profiling with a central BRAF alteration assay being evaluated by the Sponsor	• Molecular analysis of cells obtained from archival tissue

Table 6: Arm 3 (Advanced Solid Tumor) Objectives and Endpoints

PRIMARY OBJECTIVES AND ENDPOINTS	
**OBJECTIVE**	**ENDPOINTS**
• To evaluate the preliminary efficacy of DAY101 as measured by the ORR as determined by an IRC following treatment with DAY101 in pediatric patients aged 6 months to 25 years, inclusive, with a relapsed or progressive advanced solid tumor harboring a known or expected to be activating RAF fusion	• Measured by the proportion of patients with best overall confirmed response of CR or PR by RECIST v1.1 criteria
**SECONDARY OBJECTIVES AND ENDPOINTS**	
**OBJECTIVE**	**ENDPOINTS**
• To assess the safety and tolerability of DAY101 in pediatric patients with advanced solid tumors	• Type, frequency, and severity of AEs and laboratory abnormalities
• To determine the relationship between PK and drug effects, including efficacy and safety	• Pharmacokinetic profile of DAY101 (e.g., AUC, C_{min}, etc.)
• To evaluate the effect of DAY101 on QTcF prolongation and to explore the effects of DAY101 on ECG parameters	• ΔQTcF<br>• ΔPR<br>• ΔQRS<br>• ΔHR<br>• ECG waveform morphology
• To determine the ORR based on the treating investigator's response assessment using RECIST v1.1 criteria	• Measured by the proportion of patients with best overall confirmed response of CR or PR by RECIST v1.1 criteria
• To evaluate the duration of PFS based on RECIST v1.1 criteria following initiation of DAY101 as determined by 1) an IRC and 2) the treating investigator	• Measured by the time following initiation of DAY101 to progression or death in patients treated with DAY101
• To evaluate the DOR in patients with BOR of CR or PR based on RECIST v1.1 criteria as determined by 1) an IRC and 2) the treating investigator	• Measured by the length of response in patients with best overall confirmed response of CR or PR by RECIST v1.1 criteria
• To evaluate TTR (CR or PR based on RECIST v1.1 criteria) following initiation of DAY101 as determined by 1) an IRC and 2) the treating investigator	• Measured by the time to first response following initiation of DAY101 in patients with best overall confirmed response of CR or PR by RECIST v1.1

• To evaluate the clinical benefit rate based on the proportion of patients with BOR of CR, PR, or SD, based on RECIST v1.1 criteria, lasting 12 months or more following initiation of DAY101 as determined by 1) an IRC and 2) the treating investigator	• Measured on the proportion of patients with BOR of CR, PR, or SD lasting 12 months or more following initiation of DAY101
• To evaluate the concordance of prior local laboratory RAF molecular profiling with a central RAF alteration assay being evaluated by the Sponsor	• Molecular analysis of cells obtained from archival tissue
**EXPLORATORY OBJECTIVES AND ENDPOINTS**	
**OBJECTIVES**	**ENDPOINTS**
• To evaluate changes in quality-of-life and health utilities measures using the PedsQL-Core, PedsQL-Cancer, and PROMIS® assessment	• Measured by changes from baseline in quality-of-life and health utilities measures using the PedsQL-Core, PedsQL-Cancer, and PROMIS® assessment
• To determine the durability of response following discontinuation of DAY101 for patients with a radiographic response to DAY101 (CR or PR as based on RECIST v1.1 criteria) as determined by 1) an IRC and 2) the treating investigator	• Measured by the proportion of patients with best overall confirmed response of CR or PR who enter a drug holiday period and time to progression as determined by RECIST v1.1 or clinical criteria

3. INVESTIGATIONAL PLAN

3.1. Study Design

This is a Phase 2, multicenter, multi-arm, open-label study evaluating DAY101 in pediatric patients with low-grade gliomas and advanced solid tumors. The study will consist of the following treatment arms:

- **Arm 1 (Low-Grade Glioma):** Patients aged 6 months to 25 years, inclusive, with recurrent or progressive low-grade glioma harboring a known activating BRAF alteration, including BRAF V600 mutations and KIAA1549:BRAF fusions.
- **Arm 2 (Low-Grade Glioma Extension):** Patients aged 6 months to 25 years, inclusive, with recurrent or progressive low-grade glioma harboring a known or expected to be activating RAF alteration. Opening of Arm 2 to enrollment will be based on the recommendation of the DSMB as described below in Section 3.1.2.
- **Arm 3 (Advanced Solid Tumor):** Patients aged 6 months to 25 years, inclusive, with advanced solid tumors harboring a known or expected to be activating RAF fusion.

Study objectives, endpoints, eligibility criteria, assessments, treatment schedule, and planned safety and efficacy analyses for Arm 1 (patients with relapsed or progressive pediatric low-grade glioma, as originally written and agreed upon with regulatory agencies) are consistent with the previous version(s) of the protocol, unless otherwise specified in the

summary of changes. The Sponsor continues to plan for registration based on data from Arm 1. The study design schema is shown in Figure 5.

Figure 5: Study Design Schema

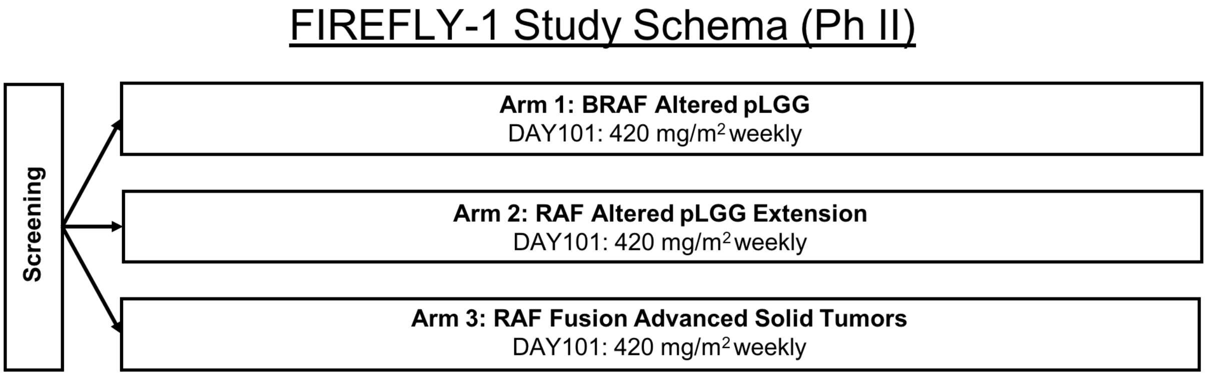

Abbreviations: pLGG, pediatric low-grade glioma.

The study will consist of a screening period, a treatment period, an end of treatment (EOT) visit, a safety follow-up visit, and long-term follow-up assessments. Ongoing safety, disease stability/progression, survival, and subsequent anticancer therapies will be assessed in the long-term follow-up period.

For all arms, treatment cycles will repeat every 28 days in the absence of disease progression or unacceptable toxicity. Patients will continue on DAY101 until radiographic evidence of disease progression as determined by treating investigator, unacceptable toxicity, decision to enter a “drug holiday” period, patient withdrawal of consent, or death.

Patients will be treated with DAY101 for a planned period of 26 cycles (approximately 24 months), after which they may continue on DAY101 or, at any point, opt to enter a “drug holiday” discontinuation period. Patients will continue to undergo routine periodic radiographic evaluations per protocol-defined timelines during the discontinuation period and may be re-treated with DAY101 if there is radiographic evidence of disease progression after drug discontinuation.

Patients who have radiographic evidence of disease progression may be allowed to continue DAY101 if, in the opinion of the investigator, and approved by the Sponsor, the patient is deriving clinical benefit from continuing study treatment. Patients being treated beyond progression with DAY101 will be reconsented prior to continuation of therapy. Disease assessments for patients being treated beyond progression should continue as per regular schedule.

DAY101 will be administered at the RP2D dose of 420 mg/m^2 (not to exceed 600 mg), PO QW (Days 1, 8, 15, and 22 of a 28-day cycle). Body surface area will be determined by the

Mösteller Formula [√(height × weight)/3600)] (Mosteller 1987). DAY101 is administered as an oral tablet or reconstituted liquid suspension formulation.

A schedule of assessments is provided in Appendix A. The study treatment schema is shown in Figure 6.

Figure 6: Treatment Schema

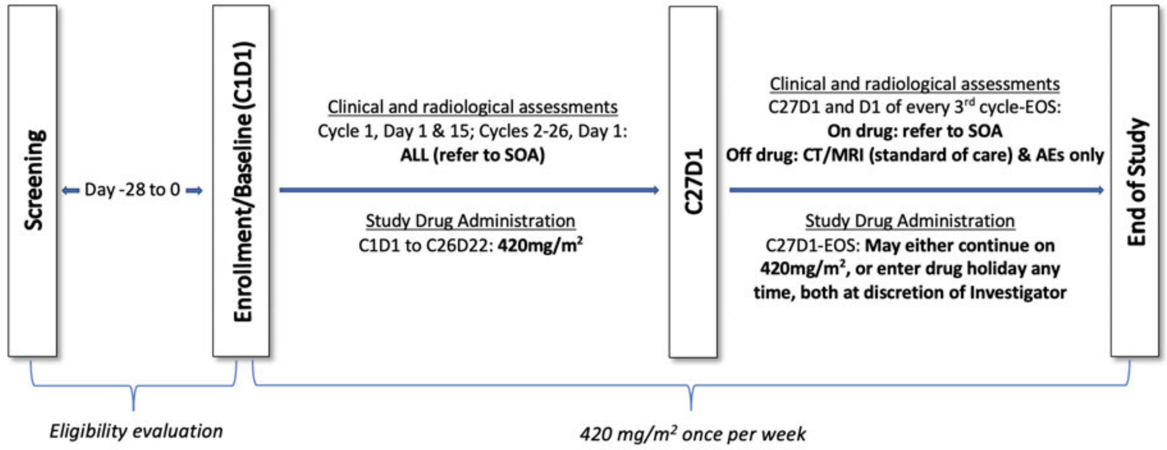

Abbreviations: C, cycle; D, day; EOS, end of study; CT, computerized tomography; MRI, magnetic resonance imaging; SOA, schedule of assessments.

3.1.1. Arm 1: Low-Grade Glioma

Arm 1 will enroll patients aged 6 months to 25 years, inclusive, with recurrent or progressive low-grade glioma harboring a known activating BRAF alteration, including BRAF V600 mutations and KIAA1549:BRAF fusions. Patients with BRAF alterations will be identified through molecular assays as routinely performed at Clinical Laboratory Improvement Amendments (CLIA) of 1988 or other similarly certified laboratories.

Patients will undergo radiographic evaluation of their disease at the end of every third cycle, starting with the end of Cycle (C) 3 (i.e., on or up to seven days before C4 Day [D] 1). Patients will continue on DAY101 until radiographic evidence of disease progression by RANO criteria as determined by treating investigator, unacceptable toxicity, decision to enter a "drug holiday" period after completion of 26 cycles, patient withdrawal of consent, or death.

3.1.2. Arm 2: Low-Grade Glioma Extension

Arm 2 will enroll patients aged 6 months to 25 years, inclusive, with recurrent or progressive low-grade glioma harboring a known or expected to be activating RAF alteration (e.g., BRAF or CRAF/RAF1 fusion or BRAF V600 mutations). Patients with RAF alterations will be identified through molecular assays as routinely performed at CLIA-certified or other similarly certified laboratories.

Once Arm 1 is approximately 90% enrolled (i.e., ~54 patients), the DSMB will convene to review all available safety and efficacy data (at a regularly scheduled meeting or on an ad hoc basis, as appropriate). After reviewing the data, the DSMB will make a formal recommendation based on assessment of benefit/risk as to whether to open Arm 2 to enrollment after Arm 1 is fully accrued. Arm 2 will remain open to enrollment during regulatory review of a marketing application for DAY101. Should Arm 1 not meet its primary endpoint, Arm 2 will immediately close to further enrollment.

Patients enrolled in Arm 2 will undergo the same treatment and radiographic assessment schedule as Arm 1. Additional details can be found in the schedule of assessments (Appendix A).

3.1.3. Arm 3: Advanced Solid Tumor

Arm 3 will enroll patients aged 6 months to 25 years, inclusive, with advanced solid tumors harboring a known or expected to be activating RAF fusion (e.g., BRAF or CRAF/RAF1 fusion). Patients with RAF fusions will be identified through molecular assays as routinely performed at CLIA-certified or other similarly certified laboratories.

For this portion of this study, a modified Simon 2-stage design will be used to enroll up to approximately 20 patients based on evaluation of ORR (confirmed PR or CR per RECIST v1.1) (Simon 1989). The statistical assumptions will require approximately 12 evaluable patients in the first stage and 20 evaluable patients in total. In the first stage, if ≤ 1 objective response (PR or CR per RECIST v1.1) is seen in the first 12 patients, the study will stop any further recruitment and the treatment will be considered as not effective in this setting; however, if ≥ 2 responses occur, an additional eight patients will be recruited.

Patients will undergo radiographic evaluation of their disease at the end of every second cycle, starting with the end of C2 (i.e., on or up to seven days before C3D1), and at the end of every two cycles through 12 months of treatment (i.e., on or up to seven days before C5D1, C7D1, etc.). Thereafter, patients will undergo radiographic evaluation at the end of every three cycles. Patients will continue DAY101 until radiographic evidence of disease progression by RECIST v1.1 criteria as determined by treating investigator, unacceptable toxicity, decision to enter a “drug holiday” period after completion of 26 cycles, patient withdrawal of consent, or death.

3.1.4. End of Study and Length of Study

The end of this study is defined as the date when the last visit occurs for the last patient enrolled. The end of the study is expected to occur 25 months after the last patient is enrolled into Arm 1.

In addition, the Sponsor may decide to terminate the study at any time.

The total length of the study, from screening of the first patient to the end of the study, is expected to be approximately four years.

3.1.5. Number of Patients

Approximately 140 patients in total will be enrolled across all treatment arms of this study. The arms will be enrolled as follows:

- Arm 1: Approximately 60 patients
- Arm 2: Up to 60 patients
- Arm 3: Up to 20 patients

Patients will be considered as enrolled when they have ingested a dose of DAY101 on C1D1.

3.2. Investigational Sites

Approximately 40 institutions will participate in this study.

4. SELECTION OF STUDY POPULATION

Potential patients and/or their parents must sign an informed consent form (ICF) and pediatric assent form, where applicable, before any study-specific screening tests may be conducted.

4.1. Inclusion Criteria

Patients must meet all of the following criteria to be eligible for enrollment in the study:

1. Patients must be age 6 months to 25 years, inclusive, with:
    a) Arm 1 (Low-Grade Glioma): A relapsed or progressive low-grade glioma with a documented known activating BRAF alteration, as identified through molecular assays as routinely performed at CLIA or other similarly certified laboratories
    b) Arm 2 (Low-Grade Glioma Extension): A relapsed or progressive low-grade glioma with a documented known or expected to be activating BRAF mutation or RAF fusion, as identified through molecular assays as routinely performed at CLIA-certified or other similarly certified laboratories
    c) Arm 3 (Advanced Solid Tumor): Locally advanced or metastatic solid tumor with a documented known or expected to be activating RAF fusion, as identified through molecular assays as routinely performed at CLIA-certified or other similarly certified laboratories, that has relapsed or progressed or was nonresponsive to available therapies and for which no standard or available systemic curative therapy exists
2. Patients must have histopathologic verification of malignancy at either original diagnosis or relapse.
3. Patients must have received at least one line of prior systemic therapy and have documented evidence of radiographic progression.
4. Patients must have evaluable and/or measurable disease (imaging must be performed within 28 days of the initiation of treatment) as specified below:
    a) Arm 1 (Low-Grade Glioma): Must have at least one measurable lesion as defined by RANO criteria (T1-weighted lesion that can be reproducibly measured in at

least 2 dimensions of at least 10 mm, visible on 2 or more axial slices that are preferably, at most, 5 mm apart with 0-mm skip)

   b) Arm 2 (Low-Grade Glioma Extension): Must have evaluable (either unidimensionally measurable lesions, masses with margins not clearly defined, or lesions with maximal perpendicular diameters less than 10 mm) and/or measurable disease as defined by RANO criteria
   c) Arm 3 (Advanced Solid Tumor): Must have at least one measurable lesion as defined by RECIST v1.1 (≥ 10 mm by CT/MRI scan [slice thickness ≤ 5 mm], ≥ 20 mm by chest X-ray, or ≥ 10 mm with calipers by clinical exam, or pathologic lymph nodes with a short axis of ≥ 15 mm by CT scan/MRI)

5. Radiation therapy to the measurable lesion(s) must be completed at least six months prior to administration of DAY101. Patients who have documented radiographic progression less than six months from radiotherapy in one or more measurable lesions are eligible.
6. Patients must have Karnofsky (those 16 years and older) or Lansky (those younger than 16 years) performance score of at least 50. Patients who are unable to walk because of paralysis, but who are able to sit in a wheelchair, will be considered ambulatory for the purpose of assessing the performance score.
7. Patients must have fully recovered from the acute toxic effects of all prior anticancer chemotherapy and have undergone the following washout periods, as applicable:
   a) Myelosuppressive chemotherapy: At least 21 days must have elapsed after the last dose of myelosuppressive chemotherapy (42 days if prior nitrosourea).
   b) Radiation therapy (XRT): At least 14 days must have elapsed after the last dose fraction of XRT.
   c) Stem cell transplant or adoptive cell therapy: At least 100 days must have elapsed after cell infusion.
   d) Investigational agent or any other anticancer therapy not defined above: At least four weeks prior to planned start of DAY101, or five half-lives, whichever is shorter.
8. Chronic toxicities from prior anticancer therapy must be stable and at Common Terminology Criteria for Adverse Events (CTCAE) version 5.0 Grade ≤ 2.
   a) Ongoing retinopathy must be ≤ Grade 1.
9. Patients must have fully recovered from any prior surgery.
10. An archival tumor tissue sample must be available. If an archival tumor tissue sample is not available, a fresh biopsy should be performed at baseline. For patients enrolling to Arm 2 (Low-Grade Glioma Extension) that do not have archival tumor tissue, enrollment may be considered on a case-by-case basis following a discussion between the investigator and the Day One Medical Monitor.
11. Patients must have adequate hematologic function, as defined by the following:
    a) Absolute neutrophil count ≥ $1000/mm^3$
    b) Platelet count ≥ 75.0×10^9/L (transfusions allowed per institutional guidelines; last transfusion > 2 weeks prior to C1D1)
    c) Hemoglobin ≥ 10.0 g/dL (transfusions allowed per institutional guidelines; last transfusion > 4 weeks prior to C1D1)

   d) Hematopoietic growth factors: At least 14 days after the last dose of a long-acting growth factor (e.g., Neulasta®) or seven days for short-acting growth factor

12. Patients must have adequate hepatic and renal function, defined by the following:
    a) Total bilirubin ≤ 1.5 × upper limit of normal (ULN) for age (patients with documented Gilbert's Disease may be enrolled with Sponsor approval and total bilirubin ≤ 2.0 × ULN)
    b) Serum glutamic-pyruvic transaminase (SGPT)/alanine aminotransferase (ALT) ≤ 2.5 × ULN
    c) Serum glutamic-oxaloacetic transaminase (SGOT)/aspartate transaminase (AST) ≤ 2.5 × ULN
    d) Serum creatinine within normal limits, or estimated glomerular filtration rate ≥ 60 mL/min/1.73 m^2 based on local institutional practice for determination
13. Thyroid function tests must be consistent with stable thyroid function. Patients on a stable dose of thyroid replacement therapy for a minimum of three weeks before starting DAY101 are eligible.
14. Patients must have left ventricular ejection fraction (LVEF) of ≥ 50% as measured by echocardiogram (ECHO) or multiple-gated acquisition (MUGA) scan, or fractional shortening (FS) ≥ 25% (Tissot et al., 2018) as measured by ECHO, within 28 days before the first dose of DAY101. If normal practice at the institution is to provide the LVEF result as a range of values, then the upper value of the range will be used to determine the result.
15. Patients receiving steroids for tumor-associated symptoms must be on a stable dose (e.g., no initial/loading dose, no increase or decrease) for 14 days prior to C1D1.
16. Patients must be able to comply with treatment, laboratory monitoring, and required clinic visits for the duration of study participation.
17. Male and female patients with reproductive potential must be willing to use a highly effective birth control method for the duration of treatment and for 180 days following the last dose of study drug. Highly effective birth control methods are described in Appendix K.
18. Patients must have ability to swallow tablets or liquid or be willing to comply with administration of a nasal or gastric tube for gastric access.
19. Parent/guardian of child or adolescent patient must have the ability to understand, agree to, and sign the study ICF and applicable pediatric assent form before initiation of any protocol-related procedures; patient must have the ability to give assent, as applicable, at the time of parental/guardian consent.

4.2. Exclusion Criteria

Patients meeting any of the following criteria are to be excluded from study participation:

1. Patient's tumor has an additional previously known or expected to be activating molecular alteration(s) (e.g., histone mutation, IDH1/2 mutations, FGFR mutations or fusions, MYBL alterations, NF-1 somatic or germline mutations).
2. Patient has symptoms of clinical progression without radiographically recurrent or radiographically progressive disease.

3. Patient has known or suspected diagnosis of neurofibromatosis type 1 (NF-1) via genetic testing or current diagnostic criteria.
4. Patient has history of any major disease, other than the primary malignancy under study, that might interfere with safe protocol participation.
5. Patient has a history or current evidence of central serous retinopathy (CSR), retinal vein occlusion (RVO), or ophthalmopathy present at baseline that would be considered a risk factor for CSR or RVO. Ophthalmological findings secondary to long-standing optic pathway glioma (such as visual loss, optic nerve pallor, or strabismus) will NOT be considered significant abnormalities for the purposes of this study.
6. Patient has major surgery within 14 days (two weeks) prior to C1D1 (does not include central venous access, cyst fenestration or cyst drainage, or ventriculoperitoneal shunt placement or revision).
7. Patient has clinically significant active cardiovascular disease, or history of myocardial infarction, or deep vein thrombosis/pulmonary embolism within six months prior to C1D1, ongoing cardiomyopathy, or current prolonged QT interval corrected for heart rate by Fridericia's formula (QTcF) interval > 470 milliseconds based on triplicate electrocardiogram (ECG) average.
8. Patient is currently enrolled in any other investigational treatment study. Participation in a concurrent observational or bio-sampling study is allowed.
9. Patient has active systemic bacterial, viral, or fungal infection.
10. Patient has nausea and vomiting ≥ National Cancer Institute (NCI) Common Terminology Criteria for Adverse Events (CTCAE) v5.0 Grade 2, malabsorption requiring supplementation, or significant bowel or stomach resection that would preclude adequate absorption of DAY101.
11. Patient is neurologically unstable despite adequate treatment (e.g., uncontrolled seizures).
12. Patient is currently being treated with a strong CYP2C8 inhibitor or inducer other than those allowed per Section 5.3.2. Medications that are substrates of CYP2C8 are allowed but should be used with caution.
13. Patient is pregnant or lactating.
14. Patient has a history of any drug reaction with eosinophilia and systemic symptoms (DRESS) syndrome or Stevens Johnsons syndrome (SJS), or hypersensitivity to the investigational medicinal product or to any drug with similar chemical structure or to any other excipient present in the pharmaceutical form of the investigational medicinal product.
15. There are other unspecified reasons that, in the opinion of the investigator, make the patient unsuitable for enrollment.

5. TREATMENT

5.1. Investigational Product

DAY101 for oral dosing is provided as an immediate-release tablet in one strength, 100 mg. The 100-mg tablets are red to yellowish red oval tablets and labeled DAY101. In addition, DAY101 is provided in bottles as a powder for reconstitution (430 mg per bottle to deliver

300-mg dose) that is reconstituted with water to form a suspension with a concentration of 25 mg/mL.

The site pharmacist will dispense the tablets. The powder for reconstitution will be dispensed to the patient in a kit containing a dosing syringe and bottle adaptor, or to the patient's parent or legal guardian, in an amount necessary to allow for outpatient administration. Further information is provided in the Pharmacy Manual.

DAY101 tablets are to be stored at controlled room temperature (between 15°C and 25°C) and protected from light. DAY101 powder for reconstitution is to be stored at controlled room temperature (between 15°C and 25°C) and protected from light. Additional details regarding the investigational product are provided in the DAY101 IB.

5.2. DAY101 Administration

5.2.1. General Dosing Instructions

Dosing for an individual should be at a consistent day and time (± 12 hours) each week. There is no restriction with regard to intake of food (patients can be fed or fasted). Tablets should not be cut or crushed. The powder for reconstitution will be reconstituted with water using the provided dosing syringe, and the resulting liquid suspension will be administered orally or enterally via a nasal or gastric feeding tube with a dosing syringe.

If the patient spits up a portion of the oral suspension, the patient or parent or legal guardian should be instructed to call the study doctor or nurse to discuss if they should take any additional suspension. If after two attempts the patient does not swallow the dose, it will be considered a "missed" dose. Record the missed dose in the diary as "missed" and note next to the entry that the patient "spit up the dose." If the patient swallows the oral suspension and vomits after ingesting it, another dose should not be given at that time.

If the patient vomits the tablets and they are still recognizable, then the patient can be redosed. If the patient vomits within 30 minutes of ingesting tablets and the tablets are not recognizable, the patient or parent or legal guardian should be instructed to call the investigator or study nurse to discuss whether the patient should take any additional tablets. If it has been longer than 30 minutes or the investigator or study nurse is not reachable, the patient should not be redosed.

The patient, or the patient's parent or legal guardian, will complete a study drug administration diary to record dosing compliance. The study drug administration diary will be reviewed at each clinic visit by the investigator or study nurse.

Late doses (i.e., between 12 and 72 hours of regularly scheduled dose) should be noted in the diary. Doses that are late by more than 72 hours (three days) should be skipped and recorded in the dosing diary as missed. A minimum of four days should occur between doses. Any dose that cannot be given within three days of the scheduled dose should be recorded as

missed. Dose rounding guidelines for tablets are provided in Table 7. Dose rounding guidelines for the reconstituted liquid suspension are provided in Table 8.

All patients will be initiated on DAY101 at 420 mg/m^2 according to the patient's baseline BSA. Patients with a BSA of 0.6m^2 or less will be required to receive the liquid suspension formulation. If appropriate in the opinion of the investigator, patients may change formulations at any point during treatment (see Section 6.8 for PK sampling instructions).

Table 7: Dose Rounding Guidelines for DAY101 Tablets (100 mg)

BSA (m^2)	Second Dose Reduction (mg)	First Dose Reduction (mg)	Starting Dose 420 mg/m^2 (mg)
0.3	Liquid formulation required; please see Table 8.		
0.4			
0.5			
0.6			
0.7	100	200	300
0.8	100	200	300
0.9	200	300	400
1.0	200	300	400
1.1	300	400	500
1.2	300	400	500
1.3	400	500	600
1.4	400	500	600
1.5	400	500	600
1.6	400	500	600
1.7	400	500	600
1.8	400	500	600
≥ 1.9	400	500	600

Abbreviations: BSA, body surface area.

Table 8: Dose and Volume Rounding Guidelines for DAY101 Reconstituted Liquid Suspension (25 mg/mL)

BSA (m^2)	Second Dose Reduction	First Dose Reduction	Starting Dose 420 mg/m^2
	Suspension Volume (mL)	Suspension Volume (mL)	Suspension Volume (mL)
0.3	3	4	5
0.4	4	5	7
0.5	5	7	8
0.6	6	8	10
0.7	8	10	12
0.8	9	11	13
0.9	10	12	15
1.0	11	14	17
1.1	12	15	18
1.2	13	17	20
1.3	15	18	22
1.4	16	20	24
1.5	16	20	24
1.6	16	20	24
1.7	16	20	24
1.8	16	20	24
≥ 1.9	16	20	24

Abbreviations: BSA, body surface area.

Any questions regarding dose rounding should be directed to the Medical Monitor.

Body surface area should be calculated on D1 of each cycle to confirm dose. The dose should be increased or decreased for any change in BSA greater than 10% on D1 of any cycle. The maximum dose will be no higher than the adult RP2D of 600 mg QW, established in the adult Phase 2 trial (C28001), regardless of the patient's BSA.

Refer to the pharmacy manual for additional dosing details.

5.2.2. Dose Modifications

National Cancer Institute (NCI) CTCAE version 5.0 should be used when evaluating unacceptable toxicities. According to the CTCAE, adverse reactions are reported by grade (level of severity) on a scale of Grades 1 to 5. Generally, the descriptions for adverse reactions by grade level (scale of Grades 1 to 5) follow the guidelines outlined below in Table 15.

The dose of DAY101 may be reduced at the discretion of the investigator for an AE that is clinically or medically intolerable. Patients who experience an AE that is clinically or medically intolerable, or otherwise described in Section 5.2.3, should have DAY101 dosing interrupted until resolution to Grade 1 or baseline level. Patients should be reevaluated at least weekly for resolution of the AE. Upon resolution, dosing may be restarted at the same dose or reduced to a lower dose of DAY101 at the discretion of the investigator (refer to Table 7 and Table 8). If the dose is reduced, re-escalation to a higher dose may be permitted after approval by the Sponsor.

All patients who experience drug-related toxicity requiring a recovery period longer than 28 days will be withdrawn from study drug administration unless there is evidence of benefit and no alternative treatment available as determined by the investigator and approved by the Sponsor.

5.2.3. Management of Treatment-Related Events

Included below are toxicity management guidelines. Modification of DAY101 dose is outlined in Section 5.2.2.

Table 9: Treatment-Emergent Adverse Events Requiring Dose Interruption Until Resolution to ≤ Grade 1 or Baseline

Drug-Related Toxicity	Dose Hold Criteria
Hematologic	CTCAE Grade 4 anemia
	CTCAE Grade 4 thrombocytopenia or<br>CTCAE Grade 3 thrombocytopenia with Grade 1 or higher bleeding
	CTCAE Grade 4 neutropenia
Vomiting	CTCAE Grade 3 or 4 only if it persists for > 72 hours despite standard-of-care treatment<br>Note: Admission to the hospital for patients < 1 year of age with Grade 3 or 4 vomiting will be required until these events resolve to Grade 1.
Diarrhea	CTCAE Grade 3 or 4 only if it persists for > 72 hours despite standard-of-care treatment<br>Note: Admission to the hospital for patients < 1 year of age with Grade 3 or 4 diarrhea will be required until these events resolve to Grade 1.
Rash, HFSR, or photosensitivity	CTCAE Grade 3 lasting > 8 consecutive days despite skin toxicity treatment, as per local practice, or<br>CTCAE Grade 4

Drug-Related Toxicity	Dose Hold Criteria
Retinal disorder<br>(e.g., retinal detachment/tear, retinal vascular disorder, retinopathy)	CTCAE Grade ≥ 2 confirmed by ophthalmologic examination
Visual disturbances without ocular (retinal) changes	CTCAE Grade ≥ 3
Other events	CTCAE Grade ≥ 3, except for the exceptions noted below
Exceptions to dose hold criteria	CTCAE Grade 3 fatigue only if it persists for < 5 days<br>CTCAE Grade 3 edema only if it persists for < 5 days<br>Asymptomatic CTCAE Grade 3 laboratory abnormalities

Abbreviations: CTCAE, Common Terminology Criteria for Adverse Events; HFSR, hand-foot skin reaction.

The toxicities in Sections 5.2.3.1 through 5.2.3.5 have been observed with DAY101 administration or have been observed with other therapies that have a similar mechanism of action.

5.2.3.1. Ocular Disturbances

An eye examination will be performed by an ophthalmologist if visual abnormalities are described by the patient. Careful monitoring of eye complaints should be followed, and the eye examination should include the clinically indicated assessments (e.g., slit-lamp examination of the cornea, retinal photographs, and intraocular pressure measurements). Patients must be instructed to report visual symptoms as soon as they occur. Early and aggressive dose interruption of mild visual symptoms may avoid more serious ocular complications.

5.2.3.2. Photosensitivity

Photosensitivity is a recognized class effect of RAF kinase inhibitors. Patients should avoid excess exposure to sunlight and UV light and use broad-spectrum sunscreen (e.g., containing titanium dioxide or zinc oxide) with a skin protection factor (SPF) ≥ 30. Patients should be instructed to monitor for signs and symptoms of photosensitivity (e.g., itchy eruptions or areas of redness and inflammation on patches of sun-exposed skin).

5.2.3.3. Rashes

Rashes (maculopapular, dermatitis acneiform, and pruritis) have been observed with DAY101 administration. Patients should be referred to a dermatology department when symptoms are impairing function (e.g., cannot sleep, sit still) or psychosocially bothersome, and when management techniques fail to resolve.

Should a rash occur, see Appendix H for management options.

5.2.3.4. Increased Creatine Phosphokinase

While an asymptomatic increase in CPK level alone is not reason to reduce the dose of DAY101, it is important to rule out an accompanying clinical condition. See the recommendations in Table 10.

Table 10: Increased Creatine Phosphokinase Recommendations

AE Grade	Event Definition	Recommendation
Grade 1	> ULN – 2.5 × ULN	• Rule out increased physical activity, trauma, falls, muscle injury<br>• Rule out concomitant use of statins or excessive environmental and other causes (e.g., alcohol, drugs, toxins, heat illness, seizures, etc.)<br>• Adequate hydration is recommended to maintain fluid and electrolyte balance and tissue perfusion
Grade 2	> 2.5 × ULN – 5 × ULN	• Rule out increased physical activity, trauma, falls, muscle injury<br>• Rule out concomitant use of statins or excessive environmental or other causes (e.g., alcohol, drugs, toxins, heat illness, seizures, etc.)<br>• BUN, creatinine, urinalysis<br>• Myoglobin test in urine (urine dipstick, heme +, RBC −)
Grade 3/ Grade 4	> 5 × ULN – 10 × ULN<br>> 10 × ULN	• Rule out increased physical activity, trauma, falls, muscle injury<br>• Rule out concomitant use of statins or excessive environmental or other causes (e.g., alcohol, drugs, toxins, heat illness, seizures, etc.)<br>• BUN, creatinine, urinalysis<br>• Myoglobin test in urine (urine dipstick, heme +, RBC −)<br>• In the presence of chest pain, test levels of troponin I or troponin T

Abbreviations: AE, adverse event; BUN, blood urea nitrogen; RBC, red blood cell; ULN, upper limit of normal.

Source: Common Terminology Criteria for Adverse Events, version 5.0.

5.2.3.5. Increased Liver Transaminases

Per the DAY101 IB, Grade 3 or higher elevations in aspartate aminotransferase (AST) have been observed in up to 5% of patients enrolled in Study C28001 with baseline AST/ALT elevations < 2.5 × ULN at study entry. This treatment plan allows a baseline AST/ALT elevation of up to 2.5 × ULN at enrollment. Should an increase in AST/ALT be observed while receiving DAY101, the recommendations from Table 11 should be followed.

Table 11: Increased Liver Transaminases Recommendations

Event Definition	Recommendation
Asymptomatic, absolute increase in AST or ALT to > 5 × ULN but ≤ 2 × increase from baseline	• Do not modify the dose of DAY101
Asymptomatic ≥ Grade 3 AST or ALT AND > 2 × increase from baseline	• Withhold DAY101 until the laboratory abnormality resolves to < 5 × ULN or baseline<br>• The patient may resume taking the original dose of DAY101 if the AST or ALT abnormality resolves within 8 days.<br>• Patients whose resolution of AST or ALT abnormality is taking longer than 8 days may have their DAY101 dose decreased by 1 dose level to the next lower dose (once weekly).

Abbreviations: ALT, alanine aminotransferase; AST, aspartate aminotransferase; ULN, upper limit of normal.

5.3. Prior and Concomitant Medications

5.3.1. General

All medications that were used from 28 days prior to enrollment through the end of study participation will be recorded in the electronic case report form (eCRF). All prior anticancer treatments, regardless of when they were administered, will also be collected. These are to include prescription medications and transfusions. Excluded prior medications are those excluded via the eligibility criteria (Section 4.2).

5.3.2. Allowed Concomitant Medications

The investigator should instruct the patient or the patient's parent or legal guardian to notify the study site about any new medications he/she takes after starting study drug.

Patients taking medication chronically should be maintained on the same dose and schedule throughout the study period, as medically feasible. The days of full PK blood sampling should be representative of the other study days with regard to the use of the chronically administered concomitant medications. However, if a concomitant medication is used intermittently during the study, this medication should be avoided on the days of full PK sampling, if medically feasible.

In general, the use of any concomitant medication/therapies deemed necessary for the care of the patient is permitted, except as specifically prohibited in Section 5.3.3. Please refer to the DAY101 IB for possible interactions with other drugs. Medications required to treat AEs and manage cancer symptoms and concurrent stable diseases, and supportive care agents, such as pain medications, antiemetics, short courses of steroids, and antidiarrheals, are allowed. Oral contraceptive pills are permitted.

As a general precaution, patients receiving concomitant medications, particularly those with narrow therapeutic indices, should be carefully monitored, as potential drug-drug interactions between DAY101 and other drugs have not been studied in humans. Patients should also be instructed to consult with the investigator before taking any new medications, including over-the-counter products.

Routine vaccinations, including the COVID-19 vaccine, should be administered per local institutional guidelines.

5.3.3. Prohibited Concomitant Medications

As DAY101 is a substrate of CYP2C8, patients should not take strong inhibitors or inducers of CYP2C8 (refer to Appendix C), as they could alter the drug's PK. Medications that are substrates of CYP2C8 are allowed, but should be used with caution. Medications that are substrates of breast cancer resistance protein (BCRP) are prohibited during this study. During this study, patients are not allowed to receive other chemotherapeutic agents (traditional chemotherapy, targeted agents, monoclonal antibodies, etc.), drugs with immunosuppressant properties (other than steroids as allowed per Section 5.3.2), herbal supplements, or any other investigational agents besides DAY101.

5.3.4. Concurrent Surgery

Patients may undergo surgical intervention and remain on study when, in the opinion of the treating investigator, surgery is indicated for anything other than tumor resection for clinical or radiographic disease progression (e.g., cyst decompression, cerebrospinal fluid shunt revision). Therapy should be held for six days before intervention. Patients undergoing surgical intervention are permitted to restart study treatment six days following surgical intervention after discussion with and approval by the Sponsor. The surgical intervention should be recorded on the appropriate eCRF. For patients requiring surgical intervention due to clinical or radiographic evidence of tumor progression, patients should be withdrawn from study.

If surgery is clinically indicated while on study, the Sponsor will request a tumor specimen and a concurrent PK sample. A separate tissue collection ICF will be provided. Please see details regarding surgical sample collection in Section 6.5.

Arm 3 (Advanced Solid Tumor) only: For patients who receive DAY101 therapy prior to attempted surgical resection, DAY101 should be continued until a tumor is deemed to be resectable by the investigator. While timing of surgery is not protocol specified, ideally surgery should occur within three months of best radiographic response. If the resection surgery results in negative margins and study treatment is stopped, disease assessments, as described in Appendix A, should be performed every three months following the last dose of study medication. Patients whose treatment is stopped for this reason are permitted to restart study treatment if they experience disease recurrence only after discussion with the Medical Monitor. Patients who restart therapy will undergo assessments as if they were on study as outlined in Appendix A.

Patients who receive DAY101 and go on to an attempt at surgical resection will be re-staged at the time of surgery. Staging will be defined according to the tumor, node, metastasis (TNM) classification system: T1 (localized to the organ or tissue of origin) or T2 (extending beyond the tissue or organ of origin) according to the invasion of contiguous organs; N0 or N1 and M0 or M1 according to the presence of lymph node or distant metastasis. Lymph node involvement should be evaluated clinically or by radiographic imaging and confirmed, when necessary, by cytological or histological biopsy.

The status of resection margins will be classified according to the UICC-R classification and the Intergroup Rhadbomyosarcoma Staging (IRS) systems, which is generally used for primary surgery in pediatric rhabdomyosarcomas. UICC-R R0 or IRS Group I will correspond to complete tumor resection with histologically free margins; UICC-R R1 or IRS II will correspond to macroscopic resection but invaded margins on histology; UICC-R R2 or IRS III correspond to macroscopic residual tumor after surgery (III B) or biopsy (III A). IRS IV corresponds to distant metastatic tumor.

Patients who undergo surgical resection for local control may continue to receive DAY101 after surgical recovery and discussion between the investigator and the Sponsor.

5.3.5. Palliative Radiotherapy

In general, measurable lesions being used to measure response should not be irradiated without discussion with the Sponsor, and may be reason for the patient to be removed from study for progressive disease. Irradiated non-measurable lesions will be considered not evaluable for response, but still can be used to assess progressive disease. The intensities, number, and dates of doses received for allowed palliative radiotherapy should be recorded on the appropriate eCRF.

Palliative radiotherapy to specific sites of disease is permitted if considered medically necessary by the treating physician. DAY101 should be held for six days before intervention. These patients are permitted to restart study treatment six days following radiotherapy after discussion with and approval by the Sponsor.

5.4. Removal of Patients from Therapy or Assessment

Patients will be advised that they are free to withdraw from the study at any time. Patients who withdraw from therapy but do not withdraw consent will be followed for long-term follow-up. Over the course of the study, the investigator and/or the Sponsor may withdraw a patient from treatment for any of the reasons listed below:

- Disease progression as assessed by the investigator using RANO (Arms 1 and 2: Low-Grade Glioma) or RECIST v1.1 (Arm 3: Advanced Solid Tumor) criteria, as applicable. Patients may continue treatment beyond progression if, in the opinion of the investigator and with permission of the Sponsor, the patient is deriving clinical benefit from continued study treatment.
- Unacceptable toxicity that does not improve after dose reduction(s)

- Intercurrent illness compromising the ability to fulfill protocol requirements
- Dosing interruption > 28 days, unless resumption of treatment approved by the Sponsor
- Requirement for alternative treatment in the opinion of the investigator
- Significant noncompliance to protocol
- Pregnancy
- Withdrawal of consent or assent by the patient or the patient's parent or legal guardian
- Loss to follow-up
- Death

When a patient withdraws from treatment, the patient should undergo the EOT visit, as outlined in Section 6.14.

6. TESTS AND EVALUATIONS

All required observations and schedules are described in more detail in Sections 6.1 through 6.17, and summarized in Appendix A.

Most study assessments and procedures (including treatment administration) will be performed in cycles, defined as 28-day intervals starting with the date of DAY101 administration at baseline. Unless otherwise indicated, in the absence of toxicity, all scheduled visits will occur within windows for the protocol-specified visit schedule. If the patient is unable to have a study assessment within the defined time window due to an event outside of his or her control (e.g., clinic closure or travel restrictions due to COVID-19, personal/family emergency, inclement weather), the assessment should be performed as close as possible to the required schedule.

6.1. Screening

The screening procedures must be conducted within 28 days prior to C1D1, unless otherwise noted. Patients who cannot complete the procedures within the screening window may be rescreened as many times as the investigator deems appropriate. Procedures that were obtained as part of the patient's standard care prior to providing informed consent for this study do not need to be repeated, with the exception of tumor imaging, which must be repeated if the prior imaging does not collect the appropriate sequences required for study as defined in the Imaging Manual. All patients receiving protocol therapy will have historical scans from the 12-month period immediately prior to the eligibility scan (if available) transferred to the central imaging laboratory as specified in the Imaging Manual.

Screening tests that are completed in the 48 hours prior to start of DAY101 and duplicate C1D1 tests according to the schedule of assessments (Appendix A) will be accepted as

fulfilling the C1D1 assessments. Repeat testing on C1D1 is not necessary for this scenario, and results should be recorded in the screening set of eCRFs.

A representative from the investigational site will enter demographic data for potential study candidates into the interactive response technology (IRT) system. The IRT system will assign potential study candidates a unique patient identification number. The patient identification number will be entered onto an eligibility form. A completed eligibility form will be sent to the Sponsor or designee when a potential study candidate is identified. Any reports (e.g., pathology report, radiology report) supportive to the eligibility form must have Protected Health Information (name, date of birth, address, parents' names, medical record number, etc.) removed prior to submission to Sponsor. The site representative will be contacted by either the Sponsor or its designee to 1) confirm eligibility, or 2) provide additional information.

Patients who are determined to be screen failures can be rescreened. Rescreened patients will maintain the same unique patient number assigned to them originally by the IRT system. Site representatives should not enter revised screening data into the IRT, as a new patient identification number need not be assigned. A revised eligibility form should be submitted to the Sponsor or its designee for rescreened patients. See the Study binder for eligibility form, patient number assignment, contact numbers, and other details of enrollment.

The approved eligibility form will be returned to the site with the cohort assignment (if applicable) as verification of eligibility in the trial, and for record-keeping purposes.

6.2. Enrollment

Enrollment procedures will be conducted in accordance with the schedule of assessments (Appendix A). Patients who meet the eligibility criteria outlined in Section 4.1 will be enrolled in this study.

6.3. Procedure Details

Timing of assessments and further details of assessments can be found in the Appendices.

6.4. Medical History and Malignancy History

Medical, surgical, and malignancy history, including histologic confirmation of tumor, primary tumor diagnosis and recurrence dates, prior treatments, treatment dates, tumor responses, etc., should be recorded on the appropriate eCRF. Demographics, including age, sex, race, and ethnicity, will be recorded.

Arm 3 (Advanced Solid Tumor) only: In addition to the histories described above, patients who receive DAY101 and go on to surgery will be staged by conventional TNM classification at study entry and again at the time of en bloc surgical resection. As part of screening procedures, the investigator will collect in the electronic data capture (EDC) system the patient's treatment plan, including surgical procedure and anticipated outcomes

(amputation, functional deficit, etc.) had they not enrolled in this study. The surgical path specimen will be collected and submitted for central pathology review.

6.5. Archival Tumor

Archival tumor samples representing current disease status are required for all patients during screening. (Patients enrolling to Arm 2 [Low-Grade Glioma Extension] who do not have archival tissue may be considered for enrollment on a case-by-case basis after discussion between the investigator and the Day One Medical Monitor.) Archival tumor tissue may come from a specimen obtained at either diagnosis or recurrence. Tumor samples may be either formalin-fixed, paraffin-embedded (FFPE) tissue or fresh frozen tissue. If an archival tumor tissue sample is not available, a fresh biopsy should be performed at baseline. Tissue collection on-study or at the EOT visit is optional.

An optional tumor tissue sample from patients who undergo on-study, tumor-directed surgery is requested.

6.6. Safety Assessments

6.6.1. Physical Examination

Physical examinations will also include review of systems (chest, extremities, genitourinary, head/ears/eyes/nose/throat, lymph nodes, musculoskeletal, pulmonary, and skin) during screening. Symptom-directed physical examination, including measurement of weight and height, may be performed at other time points after screening.

6.6.2. Karnofsky or Lansky Performance Status Scales

The Karnofsky score should be assessed for those aged 16 years or older, and the Lansky score should be assessed for those younger than 16 years (refer to Appendix B).

6.6.3. Vital Signs

Vital signs should include the following:

- Systolic and diastolic blood pressure (BP) (For more information on normal ranges of BP by height and weight refer to Appendix G.)
- Heart rate, respiratory rate, body temperature (to be collected in an age-appropriate fashion, i.e., orally, axillary, auricularly, or rectally)
- Height and weight (to be measured consistently without shoes using the same instruments)

6.6.4. Ophthalmology Examination

Arms 1, 2, and 3 (only patients with an optic pathway glioma [OPG] or an underlying visual deficit related to the primary malignancy): Ophthalmology examinations will be performed at baseline, at the time of radiographic assessment, and at the EOT visit as described in the schedule of assessments (Appendix A). The examinations will be performed

by an ophthalmologist or other qualified site clinical personnel (Appendix I). The examinations should include the following:

- A slit-lamp examination, specifically looking for corneal/lens abnormalities
- A fundus examination with comment on retinal abnormalities
- Visual fields to confrontation
- Optic disc appearance
- Best corrected visual acuity (BCVA) (see Section 6.9.1.2)

Arms 1, 2, and 3 (patients without an OPG or an underlying visual deficit related to the primary malignancy): A baseline ophthalmology examination will be performed during screening by an ophthalmologist or other qualified site clinical personnel. The examination should include the following:

- A fundus examination with comment on retinal abnormalities
- BCVA (Section 6.9.1.2)

Symptom-directed examinations may be completed as needed at subsequent visits. Consultation with an ophthalmologist is required if abnormal ocular AEs arise as specified in Section 5.2.3.1.

6.6.5. Dermatologic Examination

A baseline dermatologic examination of the entire skin should be performed by the investigator and/or a dermatologist. The initial examination by the investigator/dermatologist should include a complete dermatological history of prior medications and cutaneous squamous cell carcinoma (SCC) risk factors (i.e., radiation therapy, sun exposure, immunosuppression, prior SCC, use of tanning beds, precursor lesions, and photochemotherapy for psoriasis). Skin lesions present at baseline should be recorded in medical history and only recorded as an AE if worsening of the lesion is observed.

Subsequent examinations should be symptom-directed and as clinically indicated, and patients should be assessed by the investigator and/or a consulting dermatologist for skin lesions, especially for keratoacanthomas and squamous cell carcinomas.

For each skin lesion observed, the dimensions and location on the body will be recorded. Existing lesions will be monitored throughout the study, and changes to the lesions should be recorded in the eCRF. Lesions developing during therapy that are suspected keratoacanthomas or squamous cell carcinomas will be biopsied and adequately treated; other lesions may be biopsied per the discretion of the investigator/dermatologist.

6.6.6. Neurologic Examination

A baseline complete neurologic examination should be performed by the investigator and/or a neurologist. Symptom-directed neurologic examinations may be completed at subsequent visits.

6.6.7. Assessment of Change in Pubertal Development

For all arms, in patients > 8 years of age, pubertal stage will be measured according to the Tanner Stages before the start of treatment (screening) and yearly thereafter (Marshall and Tanner 1969, Marshall and Tanner 1970). For female patients, age at menarche will be recorded. Assessments will occur while on active treatment and during long-term follow-up through sexual maturity.

6.6.8. Cardiac Function

6.6.8.1. Electrocardiograms

Twelve-lead resting ECGs will be performed in triplicate at baseline, with PK blood draws (per Table 12), and at the EOT visit. **Note**: there are multiple ECGs performed on C1D1, predose and corresponding with each PK blood draw.

To minimize variability, it is important that patients be in a resting position for at least 5 minutes prior to each ECG evaluation. There should be no more than 5 minutes between ECGs. Body position should be consistently maintained for each ECG to prevent changes in heart rate. ECGs for each patient should be obtained from the same machine whenever possible. When ECGs coincide with PK draw days, ECGs should be performed after dosing but before the PK blood draw. Any clinically significant changes in ECGs that occur during the study should be reported as AEs in the eCRF.

6.6.8.2. Echocardiogram/Multiple-Gated Acquisition

Echocardiogram or MUGA will be performed per normal site protocol. The choice of ECHO or MUGA is left up to the investigator, but the same technique should be used for each patient for each evaluation period. Any clinically significant changes in ECHO/MUGA that occur during the study should be reported as AEs in the eCRF.

6.7. Laboratory Tests

Routine laboratory tests, e.g., hematology and serum chemistries, will be performed locally. Special assessments such as PK will be performed centrally or as individually indicated. Additional information on the handling and processing of PK samples is provided in the separate Laboratory Manual.

6.7.1. Pregnancy Test

Urine or serum pregnancy tests are required for female patients of childbearing potential (surgically sterilized female patients or those who have not experienced menses for at least two years are not required to be tested). If any urine pregnancy test is positive, study treatment will be delayed until the patient pregnancy status is confirmed by a serum pregnancy test. If the serum pregnancy test is positive, the patient will be permanently discontinued from study treatment. Pregnancy reporting information is provided in Section 8.1.5.

6.7.2. Hematology

Hematology should include assessment of the following: hemoglobin, hematocrit, red blood cell count, white blood cell count with differential (neutrophils [count and percentage], lymphocytes, monocytes, eosinophils, and basophils [percentage]), and platelet count.

6.7.3. Serum Chemistries

Serum chemistries (nonfasting) should include assessment of the following: alkaline phosphatase, albumin, ALT, AST, CPK, blood urea nitrogen/urea, creatinine, glucose, lactate dehydrogenase, total bilirubin, total protein, sodium, potassium, chloride, phosphorus, and bicarbonate.

6.7.4. Thyroid Function Tests

For Arms 1 and 2 (Arm 3 required only if clinically indicated [e.g., in patients with thyroid cancer]), thyroid function tests should include assessment of the following: thyroid stimulating hormone (TSH), triiodothyronine (T3), and tetraiodothyronine (T4). Either free or total T3/T4 may be assessed based on local practice.

6.8. Pharmacokinetic Samples: Blood

The dose of DAY101 should be taken in the clinic on PK sampling days to ensure that a PK sample is collected as scheduled. Pharmacokinetic samples will be collected and handled as outlined in the Laboratory Manual.

The blood sampling regimens for determining the PK of DAY101 is given in Table 12. Blood samples for DAY101 concentration measurements will be collected on all patients in the study. Exact dates and clock times of drug administration and actual PK blood draw should be recorded on the appropriate eCRF. If a patient experiences an AE that fits the criteria of an SAE as determined by the investigator, a blood sample should be collected, whenever possible, for measurement of drug concentrations at or around that time.

Table 12: Schedule of Blood Sample Collections for DAY101 Pharmacokinetics

Cycle	Day	Pharmacokinetics Timepoint
1	1	1, 2, and 4 hours postdose
1	15 [a]	A sample between 1 and 4 hours postdose
2	1 [a]	Within 1 hour predose
4	1 [a]	A sample between 1 and 4 hours postdose
Every subsequent third cycle through C13 (e.g., C4, C7, C10, and C13)	1 [a]	A sample between 1 and 4 hours postdose
At the time of toxicity and/or at time of surgery, if clinically indicated	Unscheduled	Random level
At the time of a change in formulation (e.g., tablets to liquid, or vice versa) [b]	On Day 1 of the next cycle following the formulation change	Predose, 1, 2, and 4 hours postdose

Abbreviation: C, Cycle.
[a] ± 3-day window.
[b] ECGs are not required for this assessment.

6.9. Disease Assessment

6.9.1. Tumor Measurements

Arms 1 and 2 (Low-Grade Glioma): Disease assessment must be conducted in accordance with the Imaging Manual. Tumors will be assessed by radiographic tumor measurements using MRI of the brain and spine (as indicated).

Investigators must conform to the MRI acquisition requirements specified in the Imaging Manual consistently for all patients throughout the study. Disease assessments will use RANO criteria.

Arm 3 (Advanced Solid Tumor): Disease assessment will be conducted using disease-appropriate standard-of-care imaging modalities (MRI, CT) of chest, abdomen, pelvis, and any other areas with suspected disease involvement, as indicated. Investigators must conform to the acquisition requirements specified in the Imaging Manual consistently for all patients throughout the study. All patients will have imaging of chest/abdomen/pelvis with any other areas of suspected disease involvement at baseline. Patients enrolled with a history of CNS metastases should additionally have a head CT or MRI scan performed at each tumor assessment. Investigators should use the same method consistently for an individual patient throughout the study. Assessments of both measurable and nonmeasurable disease will be made using RECIST v1.1. Patients with locally advanced soft tissue sarcoma should have an MRI or CT of the site of disease and a CT or X-ray of the chest, plus any other areas of disease involvement.

For any patients with locally advanced disease at baseline, follow-up images can be limited to areas with known disease from baseline images.

6.9.1.1. Collection of Radiographic Studies for Independent Review

Baseline screening radiographic studies and all subsequent radiographic studies will be collected and stored for independent radiological review. In addition, prior radiographic scans should be submitted if acquired up to one year prior to enrollment.

Identification of radiographic findings that may affect patient management or outcomes during the course of patient participation in the study are the responsibility of the treating physician. Independent radiology review for this trial is only used to assess the objectives of the study. Any unexpected medical findings identified during subsequent independent radiologic review will not be reported back to the investigator.

6.9.1.2. Visual Acuity Assessments

Arms 1, 2, and 3 (only patients with an OPG or an underlying visual deficit related to the primary malignancy): Patients who have an underlying visual function deficit related to the primary malignancy or OPG will have a visual acuity examination every time they have a radiographic disease assessment and at the EOT visit. These examinations can be performed by an ophthalmologist or other qualified site clinical personnel.

Age-specific functional visual acuity assessments should be performed, which may include, but are not limited to, Teller Acuity Cards® (TAC; all patients), HOTV, or other Early Treatment Diabetic Retinopathy Study (ETDRS) charts (in patients developmentally able to perform). Best corrected visual acuity and method of age-specific visual acuity testing should be recorded. If the BCVA data at a particular visit are felt to be unreliable due to poor cooperation, testing should be repeated in one to two weeks. Only the visit believed to have yielded the most reliable data should be reported. More details can be found in Appendix I.

Arms 1, 2, and 3 (patients without an OPG or an underlying visual deficit related to the primary malignancy): A baseline ophthalmology examination will be performed during screening by an ophthalmologist or other qualified site clinical personnel. Symptom-directed examinations may be completed as needed at subsequent visits. Best corrected visual acuity and method of age-specific visual acuity testing should be recorded.

6.9.1.3. Motor Function Evaluation Using the Vineland Motor Scale

Arms 1 and 2 (Low-Grade Glioma): The purpose of these assessments is to document change in motor function from baseline, as measured by the Vineland Adaptive Behavior Scales, Third Edition (Vineland-3) Motor Skills domain of the Comprehensive Parent Rating Form.

Parents or caregivers will be assisted by telephone in answering the parent/caregiver questionnaire by study personnel. The administration of the Vineland-3 is expected to take approximately 30 minutes to complete. It is important that the same parent who completes the baseline questionnaire also complete the follow-up questionnaire.

The Vineland-3 Motor Skills questionnaire will only be administered to patients with baseline motor function deficits enrolled in the U.S., and only by third-party (trained and centralized) personnel.

6.9.2. Quality of Life: Pediatric Quality of Life Inventory—Core, Cancer, and Patient-Reported Outcomes Measurement Information System

In patients 2 years of age or older, Health-Related Quality of Life will be assessed using the Pediatrics Quality of Life™—Core (PedsQL-Core), Pediatrics Quality of Life™—Cancer (PedsQL-Cancer), and Patient-Reported Outcomes Measurement Information System (PROMIS®) assessment for the patient or parent/caregiver.

PedsQL:

The PedsQL 4.0 Generic Core Scales and the PedsQL 3.0 Cancer Module will be used to assess treatment and disease impact on quality of life. These rating forms have multidimensional child *self-report* and *parent proxy report* scales to assess health-related quality of life (QOL) in children, adolescents, and young adults aged 2 to 25 years. It consists of a 23-item core measure of global QOL that has four subscales: physical functioning, emotional functioning, social functioning, and school functioning.

There are different forms for parents of patients aged 2 to 17 years (toddler: 2–4 years; young child: 5–7 years; child: 8–12 years; teen: 13–17 years) and parallel self-report forms for patients aged 5 to 25 years (young child: 5–7 years; child: 8–12 years; teen: 13–17 years; young adult: 18–25 years). It takes approximately 5 to 10 minutes to complete.

PROMIS:

The PROMIS Pediatric/Parent Proxy Profile 49 will be used to assess treatment and disease impact on overall health. This measure consists of seven 8-item short forms to assess mental health, physical health, and social health. The specific short forms are the following:

- Emotional Distress—Anxiety
- Emotional Distress—Depression
- Fatigue
- Pain—Interference
- Pain—Intensity
- Physical Function—Mobility
- Peer Relationships

Information about cognitive function will also be collected through pediatric cognitive supplement short form 7a. To include patients greater than 17 years of age, the PROMIS-57 for adults, as well as adult cognitive short form 8a, will be used.

The PROMIS® questionnaire will only be administered to English-speaking patients enrolled in the U.S., Australia, and U.K.

6.10. Telephone/Telemedicine Visit

The site will contact the patient or the patient's parent/legal guardian by telephone for telephonic/telemedicine visits to assess for tolerability, continuation of study drug, and whether the patient needs to return to clinic earlier than planned. If not tolerating the drug well, the patient should be seen at least every seven days to assess AEs, and the patient or the patient's parent/legal guardian should be contacted regularly to assess AE status.

6.11. Survival Status

Patients will be followed for survival status and subsequent anticancer therapies by telephone or any other method.

6.12. Study Drug Administration and Dosing Diary

Completion of the outpatient dosing diary will include recording of DAY101 dosing.

Special considerations: Patients who enter a "drug holiday" discontinuation period after Cycle 26 may be considered for resumption of study drug if there is disease recurrence. As such, these patients should continue radiographic and/or clinical assessment of disease

response using the same modality and frequency used during the treatment portion of the study. These response assessments should be collected and reported every three months. These patients remain on study and will not have an EOT visit until death, loss of follow-up, withdrawal of consent, initiation of a new anticancer therapies, or study termination. The EOT visit will only occur once per patient.

6.13. Concomitant Medication

All medications that were used from 28 days prior to enrollment through the end of study participation will be recorded in the eCRF.

6.14. End of Treatment Visit

End of treatment is defined as Day 28 (± 7 days) of the final cycle of treatment (or time of premature discontinuation of treatment if clinically feasible). The EOT assessments and procedures will be conducted in accordance with the schedule of assessments (Appendix A).

6.15. Safety Follow-Up Visit

Safety follow-up assessments may be performed as part of the EOT visit if the latter was performed at least 28 days after final dose of the last cycle. The safety follow-up visit may be conducted by telephone if patient is not able to return to clinic.

The status of unresolved AEs should be assessed at the safety follow-up visit.

6.16. Long-Term Follow-Up

After permanent early treatment discontinuation, long-term follow-up assessments will occur approximately every three months (± 1 month) until the patient withdraws consent for further participation, is lost to follow-up, has died, or the study is closed. Patients who discontinue treatment due to a reason other than disease progression will continue radiographic tumor assessments per the protocol.

For patients in long-term follow-up with radiographic evidence of disease progression (or those unable to continue tumor assessments), the site will contact the patient or the patient's parent/legal guardian for follow-up assessments that may include subsequent anticancer therapies and survival status. Long-term follow-up may be conducted by telephone. For any patient who is lost to follow-up, the study site will attempt to ascertain survival information via public database search. If survival status still cannot be ascertained, patients will be considered lost to follow-up and will be censored appropriately. The first long-term follow-up assessment will occur three months (± 1 month) after the EOT visit.

6.17. Force Majeure Flexibility

With the outbreak of severe acute respiratory syndrome coronavirus 2 (SARS-CoV-2) and the impact of coronavirus disease 2019 (COVID-19) on the health-care system, the Sponsor of this study recognizes the need for flexibility in ensuring access to therapy. This need has been clearly elucidated in the FDA Guidance on Conduct of Clinical Trials of Medical Products during COVID-19 Public Health Emergency (FDA-2020-D-1106) and the EMA

Guidance on the Management of Clinical Trials during the COVID-19 (Coronavirus) pandemic (Version 3, 28/04/2020). While every effort should be made to follow the timing of study visits and procedures as closely as possible, the Sponsor may introduce flexibility in the nature and timing of visits. Changes may include using home health-care visits, telemedicine visits, local imaging, and distribution of multiple cycles of investigational product (or shipping investigational product to a patient's residence).

If such flexibility needs to be implemented for the safe conduct of this study, the Sponsor will notify sites of what procedures may be delayed or provide flexibility in how data are collected. As potential impacts to the health-care system may not be uniform through all regions where the Sponsor is conducting this study, guidance will be informed by worldwide regulatory authority guidance and local competent and ethical authorities, and may vary from site to site. Changes to timing and procedures will be communicated by the Sponsor through an official memo and training.

Visits occurring on C1D1, C1D15, and C2D1 must occur within specified protocol window for all patients enrolled. Visits held in conjunction with a response assessment must also occur in person, within the specified window. At a minimum, height and weight, chemistry, hematology, liver function tests, CPK, AEs, concomitant medications, ECGs, and imaging studies need to be collected. If needed, the use of a local hospital laboratory or commercial laboratory (e.g., LabCorp, Quest, etc.), may be used, if results can be provided to the treating physician prior to the visit. Imaging at a qualified MRI facility closer to the patient's residence may be used following training by the imaging vendor, with all imaging data submitted to the Sponsor. Following the C2D1 visit, windows around study visits and assessments may be expanded up to ± 7 days. For patients with a confirmed RANO response without clinical signs of progressive disease, imaging may occur up to seven days after the planned cycle visit date.

7. PLANNED ANALYSES

The Statistical Analysis Plan provides specific details on the analytical methods and data displays.

7.1. Analysis Populations

The populations for analysis are defined in Table 13:

Table 13: Population Definitions

Population	Description
Evaluable	Evaluable for Efficacy (FAS): All patients enrolled in the study who received at least 1 dose of study treatment and meet the definition for Efficacy Analysis as outlined in Section 7.3.1<br>Evaluable for DOR: Patients in the FAS who have a best overall confirmed response of CR or PR
Safety	All patients enrolled in the study who received at least 1 dose of study treatment (SAS)

Abbreviations: CR, complete response; DOR, duration of response; FAS, Full Analysis Set; ICF, informed consent form; PR, partial response; SAS, Safety Analysis Set.

7.2. Determination of Sample Size

Arm 1 (Low-Grade Glioma): A sample size of 60 patients provides 88% power to reject the null ORR of 21%, assuming that the true underlying ORR of DAY101 is 40% based on a test at the 2-sided 0.05 level. A result of at least 20 out of 60 (0.33) will be statistically significant.

A Phase 2 study of single-agent vinblastine (Bouffet et al. 2012), the largest study published to date in patients with relapsed or refractory low-grade glioma, reported objective responses (CR + PR) in 11 of 48 patients (ORR: 23% [95% CI: 12–37%]). This response rate represents the anticipated RANO ORR applied to this dataset (CR + PR, but not minor responses).

Arm 2 (Low-Grade Glioma Extension): Up to approximately 60 patients may be enrolled. Arm 2 is not powered based on formal statistical hypothesis testing, but for practical considerations to allow patients with pediatric low-grade glioma to receive treatment with DAY101 after Arm 1 has fully accrued and the DSMB has recommended opening to enrollment.

Arm 3 (Advanced Solid Tumor): Up to approximately 20 patients may be enrolled to provide a preliminary evaluation of DAY101 in pediatric patients with advanced solid tumors. For this portion of this study, a modified Simon 2-stage design will be used to enroll up to approximately 20 patients based on evaluation of ORR (confirmed PR or CR per RECIST v1.1) (Simon 1989). The statistical assumptions will require approximately 12 evaluable patients in the first stage and 20 evaluable patients in total. In the first stage, if ≤ 1 objective response (PR or CR per RECIST v1.1) is seen in the first 12 patients, the study will stop any further recruitment and the treatment will be considered as not effective in this setting; however, if ≥ 2 objective responses occur, an additional eight patients will be recruited. The anticipated response rate is expected to be near zero in patients with relapsed or progressive advanced solid tumors that have progressed after standard-of-care therapy and harbor an activating driver alteration. However, for the purposes of sample size calculation, a null hypothesis (H_0) of 5% is assumed. A sample size of 20 patients provides 90% power to reject the null ORR of 5%, assuming that the true underlying ORR of DAY101 is 25% based

on a test at alpha 1-sided at 0.1. A result of at least three out of 20 will be statistically significant.

7.3. Statistical Methods

7.3.1. Efficacy Analyses

7.3.1.1. Arm 1: Low-Grade Glioma

The efficacy analyses will include all patients who received at least one dose of study drug and have measurable disease as determined by the IRC at baseline and have follow-up imaging or radiographically confirmed progressive disease prior to the first imaging timepoint (Arm 1 Full Analysis Set [FAS]). Patients who do not meet this criterion (e.g., lost to follow-up with no follow-up imaging) will be replaced. Some efficacy analyses may be performed with one or more tumor types combined (e.g., subgroup of Arm 1 FAS consisting of patients with BRAF V600 mutation vs. BRAF fusion).

Primary Endpoint:

Overall response rate will be calculated by the number of patients with best overall confirmed response of CR or PR as determined by an IRC using RANO criteria among patients in the Arm 1 FAS. All responses will be confirmed by a second scan ≥ 28 days after the initial response. An exact binomial test will be used to compare the observed response rate to the hypothesized null ORR of 21%, and a 95% CI will be calculated using the Clopper-Pearson method.

Secondary Endpoints:

Clinical efficacy in antitumor activity will be assessed as the following secondary endpoint efficacy outcome measures:

Overall response rate will be calculated by the number of patients with best overall confirmed response of CR or PR as determined by the treating investigator using the RANO criteria among patients in FAS.

Overall response rate will be calculated by the number of patients with best overall confirmed response of CR or PR as determined by an IRC using RAPNO–low-grade glioma criteria among patients in FAS.

Best overall response (BOR) is defined as the best response (CR, PR, minor response, SD, progressive disease [PD], not evaluable [NE], and unknown) recorded from the start of the treatment until disease progression/recurrence or death (taking the smallest measurements recorded since the treatment started as a reference for PD). The patient's best response assignment will depend on the achievement of both measurement and confirmation criteria. The first disease assessment will occur at approximately 12 weeks post-treatment. Response assessment will continue until progression or death. If a patient is given antitumor treatment (anticancer surgery/procedure, anticancer radiotherapy, and anticancer systemic therapy other

than the study treatment) prior to PD or death, determination of BOR will be calculated using tumor assessment responses up to the date of the last tumor assessment prior to the start date of the antitumor treatment.

The DOR will be evaluated in patients with BOR of CR or PR based on RANO and RAPNO criteria as determined by 1) an IRC and 2) the treating investigator (RANO only). Duration of response is measured by the length of response in FAS patients with best overall confirmed response of CR or PR using RANO criteria.

The length of response is defined as the time from the first date of a patient's best overall objective response until the time of disease progression using RANO and RAPNO criteria as determined by 1) an IRC and 2) the treating investigator (RANO only).

The duration of PFS will be evaluated in FAS based on RANO and RAPNO criteria following initiation of DAY101 as determined by 1) an IRC and 2) the treating investigator (RANO only). Progression-free survival is measured by the time following initiation of DAY101 to progression or death in patients treated with DAY101, whichever occurs first.

The TTR will be evaluated in FAS patients with response of CR or PR following initiation of DAY101 based on RANO and RAPNO criteria as determined by 1) an IRC and 2) the treating investigator (RANO only). Time to response is measured by the time to the first response following initiation of DAY101 in FAS patients with best overall confirmed response of CR or PR.

The clinical benefit rate will be evaluated based on the proportion of patients with BOR of CR, PR, MR, or SD in FAS. The BOR will be defined by RANO and RAPNO criteria, lasting 12 months or more following initiation of DAY101 as determined by 1) an IRC and 2) the treating investigator (RANO only).

Overall survival is defined as the date of enrollment to the date of death. Overall survival in months will be calculated as: (death date − enrollment date + 1) • (12/365.25). Censoring will occur as if there is no confirmation of death. Overall survival will be censored on the date that the patient is last known to be alive.

7.3.1.2. Arm 2: Low-Grade Glioma Extension

Where appropriate (e.g., in patients with measurable disease), patients will be evaluated via the same statistical methods as described for Arm 1.

7.3.1.3. Arm 3: Advanced Solid Tumor

The efficacy analyses will include all patients who received at least one dose of study drug, have measurable disease as determined by the IRC at baseline, and have follow-up imaging or radiographically confirmed progressive disease prior to the first imaging timepoint (Arm 3 FAS). Patients who do not meet this criterion (e.g., lost to follow-up with no follow-up

imaging) will be replaced. These analyses may be summarized by tumor type, and some efficacy analyses may be performed with one or more tumor types combined.

Primary Endpoint:

The estimate of the ORR will be calculated by the number of patients with best overall confirmed response of CR or PR as determined by an IRC using RECIST v1.1 criteria among the Arm 3 FAS. All responses will be confirmed by a second scan ≥ 28 days after the initial response. The estimate of the ORR will be accompanied by 1- and 2-sided CIs with various coverage probabilities (e.g., 80%, 95%). The primary analysis of ORR will be based on the responses determined by an IRC. A secondary analysis will be based on the treating investigator's assessment.

Secondary Endpoints:

Clinical efficacy will also be assessed by the following secondary endpoint efficacy outcome measures:

Overall response rate will be calculated by the number of patients with best overall confirmed response of CR or PR as determined by the treating investigator using RECIST v1.1 criteria among patients in FAS.

Best overall response is defined as the best response (CR, PR, SD, PD, NE, and unknown) recorded from the start of the treatment until disease progression/recurrence or death (taking the smallest measurements recorded since the treatment started as a reference for PD). The patient's best response assignment will depend on the achievement of both measurement and confirmation criteria. The first disease assessment will occur at approximately eight weeks post-treatment. Response assessment will continue until progression or death. If a patient is given antitumor treatment (anticancer surgery/procedure, anticancer radiotherapy, and anticancer systemic therapy other than the study treatment) prior to PD or death, determination of BOR will be calculated using tumor assessment responses up to the date of the last tumor assessment prior to the start date of the antitumor treatment.

Best overall response, PFS, TTR, CBR, and OS will be evaluated as defined above (per RECIST v1.1, as applicable).

7.3.2. Safety Analyses

Safety will be assessed by clinical review of all relevant parameters, including AEs, SAEs, laboratory values, and vital signs. Unless specified otherwise, the safety analyses will be conducted for the safety population defined in Section 7.1.

Summary tables and listings will be provided for all reported TEAEs, defined as AEs that start on or after the first administration of study drug. The reported AE term will be assigned a standardized Preferred Term using the Medical Dictionary for Regulatory Activities (MedDRA), version 17.1 or later.

7.3.2.1. Deaths

All deaths on study should be reported and recorded on the Death CRF page and should include the date and cause of death. Deaths will be reported in a patient listing and will include the primary cause of death and the number of days between the date of the last dose of study drug and death.

7.3.2.2. Laboratory Values

Laboratory values will be assigned toxicity grades when available using the NCI CTCAE, version 5.0 or later. Directional shifts in laboratory toxicity grades (comparing baseline grade with worst postbaseline grade) will be analyzed using standard shift tables, presenting number and proportion of patients and their maximum grade shift. For analytes without a toxicity grading scale, the shift table will present directional shifts from baseline to above or below the laboratory standard normal range using the maximum increase and/or decrease observed throughout the course of treatment/observation.

7.3.2.3. Vital Signs

All patients will have pretreatment baseline vital signs and predose measurements as described in Appendix A. The results for each vital sign will be summarized in a descriptive manner by calculating the mean, standard deviation, median, and range by time point in the same manner described for laboratory values. For these analyses, the minimum, maximum, average, and last postbaseline value will be determined relative to the baseline vital sign measurements only. The Wilcoxon signed rank test may be used to assist in the identification of any systematic changes.

7.3.2.4. Concentration QTc (cQTc) Analysis

All patients who received at least one dose of DAY101 and had at least one pair of evaluable ΔQTcF and DAY101 plasma concentration at any postdose time point. This will be the primary analysis set for exploring the relationship between ΔQTc intervals and plasma concentrations of DAY101. The relationship between ΔQTcF and the plasma concentrations of DAY101 may be investigated in an exploratory manner and will be reported in a standalone document.

7.3.2.5. Concomitant Medications

Prior and concomitant medications will be coded to the generic term using the current version of the WHO Drug Dictionary and listed by patient.

7.3.2.6. Pharmacokinetic Analyses

Plasma concentrations of DAY101 will be determined with a validated bioanalytical assay. The following PK parameters may be calculated where appropriate: C_{max}, minimum drug concentration (C_{min}), C_{ave}, time at which maximum observed concentration occurs (T_{max}), AUC from time zero to t ($AUC_{0–t}$), and apparent oral clearance of drug (CL/F).

Summary statistics will be generated as appropriate.

7.3.3. Health-Related Quality of Life

Changes in quality-of-life measures from baseline will be evaluated for patients ≥ 2 years of age or older using the PedsQL-Core, PedsQL-Cancer, and PROMIS® assessment. The PROMIS® questionnaire will only be administered to English-speaking patients enrolled in the U.S., Australia, and U.K. Longitudinal changes in quality-of-life scores from baseline will be summarized in a descriptive manner.

7.3.4. Visual Acuity Outcomes

Arms 1 and 2 (Low-Grade Glioma): Visual acuity (VA) progression is defined as a decline ≥ 0.2 logMAR (corrected for age). Visual acuity response is an improvement of ≥ 0.2 logMAR (corrected for age).

Summary statistics will be generated as appropriate.

7.3.5. Motor Function (Vineland) Outcomes

Arms 1 and 2 (Low-Grade Glioma): Change in the Vineland-3 Motor Skills domain from baseline will be evaluated after one and two years of treatment. The Vineland-3 Motor Skills questionnaire will be administered to patients with baseline motor function deficits at U.S. sites only.

Summary statistics will be generated as appropriate.

8. SAFETY

8.1. Adverse Events and Serious Adverse Events

All AEs, whether reported by the patient or the patient's parent/legal guardian or noted by study personnel, will be recorded in the patient's medical record on the Adverse Event eCRF.

See Appendix J for instructions on collecting, assessing, and reporting of AEs.

8.1.1. Time Period and Frequency for Collecting Adverse Event and Serious Adverse Event Information

Report only SAEs deemed related to protocol-mandated procedures from the signing of informed consent through to start of treatment with DAY101.

Report all AEs, including SAEs, from the start of treatment with DAY101 until 30 days after the last dose of DAY101.

After 30 days post–treatment discontinuation, report any targeted SAEs (e.g., neurological disorders, autoimmune disorders, ocular disorders, cardiac disorders, and secondary malignancies) observed by the investigator or reported by the patient, regardless of causality to study drug. In addition, report any SAEs considered related to DAY101.

For patients who screen fail or are enrolled, but do not receive DAY101, the reporting period for SAEs ends 30 days after the last procedure (e.g., screening procedure).

Table 14: Summary of Adverse Event Collection Periods

Time Period	AE Collection
ICF signature prior to first dose of DAY101	Protocol/procedure-related SAEs only
DAY101 to 30 days after last study drug dose	All AEs and SAEs
Beyond 30 days after last study drug dose (DAY101)	Targeted SAEs (e.g., neurological disorders, autoimmune disorders, ocular disorders, cardiac disorders, and secondary malignancies), regardless of causality to study drug<br>Any SAEs related to DAY101

Abbreviations: AE, adverse event; ICF, informed consent form; SAE, serious adverse event.

Report and record all SAEs to the Sponsor or designee within 24 hours following the investigator's knowledge of the events, as indicated in Appendix J. The investigator will submit any updated SAE data to the Sponsor within 24 hours of their being available.

If the investigator learns of any SAE, including a death, at any time after a patient has been discharged from the study, and he/she considers the event to be reasonably related to the study intervention or study participation, the investigator must promptly notify the Sponsor.

8.1.2. Method of Detecting Adverse Events and Serious Adverse Events

The method of recording, evaluating, and assessing causality of AE and SAE and the procedures for completing and transmitting SAE reports are provided in Appendix J.

Care will be taken not to introduce bias when detecting AEs and/or SAEs. Open-ended and nonleading verbal questioning of the patient or the patient's parent/legal guardian is the preferred method to inquire about AE occurrences.

8.1.3. Follow-Up of Adverse Events and Serious Adverse Events

After the initial AE/SAE report, the investigator is required to proactively follow each patient at subsequent visits/contacts. All SAEs will be followed until resolution, stabilization, the event is otherwise explained, or the patient is lost to follow-up. Further information on follow-up procedures is provided in Appendix J.

If a patient begins a new anticancer therapy, the AE reporting period for non-SAEs ends at the time the new treatment is started.

8.1.4. Regulatory Reporting Requirements for Serious Adverse Events

Any SAEs occurring during this study must be reported as follows:

- Prompt (within 24 hours of awareness) notification by the investigator to the Sponsor of an SAE is essential so that legal obligations and ethical responsibilities

toward the safety of patients and the safety of a study intervention under clinical investigation are met. Serious Adverse Events should be reported to the Sponsor via email at safetyreporting@dayonebio.com.

- The Sponsor has a legal responsibility to notify both the local regulatory authority and other regulatory agencies about the safety of a study intervention under clinical investigation. The Sponsor will comply with country-specific regulatory requirements relating to safety reporting to the regulatory authority, Institutional Review Boards (IRB)/Independent Ethics Committees (IEC), and investigators.
- An investigator who receives an investigator safety report describing an SAE or other specific safety information (e.g., summary or listing of SAEs) from the Sponsor will review and then file it along with the IB and will notify the IRB/IEC, if appropriate, according to local requirements.

8.1.5. Pregnancy

There is no relevant clinical experience with DAY101 in pregnant or lactating female patients, and animal reproductive studies have not been performed. Female patients of childbearing potential must have a negative pregnancy test prior to enrollment because of the potentially dangerous effects of the preparative chemotherapy on the fetus. This experimental therapy should not be administered to pregnant women or women who are breastfeeding.

- Details of all pregnancies in female patients and female partners of male patients will be collected for pregnancies occurring after the start of study intervention until six months after completing DAY101.
- If a pregnancy is reported, the investigator should inform the Sponsor within 24 hours of learning of the pregnancy and should follow the procedures outlined in Appendix K.
- Abnormal pregnancy outcomes (e.g., spontaneous abortion, fetal death, stillbirth, congenital anomalies, ectopic pregnancy) are considered SAEs.
- Details of all lactation cases in female patients will be collected while taking protocol-required therapies.
- If a lactation case is reported, the investigator should inform the Sponsor within 24 hours of learning of the lactation case and should follow the procedures outlined in Appendix K.

In addition to reporting any pregnancies or lactation cases occurring during the study, investigators should monitor for pregnancies or lactation cases that occur after the last dose of DAY101 through six months for female patients and for six months for female partners of male patients.

8.1.6. Deaths

Deaths occurring during the AE reporting period, regardless of attribution to DAY101, will be recorded as an AE and expeditiously reported to the Sponsor. However, deaths clearly attributed to underlying progression of malignancy do not need to be reported as SAEs.

When recording a death, the event or condition that caused or contributed to the fatal outcome should be recorded as the single medical condition on the Adverse Event eCRF and SAE report form, as applicable. If the cause of death is unknown and cannot be ascertained at the time or reporting, then “unexplained death” should be recorded on the Adverse Event eCRF and SAE reporting form, as applicable.

8.2. Safety Review Committee and Data Safety Monitoring Board

An SRC will be established to oversee the safety aspects of the study. The SRC membership will consist of the Sponsor’s Medical Monitor for this study, a Clinical Operations representative, and a clinically qualified representative of the Principal Investigator from each active clinical site contributing patients to that cohort. The SRC will meet monthly until completion of enrollment, and then quarterly until study closure.

In addition, an independent DSMB will be established and will meet approximately every three months to review safety and efficacy data across the entire DAY101 program. Any emergent safety issue identified by the SRC will be escalated to the DSMB.

8.3. Stopping Rules

Adverse events and SAEs are expected to occur frequently in this study, based on the patient population being accrued and on the nature of the advanced malignancies under study. As a result, there is no specific incidence rate of SAEs that will define a stopping rule. Instead, regular systematic review of SAEs will serve as the basis for pausing or prematurely stopping the study. Unexpected SAEs that are related to DAY101 will be the primary criteria for pausing or stopping the study. Review of these SAEs, and any decision to pause enrollment or terminate the study, will be determined by the DSMB, the Sponsor, and the Medical Monitor. Decisions to pause enrollment or terminate the study will be communicated promptly to investigators, IRBs/ IECs, Institutional Biosafety Committees (if applicable), and appropriate regulatory authorities.

8.4. Criteria for Pausing or Stopping the Study

The study will be paused for enrollment pending notification of the DSMB and appropriate regulatory authorities if any patient experiences any of the following events:

- Life-threatening (Grade 4) toxicity attributable to DAY101 that is unexpected and unmanageable (i.e., does not resolve to Grade 3 or lower within seven days)
- Death related to DAY101 therapy
- The study will be terminated for the following reasons:
- The Sponsor, IRB/IEC, or DSMB decides that patient safety may be compromised by continuing the study
- The Sponsor decides to discontinue the development of DAY101 for this indication or the development of DAY101 for all indications

8.5. Grading and Intensity of Adverse Events

The investigator will grade the severity of each AE using, when applicable, the NCI CTCAE, version 5.0. In the event of an AE for which no grading scale exists, the investigator will classify the AE as mild, moderate, severe, life-threatening/debilitating, or fatal, as defined in Table 15.

Table 15: NCI CTCAE Definitions of Severity for Adverse Reactions

Toxicity	Grade / Details
**Grade 1**	**Mild**; asymptomatic or mild symptoms; clinical or diagnostic observations only; no interventions required<br>(an event that is usually transient in nature and generally not interfering with normal activities)
**Grade 2**	**Moderate**; minimal, local, or noninvasive intervention indicated; some limitation of activities<br>(an event that is sufficiently discomforting to interfere with normal activities)
**Grade 3**	**Severe or medically significant but not immediately life-threatening**; hospitalization or prolongation of hospitalization required; disabling; limitation of patient's ability to care for him/herself<br>(an event that is incapacitating with inability to work or do usual activity, or inability to work or perform normal daily activity)
**Grade 4**	**Life-threatening consequences**; urgent intervention required<br>(an event that puts the patient at immediate or potential risk of death, requires hospitalization, or which drastically impacts a patient's well-being)
**Grade 5**	**Death related to adverse event** (fatal)

Abbreviations: NCI CTCAE, National Cancer Institute-Common Terminology Criteria for Adverse Events.

8.6. Relationship to Study Drug

The investigator will categorize each AE as to its potential relationship to investigational product using the categories of Yes (causally related) and No (unrelated), as defined below. The assessment of the relationship of an AE to the administration of investigational product is a clinical decision based on all available information at the time.

No:

The time course between the administration of study drug and the occurrence or worsening of the AE rules out a causal relationship, and another cause (concomitant drugs, therapies, complications, comorbidities, etc.) is suspected.

Yes:

A causal relationship between medicinal product and an AE is at least a reasonable possibility. The AE cannot reasonably be explained by the subject's clinical state, concomitant medical condition or concomitant therapies, and a temporal relationship exists between the event onset and administration of the study drug.

An unexpected AE is an experience not previously reported or an AE that occurs with specificity, severity, or frequency that is not consistent with the current DAY101 IB.

8.7. Serious Adverse Event Follow-Up

For all SAEs occurring during the study, the investigator must submit follow-up reports to the Sponsor regarding the status of the SAE and the patient's subsequent course until the SAE has resolved, the condition stabilizes or is deemed chronic (in the case of persistent impairment), the patient or the patient's parent/legal guardian withdraws consent for further follow-up, or the patient dies.

9. STUDY ADMINISTRATION

9.1. Regulatory and Ethical Considerations

9.1.1. Regulatory Authority Approval

This study will be conducted in accordance with the International Council for Harmonisation guidelines for Good Clinical Practices (ICH-GCP E6 [R2]), an international ethical and scientific quality standard for designing, conducting, recording, and reporting studies that involve human patients. All applicable country and local regulations will also be observed; these include the provisions of the German Medicinal Products Act (Arzneimittelgesetz [AMG]), the Ordinance on the implementation of Good Clinical Practice in the conduct of clinical trials on medicinal products for use in humans (GCP-V), as well as the General Data Protection Regulation 2016/679 (GDPR) and the German Federal Data Protection Act (Baden-Württemberg State Data Protection Act [BDSG])/State Data Protection Act of Baden-Württemberg (LDSG BW). Compliance with these standards provides assurance that the rights, safety, and well-being of study patients are protected, consistent with the principles in the Declaration of Helsinki, and that the clinical study data are credible.

9.1.2. Ethics Approval

It is the responsibility of the investigator to ensure that the appropriate IRB/ Research Ethics Board (REB)/IEC/ Human Research Ethics Board (HREC) has reviewed and approved this protocol prior to initiating the study. The investigator must provide the Sponsor or Sponsor's representative with current and revised IRB/REB/IEC/HREC membership rosters that include the members' occupations and qualifications or provide a copy of the US Department of Health and Human Services Assurance Number.

The IRB/REB/IEC/HREC must also review and approve the clinical site's ICF, pediatric assent form, other written information provided to the patient, and all advertisements that may be used for patient recruitment. The investigator will provide the study monitor with copies of these documents and of dated IRB/REB/IEC/HREC approval(s) prior to the start of the study.

The IRBs/REBs/IECs/HREC are required to determine whether child assents are appropriate for all studies that include pediatric patients. The IRB/REB/IEC/HREC may opt to waive assent if the patients are not capable of understanding (e.g., based on level of intellectual development or maturity) or if the study is in the best interest of the patient (i.e., strong

possibility of benefit and no other alternatives are available). If an IRB/REB/IEC/HREC chooses to waive assent, this must be documented in the IRB/REB/IEC/HREC approval letter.

If the protocol or the ICF or pediatric assent form is amended during the study, the investigator is responsible for ensuring that the IRB/REB/IEC/HREC has reviewed and approved these amended documents. Approval of the amended documents must be obtained from the IRB/REB/IEC/HREC before implementation and before new patients are consented to participate in the study using the amended version of the ICF/pediatric assent form. The investigator must provide the Sponsor with the dated IRB/REB/IEC/HREC approval of the amended documents as soon as available.

9.1.3. Patient Informed Consent and Pediatric Assent

The investigator and designated site staff who will perform the consent/assent process are responsible for knowing country-specific regulations and other local requirements with regard to child assent to ensure that the assent process is conducted in accordance with those requirements. Prior to study entry, the investigator or designee will explain the nature, purpose, benefits, and risks of participation in the study to each patient and the patient’s legally acceptable representative, legal guardian, or impartial witness. Written informed consent and pediatric assent, where applicable, must be obtained prior to the patient’s entering the study (before initiation of any study-related screening procedure). In pediatric cases, where applicable and according to local regulations, both parents may be required to sign informed consent. Sufficient time will be allowed to discuss any questions raised by the patient. The ICF/pediatric assent form, which will contain all US federally-required elements, all ICH-required elements, and Health Insurance Portability and Accountability Act authorization information in a language that is understandable to the patient, must be signed by all patients, or by the patient’s legal representative. The process of obtaining the ICF and pediatric assent form, where applicable, will be in compliance with all applicable local and country regulations and ICH requirements.

The pediatric assent form must have a date and signature line for the child. State laws differ in their requirements for patients who have not reached the legal age of majority and IRBs/REBs/IECs/HRECs are responsible for following their local regulations. Use of an assent is not a substitute for parental permission. Parents/guardians must receive a full ICF to review and sign.

If the ICF and pediatric assent form, where applicable, is amended during the study, the investigator must follow all applicable regulatory requirements pertaining to IRB/REB/IEC/HREC approval of the amended form. The clinical site must use the amended ICF/pediatric assent form for all new patients and repeat the consent/assent process with the amended form for any ongoing patients.

An initial ICF (for adult patients or parent/guardian of child/adolescent patients) and pediatric assent form (for child/adolescent patients, as applicable) will be provided to the

investigator to prepare the ICF and assent documents to be used at the Principal Investigator's site. The sample ICFs/pediatric assent forms prepared by the Sponsor are provided in the Study Manual.

9.1.4. Investigator Reporting Requirements

In accordance with applicable regulatory requirements, the investigator is solely obligated to inform the IRB/REB/IEC/HREC of progress of the study and notify the IRB/REB/IEC/HREC of study closure. The investigator must also provide the Sponsor with copies of all IRB/REB/IEC/HREC correspondence that relate to study approvals, updates, or changes. The investigator is also responsible for forwarding to the IRB/REB/IEC/HREC reports of any SAEs from other studies conducted with the same investigational product that were provided by the Sponsor.

9.2. Data Management

Data will be recorded at the site on source documents and reviewed by the Sponsor's site monitor, who will verify data recorded in the eCRFs with source documents, during periodic visits. All corrections or changes made to any study data must be appropriately tracked in an audit trail in the eCRF system. The eCRFs will be considered complete when all missing, incorrect, and/or inconsistent data have been accounted for. Additional instructions on eCRF completion are provided in the electronic data capture (EDC) Manual. eCRFs should be entered within 5 to 10 business days of each visit and all queries should be answered within 5 to 10 business days of receipt.

All eCRFs must be reviewed and signed by the investigator.

9.3. Study Monitoring

Prior to the start of the study, the Sponsor's monitor or designee will contact the clinical site to discuss the protocol and data collection procedures and conduct applicable training of site personnel. The Sponsor and its designees will also periodically contact the clinical site during the conduct of the study (which will include remote and on-site visits) in accordance with applicable regulations and GCP. During these contacts, the monitoring activities will include the following:

- Checking and assessing the progress of the study
- Reviewing study data collected to date for completeness and accuracy
- Conducting source document verifications by reviewing each patient's eCRF against source documents
- Identifying any issues and addressing resolutions
- Recording and reporting protocol deviations not previously reported to the Sponsor
- Confirming that SAEs have been properly reported to the Sponsor and submitted to the IRB/REB/IEC/HREC if appropriate

These activities will be done in order to verify that the data are authentic, accurate, and complete; that the safety and rights of the patient are being protected; and that the study is conducted in accordance with the currently approved protocol, GCP, and all applicable regulatory requirements. Additionally, to ensure compliance with GCP and all applicable regulatory requirements, the Sponsor or designee may conduct a quality assurance audit.

9.4. Termination

Upon completion of the study, the following activities, when applicable, must be conducted by the study monitor and the investigator:

- Submission of all study data to the Sponsor
- Completion of all data clarifications and/or resolutions
- Reconciliation and final disposition of investigational product
- Review of site study files for completeness

In addition, the Sponsor reserves the right to temporarily suspend or prematurely terminate this study for any reason.

If the study is suspended or terminated for safety reasons, the Sponsor will promptly inform the investigator, and will also inform the IRB/REB/IEC/HREC with the reasons for the action. In the event of prematurely termination, all study data must be submitted to the Sponsor. In addition, the clinical site must document final disposition of all unused investigational product in accordance with the Sponsor's procedures.

9.5. Records Retention

Patient records, source documents, monitoring visit logs, investigational product inventory, regulatory documents, and other correspondence pertaining to the study must be maintained in the appropriate site study files according to ICH GCP and applicable regulatory requirement(s). Records will be retained for at least two years after the last marketing application approval or two years after formal discontinuation of the clinical development of the investigational product or according to applicable regulatory requirement(s). If the investigator withdraws from the responsibility of keeping the study records, custody must be transferred to a person willing to accept the responsibility. The Sponsor must be notified immediately by telephone or e-mail and the notification confirmed in writing if a custodial change occurs.

9.6. Confidentiality of Information

Patient names will remain confidential and will not be supplied to the Sponsor or its designee. The investigator will maintain a personal patient identification list (patient and treatment numbers with the corresponding patient names) to enable records to be identified.

10. REFERENCES

Armstrong GT, Pan Z, Ness KK, Srivastava D, Robison LL. Temporal trends in cause-specific late mortality among 5-year survivors of childhood cancer. J Clin Oncol. 2010;28(7): 1224-1231.

Ater JL, Zhou T, Holmes E, et al. Randomized study of two chemotherapy regimens for treatment of low-grade glioma in young children: a report from the Children's Oncology Group. J Clin Oncol. 2012;30(21):2641-7.

Beebe DW, Ris MD, Armstrong FD, et al. Cognitive and adaptive outcome in low-grade pediatric cerebellar astrocytomas: evidence of diminished cognitive and adaptive functioning in National Collaborative Research Studies (CCG 9891/POG 9130) J Clin Oncol. 2005;23(22):5198-204.

Behling F, Schittenhelm J. Oncogenic BRAF alterations and their role in brain tumors. Cancers (Basel). 2019;11(6):Pii:E794.

Bellantoni AJ, Wagner LM. Pursuing precision: receptor tyrosine kinase inhibitors for treatment of pediatric solid tumors. Cancers (Basel). 2021;13(14):3531. PMID: 34298746; PMCID: PMC8303693.

Bergthold G, Bandopadhayay P, Bi WL, et al. Pediatric low-grade gliomas: how modern biology reshapes the clinical field. Biochim Biophys Acta. 2014;1845(2):294-307.

Bouffet E, Jakacki R, Goldman S, et al. Phase II study of weekly vinblastine in recurrent or refractory pediatric low-grade glioma. J Clin Oncol. 2012;30(12):1358-63.

Cantwell-Dorriss ER, O'Leary JJ, Sheils OM. BRAFV600E: implications for carcinogenesis and molecular therapy. Mol Cancer Ther. 2011;10(3):385-94.

Chen YH, Gutmann DH. The molecular and cell biology of pediatric low-grade gliomas. Oncogene. 2014;33(16):2019-26.

Coffin CM, Beadling C, Neff T, Corless CL, Davis JL. Infantile fibrosarcoma with a novel RAF1 rearrangement: The contemporary challenge of reconciling classic morphology with novel molecular genetics. Hum Pathol: Case Rep. 2020;22:200434.

Davies H, Bignell GR, Cox C, et al. Mutations of the BRAF gene in human cancer. Nature. 2002;417(6892):949-54.

Del Bufalo F, Ceglie G, Cacchione A, et al. BRAF V600E inhibitor (vemurafenib) for BRAF V600E mutated low grade gliomas. Front Oncol. 2018;8:526.

Drilon A, Laetsch TW, Kummar S, et al. Efficacy of Larotrectinib in TRK Fusion-Positive Cancers in Adults and Children. N Engl J Med. 2018;378(8):731-739. PMID: 29466156; PMCID: PMC5857389.

Eisenhauer EA, Therasse P, Bogaerts J, et al. New response evaluation criteria in solid tumors: revised RECIST guideline (version 1.1). Eur J Cancer. 2009;45:228-247.

European Commission (EC) Ethical considerations for clinical trials on medicinal products conducted with the paediatric population - Recommendations of the ad hoc group for the development of implementing guidelines for Directive 2001/20/EC relating to good clinical practice in the conduct of clinical trials on medicinal products for human use. 2008. https://ec.europa.eu/health//sites/health/files/files/eudralex/vol-10/ethical_considerations_en.pdf

European Commission (EC) Ethical considerations for clinical trials on medicinal products conducted with minors - Recommendations of the expert group on clinical trials for the implementation of Regulation (EU) No 536/2014 on clinical trials on medicinal products for human use, Revision 1, 2017. https://ec.europa.eu/health/sites/health/files/files/eudralex/vol-10/2017_09_18_ethical_consid_ct_with_minors.pdf

European Medicines Agency (2020). Guidance On The Management Of Clinical Trials During The COVID-19 (Coronavirus) Pandemic. https://ec.europa.eu/health/sites/health/files/files/eudralex/vol-10/guidanceclinicaltrials_covid19_en.pdf

Fangusaro J, Onar-Thomas A, Young Poussaint T, et al. Selumetinib in pediatric patients with BRAF-aberrant or neurofibromatosis type 1-associated recurrent, refractory, or progressive low-grade glioma: a multicenter, phase 2 trial. Lancet Oncol. 2019;20(7):1011-22.

Flaherty KT, Puzanov I, Kim KB, et al. Inhibition of mutated, activated BRAF in metastatic melanoma. N Engl J Med. 2010;363:809-19.

Food and Drug Administration (2007). Guidance for Industry: Clinical Trial Endpoints for the Approval of Cancer Drugs and Biologics. https://www.fda.gov/regulatory-information/search-fda-guidance-documents/clinical-trial-endpoints-approval-cancer-drugs-and-biologics

Food and Drug Adminsitration (2020). Guidance or Industry, Investigators, and Institutional Review Boards: FDA Guidance on Conduct of Clinical Trials of Medical Products during COVID-19 Public Health Emergency. https://www.fda.gov/regulatory-information/search-fda-guidance-documents/fda-guidance-conduct-clinical-trials-medical-products-during-covid-19-public-health-emergency

Goebel AM, Gnekow AK, Kandels D, et al. Natural history of pediatric low-grade glioma disease – first multi-state model analysis. J Cancer. 2019;10(25):6314-26.

Hargrave DR, Bouffet E, Tabori U, et al. Efficacy and safety of dabrafenib in pediatric patients with BRAF V600 mutation-positive relapsed or refractory low-grade glioma: results from a phase I/IIa study. Clin Cancer Res. 2019;25:1-9.

Hascheck WM, editor. Handbook of toxicologic pathology, 2nd ed. San Diego, CA: Academic Press;2002.

Jacob K, Quang-Khuong DA, Jones DT, et al. Genetic aberrations leading to MAPK pathway activation mediate oncogene-induced senescence in sporadic pilocytic astrocytomas. Clin Cancer Res. 2011;17(14):4650-60.

Johnson GL, Stuhlmiller TJ, Angeus SP, et al. Molecular pathways: adaptive kinome reprogramming in response to targeted inhibition of the BRAF-MEK-ERK pathway in cancer. Clin Cancer Res. 2014;20(10):2516-22.

Jones DT, Gronych J, Lichter P, et al. MAPK pathway activation in pilocytic astrocytoma. Cell Mol Life Sci. 2012;69(11):1799-811.

Jones DT, Kocialkowski S, Liu L, et al. Tandem duplication producing a novel oncogenic BRAF fusion gene defines the majority of pilocytic astrocytomas. Cancer Res. 2008;68(21):8673-7.

Karajannis MA, Legault G, Fisher MJ, et al. Phase II study of sorafenib in children with recurrent or progressive low-grade astrocytomas. Neuro Oncol. 2014;16(10):1408-16.

Karnofsky D and Burchenal J. The clinical evaluation of chemotherapeutic agents in cancer. Evaluation of Chemotherapeutic Agents 1949. e. MacLeod C. New York, NY, Columbia University Press**:** 191–205.

Kirchhoff AC, Leisenring W, Krull KR, et al. Unemployment among adult survivors of childhood cancer: a report from the childhood cancer survivor study. Med Care. 2010;48(11):1015-1025.

Knezevich SR, McFadden DE, Tao W, et al. A novel ETV6-NTRK3 gene fusion in congenital fibrosarcoma. Nat Genet. 1998;18(2):184-7.

Lansky SB, List MA, Lansky LL, et al. The measurement of performance in childhood cancer patients. Cancer. 1987;60(7):1651-56.

Mao Q, Unadkat J. Role of the breast cancer resistance protein (BCRP/ABCG2) in drug transport- an update. AAPS J. 2015;17(1):65-82.

Marshall WA, Tanner JM. Variations in pattern of pubertal changes in girls. *Arch Dis Child*. 1969;44(235):291-303.

Marshall WA, Tanner JM. Variations in the pattern of pubertal changes in boys. *Arch Dis Child*. 1970;45(239):13-23.

Mitby PA, Robison LL, Whitton JA, et al. Utilization of special education services and educational attainment among long-term survivors of childhood cancer: a report from the Childhood Cancer Survivor Study. Cancer. 2003;97(4):1115-1126.

Mösteller RD. Simplified calculation of body-surface area. N Engl J Med. 1987;317(17):1098.

Nicolaides T, Nazemi KJ, Crawford J, et al. Phase I study of vemurafenib in children with recurrent or progressive BRAFV600E mutant brain tumors: Pacific Pediatric Neuro-Oncology Consortium study (PNOC-002). Oncotarget. 2020;11(21):1942.

Offer K, McGuire M, Venetsanakos E, et al. Activity of PAN-RAF inhibitor DAY101 in a pediatric patient with a recurrent spindle cell sarcoma harboring a novel SNX8:BRAF Gene Fusion. Poster submitted to: CTOS Annual Meeting (virtual). 12 Nov 2021.

Olszanski AJ, Gonzalez R, Corrie P, et al. Phase 1 study of the investigational, oral pan-RAF kinase inhibitor TAK-580 (MLN2480) in patients with advanced solid tumors or melanoma: final analysis. #410P. Poster presented at ESMO Congress 08-12 September 2017.

Ostrom QT, Cioffi G, Gittleman H, et al. CBTRUS statistical report: primary brain and other central nervous system tumors diagnosed in the United States in 2012-2016. Neuro Oncol. 2019;21(Supplement_5):v1-v100.

Packer RJ, Pfister S, Bouffet E, et al. Pediatric low-grade gliomas: implications of the biologic era. Neuro Oncol. 2017;19(6):750-61.

Penman CL, Faulkner C, Lowis SP, et al. Current understanding of BRAF alterations in diagnosis, prognosis, and therapeutic targeting in pediatric low-grade gliomas. Front Oncol. 2015;5:54.

Penning AJ, Al-Ibraheemi A, Michal M, Larsen BT, et al. Novel BRAF gene fusions and activating point mutations in spindle cell sarcomas with histologic overlap with infantile fibrosarcoma. Mod Pathol. 2021;34(8):1530-1540. Epub 2021 Apr 13. PMID: 33850302.

Pfister S, Janzarik WG, Remke M, et al. BRAF gene duplication constitutes a mechanism of MAPK pathway activation in low-grade astrocytomas. J Clin Invest. 2008;118(5):1739-49.

Pui CH, Cheng C, Leung W, et al. Extended follow-up of long-term survivors of childhood acute lymphoblastic leukemia. N Engl J Med. 2003;349(7): 640-649.

Rankin A, Johnson A, Roos A, et al. Targetable BRAF and RAF1 alterations in advanced pediatric cancers. Oncologist. 2021;26(1):e153-e163.

Ris MD, Beebe DW, Armstrong FD, et al. Cognitive and adaptive outcome in extracerebellar low-grade brain tumors in children: a report from the Children's Oncology Group. J Clin Oncol. 2008;26(29):4765-70.

Ross JS, Wang K, Chmielecki J, et al. The distribution of BRAF gene fusions in solid tumors and response to targeted therapy. Int J Cancer. 2016;138(4):881-90.

Ryall S, Tabori U, Hawkins C. Pediatric low-grade glioma in the era of molecular diagnostics. Acta Neuropathol Commun. 2020;8(1):1-22.

Schemper M, Smith TL. A note on quantifying follow-up in studies of failure time. Control Clin Trials. 1996; 17(4):343-346.

Shaw AT, Hsu PP, Awad MM, et al. Tyrosine kinase gene rearrangements in epithelial malignancies. Nat Rev Cancer. 2013;13(110):772-87.

Sievert AJ, Jackson EM, Gai X, et al. Duplication of 7q34 in pediatric low-grade astrocytomas detected by high-density single-nucleotide polymorphism-based genotype arrays results in novel BRAF fusion gene. Brain Pathol. 2009;19(3):449-58.

Simon R. Optimal two-stage designs for phase II clinical trials. Control Clin Trials. 1989;10:1-10.

Steliarova-Foucher E, Colombet M, Ries LAG, et al. International incidence of childhood cancer, 2001-10: a population-based registry study. Lancet Oncol. 2017;18(6):719-731.

Stransky N, Cerami E, Schalm S, Kim JL, Lengauer C. The landscape of kinase fusions in cancer. Nat Commun. 2014 Sep 10;5:4846. PMID: 25204415; PMCID: PMC4175590.

Sun Y, Alberta JA, Pilarz C, et al. A brain-penetrant RAF dimer antagonist for the noncanonical BRAF oncoprotein of pediatric low-grade astrocytomas. Neuro Oncol. 2017;19(6):774-85.

Tihan T, Ersen A, Qaddoumi I, et al. Pathologic characteristics of pediatric intracranial pilocytic astrocytomas and their impact on outcome in 3 countries: a multi-institutional study. Am J Surg Pathol. 2012;36(1):43-55.

Tissot C, Singh Y, Sekarski N. Echocardiographic evaluation of ventricular function—for the neonatologist and pediatric intensivist. Front Pediatr. 2018;6:79.

Waanders AJ, Minturn JE, Fisher MJ. Pediatric low-grade gliomas: diagnosis, treatment, and future directions. In Hayat MA, ed. Tumors of the Central Nervous System, Volume 14; 2016:13-22.

Ward E, DeSantis C, Robbins A, Kohler B, Jemal A. Childhood and adolescent cancer statistics, 2014. CA Cancer J Clin. 2014;64(2): 83-103.

Weinstein IB. Addiction to oncogenes – the Achilles heal of cancer. Science. 2002;297(5578):63-4.

Wen PY, Chang SM, Van den Bent MJ, Vogelbaum MA, Macdonald DR, Lee EQ. Response Assessment in Neuro-Oncology Clinical Trials. J Clin Oncol 2017; 35(21):2439-49.

Williams EA, Shah N, Montesion M, et al. Melanomas with activating RAF1 fusions: clinical, histopathologic, and molecular profiles. Mod Pathol. 2020;33(8):1466-1474.

Wright K, Krzykwa E, Greenspan L, et al. EPCT-01. Phase I study of DAY101 (TAK580) in children and young adults with radiographically recurrent or progressive low-grade glioma (LGG). Neuro Oncol. 2020;22(Suppl 3):iii304.

Yaeger R, Corcoran RB. Targeting alterations in the RAF-MEK pathway. Cancer Discov. 2019;(3):329-41.

Yeh JM, Nekhlyudov L, Goldie SJ, Mertens AC, Diller L. A model-based estimate of cumulative excess mortality in survivors of childhood cancer. Ann Intern Med. 2010;152(7): 409-417, W131-408.

Appendix A: Study Assessments

Table 16: Arms 1 and 2 Schedule of Assessments

**Assessment**	**Pre-Enrollment**	**Post-Enrollment**									
**Visit Day (Time Window)**	**Screening [a] (Pre-First Dose) (≤ 28 days)**	**Cycle 1**				**Cycles 2–26 (± 3 days)**	**Cycle 27 – EOT (± 3 days)**		**EOT Visit (±7 days)**	**30-Day Post-EOT Safety Follow-Up (±14 days)**	**LTFU Every 3 ± 1 mo [d]**
		**Day 1 [b] (Pre-Dose)**	**Day 8 (± 3 days)**	**Day 15 (± 3 days)**	**Day 22 (± 3 days)**		**On Drug Therapy [c]**	**Drug Holiday**			
Informed consent/assent	X [e]										
Demographics, medical, surgical, and cancer history (including prior 12 months of scans, date of progression, best response, and DOR from prior therapies, and tolerability of prior therapy, if available)	X										
Confirmation that archival tumor tissue sample is available [f]	X										
Physical examination [g]	X	X		X		X	X		X	X	
Neurological examination [g]	X	X		X		X	X		X	X	
Dermatologic examination incl. suspicious lesions [g]	X	X		X		X	X		X	X	
Ophthalmology examination [h]	X	Perform every time tumor assessments are done.							X	X	
Visual acuity testing [i]	X	Perform every time tumor assessments are done.							X	X	
Tanner Stages [j]	X					C13D1 and C27D1	Once yearly				Once yearly
Vital signs incl. height and weight	X	X		X		X [k]	X		X	X	

Assessment	Pre-Enrollment	Post-Enrollment									
		Cycle 1					Cycle 27 – EOT (± 3 days)				
Visit Day (Time Window)	Screening [a] (Pre-First Dose) (≤ 28 days)	Day 1 [b] (Pre-Dose)	Day 8 (± 3 days)	Day 15 (± 3 days)	Day 22 (± 3 days)	Cycles 2–26 (± 3 days)	On Drug Therapy [c]	Drug Holiday	EOT Visit (±7 days)	30-Day Post-EOT Safety Follow-Up (±14 days)	LTFU Every 3 ± 1 mo [d]
12-lead ECG in triplicate [l]	X	X pre-dose and with each PK blood draw		X		C2D1, C4D1, and every 3rd cycle through C13 (C7D1, C10D1, and C13D1)			X		
Echocardiogram/MUGA [m]	X					C2D1, C4D1, and every 3rd cycle (C7D1, C10D1, etc.)	X		X		
Creatine phosphokinase	X					C2D1, C4D1 and every 4th cycle (C8D1, C12D1, etc.)	X		X		
Hematology and chemistry	X	X [n, o]		X		X [k]	X		X	X	

**Assessment**	**Pre-Enrollment**	**Post-Enrollment**									
		**Cycle 1**					**Cycle 27 – EOT (± 3 days)**				
**Visit Day (Time Window)**	**Screening [a] (Pre-First Dose) (≤ 28 days)**	**Day 1 [b] (Pre-Dose)**	**Day 8 (± 3 days)**	**Day 15 (± 3 days)**	**Day 22 (± 3 days)**	**Cycles 2–26 (± 3 days)**	**On Drug Therapy [c]**	**Drug Holiday**	**EOT Visit (±7 days)**	**30-Day Post-EOT Safety Follow-Up (±14 days)**	**LTFU Every 3 ± 1 mo [d]**
Pregnancy test by serum or urine (only for women of childbearing potential)	X	X [n]				X [k]	X	X	X	X	
Thyroid function test (TSH, T3, and T4)	X	X [n]				C2D1, C3D1, and every other cycle (C5D1, C7D1, etc.)	X		X	X	
Tumor assessment: MRI of brain/spine, as appropriate	X					X [p]					X [d]
PK blood samples [q]		X		X		C2D1, C4D1, and every subsequent third cycle through C13 (C7D1, C10D1, and C13D1)					
Karnofsky/Lansky Performance Score	X	X		X		X [k]	X		X	X	

Assessment	Pre-Enrollment	Post-Enrollment									
		Cycle 1					Cycle 27 – EOT (± 3 days)				
Visit Day (Time Window)	Screening [a] (Pre-First Dose) (≤ 28 days)	Day 1 [b] (Pre-Dose)	Day 8 (± 3 days)	Day 15 (± 3 days)	Day 22 (± 3 days)	Cycles 2–26 (± 3 days)	On Drug Therapy [c]	Drug Holiday	EOT Visit (±7 days)	30-Day Post-EOT Safety Follow-Up (±14 days)	LTFU Every 3 ± 1 mo [d]
PedsQL-Core, PedsQL-Cancer and PROMIS® [r]		X				C4D1 and every third cycle (C7D1, C10D1, etc.)	X		X		
Vineland motor scale [s]		X				C13D1 and C26D1	C39D1		X		
Telephone follow-up			X		X						
Concomitant medication [t]	X										
Adverse events	X [u]										
Optional tissue collection [v]	X										
DAY101 dosing [w]		X									
DAY101 administration diary [x]		X							X		
DAY101 drug dispensation/return [y]		X							X		
Post-treatment anti-cancer medications											X
Survival status											X

Abbreviations: AE, adverse event; C, Cycle; D, Day; DOR, duration of response; ECG, electrocardiogram; ECHO, echocardiogram; eCRF, electronic case report form; EOT, end of treatment; FFPE, formalin-fixed paraffin embedded; ICF, informed consent form; LTFU, long-term follow-up; MRI, magnetic resonance imaging; MUGA, multiple-gated acquisition; OPG; optic pathway glioma; PedsQL-Cancer, Pediatric Quality of Life™—Cancer ; PedsQL-Core, Pediatric Quality of Life Inventory™—Core; PK, pharmacokinetics; PROMIS®, Patient-Reported Outcomes Measurement Information System; SAE, serious adverse event; T3, triiodothyronine; T4, tetraiodothyronine; TSH, thyroid stimulating hormone.

[a] Results of screening assessments must be reviewed and approved before patient enrollment to confirm that the patient meets the eligibility criteria.

[b] Screening tests that are completed within the 48 hours prior to start of DAY101 and duplicate C1D1 tests will be accepted as fulfilling the C1D1 assessments. Repeat testing on C1D1 is not necessary for this scenario, and results should be recorded in the screening set of eCRFs.

[c] All assessments should be completed at C27D1 and D1 of every third cycle (e.g., C27D1, C30D1, C33D1, etc.) until the EOT visit.

[d] Only patients who discontinue treatment due to a reason other than disease progression will continue radiographic tumor assessments. Survival status and subsequent anticancer therapies will be collected for patients in LTFU; these data may be obtained by phone.

[e] Informed consent/assent may be obtained more than 28 days prior to patient enrollment, but must be provided before any study-specific procedures are performed; however, evaluations performed as part of routine care prior to informed consent can be used as screening evaluations if permitted by site Institutional Review Board/Ethics Committee policies and performed within protocol-specified windows.

[f] Tumor tissue (archival) will be obtained at enrollment whenever available. FFPE tumor blocks are preferred but in cases in which this is not possible, freshly cut FFPE slides should be obtained. See the Laboratory Manual for specific instructions. Fresh tissue biopsy is required during screening ONLY if archival tumor tissue sample is not available.

[g] All participants will have a neurological, physical, and dermatological examination during screening. For all subsequent visits, the examination will be symptom directed (at C1D1 examination should be completed before the first dose of study treatment).

[h] Screening ophthalmology examination is required for all patients. For OPG patients or those with an underlying visual deficit related to the primary malignancy, assessment completed every time tumor assessments are done and at the EOT visit. The 30-day post-EOT safety follow up visit is required only if following for ocular toxicity. For all other patients, symptom-directed examinations may be completed as needed.

[i] Screening visual acuity testing is required for all patients. For OPG patients or those with an underlying visual deficit related to the primary malignancy, assessment completed every time tumor assessments are done and at the EOT visit. The 30-day post-EOT safety follow up visit is required only if following for ocular toxicity. For all other patients, symptom-directed examinations may be completed as needed.

[j] For patients > 8 years of age, pubertal stage will be measured before the start of treatment (screening) and yearly thereafter while on active treatment and during LTFU through sexual maturity.

[k] Assessment completed at D1 of every cycle.

[l] On C1D1, ECGs are to be collected predose and in conjunction with PK draws. When ECGs coincide with PK draw days after C1D1, ECGs should be performed after dosing but before the PK blood draw. Additional ECGs are to be performed if clinically indicated.

[m] Must use the same assessment (ECHO or MUGA) at screening throughout the study.

[n] This assessment is intended to confirm suitability for treatment after enrollment and prior to first dose on C1D1. If this assessment has been performed during screening within 48 hours (seven days for pregnancy test) prior to C1D1, this assessment does not need to be performed on C1D1 unless the patient's clinical status has changed (e.g., onset of new symptoms indicative of clinical deterioration). If the assessment is performed on C1D1, the results must be reviewed by the investigator prior to any treatment being administered.

[o] See Section 6.7 and the Laboratory Manual for more detailed information on laboratory assessments.

[p] MRI of the brain and spine will be performed at screening in all patients. Assessments will be performed at the end of C3 (i.e., on or up to seven days before C4D1), end of C6 (i.e., on or up to seven days before C7D1), and then at the end of every three cycles thereafter (on or up to seven days before Day 1 of the next cycle). Follow-up images can be limited to areas with known disease from baseline images. To ensure image consistency, the same imaging modalities and acquisition protocols used at screening are to be used for subsequent tumor assessments. MRIs are to be performed per the protocol-defined schedule regardless of whether study treatment is reduced, held, or discontinued. For patients who discontinue study treatment before radiographic progressive disease, every effort should be made to document progressive disease with subsequent imaging. Confirmation of progression is not required.

[q] See Section 6.8, Table 12 for PK sampling schedule.

[r] The PROMIS® questionnaire will only be administered to English-speaking patients enrolled in the U.S., Australia, and U.K.

[s] The Vineland-3 Motor Skills questionnaire will only be administered to patients enrolled in the U.S., and only by third-party (trained and centralized) personnel.

[t] Document concomitant medication taken from 28 days prior to the planned C1D1 through the end of study participation. Only subsequent anticancer therapy(ies) will be recorded at LTFU.

[u] Refer to Section 8.1.1 for information on time period and frequency of collecting AE and SAE information. Note: only targeted SAEs will be collected beyond 30 days after last study drug dose.

[v] Participants who sign the optional ICF allowing for collection of tumor sample will have tumor specimen collected in the event a tumor-directed surgery occurs during study participation.

[w] DAY101 will be administered in clinic on C1D1 and at all PK blood draw visits. DAY101 will be taken once weekly at home otherwise until study treatment is discontinued.

[x] The study diary will be completed by the patient, or patient's parent or legal guardian, for every dose of study drug administered starting at C1D1. The Investigator or study coordinator should review diary entries for compliance, in particular for any potential missed doses, at every in-clinic or remote visit.

[y] DAY101 is to be dispensed to patients at D1 of every cycle and any unused drug should be returned at the start of the following cycle. Study drug from last cycle should be returned at EOT visit. DAY101 can be dispensed at D1 of every third cycle starting at C27.

Table 17: Arm 3 Schedule of Assessments

**Assessment**	**Pre-Enrollment**	**Post-Enrollment**									
**Visit Day (Time Window)**	**Screening [a] (Pre-First Dose) (≤ 28 days)**	**Cycle 1**				**Cycles 2–26 (± 3 days)**	**Cycle 27 – EOT (± 3 days)**		**EOT Visit (±7 days)**	**30-Day Post-EOT Safety Follow-Up (±14 days)**	**LTFU Every 3 ± 1 mo [d]**
		**Day 1 [b] (Pre-Dose)**	**Day 8 (± 3 days)**	**Day 15 (± 3 days)**	**Day 22 (± 3 days)**		**On Drug Therapy [c]**	**Drug Holiday**			
Informed consent/assent	X [e]										
Demographics, medical, surgical, and cancer history (including prior 12 months of scans, date of progression, best response, and DOR from prior therapies, and tolerability of prior therapy, if available)	X										
Confirmation that archival tumor tissue sample is available [f]	X										
Physical examination [g]	X	X		X		X	X		X	X	
Neurological examination [g]	X	X		X		X	X		X	X	
Dermatologic examination incl. suspicious lesions [g]	X	X		X		X	X		X	X	
Ophthalmology examination [h]	X	Perform every time tumor assessments are done							X	X	
Visual acuity testing [i]	X	Perform every time tumor assessments are done							X	X	
Tanner Stages [j]	X					C13D1 and C27D1	Once yearly				Once yearly
Vital signs incl. height and weight	X	X		X		X [k]	X		X	X	

**Assessment**	**Pre-Enrollment**	**Post-Enrollment**									
		**Cycle 1**					**Cycle 27 – EOT (± 3 days)**				
**Visit Day (Time Window)**	**Screening [a] (Pre-First Dose) (≤ 28 days)**	**Day 1 [b] (Pre-Dose)**	**Day 8 (± 3 days)**	**Day 15 (± 3 days)**	**Day 22 (± 3 days)**	**Cycles 2–26 (± 3 days)**	**On Drug Therapy [c]**	**Drug Holiday**	**EOT Visit (±7 days)**	**30-Day Post-EOT Safety Follow-Up (±14 days)**	**LTFU Every 3 ± 1 mo [d]**
12-lead ECG in triplicate [l]	X	X predose and with PK blood draws		X		C2D1, C4D1, and every 3rd cycle through C13 (C7D1, C10D1, and C13D1)			X		
Echocardiogram/MUGA [m]	X					C2D1, C4D1, and every 3rd cycle (C7D1, C10D1, etc.)	X		X		
Creatine phosphokinase	X					C2D1, C4D1 and every 4th cycle (C8D1, C12D1, etc.)	X		X		
Hematology and chemistry	X	X [n, o]		X		X [k]	X		X	X	

Assessment	Pre-Enrollment	Post-Enrollment									
		Cycle 1					Cycle 27 – EOT (± 3 days)				
Visit Day (Time Window)	Screening [a] (Pre-First Dose) (≤ 28 days)	Day 1 [b] (Pre-Dose)	Day 8 (± 3 days)	Day 15 (± 3 days)	Day 22 (± 3 days)	Cycles 2–26 (± 3 days)	On Drug Therapy [c]	Drug Holiday	EOT Visit (±7 days)	30-Day Post-EOT Safety Follow-Up (±14 days)	LTFU Every 3 ± 1 mo [d]
Pregnancy test by serum or urine (only for women of childbearing potential)	X	X [n]				X [k]	X	X	X	X	
Thyroid function test (TSH, T3, and T4) [p]	X	X [n]				C2D1, C3D1, and every other cycle (C5D1, C7D1, etc.)	X		X	X	
Tumor assessment: CT/MRI/PET	X					X [q]					X [d]
PK blood samples [r]		X		X		C2D1, C4D1, and every subsequent third cycle through C13 (C7D1, C10D1, and C13D1)					
Karnofsky/Lansky Performance Score	X	X		X		X [k]	X		X	X	

Assessment	Pre-Enrollment	Post-Enrollment									
		Cycle 1					Cycle 27 – EOT (± 3 days)				
Visit Day (Time Window)	Screening [a] (Pre-First Dose) (≤ 28 days)	Day 1 [b] (Pre-Dose)	Day 8 (± 3 days)	Day 15 (± 3 days)	Day 22 (± 3 days)	Cycles 2–26 (± 3 days)	On Drug Therapy [c]	Drug Holiday	EOT Visit (±7 days)	30-Day Post-EOT Safety Follow-Up (±14 days)	LTFU Every 3 ± 1 mo [d]
PedsQL-Core, PedsQL-Cancer, and PROMIS® [s]		X				C4D1 and every third cycle (C7D1, C10D1, etc.)	X		X		
Telephone follow-up			X		X						
Concomitant medication [t]	X										
Adverse events	X [u]										
Optional tissue collection [v]	X										
DAY101 dosing [w]		X									
DAY101 administration diary [x]		X							X		
DAY101 drug dispensation/return [y]		X							X		
Post-treatment anticancer medications											X
Survival status											X

Abbreviations: AE, adverse event; C, Cycle; D, Day; DOR, duration of response; ECG, electrocardiogram; ECHO, echocardiogram; eCRF, electronic case report form; EOT, end of treatment; FFPE, formalin-fixed paraffin embedded; ICF, informed consent form; LTFU, long-term follow-up; MRI, magnetic resonance imaging; MUGA, multiple-gated acquisition; OPG; optic pathway glioma; PedsQL-Cancer, Pediatric Quality of Life™—Cancer; PedsQL-Core, Pediatric Quality of Life Inventory™—Core; PK, pharmacokinetics; PROMIS®, Patient-Reported Outcomes Measurement Information System; SAE, serious adverse event; T3, triiodothyronine; T4, tetraiodothyronine; TSH, thyroid stimulating hormone.

[a] Results of screening assessments must be reviewed and approved before patient enrollment to confirm that the patient meets the eligibility criteria.

[b] Screening tests that are completed within the 48 hours prior to start of DAY101 and duplicate C1D1 tests will be accepted as fulfilling the C1D1 assessments. Repeat testing on C1D1 is not necessary for this scenario, and results should be recorded in the screening set of eCRFs.

[c] All assessments should be completed at C27D1 and D1 of every third cycle (e.g., C27D1, C30D1, C33D1, etc.) until the EOT visit.

[d] Only patients who discontinue treatment due to a reason other than disease progression will continue radiographic tumor assessments. Survival status and subsequent anticancer therapies will be collected for patients in LTFU; these data may be obtained by phone.

[e] Informed consent may be obtained more than 28 days prior to patient enrollment but must be provided before any study-specific procedures are performed; however, evaluations performed as part of routine care prior to informed consent can be used as screening evaluations if permitted by site Institutional Review Board/Ethics Committee policies and performed within protocol-specified windows.

[f] Tumor tissue (archival) will be obtained at enrollment whenever available. FFPE tumor blocks are preferred but in cases in which this is not possible, freshly cut FFPE slides should be obtained. See the Laboratory Manual for specific instructions. Fresh tissue biopsy is required during screening ONLY if archival tumor tissue sample is not available.

[g] All participants will have a neurological, physical, and dermatological examination during screening. For all subsequent visits, the examination will be symptom directed (at C1D1 examination should be completed before the first dose of study treatment).

[h] Screening ophthalmology examination is required for all patients. For patients with an underlying visual deficit related to the primary malignancy, assessment completed every time tumor assessments are done and at the EOT visit. The 30-day post-EOT safety follow up visit is required only if following for ocular toxicity. For all other patients, symptom-directed examinations may be completed as needed

[i] Screening visual acuity testing is required for all patients. For patients with an underlying visual deficit related to the primary malignancy, assessment completed every time tumor assessments are done and at the EOT visit. The 30-day post-EOT safety follow up visit is required only if following for ocular toxicity. For all other patients, symptom-directed examinations may be completed as needed.

[j] For patients > 8 years of age, pubertal stage will be measured before the start of treatment (screening) and yearly thereafter while on active treatment and during LTFU through sexual maturity.

[k] Assessment completed at D1 of every cycle.

[l] On C1D1, ECGs are to be collected predose and in conjunction with PK draws. When ECGs coincide with PK draw days after C1D1, ECGs should be performed after dosing but before the PK blood draw. Additional ECGs are to be performed if clinically indicated.

[m] Must use the same assessment (ECHO or MUGA) at screening throughout the study.

[n] This assessment is intended to confirm suitability for treatment after enrollment and prior to first dose on C1D1. If this assessment has been performed during screening within 48 hours (seven days for pregnancy test) prior to C1D1, this assessment does not need to be performed on C1D1 unless the patient’s clinical status has changed (e.g., onset of new symptoms indicative of clinical deterioration). If the assessment is performed on C1D1, the results must be reviewed by the investigator prior to any treatment being administered.

[o] See Section 6.7 and the Laboratory Manual for more detailed information on laboratory assessments.

[p] If clinically indicated (e.g. patients with thyroid cancer).

[q] Assessments will be performed at the end of C2 (i.e., on or up to seven days before C3D1), end of C4 (i.e., on or up to seven days before C5D1), and then at the end of every two cycles (on or up to seven days before Day 1 of the next cycle) through 12 months of treatment. Thereafter, patients will undergo radiographic evaluation at the end of every three cycles (on or up to seven days before Day 1 of the next cycle). Follow-up images can be limited to areas with known disease from baseline images. To ensure image consistency, the same imaging modalities and acquisition protocols used at screening are to be used for subsequent tumor assessments. Imaging is to be performed per the protocol-defined schedule regardless of whether study treatment is reduced, held, or discontinued. For patients who discontinue study treatment before radiographic progressive disease, every effort should be made to document progressive disease with subsequent imaging. Confirmation of progression is not required.

[r] See Section 6.8, Table 12 for PK sampling schedule.

[s] The PROMIS® questionnaire will only be administered to English-speaking patients enrolled in the U.S., Australia, and U.K.

[t] Document concomitant medication taken from 28 days prior to the planned C1D1 through the end of study participation. Only subsequent anticancer therapy(ies) will be recorded at LTFU.

[u] Refer to Section 8.1.1 for information on time period and frequency of collecting AE and SAE information. Note: only targeted SAEs will be collected beyond 30 days after last study drug dose.

[v] Participants who sign the optional ICF allowing for collection of tumor sample will have tumor specimen collected in the event a tumor-directed surgery occurs during study participation.

[w] DAY101 will be administered in clinic on C1D1 and at all PK blood draw visits. DAY101 will be taken once weekly at home otherwise until study treatment is discontinued.

[x] The study diary will be completed by the patient, or patient's parent or legal guardian, for every dose of study drug administered starting at C1D1. The Investigator or study coordinator should review diary entries for compliance, in particular for any potential missed doses, at every in-clinic or remote visit.

[y] DAY101 is to be dispensed to patients at D1 of every cycle, and any unused drug should be returned at the start of the following cycle. Study drug from last cycle should be returned at EOT visit. DAY101 can be dispensed at D1 of every third cycle starting at C27.

Appendix B: Karnofsky and Lansky Performance Status Scales

Table 18: Karnofsky Performance Score (≥ 16 years old)

Score	Karnofsky Description
100	Normal; no complaints; no evidence of disease
90	Able to carry on normal activity; minor signs or symptoms of disease
80	Normal activity with effort; some signs or symptoms of disease
70	Cares for self; unable to carry on normal activity or do active work
60	Requires occasional assistance, but is able to care for most personal needs
50	Requires considerable assistance and frequent medical care
40	Disabled; requires special care and assistance
30	Severely disabled; hospitalization is indicated, although death not imminent
20	Very sick; hospitalization necessary; active support treatment is necessary
10	Moribund; fatal processes progressing rapidly
0	Dead

(Karnofsky and Burchenal 1949)

Table 19: Lansky Performance Score (< 16 years old)

Score	Lansky Description
100	Fully active, normal
90	Minor restrictions in strenuous physical activity
80	Active, but tired more quickly
70	Greater restriction of play *and* less time spent in play activity
60	Up and around, but active play minimal; keeps busy by being involved in quieter activities
50	Lying around much of the day, but gets dressed; no active playing, participates in all quiet play and activities
40	Mainly in bed; participates in quiet activities
30	Bed bound; needing assistance even for quiet play
20	Sleeping often; play entirely limited to very passive activities
10	Doesn’t play; doesn’t get out of bed
0	Unresponsive

(Lansky et al. 1987)

Appendix C: List of Medications with Possible DAY101 CYP and BCRP Interactions

Table 20: List of Medications with Possible DAY101 CYP Interactions

CYP2C8 Inducers	CYP2C8 Inhibitors
paclitaxel<br>torsemide<br>amodiaquine<br>cerivastatin<br>repaglinide	gemfibrozil<br>montelukast

Abbreviations: CYP, cytochrome P450.

http://medicine.iupui.edu/clinpharm/ddis/table.asp.

Note: The above lists are not exhaustive. See also: http://www.fda.gov/drugs/developmentapprovalprocess/developmentresources/druginteractionslabeling/ucm 093664 htm.

Table 21: List of Medications with Possible DAY101 BCRP Interactions

BCRP Substrates	
**Class**	**Drug**
anthracenes	mitoxantrone
	bisantrene
	aza-anthrapyrazole (BBR3390)
camptothecin derivates	topotecan
	SN-38
	irinotecan
	diflomotecan
polyglutamates	methotrexate
	methotrexate-Glu2
	methotrexate-Glu3
nucleoside analogs	AZT
	AZT 5′-monophosphate
	lamivudine (3TC)

BCRP Substrates	
**Class**	**Drug**
other drugs	prazosin
	indolocarbazole
	flavopiridol
	canertinib (CI1033)
	imatinib mesylate (STI571)
	gefitinib (ZD1839)
	nilotinib
	glyburide
	cimetidine
	sulfasalazine
	nitrofurantoin
	rosuvastatin
	pantoprazole

Abbreviations: BCRP, breast cancer resistance protein.
(Mao et al. 2015)

Appendix D: Response Assessment in Neuro-Oncology (RANO) Criteria for Primary CNS Malignancies

The RANO criteria (Wen 2010) were developed to evaluate efficacy of investigational agents in glioblastoma clinical trials and have been more broadly used for lower grade primary CNS malignancies. These criteria were developed in part to address issues faced when assessing some lesions based on MacDonald criteria, particularly lesions with central necrosis and with a T2 component.

Table 22: RANO Response Criteria Incorporating MRI and Clinical Factor

RESPONSE CATEGORY	CRITERIA
Complete Response	• Disappearance of all measurable and nonmeasurable enhancing disease<br>• Stable or improved nonenhancing FLAIR/T2 lesions<br>• No new lesions<br>• Clinically stable or improved with no reliance on corticosteroids (except for physiological replacement)
Partial Response	• ≥ 50% decrease from baseline of all measurable enhancing lesions<br>• No progression of nonmeasurable disease<br>• Stable or improved nonenhancing FLAIR/T2 lesions<br>• No new lesions<br>• Clinically stable or improved, with stable or reduced corticosteroids compared to baseline
Progressive Disease	• ≥ 25% increase from baseline in enhancing lesions despite stable or increasing steroid dose<br>• Significant increase in nonenhancing FLAIR/T2 lesions not attributable to other nontumor causes<br>• Any new lesions<br>• Clinical deterioration no attributable to other nontumor causes and not due to steroid decrease
Stable Disease	• Does not meet other criteria for response or progression<br>• Stable nonenhancing FLAIR/T2 lesions<br>• Clinically stable with stable or reduced corticosteroids compared to baseline

Abbreviations: FLAIR/T2, T2-weighted fluid-attenuated inversion recovery; MRI, magnetic resonance imaging; RANO, Response Assessment in Neuro-Oncology.

A measurable lesion is evaluated by contrast-enhancing MRI and:

- Has clearly defined margins
- Is visible on 2 or more axial slices, preferably < 5 mm thick
- Is at least 10 mm in size if slice thickness is < 5 mm (or 2× slice thickness if > 5 mm)
- Does not measure a cystic cavity

Non-measurable lesions are those that do not fit the criteria above, and specifically lesions that are cystic, necrotic, or include a surgical cavity should not be considered measurable.

Measurements are calculated by summing the products of perpendicular diameters of all measurable enhancing lesions.

If there are multiple contrast-enhancing lesions, a minimum of the two largest lesions should be measured. However, emphasis should be placed on selecting lesions that allow reproducible repeated measurements. For patients who have multiple lesions for which only one or two are increasing in size, the enlarging lesions should be considered the measurable lesions for evaluation of response.

Appendix E: Response Evaluation Criteria in Solid Tumors: Revised RECIST Guideline (version 1.1)

A revised RECIST guideline (version 1.1) was developed by the RECIST Working Group (Eisenhauer et al. 2009). Notable changes are highlighted below:

- Number of lesions required to assess tumor burden for response determination has been reduced from a maximum of 10 to a maximum of five total (and from five to two per organ, maximum).
- Nodes with a short axis of 15 mm are considered measurable and assessable as target lesions. Nodes that shrink to < 10 mm short axis are considered normal.
- In addition to the previous definition of progression in target disease of 20% increase in sum, a 5-mm absolute increase is now required as well.

Measurability of Tumor Lesions at Baseline

Tumor lesions: Must be accurately measured in at least one dimension (longest diameter in the plane of measurement is to be recorded) with a minimum size of:

- 10 mm by CT scan (CT scan slice thickness no greater than 5 mm) and 20 mm by chest X-ray
- 10-mm caliper measurement by clinical exam (lesions which cannot be accurately measured with calipers should be recorded as non-measurable).

Malignant lymph nodes: To be considered pathologically enlarged and measurable, a lymph node must be ≥ 15 mm in short axis when assessed by CT scan (CT scan slice thickness recommended to be no greater than 5 mm). At baseline and in follow-up, only the short axis will be measured and followed.

Special considerations regarding lesion measurability of bone lesions, cystic lesions, and lesions previously treated with local therapy can be found in RECIST version 1.1 and are briefly summarized as follows:

- Lytic bone lesions or mixed lytic-blastic lesions, with identifiable soft tissue components, that can be evaluated by cross sectional imaging techniques such as CT or MRI can be considered as measurable lesions if the soft tissue component meets the definition of measurability described above.
- "Cystic lesions" thought to represent cystic metastases can be considered as measurable lesions if they meet the definition of measurability described above.
- Tumor lesions situated in a previously irradiated area, or in an area subjected to other loco-regional therapy, are usually not considered measurable unless there has been demonstrated progression in the lesion.

Specifications by Methods of Measurements

The same method of assessment and the same technique should be used to characterize each identified and reported lesion at baseline and during follow-up. Imaging based evaluation

should always be done rather than clinical examination unless the lesion(s) being followed cannot be imaged but are assessable by clinical exam.

Clinical lesions: Clinical lesions will only be considered measurable when they are superficial and ≥10mm diameter as assessed using calipers (e.g. skin nodules). For the case of skin lesions, documentation by color photography including a ruler to estimate the size of the lesion is suggested.

Chest X-ray: Chest CT is preferred over chest X-ray, particularly when progression is an important endpoint, since CT is more sensitive than X-ray, particularly in identifying new lesions. However, lesions on chest X-ray may be considered measurable if they are clearly defined and surrounded by aerated lung.

CT, MRI: CT is the best currently available and reproducible method to measure lesions selected for response assessment. This guideline has defined measurability of lesions on CT scan based on the assumption that CT slice thickness is ≤ 5mm.

MRI is also acceptable in certain situations (e.g. for body scans).

Use of ultrasound, endoscopy, laparoscopy, biomarker, and cytology/histology are not recommended for additional details refer to RECIST v1.1.

Tumor Response Evaluation

To assess objective response or future progression, it is necessary to estimate the overall tumor burden at baseline and use this as a comparator for subsequent measurements. Measurable disease is defined by the presence of at least one measurable lesion.

A maximum of five lesions total (and a maximum of two lesions per organ) representative of all involved organs should be identified as target lesions and will be recorded and measured at baseline. A sum of the diameters (longest for non-nodal lesions, short axis for nodal lesions) for all target lesions will be calculated and reported as the baseline sum diameters. All other lesions (or sites of disease) including pathological lymph nodes should be identified as non-target lesions and should also be recorded at baseline. Measurements are not required and these lesions should be followed as “present,” “absent,” or in rare cases “unequivocal progression.”

<u>Evaluation of Target Lesions</u>

- Complete Response (CR): Disappearance of all target lesions. Any pathological lymph nodes (whether target or non-target) must have reduction in short axis to < 10 mm.
- PR: At least a 30% decrease in the sum of diameters of target lesions, taking as reference the baseline sum diameters.

- Progressive Disease (PD): At least a 20% increase in the sum of diameters of target lesions, taking as reference the smallest sum on study (this includes the baseline sum if that is the smallest on study). In addition to the relative increase of 20%, the sum must also demonstrate an absolute increase of at least 5 mm. (Note: the appearance of one or more new lesions is also considered progression).
- Stable Disease (SD): Neither sufficient shrinkage to qualify for PR nor sufficient increase to qualify for PD, taking as reference the smallest sum diameters while on study.

Evaluation of Non-Target Lesions

While some non-target lesions may actually be measurable, they need not be measured and instead should be assessed only qualitatively at the time points specified in the protocol.

- Complete Response (CR): Disappearance of all non-target lesions and normalization of tumor marker level. All lymph nodes must be non-pathological in size (<10 mm short axis).
- Non-CR/Non-PD: Persistence of one or more non-target lesion(s) and/or maintenance of tumor marker level above the normal limits.
- Progressive Disease (PD): Unequivocal progression (see comments below) of existing non-target lesions. (Note: the appearance of one or more new lesions is also considered progression).

Evaluation of Best Overall Response

The BOR is determined once all the data for the patient is known. Guidance on the determination of tumor response at each time point is provided in Table 23. Complete or partial responses may be claimed only if the criteria for each are met at a subsequent time point as specified in the protocol (generally four weeks later). Guidance on the determination of best overall confirmed response is provided in Table 24.

Patients with a global deterioration of health status requiring discontinuation of treatment without objective evidence of disease progression at that time should be classified as having symptomatic deterioration. Every effort should be made to document objective disease progression, even after discontinuation of treatment.

In some circumstances, it may be difficult to distinguish residual disease from normal tissue. When the evaluation of CR depends on this determination, it is recommended that the residual lesion be investigated, i.e., biopsied if accessible.

Table 23: Overall Assessment by RECIST v1.1 at Each Time Point

Target Lesion	Non-Target Lesions	New Lesions	Overall Response
CR	CR or none at BL	No	CR
CR	Non-CR/Non-PD	No	PR
CR	Not all evaluated (NE)	No	PR
PR	Non-PD or not all evaluated or none at BL	No	PR
SD	Non-PD or not all evaluated or none at BL	No	SD
Not all evaluated	Non-PD	No	NE
PD	Any	Yes or No	PD
Any	PD	Yes or No	PD
Any	Any	Yes	PD
None identified at BL	CR	No	CR
None identified at BL	Non-CR/Non-PD	No	Non-CR/Non-PD
None identified at BL	Not all evaluated	No	NE
None identified at BL	PD	Yes or No	PD
None identified at BL	None identified at BL	Yes	PD

Abbreviations: BL, baseline; CR, complete response; NE, not all evaluated; PD, progressive disease; PR, partial response; SD, stable disease.

Table 24: Best Overall Assessment by RECIST v1.1

Overall Response First Time Point	Overall Response Subsequent Time Point	Best Confirmed Overall Response
CR	CR	CR
CR	PR	PR[a], SD, PD
CR	SD	SD provided minimum criteria for SD duration met, otherwise, PD
CR	PD	SD provided minimum criteria for SD duration met, otherwise, PD
CR	NE	SD provided minimum criteria for SD duration met, otherwise, NE
PR	CR	PR
PR	PR	PR
PR	SD	SD
PR	PD	SD provided minimum criteria for SD duration met, otherwise, PD
PR	NE	SD provided minimum criteria for SD duration met, otherwise, NE
NE	NE	NE

Abbreviations: CR, complete response; NE, not all evaluated; PD, progressive disease; PR, partial response; SD, stable disease.

[a] If a CR is truly met at first time point, then any disease seen at a subsequent time point, even disease meeting PR criteria relative to baseline, makes the disease PD at that point (since disease must have reappeared after CR). Best response would depend on whether minimum duration for SD was met. However, sometimes ‘CR’ may be claimed when subsequent scans suggest small lesions were likely still present and in fact the patient had PR, not CR at the first time point. Under these circumstances, the original CR should be changed to PR and the best response is PR.

Appendix F: Specimen and Data Use

Research Uses of Tissue and Clinical Data

Any leftover tissue samples will be banked for future analyses that are not yet available. Clinical data and tissue samples, independently and together, will be used for nontherapeutic research that advances the diagnosis, evaluation, etiology, prevention, and outcome improvement of nervous system diseases. Research on tissue samples may include detailed genomic and molecular analysis. In general, these findings will be linked to clinical care and outcome to maximize research findings. Studies may include tumor biology studies, biomarker identification studies, drug target studies, genomics and proteomics studies, genetic susceptibility studies, drug development efforts, epidemiological studies, and outcomes studies. Use of leftover tissue samples is optional and can only be done with the consent of the patient.

Retrospective analysis on clinical data performed outside of prospective formal clinical trial data will be linked with outcomes to create research findings and provide preliminary data to support further investigations.

Commercial Uses of Tissue and Clinical Data

Tissue samples collected and/or stored in the bank may be made available to commercial or corporate scientific collaborators only as part of a scientific collaboration. Derivative products from the patient tissues, including diagnostics tests or other items, may arise from study of these tissues. Patients will not have any ownership or financial benefit from their tissue samples and derivatives.

Uses That May Affect Determination of Treatment (Clinical Care) and/or Eligibility in Clinical Trials

In rare circumstances, findings from tissue testing may be clinically relevant and affect treatment decisions. All testing results communicated back to the patient’s treating clinician and/or placed into the patient’s medical record must be CLIA compliant. If the testing is first done in a laboratory that does not meet these criteria, yet the results are felt to be clinically significant, the testing will be repeated in a CLIA environment prior to informing the patient’s clinician and/or placement in the patient’s medical record. The patient’s treating clinician is responsible for treatment decisions including whether and how to inform the patient of any results.

Appendix G: Blood Pressure by Height and Age

The tables for normal blood pressure (BP) by height and age are included in this appendix. In order to use these tables, note that the height percentile is determined by the standard growth charts. The child's measured systolic and diastolic BP is compared with the numbers provided in the table (boys or girls) for age and height percentile.

- The child is normotensive if BP is below the 90th percentile.
- If the child's BP (systolic or diastolic) is at or above the 95th percentile, the child may be hypertensive, and repeated measurements are indicated.
- BP measurements between the 90th and 95th percentiles are high normal and warrant further observation and consideration of other risk factors.

Standards for systolic and diastolic BP for infants younger than 1 year are available in the second task force report. Recently, additional data have been published. In children younger than 1 year, systolic BP has been used to define hypertension.

Source: DHHS, PHS, NIH, National Heart, Lung, and Blood Institute. Update on the Task Force Report (1987) on High Blood Pressure in Children and Adolescents: A Working Group Report from the National High Blood Pressure Education Program. NIH Publication 96-3790; 1996; 7-9.

The entire NIH document can be viewed at:
http://www.nhlbi.nih.gov/health/prof/heart/hbp/hbp_ped.pdf.

90th and 95th Percentile BP for Girls 1-17 Years

Age (y)	Blood Pressure Percentile	Systolic Blood Pressure by Percentile of Height (mm Hg)							Diastolic Blood Pressure by Percentile of Height (mm Hg)						
		5%	10%	25%	50%	75%	90%	95%	5%	10%	25%	50%	75%	90%	95%
1	90th	94	95	97	98	100	102	102	50	51	52	53	54	54	55
	95th	98	99	101	102	104	106	106	55	55	56	57	58	59	59
2	90th	98	99	100	102	104	105	106	55	55	56	57	58	59	59
	95th	101	102	104	106	108	109	110	59	59	60	61	62	63	63
3	90th	100	101	103	105	107	108	109	59	59	60	61	62	63	63
	95th	104	105	107	109	111	112	113	63	63	64	65	66	67	67
4	90th	102	103	105	107	109	110	111	62	62	63	64	65	66	66
	95th	106	107	109	111	113	114	115	66	67	67	68	69	70	71
5	90th	104	105	106	108	110	112	112	65	65	66	67	68	69	69
	95th	108	109	110	112	114	115	116	69	70	70	71	72	73	74
6	90th	105	106	108	110	111	113	114	67	68	69	70	70	71	72
	95th	109	110	112	114	115	117	117	72	72	73	74	75	76	76
7	90th	106	107	109	111	113	114	115	69	70	71	72	72	73	74
	95th	110	111	113	115	116	118	119	74	74	75	76	77	78	78
8	90th	107	108	110	112	114	115	116	71	71	72	73	74	75	75
	95th	111	112	114	116	118	119	120	75	76	76	77	78	79	80
9	90th	109	110	112	113	115	117	117	72	73	73	74	75	76	77
	95th	113	114	116	117	119	121	121	76	77	78	79	80	80	81
10	90th	110	112	113	115	117	118	119	73	74	74	75	76	77	78
	95th	114	115	117	119	121	122	123	77	78	79	80	80	81	82
11	90th	112	113	115	117	119	120	121	74	74	75	76	77	78	78
	95th	116	117	119	121	123	124	125	78	79	79	80	81	82	83
12	90th	115	116	117	119	121	123	123	75	75	76	77	78	78	79
	95th	119	120	121	123	125	126	127	79	79	80	81	82	83	83
13	90th	117	118	120	122	124	125	126	75	76	76	77	78	79	80
	95th	121	122	124	126	128	129	130	79	80	81	82	83	83	84
14	90th	120	121	123	125	126	128	128	76	76	77	78	79	80	80
	95th	124	125	127	128	130	132	132	80	81	81	82	83	84	85
15	90th	123	124	125	127	129	131	131	77	77	78	79	80	81	81
	95th	127	128	129	131	133	134	135	81	82	83	83	84	85	86
16	90th	125	126	128	130	132	133	134	79	79	80	81	82	82	83
	95th	129	130	132	134	136	137	138	83	83	84	85	86	87	87
17	90th	128	129	131	133	134	136	136	81	81	82	83	84	85	85
	95th	132	133	135	136	138	140	140	85	85	86	87	88	89	89

90th and 95th Percentile BP For Boys 1-17 Years

Age (y)	Blood Pressure Percentile	Systolic Blood Pressure by Percentile of Height (mm Hg)							Diastolic Blood Pressure by Percentile of Height (mm Hg)						
		5%	10%	25%	50%	75%	90%	95%	5%	10%	25%	50%	75%	90%	95%
1	90th	94	95	97	98	100	102	102	50	51	52	53	54	54	55
	95th	98	99	101	102	104	106	106	55	55	56	57	58	59	59
2	90th	98	99	100	102	104	105	106	55	55	56	57	58	59	59
	95th	101	102	104	106	108	109	110	59	59	60	61	62	63	63
3	90th	100	101	103	105	107	108	109	59	59	60	61	62	63	63
	95th	104	105	107	109	111	112	113	63	63	64	65	66	67	67
4	90th	102	103	105	107	109	110	111	62	62	63	64	65	66	66
	95th	106	107	109	111	113	114	115	66	67	67	68	69	70	71
5	90th	104	105	106	108	110	112	112	65	65	66	67	68	69	69
	95th	108	109	110	112	114	115	116	69	70	70	71	72	73	74
6	90th	105	106	108	110	111	113	114	67	68	69	70	70	71	72
	95th	109	110	112	114	115	117	117	72	72	73	74	75	76	76
7	90th	106	107	109	111	113	114	115	69	70	71	72	72	73	74
	95th	110	111	113	115	116	118	119	74	74	75	76	77	78	78
8	90th	107	108	110	112	114	115	116	71	71	72	73	74	75	75
	95th	111	112	114	116	118	119	120	75	76	76	77	78	79	80
9	90th	109	110	112	113	115	117	117	72	73	73	74	75	76	77
	95th	113	114	116	117	119	121	121	76	77	78	79	80	80	81
10	90th	110	112	113	115	117	118	119	73	74	74	75	76	77	78
	95th	114	115	117	119	121	122	123	77	78	79	80	80	81	82
11	90th	112	113	115	117	119	120	121	74	74	75	76	77	78	78
	95th	116	117	119	121	123	124	125	78	79	79	80	81	82	83
12	90th	115	116	117	119	121	123	123	75	75	76	77	78	78	79
	95th	119	120	121	123	125	126	127	79	79	80	81	82	83	83
13	90th	117	118	120	122	124	125	126	75	76	76	77	78	79	80
	95th	121	122	124	126	128	129	130	79	80	81	82	83	83	84
14	90th	120	121	123	125	126	128	128	76	76	77	78	79	80	80
	95th	124	125	127	128	130	132	132	80	81	81	82	83	84	85
15	90th	123	124	125	127	129	131	131	77	77	78	79	80	81	81
	95th	127	128	129	131	133	134	135	81	82	83	83	84	85	86
16	90th	125	126	128	130	132	133	134	79	79	80	81	82	82	83
	95th	129	130	132	134	136	137	138	83	83	84	85	86	87	87
17	90th	128	129	131	133	134	136	136	81	81	82	83	84	85	85
	95th	132	133	135	136	138	140	140	85	85	86	87	88	89	89

Appendix H: Rash Management

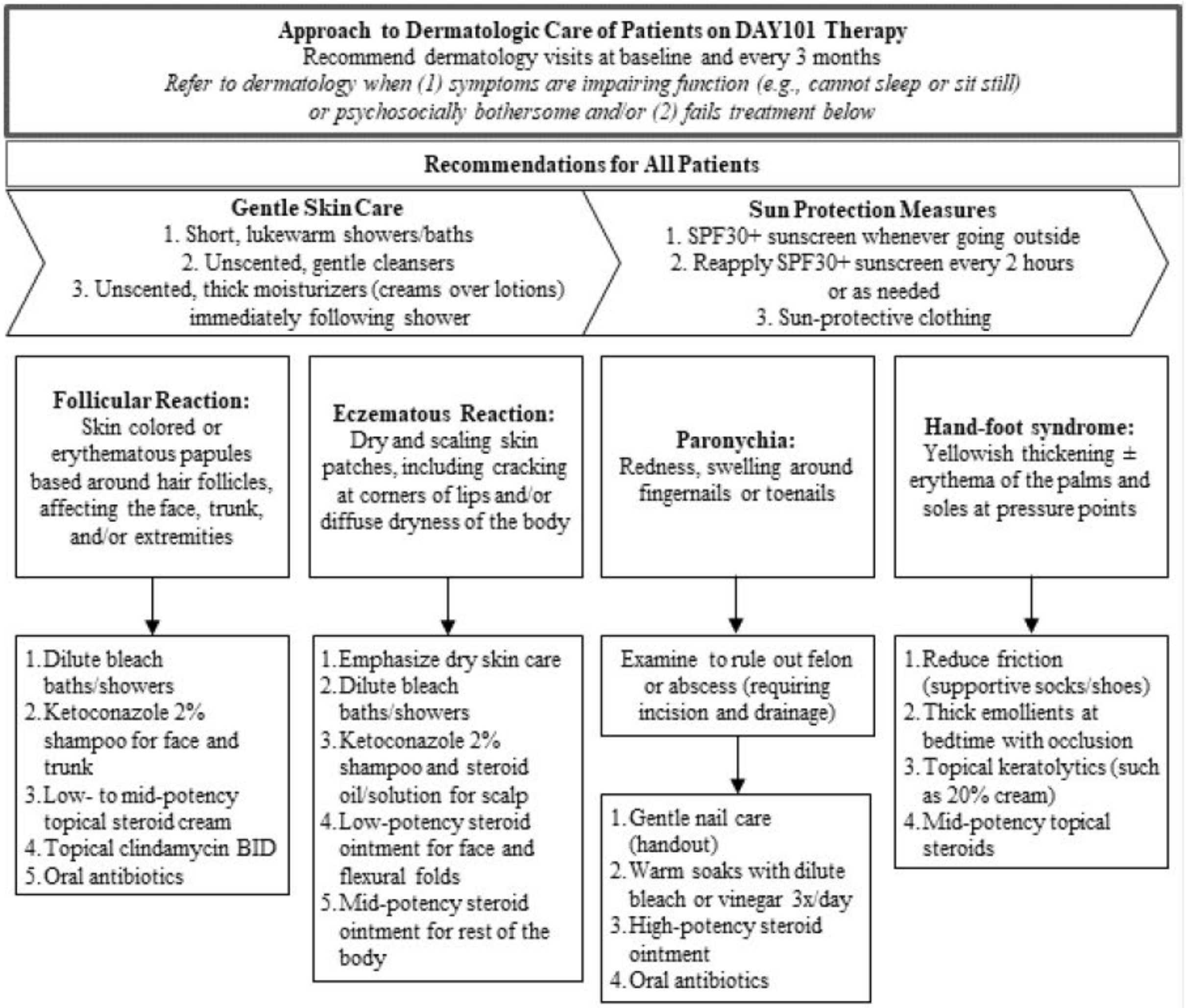

Guidelines for Rash/Dermatitis adapted from Lemech and Arkenau 2012.

Grade 1

Macular or papular eruption or erythema without associated symptoms

Grade 2

Macular or papular eruption or erythema with pruritus or other associated symptoms; localized desquamation or other lesions covering < 50% of body surface area (BSA)

- *Consider dose reduction by one dose level once weekly after discussion with the investigator.*

Grade 3 or higher

Severe, generalized erythroderma or macular, papular, or vesicular eruption, desquamation covering ≥ 50% BSA
Generalized exfoliative, ulcerative, or bullous dermatitis

- *DAY101 should be delayed until the rash improves.*
- *A dermatologist should be consulted; a biopsy could be considered for rash characterization.*
- *Consider dose reduction by two dose levels or holding administration of DAY101 until resolved to Grade 1 or baseline.*

Note: Should a Grade 2 or 3 rash occur, photographic documentation is recommended.

DILUTE BLEACH BATH INSTRUCTIONS

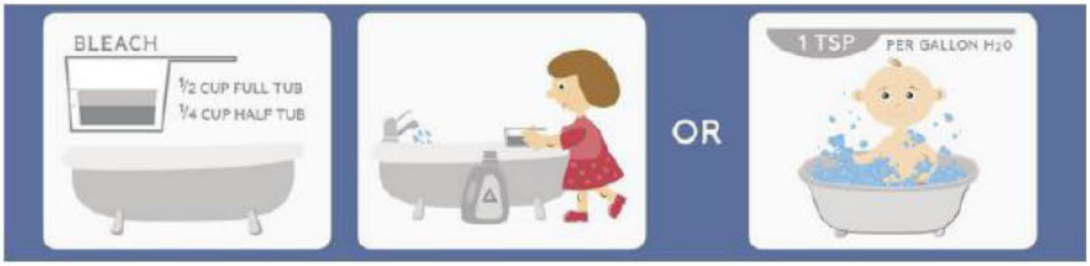

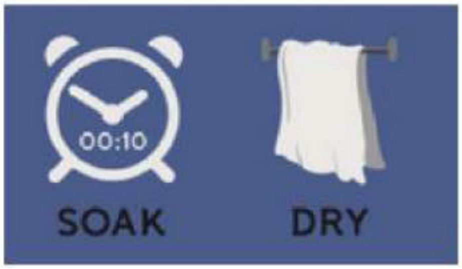

Once every other day:

1. Fill your bathtub ½ full with warm water
2. Place ¼ cup Clorox Bleach into bath water
3. Soak for 10 minutes
4. Gently pat skin dry following bath
5. Apply a gentle moisturizer

Appendix I: Visual Acuity Testing

Visual acuity testing using the appropriate age-specific testing methodology will be performed for all patients at screening. For patients with OPG or an underlying visual deficit related to the primary malignancy, visual acuity testing will be performed every time patients have a radiographic disease assessment. A forced choice preferential looking test is preferred. Testing may be done by an ophthalmologist or other qualified site clinical personnel. To reduce variability in scoring, the same BCVA testing methodology should be used throughout the treatment period. Best corrected visual acuity will be reported in logMAR. Reasons for BCVA testing not being completed will be recorded.

Visual Acuity in Feet and logMAR Equivalents for Acuity in Cycles/cm

TAC II @ 55 cm (Cycles/cm)	VA equivalent	logMAR
26	20/24	0.06
19	20/32	0.20
13	20/47	0.36
9.8	20/63	0.49
6.5	20/94	0.66
4.8	20/130	0.80
3.2	20/190	0.97
2.4	20/260	1.10
1.6	20/380	1.27
1.3	20/470	1.36
0.86	20/710	1.54
0.64	20/960	1.67
0.43	20/1400	1.84
0.32	20/1900	1.97

Abbreviations: logMAR, logarithm of the minimum angle of resolution; TAC, Teller Acuity Cards®; VA, visual acuity.

HOTV or other ETDRS assessments should be attempted on patients who are old enough, based on local standard practice. Assessments will be performed in each eye separately at a recommended testing distance of 3 meters (or local standard practice). Best corrected visual acuity will be reported in logMAR.

Reasons for VA testing not being completed will be recorded (e.g., bilateral blindness with no light perception).

NOTE: A 0.2 logMAR worsening (or up to a two-card drop in Teller acuity) will not be considered visual progressive disease at the first on-therapy staging evaluation (at 12 weeks) if the tumor is stable or smaller on the MRI. In these cases, a repeat VA assessment will be required six weeks later to confirm that further visual progression is not occurring. If the repeat VA is stable or improved (0.2 logMAR or less [or a two-card drop in Teller acuity or less] from pretreatment baseline), the patient will remain on protocol therapy.

VA Norms

Note: The rate of visual development varies across patients. The table is presented as a reference only.

Age	Grating Acuity by TAC II			Recognition Acuity	
	Normal VA (cy/cm)	logMAR	Snellen Equivalent [b]	Normal VA (feet)	logMAR
6 m	2.4	1.097	20/260	—	—
12 m	3.2	0.972	20/190	—	—
18 m	4.8	0.796	20/130	—	—
24 m	4.8	0.796	20/130	—	—
30 m	6.5	0.664	20/94	—	—
36 m	9.8	0.486	20/63	—	—
42 m	13.0	0.363	20/47	—	—
48 m	—	—	—	20/32	0.2
5 y	—	—	—	20/25	0.10
≥ 6 y	—	—	—	20/20	0.00

Abbreviations: CI, confidence interval; logMAR, logarithm of the minimum angle of resolution; TAC, Teller Acuity Cards®; VA, visual acuity.
[a] Normal VA with TAC [low limit of normal at 95% CI]
[b] Conversion of grating acuity to Snellen equivalents is not recommended and is only provided for purposes of communication.

Visual Fields:

Visual field testing will be performed unilaterally by confrontation and reported by capturing the corresponding quadrant(s) with a deficit (1, 2, 3, or 4) .

Optic Disc Appearance:

Optic discs will be assessed unliterally for the presence or absence of pallor and edema.

Appendix J: Adverse Events. Definitions and Procedures for Recording, Evaluating, Follow-Up, and Reporting

**Adverse Event Definition**
• An adverse event (AE) is any untoward medical occurrence in a patient or clinical study patient, temporally associated with the use of study intervention, whether or not considered related to the study intervention.<br>• NOTE: An AE can therefore be any unfavorable and unintended sign (including an abnormal laboratory finding), symptom, or disease (new or exacerbated) temporally associated with the use of study intervention.<br>• The investigator is responsible for ensuring that any AEs observed by the investigator or reported by the patient are recorded in the patient's medical record.

**Events Meeting the AE Definition**
• Any abnormal laboratory test results (hematology, clinical chemistry, or urinalysis) or other safety assessments (e.g., electrocardiogram [ECG], radiological scans, vital signs measurements), including those that worsen from baseline, considered clinically significant in the medical and scientific judgment of the investigator (i.e., not related to progression of underlying disease). **In general, abnormal laboratory findings without clinical significance (based on the investigator's judgment) are not to be recorded as AEs. However, abnormal laboratory findings that result in new or worsening clinical sequelae, require therapy, or adjustment in current therapy are considered AEs. Where applicable, clinical sequelae (not the laboratory abnormality) are to be recorded as the AE.** If the clinically significant laboratory abnormality is a sign of a disease or syndrome (e.g., alkaline phosphatase and bilirubin > 5 × upper limit of normal [ULN] associated with cholecystitis), only the diagnosis (e.g., cholecystitis) needs to be recorded on the Adverse Event electronic Case Report Form (eCRF).<br>• Exacerbation of a chronic or intermittent preexisting condition, including either an increase in frequency and/or intensity of the condition<br>• New conditions detected or diagnosed after study intervention administration, even though it may have been present before the start of the study<br>• Signs, symptoms, or the clinical sequelae of a suspected drug-drug interaction<br>• Signs, symptoms, or the clinical sequelae of a suspected overdose of either study intervention or a concomitant medication. Overdose per se will not be reported as an AE/serious adverse event (SAE) unless it is an intentional overdose taken with possible suicidal/self-harming intent. Such overdoses should be reported regardless of sequelae.<br>- Lack of efficacy will not be reported as an AE or SAE. Such instances will be captured in the efficacy assessments. Nonetheless, the signs, symptoms, and/or clinical sequelae resulting from lack of efficacy will be reported as AEs or SAEs if they fulfill the definition of an AE or SAE.<br>- The term "disease progression" or "progressive disease" should not be reported as an AE, rather, report the specific malignancy as the AE term.

**Events <u>NOT</u> Meeting the AE Definition**
• Any clinically significant abnormal laboratory findings or other abnormal safety assessments that are associated with the underlying disease, unless judged by the investigator to be more severe than expected for the patient's condition<br>• The disease/disorder being studied or expected progression, signs, or symptoms of the disease/disorder being studied, unless more severe than expected for the patient's condition<br>• Interventions for pretreatment conditions (e.g., elective cosmetic surgery) or medical procedures that were planned before study participation (e.g., port placement)<br>• Unplanned medical or surgical procedures (e.g., endoscopy, appendectomy). The condition that leads to the procedure is the AE.<br>• Situations in which an untoward medical occurrence did not occur (social, logistical, and/or convenience admission to a hospital)<br>• Anticipated day-to-day fluctuations of preexisting disease(s) or condition(s) present or detected at the start of the study that do not worsen

Definition of SAE

If an event is not an AE per definition above, then it cannot be an SAE, even if serious conditions are met (e.g., hospitalization for signs/symptoms of the disease under study, death due to progression of disease).

**An SAE is defined as any untoward medical occurrence that, at any dose:**
**a. Results in death**
**b. Is life-threatening**<br>The term "life-threatening" in the definition of "serious" refers to an event in which the patient was at risk of death at the time of the event. It does not refer to an event that hypothetically might have caused death, if it were more severe.
**c. Requires inpatient hospitalization or prolongation of existing hospitalization**<br>• In general, hospitalization signifies that the patient has been detained (usually involving at least an overnight stay) at the hospital or emergency ward for observation and/or treatment that would not have been appropriate in the physician's office or outpatient setting. Complications that occur during hospitalization are AEs. If a complication prolongs hospitalization or fulfills any other serious criteria, the event is serious. When in doubt as to whether hospitalization occurred or was necessary, the AE should be considered serious.<br>• Events that require an escalation of care when the patient is already hospitalized should be recorded as an SAE. Examples of such events include movement from routine care in the hospital to the intensive care unit or if that event resulted in a prolongation of the existing planned hospitalization.<br>• Hospitalization for conditioning chemotherapy, infusion, and monitoring as required by the protocol and elective treatment of a preexisting condition that did not worsen from baseline is not considered an AE.

	• Planned admissions to hospital for administration of DAY101 or other protocol-mandated procedures do not qualify as serious unless a new AE occurs that results in prolongation or meets any of the other "serious" criteria.
**d.**	**Results in persistent disability/incapacity**<br>• The term "disability" means a substantial disruption of a person's ability to conduct normal life functions.<br>• This definition is not intended to include experiences of relatively minor medical significance, such as uncomplicated headache, nausea, vomiting, diarrhea, influenza, and accidental trauma (e.g., sprained ankle) that may interfere with or prevent everyday life functions but do not constitute a substantial disruption.
**e.**	**Is a congenital anomaly/birth defect**
**f.**	**Other situations:**<br>• Medical or scientific judgment should be exercised in deciding whether SAE reporting is appropriate in other situations, such as important medical events that may not be immediately life-threatening or result in death or hospitalization but may jeopardize the patient or may require medical or surgical intervention to prevent one of the other outcomes listed in the above definition. These events should usually be considered serious.<br>• Examples of such events include invasive or malignant cancers, intensive treatment in an emergency room or at home for allergic bronchospasm, blood dyscrasias, or convulsions that do not result in hospitalization, or development of drug dependency or drug abuse.

Recording and Follow-Up of AE and/or SAE

AE and SAE Recording
• When an AE/SAE occurs, it is the responsibility of the investigator to review all documentation (e.g., hospital progress notes, laboratory reports, and diagnostics reports) related to the event.<br>• The investigator will then record all relevant AE/SAE information in the eCRF.<br>• It is not acceptable for the investigator to send photocopies of the patient's medical records to the Sponsor in lieu of completion of the AE/SAE eCRF page.<br>• There may be instances when copies of medical records for certain cases are requested by Sponsor. In this case, all patient identifiers, with the exception of the patient number, will be redacted on the copies of the medical records before submission to Sponsor.<br>• **The investigator will attempt to establish a diagnosis of the event based on signs, symptoms, and/or other clinical information.** Whenever possible, the diagnosis (not the individual signs/symptoms) will be documented as the AE/SAE (e.g., record liver failure or hepatitis rather than jaundice, asterixis, and elevated transaminases). In addition, AEs occurring secondary to other events (e.g., cascade events or clinical sequelae) should be identified by their primary cause (e.g., if diarrhea is known to have resulted in dehydration, then it is sufficient to record only diarrhea as an AE on the eCRF).

Assessment of Causality

- The investigator is obligated to assess the relationship between study intervention (DAY101) and each occurrence of each AE/SAE.
- A "reasonable possibility" of a relationship conveys that there are facts, evidence, and/or arguments to suggest a causal relationship, rather than that a relationship cannot be ruled out.
- The investigator will use clinical judgment to determine the relationship.
- Alternative causes, such as underlying disease(s), concomitant therapy, and other risk factors, as well as the temporal relationship of the event to study intervention administration, will be considered and investigated.
- The investigator will also consult the Investigator's Brochure (IB) and/or Product Information for marketed products in his/her assessment.
- For each AE/SAE, the investigator must document in the medical notes that he/she has reviewed the AE/SAE and has provided an assessment of causality.
- There may be situations in which an SAE has occurred, and the investigator has minimal information to include in the initial report to the Sponsor's Drug Safety Group. **However, it is very important that the investigator always make an assessment of causality for every event before the initial transmission of the SAE data to the Sponsor's Drug Safety Group or designees.**
- The investigator may change his/her opinion of causality in light of follow-up information and send an SAE follow-up report with the updated causality assessment.
- The causality assessment is one of the criteria used when determining regulatory reporting requirements.

In reviewing AEs, investigators must assess whether the AE is possibly related to 1) DAY101, 2) any protocol-required study procedure (e.g., biopsy). The relationship is indicated by a yes or no response and entered in the eCRF. A yes response should indicate a causal relationship between medicinal product and an AE is at least a reasonable possibility. The AE cannot reasonably be explained by the subject's clinical state, concomitant medical condition or concomitant therapies, and a temporal relationship exists between the event onset and administration of the study drug.

Follow-Up of AEs and SAEs

- The investigator is obligated to perform or arrange for the conduct of supplemental measurements and/or evaluations as medically indicated or as requested by the Sponsor's Drug Safety Group or designee to elucidate the nature and/or causality of the AE or SAE as fully as possible. This may include additional laboratory tests or investigations, histopathological examinations, or consultation with other health-care professionals.
- If a patient dies during participation in the study or during a recognized follow-up period, the investigator will provide the Sponsor with a copy of any post-mortem findings, including histopathology, as applicable.
- The investigator will submit any updated SAE data to Sponsor within 24 hours of receipt of the information.

Reporting of SAEs

SAE Reporting to Sponsor via the SAE Report Form
The investigator is responsible for ensuring that AEs are monitored and reported. See Section 8.1.1 for greater details regarding the AE and SAE reporting periods.<br>• Report SAEs by completing the paper SAE (see next section) in order to report the event within 24 hours.<br>• Contacts for SAE reporting can be found in the Study Manual.<br>• In rare circumstances and in the absence of e-mail or facsimile equipment, notification by telephone is acceptable with a copy of the SAE data collection tool sent by overnight mail or courier service.<br>• Initial notification via telephone does not replace the need for the investigator to complete and sign the SAE Report Form within the designated reporting time frames.

Appendix K: Contraceptive Guidance and Collection of Pregnancy Information

Definitions:

Woman of Childbearing Potential (WOCBP)

A woman is considered to be fertile following menarche and until becoming postmenopausal, unless permanently sterile (see below).

If fertility is unclear (e.g., amenorrhea in adolescents or athletes) and a menstrual cycle cannot be confirmed before first dose of study intervention, additional evaluation should be considered.

Women in the following categories are not considered WOCBPs:

- Premenarchal
- Premenopausal woman with one of the following:
  - Documented hysterectomy
  - Documented bilateral salpingectomy
  - Documented bilateral oophorectomy
  - For individuals with permanent infertility due to an alternate medical cause other than the above (e.g., Müllerian agenesis, androgen insensitivity), investigator discretion should be applied to determining study entry.

    Note: Documentation can come from the site personnel's review of the patient's medical records, medical examination, or medical history interview.

Contraception Guidance:

For male patients with a pregnant partner, a condom should be used for contraception in addition to one of the highly effective contraception methods described below.

For male patients with a nonpregnant female partner of childbearing potential or a female patient of childbearing potential, one of the following highly effective birth control methods with a failure rate of less than 1% per year must be applied:

- Combined estrogen- and progestogen-containing hormonal contraception associated with inhibition of ovulation given PO, intravaginally, or transdermally
- Progestin-only hormonal contraception associated with inhibition of ovulation given PO, by injection, or by implant
- Intrauterine device (IUD)
- Intrauterine hormone-releasing system (IUS)
- Bilateral tubal occlusion or ligation

- Vasectomized partner
- Sexual abstinence

In the context of this protocol, sexual abstinence is considered a highly effective method only if defined as refraining from heterosexual intercourse during the entire period specified above. Periodic abstinence (calendar, symptothermal, post-ovulation methods) and withdrawal (coitus interruptus) are not acceptable methods of contraception.

The reliability of sexual abstinence needs to be evaluated in relation to the duration of the clinical trial and the preferred and usual lifestyle of the subject.

If a male subject or male partner of a female subject uses a condom during intercourse, they must also use one of the methods above. Using a condom by itself is NOT considered a highly effective method of contraception.

Collection of Pregnancy Information

Male Patients With Partners Who Become Pregnant

- The investigator will attempt to collect pregnancy information on any male patient's female partner who becomes pregnant while the male patient is in this study. This applies only to male patients who receive DAY101.
- After obtaining the necessary signed informed consent from the pregnant female partner directly, the investigator will record pregnancy information on the appropriate form and submit it to the Sponsor within 24 hours of learning of the partner's pregnancy. The female partner will also be followed to determine the outcome of the pregnancy. Information on the status of the mother and child will be forwarded to the Sponsor. Generally, the follow-up will be no longer than six months following the estimated delivery date. Any termination of the pregnancy will be reported regardless of fetal status (presence or absence of anomalies) or indication for the procedure.

Female Patients Who Become Pregnant

- The investigator will collect pregnancy information on any female patient who becomes pregnant while participating in this study. The initial information will be recorded on the appropriate form and submitted to the Sponsor within 24 hours of learning of the patient's pregnancy.
- The patient will be followed to determine the outcome of the pregnancy. The investigator will collect follow-up information on the patient and the neonate and the information will be forwarded to the Sponsor. Generally, follow-up will not be required for longer than six months beyond the estimated delivery date. Any termination of pregnancy will be reported, regardless of fetal status (presence or absence of anomalies) or indication for the procedure.

- While pregnancy itself is not considered to be an AE or SAE, any pregnancy complication or elective termination of a pregnancy for medical reasons will be reported as an AE or SAE.
- A spontaneous abortion (occurring at < 22 weeks gestational age) or still birth (occurring at > 22 weeks gestational age) is always considered to be an SAE and will be reported as such.
- Any post-study pregnancy-related SAE considered reasonably related to the study intervention by the investigator will be reported to the Sponsor as described in Section 8.1.5. While the investigator is not obligated to actively seek this information in former study patients, he or she may learn of an SAE through spontaneous reporting.
- Any female patient who becomes pregnant while participating in the study will discontinue study intervention.
