# Supplementary material for: The type II RAF inhibitor tovorafenib in relapsed/refractory pediatric low-grade glioma: the phase 2 FIREFLY-1 trial
Source: Nat Med. 2023 Nov 17;30(1):207–17. doi: 10.1038/s41591-023-02668-y (PMC10803270; doi:10.1038/s41591-023-02668-y)
Supplement: Supplementary file 2 — Reporting Summary [file 41591_2023_2668_MOESM2_ESM.pdf]

nature portfolio

Corresponding author(s): Lindsay B. Kilburn, MD

Last updated by author(s): Nov 9, 2023

Reporting Summary

Nature Portfolio wishes to improve the reproducibility of the work that we publish. This form provides structure for consistency and transparency in reporting. For further information on Nature Portfolio policies, see our Editorial Policies and the Editorial Policy Checklist.

Statistics

For all statistical analyses, confirm that the following items are present in the figure legend, table legend, main text, or Methods section.

n/a	Confirmed	
☐	☒	The exact sample size (n) for each experimental group/condition, given as a discrete number and unit of measurement
☐	☒	A statement on whether measurements were taken from distinct samples or whether the same sample was measured repeatedly
☐	☒	The statistical test(s) used AND whether they are one- or two-sided<br>*Only common tests should be described solely by name; describe more complex techniques in the Methods section.*
☒	☐	A description of all covariates tested
☐	☒	A description of any assumptions or corrections, such as tests of normality and adjustment for multiple comparisons
☐	☒	A full description of the statistical parameters including central tendency (e.g. means) or other basic estimates (e.g. regression coefficient) AND variation (e.g. standard deviation) or associated estimates of uncertainty (e.g. confidence intervals)
☒	☐	For null hypothesis testing, the test statistic (e.g. F, t, r) with confidence intervals, effect sizes, degrees of freedom and P value noted<br>*Give P values as exact values whenever suitable.*
☒	☐	For Bayesian analysis, information on the choice of priors and Markov chain Monte Carlo settings
☒	☐	For hierarchical and complex designs, identification of the appropriate level for tests and full reporting of outcomes
☒	☐	Estimates of effect sizes (e.g. Cohen's d, Pearson's r), indicating how they were calculated

Our web collection on statistics for biologists contains articles on many of the points above.

Software and code

Policy information about availability of computer code

Data collection	Clinical data required by the protocol were entered into the Electronic Case Report Forms (eCRF) and used a fully validated secure web-enabled Electronic Data Capture (EDC) System – Medidata Classic Rave® 2022.3.2, which is compliant with 21 CRF Part 11 requirements. Automatic validation edit checks in EDC and offline listings were programmed to capture data discrepancies in the eCRFs and allowed modification and validations of the entered data. The Investigator verified and signed off the eCRFs in EDC to confirm the clinical data captured were complete and accurate. The Sponsor can attest that all data and metadata will be archived in perpetuity. The data are in the EDC (Electronic Data Capture) and TMF (Trial Master File), which are retained in perpetuity. In addition, these data have been filed with the US FDA.
Data analysis	All the data analyses were performed according to statistical analysis plan using SAS v9.4.

For manuscripts utilizing custom algorithms or software that are central to the research but not yet described in published literature, software must be made available to editors and reviewers. We strongly encourage code deposition in a community repository (e.g. GitHub). See the Nature Portfolio guidelines for submitting code & software for further information.

Data

Policy information about availability of data

All manuscripts must include a data availability statement. This statement should provide the following information, where applicable:

- Accession codes, unique identifiers, or web links for publicly available datasets
- A description of any restrictions on data availability
- For clinical datasets or third party data, please ensure that the statement adheres to our policy

The trial protocol (confidential information redacted) has been provided in the Supplementary Information. The authors declare that all data supporting the findings of this trial are available within the article and Supplementary Information. Requests for full datasets will be considered after completion of the trial and analysis of the data, which is anticipated to be December 2024. To request individual participant data associated with any Day One Biopharmaceuticals clinical trial, please email clinical@dayonebio.com. All requests will be evaluated within 8 weeks.

Research involving human participants, their data, or biological material

Policy information about studies with human participants or human data. See also policy information about sex, gender (identity/presentation), and sexual orientation and race, ethnicity and racism.

Reporting on sex and gender	The trial recruited male and female participants 6 months of age to 25 years of age, inclusive. Most participants in the registrational arm (Arm 1) plus Arm 2 were male (53%). No specific gender analyses were completed.<br><br>Sex and/or gender was not considered in the trial design as no sex differences have been seen in previous clinical trials in pLGG, though generally cancers in childhood trend a bit more towards males. pLGG appears to be consistent with this based on a single publication (Gnekow AK, et al. Neuro-Oncol. 2012;14(10):1265-1284) showing a slight male preponderance for incidence of pLGG. But there is no published data to indicate a sex-based difference in response to therapy. As such, the FIREFLY-1 trial recruited any trial-eligible patient independent of sex or gender. The sex of the participants was based on either parental or self-report. The gender of patients was not captured in our case report forms, nor was it considered as part of this trial, and as a result, no specific gender analyses were completed. Three years ago, when protocol was being designed and the FIREFLY-1 trial initiated, there was far less of a focus than at present on collecting gender-related information in pediatric oncology studies. We hope this information is prospectively collected in future pediatric oncology clinical trials.
Reporting on race, ethnicity, or other socially relevant groupings	Participants from all races, ethnicities, and other socially relevant groupings were eligible to participate. Most participants in arms 1 and 2 were white (58%). No other specific race, ethnicity, or other specially relevant groupings were completed.
Population characteristics	Arm 1 (pivotal, low-grade glioma): Patients aged 6 months to 25 years, inclusive, with relapsed or progressive low-grade glioma harboring an activating BRAF alteration, including BRAF V600 mutations and KIAA1549:BRAF fusions. Arm 2 (expansion cohort, low-grade glioma): Patients aged 6 months to 25 years inclusive, with relapsed or progressive low-grade glioma harboring an activating or expected to be activating RAF alteration.<br><br>Patients received tovorafenib (oral tablet or reconstituted liquid suspension formulation) at the RP2D of 420 mg/m2 (not to exceed 600 mg) once weekly in a 28-day treatment cycle (Days 1, 8, 15, and 22). Patients continued on tovorafenib until radiographic evidence of disease progression as determined by the treating investigator, unacceptable toxicity, decision to enter a “drug holiday” period, patient withdrawal of consent, or death.
Recruitment	Patients 6 months to 25 years of age, inclusive, with recurrent or progressive pediatric low-grade glioma (Arms 1 and 2) and with locally advanced or metastatic solid tumors harboring an activating RAF fusion (Arm 3) who met the eligibility criteria were subsequently enrolled and assigned a patient ID. Patients must have received at least one prior line of systemic therapy and have documented evidence of radiographic progression. At trial entry, patients must have demonstrated adequate cardiac, renal, and hepatic function and a Karnofsky (those 16 years and older) or Lansky (those younger than 16 years) performance score of 50 or greater. Trial inclusion and exclusion criteria [in the "Methods" section] clearly describe the trial population and how a patient was selected.
Ethics oversight	The trial was conducted in compliance with ICH Good Clinical Practice guidelines and ethical principles described in the Declaration of Helsinki. The trial protocol and all amendments were reviewed by the Independent Ethics Committee (IEC) or Institutional Review Board (IRB) for each participating trial center. All patients and/or their legally authorized representative provided written informed consent and pediatric assent before enrollment in the trial, according to local regulations. No direct compensation was provided to patients or families for participating in the trial.<br><br>Trial centers include: Children’s Health Queensland Hospital and Health Service (Queensland, Australia), Children's Hospital at Westmead (Western Sydney, Australia), Perth Children's Hospital (Western Australia, Australia), Royal Children's Hospital (Victoria, Australia), Sydney Children's Hospital (Sydney, Australia), Centre Hospitalier Universitaire Ste Justine (Québec, Canada), Centre Mere-Enfant Soleil du CHU – Pediatric Hemato-Oncology (Québec, Canada), McGill University Health Centre (MUHC) - The Montreal Children's Hospital (MCH) (Québec, Canada), Copenhagen University Hospital - Rigshospitalet (Copenhagen, Denmark), Charite - Campus Virchow Klinikum (Berlin, Germany), Universitatsklinikum Heidelberg (Heidelberg, Germany), Rambam Health Care Campus (Haifa, Israel), Schneider Children's Medical Center of Israel (Petah Tikva, Israel), The Chaim Sheba Medical Center (Tel Aviv, Israel), Prinses Máxima Centrum (Utrecht, Netherlands), Seoul National University Hospital (Seoul, South Korea), Severance Hospital, Yonsei University Health System (Seoul, South Korea), KK Women’s and Children’s Hospital (Singapore), Kinderspital Zürich (Zürich, Switzerland), Great Ormond Street Hospital for Children (GOSH) (London, United Kingdom), The Newcastle Hospitals NHS Trust (Newcastle upon Tyne, United Kingdom), Ann & Robert H.

Lurie Children's Hospital - Oncology (Illinois, United States), Children's Hospital of Philadelphia (Pennsylvania, United States), Children's National Medical Center (The District of Columbia, United States), Dana-Farber Cancer Institute-Medicine (Massachusetts, United States), Duke University Medical Center (North Carolina, United States), NYU Langone Health (New York, United States), Seattle Children's Hospital (Washington, United States), St. Louis Children's Hospital (Missouri, United States), Texas Children's Hospital (Texas, United States), University of Michigan (Michigan, United States), University of Utah (Utah, United States).

Note that full information on the approval of the study protocol must also be provided in the manuscript.

Field-specific reporting

Please select the one below that is the best fit for your research. If you are not sure, read the appropriate sections before making your selection.

☒ Life sciences ☐ Behavioural & social sciences ☐ Ecological, evolutionary & environmental sciences

For a reference copy of the document with all sections, see nature.com/documents/nr-reporting-summary-flat.pdf

Life sciences study design

All studies must disclose on these points even when the disclosure is negative.

Sample size	Approximately 140 patients in total were slated to be enrolled across all treatment arms of this trial, with the arms enrolled as registrational arm (LGG): ca. 60 patients; arm 2 (LGG extension): up to 60; patients. Patients were be considered enrolled when they have ingested a dose of tovorafenib on Cycle 1 Day 1.
Data exclusions	No data exclusions.
Replication	The inclusion of specific tumor histologies supported the reproducibility of the trial.
Randomization	This was a 3-arm, open-label trial.
Blinding	The trial was designed to be an open-label trial. Given the rarity of tumor types included in the trial, it is challenging to design a randomized trial for the included cohorts.

Reporting for specific materials, systems and methods

We require information from authors about some types of materials, experimental systems and methods used in many studies. Here, indicate whether each material, system or method listed is relevant to your study. If you are not sure if a list item applies to your research, read the appropriate section before selecting a response.

Materials & experimental systems

n/a	Involved in the study	
☒	☐	Antibodies
☒	☐	Eukaryotic cell lines
☒	☐	Palaeontology and archaeology
☒	☐	Animals and other organisms
☐	☒	Clinical data
☒	☐	Dual use research of concern
☒	☐	Plants

Methods

n/a	Involved in the study	
☒	☐	ChIP-seq
☒	☐	Flow cytometry
☐	☒	MRI-based neuroimaging

Clinical data

Policy information about clinical studies

All manuscripts should comply with the ICMJE guidelines for publication of clinical research and a completed CONSORT checklist must be included with all submissions.

Clinical trial registration	The trial is registered on clinicaltrials.gov as NCT04775485 and EudraCT as #2020-003657-30.
Study protocol	The full trial protocol (some confidential information redacted) is in the Supplementary Information supporting the article.
Data collection	The trial was conducted at academic centers in 11 countries (Australia, Canada, Denmark, Germany, Israel, Netherlands, Singapore, Switzerland, South Korea, the United Kingdom, and the United States and 32 sites enrolled patients. The enrollment centers were academic medical centers that specialize in cancer treatment. The enrollment centers include Children's Health Queensland Hospital and Health Service (Queensland, Australia), Children's Hospital at Westmead (Western Sydney, Australia), Perth Children's Hospital (Western Australia, Australia), Royal Children's Hospital (Victoria, Australia), Sydney Children's Hospital (Sydney, Australia), Centre Hospitalier Universitaire Ste Justine (Québec, Canada), Centre Mere-Enfant Soleil du CHU – Pediatric Hemato-Oncology (Québec, Canada), McGill University Health Centre (MUHC) - The Montreal Children's Hospital (MCH) (Québec, Canada), Copenhagen

University Hospital - Rigshospitalet (Copenhagen, Denmark), Charite - Campus Virchow Klinikum (Berlin, Germany), Universitatsklinikum Heidelberg (Heidelberg, Germany), Rambam Health Care Campus (Haifa, Israel), Schneider Children's Medical Center of Israel (Petah Tikva, Israel), The Chaim Sheba Medical Center (Tel Aviv, Israel), Prinses Máxima Centrum (Utrecht, Netherlands), Seoul National University Hospital (Seoul, South Korea), Severance Hospital, Yonsei University Health System (Seoul, South Korea), KK Women's and Children's Hospital (Singapore), Kinderspital Zürich (Zürich, Switzerland), Great Ormond Street Hospital for Children (GOSH) (London, United Kingdom), The Newcastle Hospitals NHS Trust (Newcastle upon Tyne, United Kingdom), Ann & Robert H. Lurie Children's Hospital - Oncology (Illinois, United States), Children's Hospital of Philadelphia (Pennsylvania, United States), Children's National Medical Center (The District of Columbia, United States), Dana-Farber Cancer Institute-Medicine (Massachusetts, United States), Duke University Medical Center (North Carolina, United States), NYU Langone Health (New York, United States), Seattle Children's Hospital (Washington, United States), St. Louis Children's Hospital (Missouri, United States), Texas Children's Hospital (Texas, United States), University of Michigan (Michigan, United States), University of Utah (Utah, United States).

Designated investigator staff entered the information required by the protocol into the electronic Case Report Form (eCRF). The eCRFs were built using fully validated secure web-enabled software that conforms to 21 CRF Part 11 requirements. Automatic validation programs checked for data discrepancies in the eCRFs and allowed for modification or verification of the entered data by the investigator staff. The investigator verified that the data entered into the eCRFs was complete and accurate.The Sponsor can attest that all data and metadata will be archived in perpetuity. The data are in the EDC (Electronic Data Capture) and TMF (Trial Master File), which are retained in perpetuity. In addition, these data have been filed with the US FDA.

The trial began recruiting patients in April 2021 and is ongoing.

Outcomes

Arm 1 (Low-Grade Glioma)
Primary endpoint
The primary endpoint in Arm 1 (LGG) was independent radiology review committee (IRC)-assessed overall response rate (ORR), defined as the proportion of patients with best overall confirmed response of complete response (CR) or partial response (PR), according to Response Assessment in Neuro-Oncology (RANO)-high grade glioma (HGG) criteria.

Select secondary endpoints
Some of the efficacy-related secondary endpoints in Arm 1 (LGG) included IRC-assessed ORR based on Response Assessment in Pediatric Neuro-Oncology (RAPNO)–LGG criteria, IRC-assessed progression-free survival (PFS), duration of response (DOR), time to response (TTR), and clinical benefit rate (CBR) (BOR of CR, PR, or stable disease [SD] of any length of time or ≥12 months), based on RANO-HGG and RAPNO-LGG criteria. The safety and tolerability of tovorafenib was also assessed by type, frequency, and severity of AEs and by evaluating the effect of tovorafenib on the QT interval corrected for heart rate by Fridericia's formula (QTcF) prolongation and electrocardiogram (ECG) parameters. The remaining secondary endpoints in Arm 1 (LGG) are described in detail in the full trial protocol (some confidential information redacted) is in the Supplementary Information supporting the article.

Select exploratory endpoints
A key exploratory endpoint in Arm 1 (LGG) was IRC-assessed ORR and TTR by RANO-LGG criteria based on the prior line of therapy. The remaining exploratory endpoints in Arm 1 (LGG) are described in detail in the full trial protocol (some confidential information redacted) is in the Supplementary Information supporting the article.

Arm 2 (Low-Grade Glioma Extension)
Primary endpoint
The primary endpoint in Arm 2 (LGG) is assessing the safety and tolerability tovorafenib by looking at type, frequency, and severity of AEs and laboratory abnormalities.

Secondary and exploratory endpoints
The secondary and exploratory endpoints in Arm 2 (LGG extension) are described in detail in the full trial protocol (some confidential information redacted) is in the Supplementary Information supporting the article.

Magnetic resonance imaging

Experimental design

Design type

A phase 2, multicenter, open-label, trial of tovorafenib monotherapy utilizing a central imaging laboratory. A central imaging laboratory was used. Imaging Endpoints (IE) (Scottsdale, AZ) is a research and imaging core laboratory providing blinded independent central review of response assessments with dual reader plus adjudication paradigm utilizing neuro-radiologists trained in in all three response assessment criteria as readers for the following assessments: RANO-HGG criteria (Wen PY, et al. J Clin Oncol. 2010;28(11):1963-1972), RAPNO-LGG criteria (Fangusaro J, et al. Lancet Oncol. 2020;21(6):e305–316), and RANO-LGG criteria (Wen PY, et al. J Clin Oncol. 2017;35(21):439-2449). All activities at IE meet or exceed GCP standards, and IE underwent GCP audit by the Sponsor. A prospectively designed imaging charter was developed for the FIREFLY-1 trial prior to the initiation of the trial. This outlined the processes for initial imaging review, data transfers, and data review and queries were followed throughout the trial.

Design specifications

IE functions as the centralized imaging core lab responsible for the collection, quality control, archival and BICR of imaging for the FIREFLY-1 trial. IE is responsible for management of the image analysis system, reporting methods, implementation of the analysis criteria, and reader management including qualification, training, and oversight.

Behavioral performance measures

Reader performance was assessed by evaluating reader variability at defined and prespecified milestones during ongoing imaging interpretation. Variability metrics included inter- and intra-reader variability to monitor for consistency of reads. If reader acceptance rate fell outside the caution or alert limits, IE determined the appropriate unbiased action(s).

Acquisition

Imaging type(s): MRI – brain tumor imaging protocol (BTIP), spine

Field strength: 1.5 Tesla – 3.0 Tesla scanner

Sequence & imaging parameters:

BTIP:
• Sagittal/axial 3D T1w pre contrast.
• Axial 2D FLAIR (TSE)
• Axial 2D T2w (TSE)
• Axial 2D DWI
• Sagittal/axial 3D T1w post contrast (Gadolinium 0.1 mmol/kg or 0.2 mL/kg (20 mL max.) with a 10 mL saline flush.

Spine:
• Axial pre contrast T1 weighted
• Axial pre-contrast T2 weighted
• Axial post-contrast T1 weighted with fat saturation
• Sagittal T1 weighted
• Sagittal STIR
• Sagittal post-contrast T1

Area of acquisition: Whole brain (foramen magnum to vertex), spine (cervical, thoracic, and lumbar regions).

Diffusion MRI ☒ Used ☐ Not used

Parameters: DWI included as an exploratory endpoint with future analyses planned.

Preprocessing

Preprocessing software: Pre-processing, normalization, noise and artifact removal, and volume censoring were performed by the investigational sites according to local standard practice.

Normalization: Pre-processing, normalization, noise and artifact removal, and volume censoring were performed by the investigational sites according to local standard practice.

Normalization template: Pre-processing, normalization, noise and artifact removal, and volume censoring were performed by the investigational sites according to local standard practice.

Noise and artifact removal: Pre-processing, normalization, noise and artifact removal, and volume censoring were performed by the investigational sites according to local standard practice.

Volume censoring: Pre-processing, normalization, noise and artifact removal, and volume censoring were performed by the investigational sites according to local standard practice.

Statistical modeling & inference

Model type and settings: *Specify type (mass univariate, multivariate, RSA, predictive, etc.) and describe essential details of the model at the first and second levels (e.g. fixed, random or mixed effects; drift or auto-correlation).*

Effect(s) tested: *Define precise effect in terms of the task or stimulus conditions instead of psychological concepts and indicate whether ANOVA or factorial designs were used.*

Specify type of analysis: ☐ Whole brain ☐ ROI-based ☐ Both

Statistic type for inference (See Eklund et al. 2016): *Specify voxel-wise or cluster-wise and report all relevant parameters for cluster-wise methods.*

Correction: *Describe the type of correction and how it is obtained for multiple comparisons (e.g. FWE, FDR, permutation or Monte Carlo).*

Models & analysis

n/a	Involved in the study
☒	☐ Functional and/or effective connectivity
☒	☐ Graph analysis
☒	☐ Multivariate modeling or predictive analysis
